# Structure–activity relationship investigation of tertiary amine derivatives of cinnamic acid as acetylcholinesterase and butyrylcholinesterase inhibitors: compared with that of phenylpropionic acid, sorbic acid and hexanoic acid

**DOI:** 10.1080/14756366.2018.1436053

**Published:** 2018-02-15

**Authors:** Xiaohui Gao, Jingjing Tang, Haoran Liu, Linbo Liu, Lu Kang, Wen Chen

**Affiliations:** a Key Laboratory Breeding Base of Hu’nan Oriented Fundamental and Applied Research of Innovative Pharmaceutics, College of Pharmacy, Changsha Medical University, Changsha, China;; b College of Chemistry and Chemical Engineering, Hu’nan University, Changsha, China;; c Department of Pharmacy, Huizhou Health Sciences Polytechnic, Huizhou, China

**Keywords:** Cinnamic acid, tertiary amine, AChE inhibitors, benzene ring, double bond

## Abstract

In the present investigation, 48 new tertiary amine derivatives of cinnamic acid, phenylpropionic acid, sorbic acid and hexanoic acid (**4d**–**6g**, **10d**–**12g**, **16d**–**18g** and **22d**–**24g**) were designed, synthesized and evaluated for the effect on AChE and BChE *in vitro*. The results revealed that the alteration of aminoalkyl types and substituted positions markedly influences the effects in inhibiting AChE. Almost of all cinnamic acid derivatives had the most potent inhibitory activity than that of other acid derivatives with the same aminoalkyl side chain. Unsaturated bond and benzene ring in cinnamic acid scaffold seems important for the inhibitory activity against AChE. Among them, compound **6g** revealed the most potent AChE inhibitory activity (IC_50_ value: 3.64 µmol/L) and highest selectivity over BChE (ratio: 28.6). Enzyme kinetic study showed that it present a mixed-type inhibition against AChE. The molecular docking study suggested that it can bind with the catalytic site and peripheral site of AChE.

## Introduction

Chalcone, as a special flavone containing an α,β-unsaturated carbonyl group, had demonstrated a wide variety of bioactivities, including anti-cancer, anti-inflammatory, anti-diabetic, cancer chemopreventive, anti-oxidant, anti-microbial activities and enzyme inhibition activities^1^. A few of investigations reported chalcone derivatives as monoamine oxidase inhibitors^2^, glucosidase inhibitors^3^, xanthine oxidase inhibitors^4^ or carbonic anhydrase inhibitors^5^, respectively. It suggested that α,β-unsaturated carbonyl group in chalcone scaffold possible contribute its activity markedly^6^^,^^7^.

In our laboratory, interestingly, several Mannich base derivatives of Flavokawain B with chalcone scaffold were found with AChE inhibitory activity after screening more than one hundred natural products^8^. Then a series of other chalcone nitrogen-containing derivatives or the analogs were synthesized and also appeared potent AChE inhibitory activity^9–12^. Based on these investigations, cinnamic acid, a natural compound also containing an α,β-unsaturated carbonyl group, was selected to study in order to develop potential new AChE inhibitors and elucidate the structure–activity relationship. Cinnamic acid and its derivatives are widely used in food^13^, fragrance material^14^, cosmetics^15^ and drugs. It is applied as the scaffold of some drugs in clinic such as *Cinepazide*^16^*, Tranilast*^17^*, Ilepcimide*^18^, etc. It is also employed to be as lead compound or starting material in a few medicinal chemistry investigations.

**Figure 1.**
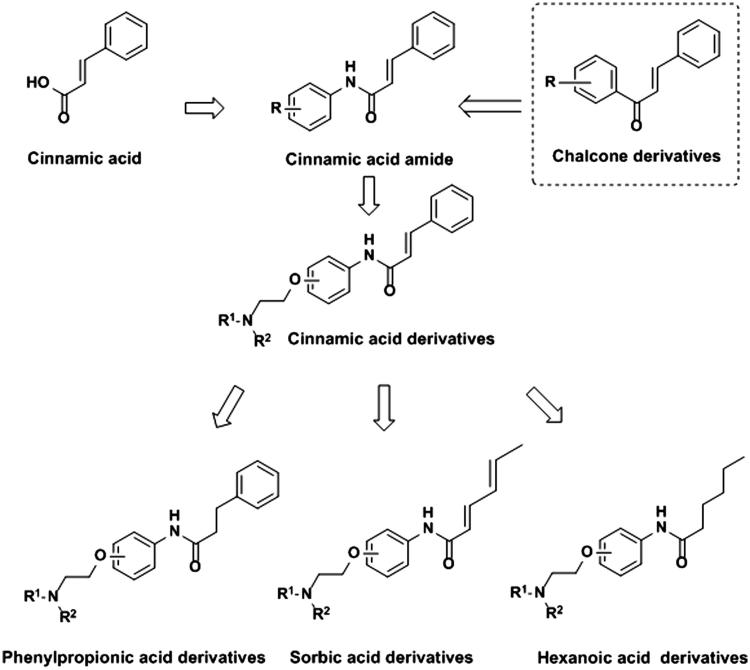
Design strategy of cinnamic acid, phenylpropionic acid, sorbic acid and hexanoic acid derivatives.

In the present investigation, a series of cinnamic acid derivatives containing aminoalkyl side chains with different substituent position (*Ortho, Meta* or *Para*) were designed and synthesized at the beginning. In order to further study the possible influence of unsaturated bond or benzene ring on enzyme inhibition, phenylpropionic acid, sorbic acid and hexanoic acid derivatives were synthesized and evaluated in inhibiting AChE, compared with that of cinnamic acid derivatives (Figure 1). Then the kinetic experiments and the molecular docking studies were conducted to explore the binding mode of the compound and AChE.

## Materials and methods

### General procedure for the synthesis of amide derivatives (3a–3c, 9a–9c, 15a–15c and 21a–21c)

Cinnamic acid, phenylpropionic acid, sorbic acid or hexanoic acid (10 mmol) were added into CH_2_Cl_2_ (40 ml), followed by the addition of oxalyl chloride (5 ml, 60 mmol) and a little of dimethylformamide (DMF) as catalyst. The mixture was stirred for 30 min at 40 °C. After the solvent and excess oxalyl chloride was removed by the evaporation under reduced pressure, the acyl chlorides were gained. These chloride derivatives were used as the initial materials to conduct subsequent reactions without further purification.

Aminophenol (2-aminophenol, 3-aminophenol, or 4-aminophenol, 11 mmol) was added to the acetonitrile solution (20 ml) containing acyl chlorides (10 mmol), then the mixture was refluxed and stirred for 7–10 h until the reaction was completed. After the solvent was removed under vacuum, the crude product was added into 20% sodium hydroxide solution. The solution was cooled to 5–6 °C and filtered. The filtrate was slowly added into 10% cold hydrochloric acid solution, followed by adjusting the solution to pH = 3–4. Then gray or white precipitates were gained with the yield of 85–95%. The intermediates mentioned above are known compounds but with no reports about bioactivity in inhibiting AchE and BChE^19–22^.

### General procedure for the synthesis of compounds 4d–6g, 10d–12g, 16d–18g and 22d–24g

The aminoethyl chloride (4.5 mmol) were added into a solution containing compounds **3a**–**3c**, **9a**–**9c**, **15a**–**15c** or **21a**–**21c** (1.5 mmol), K_2_CO_3_ (1.055 g, 7.5 mmol) and NaI (0.012 g, 0.05 mmol) in acetone (20 ml), respectively. The reaction mixture was refluxed and stirred for 10***–***15 h, then cooled to room temperature. After the mixture was filtered, the filtrate was concentrated under reduced pressure and partitioned between brine and CH_2_Cl_2_. The separated CH_2_Cl_2_ layer was washed with 10% NaOH, dried over by anhydrous Na_2_SO_4_. The solvent was removed in vacuum and the desired compounds were gained with 50–70% yield through silica gel column chromatography.

Compounds **4d** and **6d** are known compounds but without reports about bioactivity^23^. Experimental details for the characterization of compounds can be found in the Supplemental materials.

### AChE and BChE inhibition assay

The effects of the newly synthesized compounds on AChE/BChE were assayed by *Ellman* method with slight modification^24^. The brain and serum of Sprague–Dawley rat was used as the resource of AChE and BChE, respectively. The individual compound was dissolved in Tween 80 (final concentration was 0.06% in each reaction) and diluted with water to various concentrations immediately before use. Five different concentrations were tested for each compound in triplicate. The reaction mixture contained 2.70 ml Na_2_HPO_4_/NaH_2_PO_4_ buffer (pH = 7.4), 100 μL of the different concentrations of test compounds, 100 μL AChE or BChE (0.04 U/100 μL) and 100 μL 15 mmol/L acetylthiocholine iodide/S-butyrylthiocholine iodide, the mixture was incubated for 30 min. Then, the reaction was terminated by the addition of 100 μL 20% sodium dodecylsulfate (SDS), then 100 μL 10 mmol/L 5,5′-dithiobis-(2-nitrobenzoic acid) (DTNB) was added to generate the yellow anion 5-thio-2-nitrobenzoic acid. The absorbance of each assay mixture was measured at 412 nm by UV–Vis spectroscopy. The IC_50_ values were calculated by the Bliss method and expressed as Mean ± SD. All assays were conducted in triplicate. Rivastigmine was used as the control drug.

### Kinetic study

Kinetic characterization of AChE was performed by a reported method^25^. Compound **6g** was added into the assay solution and preincubated with the enzyme at 30 °C, followed by the addition of 100 μL acetylthiocholine iodide including five concentrations. The assay solution contained 100 μL compound **6g**, 100 μL DTNB, 2.79 ml 0.1 mol/l Na_2_HPO_4_/NaH_2_PO_4_ buffer (pH 7.4). Kinetic profile of the hydrolysis of acetylthiocholine iodide catalyzed by AChE was determined by the UV–Vis spectrometry at 412 nm. The parallel control experiment was carried out without compound **6g** in the mixture.

### Molecular docking

Molecular docking studies were carried out with Molecular Operating Environment (MOE) software package (Chemical Computing Group, Montreal, Canada), and structure models of AChE/BChE X-ray crystal structures (PDB ID: 1EVE/1P0I)^26^ were gained from protein data bank. The 3D structures of compounds were built with virtue of the builder interface of MOE program, and docked into the active site of the protein after energy being minimized. The dock scoring in MOE software was done by ASE scoring function.

### Log P and pKa assay

Log P is defined as the logarithm of octanol–water partition coefficient. In the present investigation, log P of compounds **4d∼24g** was measured by the shake flask method, and PBS (pH = 7.4) was used as the water phase^27^. Ultraviolet spectrophotometry was applied to determine the concentration of compounds. Log *p* values were calculated based on the data in triplicate.

pKa is determined by pH-titration method according to the report^28^. The titration curve E vs. VT was recorded and pKa value was calculated.

## Results and discussion

### Chemistry

**Scheme 1.**
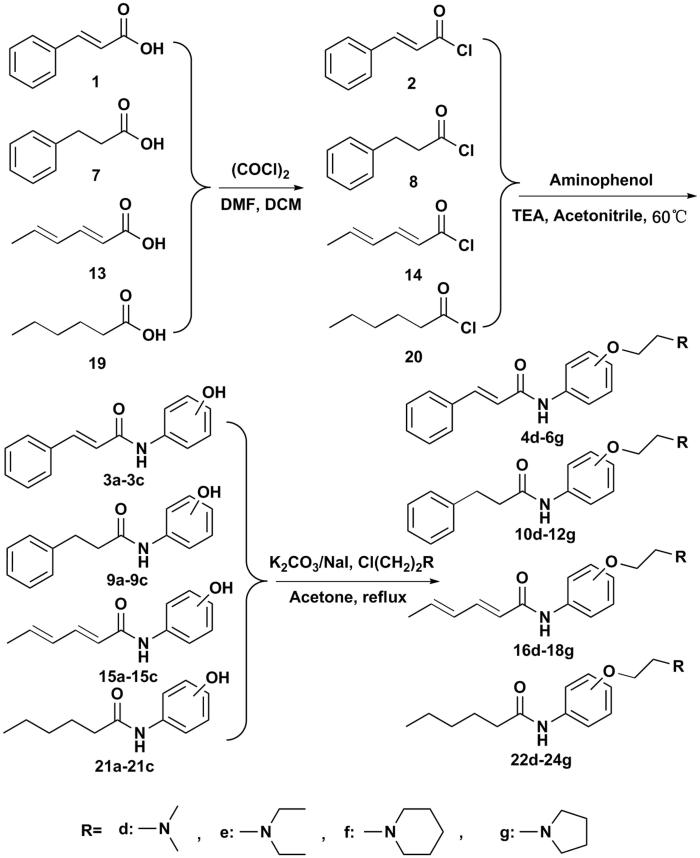
Synthesis pathway of cinnamic acid, phenylpropionic acid, sorbic acid and hexanoic acid derivatives.

The amide compounds were synthesized as followed. Briefly, the amide compounds were prepared by the condensation reaction of compounds **1**, **7**, **13** or **19** with oxalyl chloride using DMF as the catalyst. Then, the acyl chloride was reacted with the aminophenol to synthesize amide derivatives **3a**–**3c**, **9a**–**9c**, **15a**–**15c** and **21a**–**21c**, respectively, which were subsequently reacted with four commercially available aminoethyl chlorides (chloroethyldimethylamine hydrochloride, chloroethyldiethylamine hydrochloride, chloroethylpiperidinehydrochloride and chloroethylpyrrolidine hydrochloride) in the presence of K_2_CO_3_ and NaI to gain compound **4d**–**6g**, **10d**–**12g**, **16d**–**18g** and **22d**–**24g** (Scheme 1). The structures of these compounds were characterized by proton nuclear magnetic resonance spectroscopy (^1^H NMR), infrared spectrum (IR) and mass spectrometry (MS). The purity of all synthesized compounds was determined by HPLC.

### AChE and BChE inhibition effects

As shown in Table 1, it seemed that the variation of aminoalkyl position significantly influences the inhibitory potency against AChE. Except for compounds **18e** and **24e**, almost all compounds with *para*-substituted aminoalkyl had more potent inhibitory activity than the ones with *meta*- or *ortho*-substituted aminoalkyl, while most compounds with *ortho*-substituted aminoalkyl but not compounds **16d** and **22e** had poorer inhibitory activity against AChE than those compounds with *meta-* or *para*-substituted aminoalkyl. In addition, most compounds with *para*-substituted aminoalkyl but not compounds **18e** and **24e** revealed the higher selectivity in inhibiting AChE over BChE than the ones with *meta*- or *ortho*-substituted aminoalkyl.

**Table 1.** The effects in inhibiting AChE and BChE, and logp, pKa of new synthesized tertiary amine derivatives.

Compounds	R	Position	IC_50_ (µmol/L,Mean ± SD)		Selectivity for AChE^a^	Log P	p*Ka*
			AChE	BChE			
**4d**		*Ortho*	161.03 ± 2.19	11.20 ± 0.34	0.07	2.78	6.96
**5d**		*Meta*	20.49 ± 3.13	26.55 ± 0.56	1.30	0.96	6.51
**6d**		*Para*	4.98 ± 0.31	113.36 ± 1.36	22.8	1.54	7.18
**10d**		*Ortho*	284.73 ± 4.69	36.61 ± 1.38	0.13	2.14	7.19
**11d**		*Meta*	59.16 ± 3.34	15.53 ± 1.21	0.26	1.19	6.45
**12d**		*Para*	46.85 ± 1.29	132.17 ± 5.21	2.82	2.44	7.26
**16d**		*Ortho*	30.68 ± 2.34	34.11 ± 1.85	1.11	0.63	7.25
**17d**		*Meta*	52.47 ± 0.39	50.43 ± 0.46	0.96	0.99	7.23
**18d**		*Para*	12.64 ± 0.89	221.59 ± 8.63	17.5	1.79	7.21
**22d**		*Ortho*	185.84 ± 8.22	26.63 ± 1.36	0.14	1.23	7.32
**23d**		*Meta*	60.62 ± 2.18	7.89 ± 0.89	0.13	0.37	6.52
**24d**		*Para*	41.67 ± 2.89	33.78 ± 4.21	0.81	0.72	7.16
**4e**		*Ortho*	197.04 ± 6.81	6.99 ± 0.29	0.04	2.81	7.28
**5e**		*Meta*	49.36 ± 1.91	42.53 ± 0.67	0.86	2.56	7.16
**6e**		*Para*	10.57 ± 0.43	43.99 ± 3.29	4.16	1.22	6.97
**10e**		*Ortho*	>500	40.13 ± 1.29	<0.12	3.22	7.23
**11e**		*Meta*	102.23 ± 3.36	8.23 ± 0.59	0.08	2.23	6.51
**12e**		*Para*	84.63 ± 6.10	211.93 ± 4.87	2.50	2.58	6.52
**16e**		*Ortho*	>500	91.81 ± 1.45	<0.27	2.18	7.53
**17e**		*Meta*	98.74 ± 1.89	79.52 ± 1.43	0.81	1.72	7.58
**18e**		*Para*	189.45 ± 4.55	94.52 ± 4.26	0.50	1.31	7.16
**22e**		*Ortho*	>500	37.19 ± 2.24	<0.07	1.67	7.08
**23e**		*Meta*	124.76 ± 4.23	0.67 ± 0.04	0.01	0.93	7.15
**24e**		*Para*	>500	9.90 ± 1.28	<0.02	1.10	7.48
**4f**		*Ortho*	140.55 ± 7.01	1.16 ± 0.22	0.01	2.17	6.85
**5f**		*Meta*	20.51 ± 1.01	3.26 ± 0.26	0.16	1.24	6.99
**6f**		*Para*	4.35 ± 0.36	94.52 ± 8.74	23.7	1.40	7.29
**10f**		*Ortho*	249.74 ± 8.21	0.59 ± 0.09	0.002	1.18	7.02
**11f**		*Meta*	68.97 ± 2.64	63.95 ± 1.92	0.93	3.22	6.78
**12f**		*Para*	34.26 ± 0.89	226.20 ± 5.78	6.60	1.82	7.13
**16f**		*Ortho*	170.39 ± 4.11	27.74 ± 1.29	0.16	0.67	6.86
**17f**		*Meta*	44.17 ± 1.36	33.64 ± 1.59	0.76	2.23	7.17
**18f**		*Para*	12.25 ± 0.76	270.88 ± 4.56	22.1	1.61	7.06
**22f**		*Ortho*	324.71 ± 7.65	9.04 ± 0.27	0.03	1.11	6.99
**23f**		*Meta*	76.66 ± 3.17	0.22 ± 0.02	0.002	1.55	7.32
**24f**		*Para*	31.68 ± 2.74	17.04 ± 2.03	0.54	1.54	7.16
**4g**		*Ortho*	170.39 ± 8.49	1.56 ± 0.42	0.01	2.19	7.33
**5g**		*Meta*	8.52 ± 1.23	12.90 ± 1.18	1.51	2.28	7.28
**6g**		*Para*	3.64 ± 0.28	104.20 ± 1.34	28.6	1.33	6.74
**10g**		*Ortho*	209.30 ± 2.33	1.87 ± 0.16	0.01	1.30	6.88
**11g**		*Meta*	58.65 ± 3.85	83.95 ± 2.12	1.43	2.58	6.45
**12g**		*Para*	24.41 ± 0.76	98.48 ± 1.09	1.99	2.26	6.80
**16g**		*Ortho*	277.61 ± 4.58	21.88 ± 0.99	0.08	0.97	7.32
**17g**		*Meta*	18.81 ± 0.63	28.46 ± 1.24	1.51	1.41	7.06
**18g**		*Para*	13.64 ± 1.24	117.58 ± 6.79	8.63	1.18	7.73
**22g**		*Ortho*	>500	18.61 ± 0.32	<0.04	1.11	7.16
**23g**		*Meta*	50.73 ± 2.66	0.34 ± 0.01	0.01	1.37	7.69
**24g**		*Para*	16.89 ± 0.91	13.88 ± 1.66	0.82	0.16	6.92
Rivastigmine^b^			10.54 ± 0.86	0.26 ± 0.08	0.02	1.68	5.93

a Selectivity for AChE is defined as IC_50_ (BChE)/IC_50_ (AChE).

b Used as positive control.

The alteration of aminoalkyl side chains also had significant impact on inhibitory activity. In general, the derivatives with diethylamino ethyl side chain had the weaker inhibitory activity than the others. Among all compounds with *meta-* or *para*-substituted aminoalkyl, most derivatives with pyrrolidine ethyl side chain but not compound **18g** had more potent activity than the others in inhibiting AChE, while the derivatives with *ortho*-substituted aminoalkyl did not accord with this manner.

The alteration of compound scaffold also influenced the effect in inhibiting AChE. In general, all cinnamic acid derivatives but not compound **4d** had the most potent inhibitory activity than that of sorbic acid, phenylpropionic acid or hexanoic acid derivatives with the same aminoalkyl side chain. Most sorbic acid derivatives but not compound **16e** are more potent than that of hexanoic acid derivatives, which indicated that double bond was possible important for these compounds to possessing AChE inhibitory activity. Most of cinnamic acid derivatives but not compound **4d** showed more potent effects in inhibiting AChE than that of sorbic acid derivatives. It suggested that benzene ring also had significant influence on the inhibitory activity against AChE.

### Kinetic study

Compound **6g** was selected for further kinetic study since it had the strongest inhibiting activity against AChE among new synthesized compounds. The graphical analysis of the steady-state inhibition data of compound **6g** is shown in Figure 2 Supplement. According to the analysis, the increase of K_m_ and the decline of V_max_ with the increasing concentration of compound **6g** presents the characteristics of mixed-type inhibition. The competitive inhibit constant (K_i_) of compounds **6g** is 7.27 µmol/L, and the non-competitive constant (K_i_′) is 2.14 µmol/L, respectively. This result suggested that compound **6g** could simultaneously bind the active center and other sites of AChE.

### Log P and pKa assay

Log P was thought as an important physical chemistry parameter to predict the ability to cross blood brain barrier (BBB). According to report, optimum log P for central nervous system (CNS) penetration was around 2.0 ± 0.7^29^. Log *p* values of synthesized compounds are presented in Table 1. It was ranged 0.16–3.22. The results suggested that most of these compounds were able to pass BBB with sufficiently lipophilicity.

In another hand, as tertiary amine derivatives, new synthesized compounds present weakly alkaline with pKa ranged 6.45–7.73. It is known that weak basic drugs or compounds are easy to absorb from small intestine and pass the BBB^30^. The results indicated that all new synthesized compounds in present investigation exhibit excellent potency in absorb ability.

### Molecular docking

To explore the molecular mechanism of compound **6g** to inhibit AChE and BChE, molecular docking was performed with software MOE2008. As shown in Figure 3 Supplement, compound **6g** exhibited multiple points binding modes with AChE (−31.15 kJ/mol) (Figure 3A, Supplement). In the top of the gorge, the aromatic moiety adopted an appropriate orientation for its binding to peripheral anionic site (PAS), via the π–π stacking interaction with Trp279 (4.36 Å) and Tyr334 (3.66 Å).In the bottom of the gorge, the charged nitrogen of pyrrolidine ring was observed to bind to catalytic active sites (CAS) via a π–cation interaction with Trp84 (4.34 Å) and Phe330 (2.82 Å). However, compound **6g** could only binds to BChE (−13.19 kJ/mol) (Figure 3B, Supplement) via Trp82 (3.76 Å) with a π–π interaction. These results may provide partial explain for its potent and high selective inhibition against AChE over BChE.

## Conclusions

The present investigation on the effects of cinnamic acid, phenylpropionic acid, sorbic acid or hexanoic acid derivatives in inhibiting AChE revealed that the alteration of aminoalkyl types and the substituted position markedly influenced the inhibitory activity. The unsaturated bond and aromatic ring of cinnamic acid derivatives are seemed important for AChE inhibitory activity. These results provide valuable information for the development of potent and/or selective AChE inhibitors in the future.

## Disclosure statement

The authors have declared no conflict of interest.

## Supplementary Material

IENZ_1436053_Supplementary_Material.pdf
