# Supplementary material for: Structure–activity relationship investigation of tertiary amine derivatives of cinnamic acid as acetylcholinesterase and butyrylcholinesterase inhibitors: compared with that of phenylpropionic acid, sorbic acid and hexanoic acid
Source: J Enzyme Inhib Med Chem. 2018 Feb 15;33(1):519–24. doi: 10.1080/14756366.2018.1436053 (PMC6010128; doi:10.1080/14756366.2018.1436053)
Supplement: IENZ_1436053_Supplementary_Material.pdf [file IENZ_A_1436053_SM6792.pdf]

Supplemental materials

Experimental

General

All chemicals and reagents were of analytical reagent grade and used without further purification. The melting points were measured on a WRS-lA melting point detector. 1H NMR spectra were recorded by a Bruker 400 MHz instrument (Bruker Nano, Inc., Billerica, MA) in DMSO-d6 with tetramethylsilane (TMS) as the internal standard. High resolution Mass spectra (HRMS) were obtained on Waters XEVO-G2XSQTOF Liquid chromatography-mass spectrometry with electrospray ionization (ESI-MS) method. Infrared spectrum was obtained from Shimadzu Infinity-1 infrared spectrometer (Shimadzu Corporation, Kanagawa, Japan). The purity of compounds was checked by Shimadzu LC-20A high performance liquid chromatography (Shimadzu Corporation, Kanagawa, Japan).

N-(2-(2-(dimethylamino)ethoxy)phenyl)cinnamamide (4d)

Compound **4d** is a known compound but with no bioactivity reports.

N-(2-(2-(diethylamino)ethoxy)phenyl)cinnamamide (4e)

White solid, 0.21 g, yield 49.3%, mp: 80.3-81.2℃; ^{1}H NMR (400 MHz, DMSO-d_6) δ (ppm): 0.99 (6H，t，J = 12.0 Hz，2× NCH$_2$C<u>H</u>$_3$), 2.57-2.58 (4H, m, 2× NC<u>H</u>$_2$CH$_3$), 2.81 (2H, t, J = 6.0 Hz, OCH$_2$C<u>H</u>$_2$), 4.12 (2H, t, J = 6.0 Hz, OC<u>H</u>$_2$CH$_2$), 6,96 (1H, t, J = 8.0 Hz，Ar-H), 7.04 (1H, d, J = 16.0 Hz, Ar-CH=C<u>H</u>), 7.10 (2H，d，J = 8.0 Hz，Ar-H)，7.39-7.47 (3H，m，Ar-<u>H</u>)，7.58 (1H, d, J = 16.0 Hz, Ar-C<u>H</u>=CH)， 7.64 (2H， d， J = 8.0 Hz， Ar-<u>H</u>)， 8.14 (1H， d， J = 8.0 Hz， Ar-<u>H</u>)，9.29 (1H，s，NH). IR (KBr) m/cm^{-1}: 3244, 2970, 1681, 1627, 1598, 1251, 1170, 867, 78. HRMS m/z (ESI): 339.2085 [M+H]$^+$. calcd for $C_{21}H_{26}N_2O_2$: 338.2, Purity: 98.1% by HPLC (MeOH/0.1% TEA 85:15 (v/v)).

N-(2-(2-(piperidin-1-yl)ethoxy)phenyl)cinnamamide (4f)

Yellow solid, 0.29 g, yield 55.6%, mp: 83.8-85.6℃; ^1H NMR (400 MHz, DMSO-d_6) δ (ppm): 1.35-1.37 (2H，m，piperidine-H)，1.47-1.50 (4H，m，piperidine-H)，2.45 (4H，m，piperidine-H)，2.71 (2H，t，J = 6.0 Hz，OCH_2CH_2)，4.17 (2H，t，J = 6.0 Hz，OCH_2CH_2)，6.95 (1H，t，J = 8.0 Hz，Ar-H)，7.05-7.13 (3H，m，Ar-CH=CH and Ar-H)，7.39-7.47 (3H，m，Ar-H)，7.57 (1H，d，J = 16.0 Hz，Ar-CH=CH)，7.65 (2H，d，J = 8.0 Hz，Ar-H)，8.14 (1H，d，J = 8.0 Hz，Ar-H)，9.23 (1H，s，NH). IR (KBr) m/cm^{-1}: 3296, 2967, 1668, 1624, 1597, 1251, 1184, 867, 788. HRMS m/z (ESI): 351.2060 $[M+H]^+$, calcd for $C_{22}H_{26}N_2O_2$: 350.2, Purity: 98.5% by HPLC (MeOH/0.1% TEA 85:15 (v/v)).

N-(2-(2-(pyrrolidin-1-yl)ethoxy)phenyl)cinnamamide (4g)

Yellow solid, 0.30 g, yield 59.1%, mp: 78.1-79.3℃; ^1H NMR (400 MHz, DMSO-d_6) δ (ppm): ^1H NMR (400 MHz, DMSO-d_6) δ (ppm): 1.69 (4H，m，pyrrolidine-H)，2.56 (4H，m，pyrrolidine-H)，2.82 (2H，t，J = 6.0 Hz，OCH_2CH_2)，4.19 (2H，t，J = 6.0 Hz，OCH_2CH_2)，6.97 (1H，t，J = 8.0 Hz，Ar-H)，7.04 (1H, d, J = 16.0 Hz，Ar-CH=CH), 7.08-7.13 (2H，m，Ar-H)，7.41-7.47 (3H，m，Ar-H)，7.57 (1H，d，J = 16.0 Hz，Ar-CH=CH)，7.65 (2H，d，J = 8.0 Hz，Ar-H)，8.16 (1H，d，J = 8.0 Hz，Ar-H)，9.39 (1H，s，NH). IR (KBr) m/cm^{-1}: 3230, 3026, 1667, 1622, 1597, 1249, 1163, 866, 761. HRMS m/z (ESI): 337.1937 $[M+H]^+$, calcd for $C_{21}H_{24}N_2O_2$: 336.2, Purity: 98.2% by HPLC (MeOH/0.1% TEA 85:15 (v/v)).

N-(3-(2-(dimethylamino)ethoxy)phenyl)cinnamamide (5d)

Light yellow viscous liquid, yield 58.6%; ^1H NMR (400 MHz, DMSO-d_6) δ (ppm): 2.50 (6H，s，2× NCH_3)，2.99 (2H，t，J = 6.0 Hz，OCH_2CH_2)，4.15 (2H，t，J = 6.0 Hz，OCH_2CH_2)，6.69 (1H，d，J = 8.0 Hz，Ar-H)，6.83 (1H，d，J = 16.0 Hz，Ar-CH=CH)，7.18 (1H，d，J = 8.0 Hz，Ar-H)，7.25 (1H，t，J = 8.0 Hz，Ar-H)，7.42-7.47 (3H，m，Ar-H)，7.51 (1H，s，Ar-H)，7.58 (1H，d，J = 16.0 Hz，Ar-CH=CH)，7.63 (2H，d，J = 8.0 Hz，Ar-H)，10.23 (1H，s，NH). IR (KBr) m/cm^{-1}: 3253, 3026, 1668, 1633, 1598, 1284, 1257, 977, 711. HRMS m/z (ESI): 311.1757$[M+H]^+$, calcd for $C_{19}H_{22}N_2O_2$: 310.2. Purity: 98.6% by HPLC

(MeOH/0.1% TEA 85:15 (v/v)).

N-(3-(2-(diethylamino)ethoxy)phenyl)cinnamamide (5e)

Light purple viscous liquid, yield 51.3%; ^{1}H NMR (400 MHz, DMSO-d_6) δ (ppm): 1.03 (6H，m，2× $NCH_2C\underline{H}_3$)，2.60 (4H，m，2× $NC\underline{H}_2CH_3$)，2.82 (2H，t，J = 6.0 Hz，$OCH_2C\underline{H}_2$)，4.04 (2H，t，J =6.0 Hz，$OC\underline{H}_2CH_2$)，6.67 (1H，d，J = 8.0 Hz，Ar-H)，6.82 (1H，d，J = 16.0 Hz，Ar-CH=CH)，7.18 (1H，d，J = 8.0 Hz，Ar-H)，7.24 (1H，t，J = 8.0 Hz，Ar-H)，7.41-7.47 (4H，m，Ar-H)，7.58 (1H，d，J = 16.0 Hz，Ar-CH=CH)，7.63 (2H，d，J = 8.0 Hz，Ar-H)，10.21 (1H，s，NH). IR (KBr) m/cm^{-1}: 3253, 3026, 1662, 1633, 1608, 1286, 1247, 979, 711. HRMS m/z (ESI): 339.2072 [M+H]$^+$. calcd for $C_{21}H_{26}N_2O_2$: 338.2, Purity: 98.3% by HPLC (MeOH/0.1% TEA 85:15 (v/v)).

N-(3-(2-(piperidin-1-yl)ethoxy)phenyl)cinnamamide (5f)

Light yellow viscous liquid, yield 60.3%; ^{1}H NMR (400 MHz, DMSO-d_6) δ (ppm): 1.35-1.37 (2H，m，piperidine-H)，1.47-1.50 (4H，m，piperidine-H)，2.45 (4H，m，piperidine-H)，2.82 (2H，t，J = 6.0 Hz，$OCH_2C\underline{H}_2$)，4.04 (2H，t，J = 6.0 Hz，$OC\underline{H}_2CH_2$)，6.67 (1H，d，J = 8.0 Hz，Ar-H)，6.82 (1H，d，J = 16.0 Hz，Ar-CH=CH)，7.18 (1H，d，J = 8.0 Hz，Ar-H)，7.24 (1H，t，J = 8.0 Hz，Ar-H)，7.41-7.47 (4H，m，Ar-H)，7.58 (1H，d，J = 16.0 Hz，Ar-CH=CH)，7.63 (2H，d，J = 8.0 Hz，Ar-H)，10.21 (1H，s，NH). IR (KBr) m/cm^{-1}: 3259, 3030, 1660, 1633, 1600, 1282, 1159, 976, 700. HRMS m/z (ESI): 351.2062 [M+H]$^+$. calcd for $C_{22}H_{26}N_2O_2$: 350.2, Purity: 98.1% by HPLC (MeOH/0.1% TEA 85:15 (v/v)).

N-(3-(2-(pyrrolidin-1-yl)ethoxy)phenyl)cinnamamide (5g)

Light yellow solid, yield 57.2%, mp: 95.7-97.3℃; ^{1}H NMR (400 MHz, DMSO-d_6) δ (ppm): 1.83 (4H，m，pyrrolidine-H)，2.96 (4H，m，pyrrolidine-H)，3.21 (2H，t，J = 6.0 Hz，$OCH_2C\underline{H}_2$)，4.17 (2H，t，J = 6.0 Hz，$OC\underline{H}_2CH_2$)，6.70 (1H，d，J = 8.0 Hz，Ar-H)，6.84 (1H，d，J = 16.0 Hz，Ar-CH=CH)，7.18 (1H，d，J = 8.0 Hz，Ar-H)，7.26 (1H，t，J = 8.0 Hz，Ar-H)，7.40-7.48 (3H，m，Ar-H)，7.54 (1H，s，Ar-H)，7.58 (1H，d，J = 16.0 Hz，Ar-CH=CH)，7.63 (2H，d，J = 8.0 Hz，Ar-H)，10.23 (1H，s，NH). IR (KBr) m/cm^{-1}: 3261, 3030, 1662,, 1625, 1595, 1290, 1153, 979, 711. HRMS m/z (ESI):337.1908 [M+H]$^+$, calcd for

$C_{21}H_{24}N_2O_2$: 336.2, Purity: 98.1% by HPLC (MeOH/0.1% TEA 85:15 (v/v)).

N-(4-(2-(dimethylamino)ethoxy)phenyl)cinnamamide (6d)

Compound **6d** is a known compound but without reports about bio-activity.

N-(4-(2-(diethylamino)ethoxy)phenyl)cinnamamide (6e)

Light yellow solid product, yield 63.1%, mp: 105.0-106.2℃; ^{1}H NMR (400 MHz, DMSO-d_6) δ (ppm): 2.25 (6H, s, 2× NC$\underline{H}_3$), 2.66 (2H, t, J =6.0 Hz, OCH$_2$C$\underline{H}_2$), 4.03 (2H, t, J =6.0 Hz, OC$\underline{H}_2$CH$_2$), 6.81 (1H, d, J =16.0 Hz, Ar-CH=C$\underline{H}$), 6.90-6.94 (2H, m, Ar-H), 7.38-7.47 (3H, m, Ar-H), 7.58 (1H, d, J =16.0 Hz, Ar-C$\underline{H}$=CH), 7.58-7.63 (4H, m, Ar-H), 10.11 (1H, s, N$\underline{H}$). IR (KBr) m/cm^{-1}: 3296, 2968, 1678, 1625, 1598, 1292, 1172, 858, 789. HRMS m/z (ESI): 339.2083[M+H]$^+$, calcd for $C_{21}H_{26}N_2O_2$: 338.2, Purity: 98.4% by HPLC (MeOH/0.1% TEA 85:15 (v/v)).

N-(4-(2-(piperidin-1-yl)ethoxy)phenyl)cinnamamide (6f)

Light yellow solid, yield 59.8%, mp: 145.7-147.3℃; ^{1}H NMR (400 MHz, DMSO-d_6) δ (ppm): 1.01 (6H，t，J=6.0 Hz，2× NCH$_2$C$\underline{H}_3$)，2.60-2.84 (4H，m，2× NC$\underline{H}_2$CH$_3$)，3.38 (2H，t，J =6.0 Hz，OC$\underline{H}_2$CH$_2$)，4.02 (2H，t，J =6.0 Hz，OC$\underline{H}_2$CH$_2$)，6.81 (1H，d，J =16.0 Hz，Ar-CH=C$\underline{H}$)，6.90-6.94 (2H，m，Ar-H)，7.38-7.49 (3H，m，Ar-H)，7.56 (1H，d，J =16.0 Hz，Ar-C$\underline{H}$=CH)，7.60-7.64 (4H，m，Ar-H)，10.12 (1H，s，N$\underline{H}$). IR (KBr) m/cm^{-1}: 3296, 2933, 1660, 1625, 1598, 1230, 1174, 858, 790. HRMS m/z (ESI): 351.2063 [M+H]$^+$, calcd for $C_{22}H_{26}N_2O_2$: 350.2, Purity: 97.8% by HPLC (MeOH/0.1% TEA 85:15 (v/v)).

N-(4-(2-(pyrrolidin-1-yl)ethoxy)phenyl)cinnamamide (6g)

Light yellow solid, yield 57.3%, mp: 93.2-94.9℃; ^{1}H NMR (400 MHz, DMSO-d_6) δ (ppm): 1.66-1.74 (4H，m，pyrrolidine-H)，2.56 (4H，t，J =6.0 Hz，pyrrolidine-H)，2.82 (2H，t，J =6.0 Hz，OCH$_2$C$\underline{H}_2$)，4.05 (2H，t，J =6.0 Hz，OC$\underline{H}_2$CH$_2$)，6.82 (1H，d，J =16.0 Hz，Ar-CH=C$\underline{H}$)，6.91-6.94 (2H，m，Ar-H)，7.38-7.47 (3H，m，Ar-H)，7.56 (1H，d，J =16.0 Hz，Ar-C$\underline{H}$=CH)，7.61-7.64 (4H，m，Ar-H)，10.11 (1H，s，N$\underline{H}$). IR (KBr) m/cm^{-1}: 3286, 3055, 1681, 1620, 1598, 1294, 1174, 856, 786. HRMS m/z (ESI): 337.1913[M+H]$^+$, calcd for $C_{21}H_{24}N_2O_2$: 336.2, Purity: 98.3% by HPLC (MeOH/0.1% TEA 85:15 (v/v)).

N-(2-(2-(dimethylamino)ethoxy)phenyl)-3-phenylpropanamide (10d)

Light yellow viscous liquid, yield 63.3%; ^{1}H NMR (400 MHz, DMSO-d_6) δ (ppm): 2.27 (6H，s，2× NCH_3)，2.63 (2H，t，J =6.0 Hz，OCH_2CH_2)，2.72 (2H，t，J =8.0 Hz，Ar-CH_2CH_2)，2.98 (2H，t，J =8.0 Hz，Ar-CH_2CH_2)，4.14 (2H，t，J =6.0 Hz，OCH_2CH_2)，6.98-7.02 (1H，m，Ar-H)，7.10-7.15 (2H，m，Ar-H)，7.24-7.26 (1H，m，Ar-H)，7.31-7.36 (4H，m，Ar-H)，8.05 (1H，d，J =8.0 Hz，Ar-H)，9.42 (1H，s，NH). IR (KBr) m/cm^{-1}: 3421, 3032, 1668, 1622, 1598, 1282, 1186, 783. HRMS m/z (ESI): 313.1914[M+H]$^+$, calcd for $C_{19}H_{24}N_2O_2$: 312.2, Purity: 98.2% by HPLC (MeOH/0.1% TEA 85:15 (v/v)).

N-(2-(2-(diethylamino)ethoxy)phenyl)-3-phenylpropanamide (10e)

White solid, yield 71.6%, mp: 50.2～51.7℃; ^{1}H NMR (400 MHz, DMSO-d_6) δ (ppm): 0.96 (6H，t，J =8.0 Hz，2× NCH_2CH_3)，2.52-2.56 (4H，m，2× NCH_2CH_3)，2.67 (2H，t，J =6.0 Hz，OCH_2CH_2)，2.75 (2H，t，J =6.0 Hz，Ar-CH_2CH_2)，2.91 (2H，t，J =6.0 Hz，Ar-CH_2CH_2)，4.04 (2H，t，J =6.0 Hz，OCH_2CH_2)，6.89-6.93 (1H，m，Ar-H)，7.03-7.07 (2H，m，Ar-H)，7.16-7.20 (1H，m，Ar-H)，7.24-7.30 (4H，m，Ar-H)，7.94 (1H，d，J =8.0 Hz，Ar-H)，9.00 (1H，s，NH). IR (KBr) m/cm^{-1}: 3244, 3030, 1683, 1625, 1602, 1290, 1178, 762. HRMS m/z (ESI): 341.2231 [M+H]$^+$, calcd for $C_{21}H_{28}N_2O_2$: 340.2, Purity: 98.2% by HPLC (MeOH/0.1% TEA 85:15 (v/v)).

N-(2-(2-(piperidin-1-yl)ethoxy)phenyl)-3-phenylpropanamide (10f)

Light yellow solid, yield 68.5%, mp: 59.2～60.5℃; ^{1}H NMR (400 MHz, DMSO-d_6) δ (ppm): 1.36-1.37 (2H，m，piperidine-H)，1.46-1.49 (4H，m，piperidine-H)，2.42 (4H，m，piperidine-H)，2.64-2.71 (4H，m，OCH_2CH_2 and Ar-CH_2CH_2)，2.91 (2H，t，J =6.0 Hz，Ar-CH_2CH_2)，4.10 (2H，t，J =6.0 Hz，OCH_2CH_2)，6.88-6.92 (1H，m，Ar-H)，7.03-7.06 (2H，m，Ar-H)，7.16-7.20 (1H，m，Ar-H)，7.25-7.28 (4H，m，Ar-H)，7.91 (1H，d，J =8.0 Hz，Ar-H)，9.00 (1H，s，NH). IR (KBr) m/cm^{-1}: 3282, 3026, 1682, 1635, 1602, 1292, 1163, 765. HRMS m/z (ESI): 353.2229 [M+H]$^+$, calcd for $C_{22}H_{28}N_2O_2$: 352.2, Purity: 98.4% by HPLC (MeOH/0.1% TEA 85:15 (v/v)).

N-(2-(2-(pyrrolidin-1-yl)ethoxy)phenyl)-3-phenylpropanamide (10g)

Light yellow solid, yield 72.1%, mp: 53.6～54.8℃; ^1H NMR (400 MHz, DMSO-d_6) δ (ppm): 1.66 (4H，m，pyrrolidine-H)，2.50-2.52 (4H，m，pyrrolidine-H)，2.67 (2H，t，J =6.0 Hz，$OCH_2C\underline{H}_2$)，2.75 (2H，t，J =8.0 Hz，$Ar\text{-}CH_2C\underline{H}_2$)，2.91 (2H，t，$J$ =8.0 Hz，$Ar\text{-}C\underline{H}_2CH_2$)，4.11 (2H，t，$J$ =6.0 Hz，$OC\underline{H}_2CH_2$)，6.90-6.94 (1H，m，Ar-$\underline{H}$)，7.02-7.08 (2H，m，Ar-$\underline{H}$)，7.16-7.20 (1H，m，Ar-$\underline{H}$)，7.26-7.30 (4H，m，Ar-H)，7.93 (1H，d，J =8.0 Hz , Ar-$\underline{H}$)，9.16 (1H，s，N$\underline{H}$). IR (KBr) m/cm^{-1}: 3502, 3057, 1668, 1647, 1597, 1288, 1215, 746. HRMS m/z (ESI): 339.2084 $[M+H]^+$, calcd for $C_{21}H_{26}N_2O_2$: 338.2, Purity: 98.3% by HPLC (MeOH/0.1% TEA 85:15 (v/v)).

N-(3-(2-(dimethylamino)ethoxy)phenyl)-3-phenylpropanamide (11d)

Light yellow viscous liquid, yield 57.4%; ^1H NMR (400 MHz, DMSO-d_6) δ (ppm): 2.45 (6H，s，2× $NC\underline{H}_3$)，2.62 (2H，t，J =6.0 Hz，$OCH_2C\underline{H}_2$)，2.90 (2H，t，J =8.0 Hz，$Ar\text{-}CH_2C\underline{H}_2$)，2.95 (2H，t，$J$ =8.0 Hz，$Ar\text{-}C\underline{H}_2CH_2$)，4.11 (2H，t，$J$ =6.0 Hz，$OC\underline{H}_2CH_2$)，6.64 (1H，dd，J =8.0 Hz，4.0 Hz，Ar-$\underline{H}$)，7.07 (1H，d，J =8.0 Hz，Ar-$\underline{H}$)，7.17-7.21 (2H，m，Ar-$\underline{H}$)，7.24-7.30 (4H，m，Ar-$\underline{H}$)，7.38 (1H，s，Ar-$\underline{H}$)，9.94 (1H，s，N$\underline{H}$). IR (KBr) m/cm^{-1}: 3253, 3026, 1662, 1635, 1604, 1286, 1161, 700. HRMS m/z (ESI): 313.1933 $[M+H]^+$, calcd for $C_{19}H_{24}N_2O_2$: 312.2, Purity: 98.6% by HPLC (MeOH/0.1% TEA 85:15 (v/v)).

N-(3-(2-(diethylamino)ethoxy)phenyl)-3-phenylpropanamide (11e)

Light yellow viscous liquid, yield 61.5%; ^1H NMR (400 MHz, DMSO-d_6) δ (ppm): 1.14 (6H，t，J =6.0 Hz，2× $NCH_2C\underline{H}_3$)，2.63 (2H，t，J =6.0 Hz，$OCH_2C\underline{H}_2$)，2.89-2.93 (4H，m，2× $NC\underline{H}_2CH_3$)，2.65 (2H，t，J =8.0 Hz，$Ar\text{-}CH_2C\underline{H}_2$)，3.23 (2H，t，$J$ =8.0 Hz，$Ar\text{-}C\underline{H}_2CH_2$)，4.15 (2H，t，$J$ =6.0 Hz，$OC\underline{H}_2CH_2$)，6.66 (1H，dd，J =8.0 Hz，4.0 Hz，Ar-$\underline{H}$)，7.07 (1H，d，J =8.0 Hz，Ar-$\underline{H}$)，7.17-7.21 (2H，m，Ar-$\underline{H}$)，7.23-7.31 (4H，m，Ar-$\underline{H}$)，7.42 (1H，s，Ar-$\underline{H}$)，9.92 (1H，s，N$\underline{H}$). IR (KBr) m/cm^{-1}: 3271, 3026, 1668, 1635, 1598, 1288, 1161, 700. HRMS m/z (ESI): 341.2238 $[M+H]^+$, calcd for $C_{21}H_{28}N_2O_2$: 340.2, Purity: 98.2% by HPLC (MeOH/0.1% TEA 85:15 (v/v)).

N-(3-(2-(piperidin-1-yl)ethoxy)phenyl)-3-phenylpropanamide (11f)

Light yellow solid, yield 67.3%, mp: 85.3-87.1℃; ^1H NMR (400 MHz, DMSO-d_6)

δ (ppm): 1.40-1.43 (2H，m，piperidine-H)，1.58 (4H，m，piperidine-H)，2.37 (4H，m，piperidine-H)，2.62 (2H，t，J =6.0 Hz，$OCH_2C\underline{H}_2$), 2.73 (2H，t，J =8.0 Hz，Ar-$CH_2C\underline{H}_2$)，2.90 (2H，t，J =8.0 Hz，Ar-$C\underline{H}_2CH_2$)，4.10 (2H，t，J =6.0 Hz，$OC\underline{H}_2CH_2$)，6.64 (1H，dd，J =8.0 Hz，4.0 Hz，Ar-$\underline{H}$)，7.06 (1H，d，J =8.0 Hz，Ar-$\underline{H}$)，7.16-7.20 (2H，m，Ar-$\underline{H}$)，7.24-7.30 (4H，m，Ar-$\underline{H}$)，7.37 (1H，s，Ar-$\underline{H}$)，9.89 (1H，s，N$\underline{H}$). IR (KBr) m/cm^{-1}: 3253, 3030, 1662, 1622, 1600, 1282, 1163, 700. HRMS m/z (ESI): 353.2238 $[M+H]^+$, calcd for $C_{22}H_{28}N_2O_2$: 352.2, Purity: 98.3% by HPLC (MeOH/0.1% TEA 85:15 (v/v)).

N-(3-(2-(pyrrolidin-1-yl)ethoxy)phenyl)-3-phenylpropanamide (11g)

Light yellow solid, yield 73.8%, mp: 116.6-118.3℃; ^{1}H NMR (400 MHz, DMSO-d_6) δ (ppm): 1.66 (4H，m，pyrrolidine-H)，1.96 (4H，m，pyrrolidine-H)，2.64 (2H，t，J =6.0 Hz，$OCH_2C\underline{H}_2$)，2.91 (2H，t，J =8.0 Hz，Ar-$C\underline{H}_2CH_2$)，3.58 (2H，t，J =8.0 Hz，Ar-$CH_2C\underline{H}_2$)，4.25 (2H，t，J =6.0 Hz，$OC\underline{H}_2CH_2$)，6.70 (1H，dd，J =8.0 Hz，4.0 Hz，Ar-$\underline{H}$)，7.05 (1H，d，J =8.0 Hz，Ar-$\underline{H}$)，7.19-7.23 (2H，m，Ar-$\underline{H}$)，7.24-7.31 (4H，m，Ar-$\underline{H}$)，7.51 (1H，s，Ar-$\underline{H}$)，9.95 (1H，s，N$\underline{H}$). IR (KBr) m/cm^{-1}: 3248, 3028, 1653, 1616, 1597, 1290, 1165, 700. HRMS m/z (ESI): 339.2073$[M+H]^+$, calcd for $C_{21}H_{26}N_2O_2$: 338.2, Purity: 98.1% by HPLC (MeOH/0.1% TEA 85:15 (v/v)).

N-(4-(2-(dimethylamino)ethoxy)phenyl)-3-phenylpropanamide (12d)

Gray solid, yield 54.1%, mp: 90.2-91.8℃; ^{1}H NMR (400 MHz, DMSO-d_6) δ (ppm): 2.28 (6H，s，2× $NC\underline{H}_3$)，2.59 (2H，t，J =6.0 Hz，$OCH_2C\underline{H}_2$)，2.70 (2H，t，J =8.0 Hz，Ar-$CH_2C\underline{H}_2$)，2.90 (2H，t，J =8.0 Hz，Ar-$C\underline{H}_2CH_2$)，4.03 (2H，t，J =6.0 Hz，$OC\underline{H}_2CH_2$)，6.88 (2H，d，J =6.0 Hz，Ar-$\underline{H}$)，7.19 (1H，t，J =6.0 Hz，Ar-$\underline{H}$)，7.24-7.31 (4H，m，Ar-$\underline{H}$)，7.47 (2H，d，J =8.0 Hz，Ar-$\underline{H}$)，9.76 (1H，s，N$\underline{H}$). IR (KBr) m/cm^{-1}: 3367, 2953, 1667, 1629, 1598, 1267, 1182, 775. HRMS m/z (ESI): 313.1943 $[M+H]^+$, calcd for $C_{19}H_{24}N_2O_2$: 312.2, Purity: 98.7% by HPLC (MeOH/0.1% TEA 85:15 (v/v)).

N-(4-(2-(diethylamino)ethoxy)phenyl)-3-phenylpropanamide (12e)

White solid, yield 56.3%, mp: 113.3-115.1℃; ^{1}H NMR (400 MHz, DMSO-d_6) δ

(ppm): 1.16 (6H，t，*J* =8.0 Hz，2× $NCH_2C\underline{H}_3$)，2.59 (2H，t，*J* =6.0 Hz，$OCH_2C\underline{H}_2$)，2.90 (2H，t，*J* =8.0 Hz，Ar-$CH_2C\underline{H}_2$)，3.13 (2H，t，*J* =8.0 Hz，Ar-$C\underline{H}_2CH_2$)，3.33 (4H，m，2× $NC\underline{H}_2CH_3$)，4.19 (2H，t，*J* =6.0 Hz，$OC\underline{H}_2CH_2$)，6.93 (2H，d，*J* =6.0 Hz，Ar-$\underline{H}$)，7.19 (1H，t，*J* =8.0 Hz，Ar-$\underline{H}$)，7.24-7.31 (4H，m，Ar-$\underline{H}$)，7.51 (2H，d，*J* =8.0 Hz，Ar-$\underline{H}$)，9.80 (1H，s，N$\underline{H}$). IR (KBr) m/cm^{-1}: 3309, 2960, 1667, 1624, 1598, 1265, 1174, 776. HRMS m/z (ESI): 341.2232 $[M+H]^+$, calcd for $C_{21}H_{28}N_2O_2$: 340.2, Purity: 98.2% by HPLC (MeOH/0.1% TEA 85:15 (v/v)).

N-(4-(2-(piperidin-1-yl)ethoxy)phenyl)-3-phenylpropanamide (12f)

Light yellow solid, yield 61.9%, mp: 116.8-117.8℃; ^{1}H NMR (400 MHz, DMSO-d_6) δ (ppm): 1.43 (6H，m，piperidine-H)，1.58 (4H，m，piperidine-H)，2.59 (2H，t，*J* =6.0 Hz，$OCH_2C\underline{H}_2$)，2.90 (2H，t，*J* =8.0 Hz，Ar-$CH_2C\underline{H}_2$)，3.33 (2H，t，*J* =8.0 Hz，Ar-$C\underline{H}_2CH_2$)，4.10 (2H，t，*J* =6.0 Hz，$OC\underline{H}_2CH_2$)，6.90 (2H，d，*J* =6.0 Hz，Ar-$\underline{H}$)，7.19 (1H，t，*J* =6.0 Hz，Ar-$\underline{H}$)，7.24-7.31 (4H，m，Ar-$\underline{H}$)，7.48 (2H，d，*J* =8.0 Hz，Ar-$\underline{H}$)，9.77 (1H，s，N$\underline{H}$). IR (KBr) m/cm^{-1}: 3277, 3062, 1672, 1622, 1600, 1288, 1172, 765. HRMS m/z (ESI): 353.2230 $[M+H]^+$, calcd for $C_{22}H_{28}N_2O_2$: 352.2, Purity: 98.4% by HPLC (MeOH/0.1% TEA 85:15 (v/v)).

3-phenyl-N-(4-(2-(pyrrolidin-1-yl)ethoxy)phenyl)propanamide (12g)

White solid, yield 58.1%, mp: 163.6-165.5℃; ^{1}H NMR (400 MHz, DMSO-d_6) δ (ppm): 1.95 (4H，m，pyrrolidine-H)，2.60 (2H，t，*J* =6.0 Hz，$OCH_2C\underline{H}_2$)，2.91 (2H，t，*J* =8.0 Hz，Ar-$C\underline{H}_2CH_2$)，3.15 (2H，t，*J* =8.0 Hz，Ar-$CH_2C\underline{H}_2$)，3.67-3.58 (4H，m，pyrrolidine-H)，4.25 (2H，t，*J* =6.0 Hz，$OC\underline{H}_2CH_2$)，6.96 (2H，d，*J* =8.0 Hz，Ar-$\underline{H}$)，7.19 (1H，t，*J* =8.0 Hz，Ar-$\underline{H}$)，7.24-7.31 (4H，m，Ar-$\underline{H}$)，7.52 (2H，d，*J* =8.0 Hz，Ar-$\underline{H}$)，9.81 (1H，s，N$\underline{H}$). IR (KBr) m/cm^{-1}: 3269, 3061, 1668, 1622, 1598, 1294, 1176, 771. HRMS m/z (ESI): 339.2067 $[M+H]^+$, calcd for $C_{21}H_{26}N_2O_2$: 338.2, Purity: 98.6% by HPLC (MeOH/0.1% TEA 85:15 (v/v)).

(2E,4E)-N-(2-(2-(dimethylamino)ethoxy)phenyl)hexa-2,4-dienamide (16d)

Light yellow viscous liquid, .032 g, yield 78.6%; ^{1}H NMR (400 MHz, DMSO-d_6) δ (ppm): 1.83 (3H，d，*J* =8.0 Hz，$C\underline{H}_3CHCHCHCHCO$)，2.28 (6H，s，2× $NC\underline{H}_3$)，

2.73 (2H，t，*J* =6.0 Hz，$OCH_2C\underline{H}_2$)，4.03 (2H，t，*J* =6.0 Hz，$OC\underline{H}_2CH_2$)，6.10 (1H，d，*J* =16.0 Hz，$CH_3CHCHCHC\underline{H}CO$)，6.16-6.22 (1H，m，$CH_3C\underline{H}CHCHCHCO$)，6.27-6.33 (1H，m，$CH_3CHC\underline{H}CHCHCO$)，6.63 (1H，d，*J* =8.0 Hz，Ar-$\underline{H}$)，7.12-7.21 (3H，m，$CH_3CHCHC\underline{H}CHCO$ and 2× Ar-$\underline{H}$)，7.41 (1H，d，*J* =8.0 Hz，Ar-$\underline{H}$)，10.03 (1H，s，N$\underline{H}$). IR (KBr) m/cm^{-1}: 3250, 3064, 1670, 1639, 1597, 1284, 1213, 927, 746. HRMS m/z (ESI): 275.1770 $[M+H]^+$, calcd for $C_{16}H_{22}N_2O_2$: 274.2, Purity: 98.3% by HPLC (MeOH/0.1% TEA 85:15 (v/v)).

(2E,4E)-N-(2-(2-(diethylamino)ethoxy)phenyl)hexa-2,4-dienamide (16e)

Light yellow viscous liquid, yield 72.1%; ^1H NMR (400 MHz, DMSO-d_6) δ (ppm): 1.02 (6H，m，2× $NCH_2C\underline{H}_3$)，1.83 (3H，d，*J* =6.0 Hz，$C\underline{H}_3CHCHCHCHCO$)，2.66 (4H，m，2× $NC\underline{H}_2CH_3$)，2.89 (2H，t，*J* =6.0 Hz，$OCH_2C\underline{H}_2$)，4.02 (2H，t，*J* =6.0 Hz，$OC\underline{H}_2CH_2$)，6.10 (1H，d，*J* =16.0 Hz，$CH_3CHCHCHC\underline{H}CO$)，6.16-6.22 (1H，m，$CH_3C\underline{H}CHCHCHCO$)，6.27-6.33 (1H，m，$CH_3CHC\underline{H}CHCHCO$)，6.63 (1H，d，*J* =8.0 Hz，Ar-$\underline{H}$)，7.12-7.22 (3H，m，$CH_3CHCHC\underline{H}CHCO$ and 2× Ar-$\underline{H}$)，7.42 (1H，d，*J* =8.0 Hz，Ar-$\underline{H}$)，10.01 (1H，s，N$\underline{H}$). IR (KBr) m/cm^{-1}: 3566, 3062, 1670, 1635, 1593, 1290, 1236, 925, 776. HRMS m/z (ESI): 303.2076 $[M+H]^+$, calcd for $C_{18}H_{26}N_2O_2$: 302.2, Purity: 97.8% by HPLC (MeOH/0.1% TEA 85:15 (v/v)).

(2E,4E)-N-(2-(2-(piperidin-1-yl)ethoxy)phenyl)hexa-2,4-dienamide (16f)

Light purple viscous liquid, yield 68.8%; ^1H NMR (400 MHz, DMSO-d_6) δ (ppm): 1.40 (2H，m，piperidine-$\underline{H}$)，1.52 (4H，m，piperidine-$\underline{H}$)，1.83 (3H，d，*J* =8.0 Hz，$C\underline{H}_3CHCHCHCHCO$)，2.33 (4H，m，piperidine-$\underline{H}$)，2.67 (2H，t，*J* =6.0 Hz，$OCH_2C\underline{H}_2$)，4.04 (2H，t，*J* =6.0 Hz，$OC\underline{H}_2CH_2$)，6.09 (1H，d，*J* =16.0 Hz，$CH_3CHCHCHC\underline{H}CO$)，6.16-6.22 (1H，m，$CH_3C\underline{H}CHCHCHCO$)，6.27-6.33 (1H，m，$CH_3CHC\underline{H}CHCHCO$)，6.64 (1H，d，*J* =8.0 Hz，Ar-$\underline{H}$)，7.12-7.20 (3H，m，$CH_3CHCHC\underline{H}CHCO$ and 2× Ar-$\underline{H}$)，7.41 (1H，d，*J* =8.0 Hz，Ar-$\underline{H}$)，10.01 (1H，s，N$\underline{H}$). IR (KBr) m/cm^{-1}: 3232, 3024, 1662, 1635, 1599, 1286, 1244, 924, 750. HRMS m/z (ESI): 315.2075 $[M+H]^+$, calcd for $C_{19}H_{26}N_2O_2$: 314.2, Purity: 98.4% by HPLC (MeOH/0.1% TEA 85:15 (v/v)).

(2E,4E)-N-(2-(2-(pyrrolidin-1-yl)ethoxy)phenyl)hexa-2,4-dienamide (16g)

Light yellow viscous liquid, yield 51.2% ; ^{1}H NMR (400 MHz, DMSO-d_6) δ (ppm): 1.74 (4H， m， pyrrolidine-$\underline{H}$)， 1.83 (3H， d， J =8.0 Hz， $C\underline{H}_3CHCHCHCHCO$)，2.69 (4H，m，pyrrolidine-$\underline{H}$)，2.95 (2H，t，J =6.0 Hz，$OCH_2C\underline{H}_2$)，4.07 (2H，t，J =6.0 Hz，$OC\underline{H}_2CH_2$)，6.10 (1H，d，J =16.0 Hz，$CH_3CHCHCHC\underline{H}CO$)，6.16-6.22 (1H，m，$CH_3C\underline{H}CHCHCHCO$)，6.27-6.33 (1H，m，$CH_3CHC\underline{H}CHCHCO$)，6.64 (1H，d，$J$ =8.0 Hz，Ar-$\underline{H}$)，7.12-7.22 (3H，m，$CH_3CHCHC\underline{H}CHCO$ and 2× Ar-$\underline{H}$)，7.43 (1H，d，J =8.0 Hz，Ar-$\underline{H}$)，10.03 (1H，s，N$\underline{H}$). IR (KBr) m/cm^{-1}: 3242, 3055, 1674, 1637, 1595, 1286, 1253, 916, 750. HRMS m/z (ESI): 301.1915 [M+H]$^+$, calcd for $C_{18}H_{24}N_2O_2$: 300.2, Purity: 97.7% by HPLC (MeOH/0.1% TEA 85:15 (v/v)).

(2E,4E)-N-(3-(2-(dimethylamino)ethoxy)phenyl)hexa-2,4-dienamide (17d)

Light yellow viscous liquid, yield 74.5%; ^{1}H NMR (400 MHz, DMSO-d_6) δ (ppm): 1.83 (3H，d，J =4.0 Hz，$C\underline{H}_3CHCHCHCHCO$)，2.25 (6H，s，2× $NC\underline{H}_3$)，2.66 (2H，t，J =6.0 Hz，$OCH_2C\underline{H}_2$)，4.64 (2H，t，J =6.0 Hz，$OC\underline{H}_2CH_2$)，7.05 (1H，t， J =16.0 Hz， $CH_3CHCHCHC\underline{H}CO$)， 7.11 (1H， d， J =8.0 Hz， $CH_3C\underline{H}CHCHCHCO$)，7.15-7.19 (1H，m，$CH_3CHC\underline{H}CHCHCO$)，7.47 (1H，t，$J$ =6.0 Hz，Ar-$\underline{H}$)，7.62-7.66 (1H，m，Ar-$\underline{H}$)，7.83 (1H，d，J =8.0 Hz，Ar-$\underline{H}$)，8.07 (1H，d，J =8.0 Hz，Ar-$\underline{H}$)，8.11-8.13 (1H，m，$CH_3CHCHC\underline{H}CHCO$)，9.52 (1H，s，N$\underline{H}$). IR (KBr) m/cm^{-1}: 3251, 3028, 1668, 1635, 1608, 1286, 1247, 929, 750. HRMS m/z (ESI): 275.1759 [M+H]$^+$, calcd for $C_{16}H_{22}N_2O_2$: 274.2, Purity: 98.5% by HPLC (MeOH/0.1% TEA 85:15 (v/v)).

(2E,4E)-N-(3-(2-(diethylamino)ethoxy)phenyl)hexa-2,4-dienamide (17e)

Light yellow viscous liquid, yield 70.4%; ^{1}H NMR (400 MHz, DMSO-d_6) δ (ppm): 0.98 (6H，t，J =8.0 Hz ， $2{\times}NCH_2C\underline{H}_3$)， 1.83 (3H，d，$J$ =8.0 Hz， $C\underline{H}_3CHCHCHCHCO$)，2.54-2.59 (4H，m，$2{\times}NC\underline{H}_2CH_3$)，2.78 (2H，t，$J$ =6.0 Hz，$OCH_2C\underline{H}_2$)，4.07 (2H，t，J =6.0 Hz，$OC\underline{H}_2CH_2$)，6.16-6.31 (3H，m，$CH_3C\underline{H}CHC\underline{H}C\underline{H}CO$)，6.92 (1H，t，$J$=8 Hz，Ar-$\underline{H}$)，7.02-7.18 (3H，m，$CH_3CHC\underline{H}CHCHCO$ and 2× Ar-$\underline{H}$)，8.06 (1H，d，J =4.0 Hz, Ar-$\underline{H}$)，9.17 (1H，

s，NH). IR (KBr) m/cm^{-1}: 3275, 3055, 1685, 1635, 1589, 1288, 1240, 927, 756. HRMS m/z (ESI): 303.2070[M+H]$^{+}$, calcd for $C_{18}H_{26}N_2O_2$: 302.2, Purity: 98.3% by HPLC (MeOH/0.1% TEA 85:15 (v/v)).

(2E,4E)-N-(3-(2-(piperidin-1-yl)ethoxy)phenyl)hexa-2,4-dienamide (17f)

Light yellow viscous liquid, yield 74.4%; ^{1}H NMR (400 MHz, DMSO-d_6) δ (ppm): 1.16-1.20 (2H，m，piperidine-H)，1.71 (4H，m，piperidine-H)，1.84 (3H，d，*J* =8.0 Hz，CH_3CHCHCHCHCO)，3.10 (2H，t, *J* =6.0 Hz，OCH_2CH_2)，3.53 (4H，m，piperidine-H)，4.39 (2H，t，*J* =6.0 Hz，OCH_2CH_2)，6.18-6.34 (3H，m，CH_3CHCHCHCHCO)，6.96-7.20 (4H，m，CH_3CHCHCHCHCO and 3× Ar-H)，7.93 (1H，d，*J* =8 Hz，Ar-H)，9.27 (1H，s，NH). IR (KBr) m/cm^{-1}: 3255, 3026, 1668, 1635, 1608, 1286, 1246, 929, 749. HRMS m/z (ESI): 315.2069 [M+H]$^{+}$, calcd for $C_{19}H_{26}N_2O_2$: 314.2, Purity: 98.6% by HPLC (MeOH/0.1% TEA 85:15 (v/v)).

(2E,4E)-N-(3-(2-(pyrrolidin-1-yl)ethoxy)phenyl)hexa-2,4-dienamide (17g)

Light yellow viscous liquid, yield 65.8%; ^{1}H NMR (400 MHz, DMSO-d_6) δ (ppm): 1.71 (4H，m，pyrrolidine-H)，1.83 (3H，d，*J* =8.0 Hz，CH_3CHCHCHCHCO)，2.54 (4H，m，pyrrolidine-H)，2.77 (2H，t，*J* =6.0 Hz，OCH_2CH_2)，4.15 (2H，t，*J* =6.0 Hz，OCH_2CH_2)，6.16-6.30 (3H，m，CH_3CHCHCHCHCO)，6.94 (1H，t，*J* =8 Hz，Ar-H)，7.03-7.08 (3H，m，CH_3CHCHCHCHCO and 2× Ar-H)，8.07 (1H，d，*J* =8 Hz，Ar-H)，9.30 (1H，s，NH). IR (KBr) m/cm^{-1}: 3250, 3024, 1668, 1635, 1608, 1286, 1246, 948, 727. HRMS m/z (ESI): 301.1921 [M+H]$^{+}$, calcd for $C_{18}H_{24}N_2O_2$: 300.2, Purity: 98.2% by HPLC (MeOH/0.1% TEA 85:15 (v/v)).

(2E,4E)-N-(4-(2-(dimethylamino)ethoxy)phenyl)hexa-2,4-dienamide (18d)

White solid, yield 71.3%, mp: 122.2-123.1℃; ^{1}H NMR (400 MHz, DMSO-d_6) δ (ppm): 1.83 (3H，d，*J* =4.0 Hz，CH_3CHCHCHCHCO)，2.20 (6H，s，2×NCH_3)，2.59 (2H，t，*J* =6.0 Hz，OCH_2CH_2)，4.00 (2H，t，*J* =6.0 Hz，OCH_2CH_2)，6.07 (1H，d，*J* =16.0 Hz，CH_3CHCHCHCHCO)，6.14-6.19 (1H，m，CH_3CHCHCHCHCO)，6.25-6.32 (1H，m，CH_3CHCHCHCHCO)，6.88 (2H，

d，J =8.0 Hz，Ar-H)，7.09-7.16 (1H，m，CH_3CHCHCHCHCO)，7.55 (2H，d，J =8.0 Hz，Ar-H)，9.88 (1H，s，NH). IR (KBr) m/cm^{-1}: 3300, 3024, 1658, 1633, 1598, 1292, 1244, 923, 723. HRMS m/z (ESI): 275.1760 $[M+H]^+$, calcd for $C_{16}H_{22}N_2O_2$: 274.2, Purity: 99.2% by HPLC (MeOH/0.1% TEA 85:15 (v/v)).

(2E,4E)-N-(4-(2-(diethylamino)ethoxy)phenyl)hexa-2,4-dienamide (18e)

White solid, yield 73.9%, mp: 103.2-104.7℃; ^{1}H NMR (400 MHz, DMSO-d_6) δ (ppm): 0.98 (6H，t，J =8.0 Hz，2×NCH_2CH_3)，1.83 (3H，d，J =4.0 Hz，CH_3CHCHCHCHCO)，2.56-2.57 (4H，m，2×NCH_2CH_3)，2.77 (2H，t，J =6.0 Hz，OCH_2CH_2)，3.98 (2H，t，J =6.0 Hz，OCH_2CH_2)，6.07 (1H，d，J =16.0 Hz，CH_3CHCHCHCHCO)，6.14-6.21 (1H，m，CH_3CHCHCHCHCO)，6.25-6.32 (1H，m，CH_3CHCHCHCHCO)，6.88 (2H，d，J =8.0 Hz，Ar-H)，7.09-7.16 (1H，m，CH_3CHCHCHCHCO)，7.55 (2H，d，J =8.0 Hz，Ar-H)，9.89 (1H，s，NH). IR (KBr) m/cm^{-1}: 3319, 3028, 1660, 1633, 1598, 1301, 1246, 927, 721. HRMS m/z (ESI): 303.2087 $[M+H]^+$, calcd for $C_{18}H_{26}N_2O_2$: 302.2, Purity: 99.2% by HPLC (MeOH/0.1% TEA 85:15 (v/v)).

(2E,4E)-N-(4-(2-(piperidin-1-yl)ethoxy)phenyl)hexa-2,4-dienamide (18f)

White solid, yield 69.4%, mp: 134.7-136.2℃; ^{1}H NMR (400 MHz, DMSO-d_6) δ (ppm): 1.40 (2H，m，piperidine-H)，1.52 (4H，m，piperidine-H)，1.83 (3H，d，J =4.0 Hz，CH_3CHCHCHCHCO)，2.67 (4H，m，piperidine-H)，3.32 (2H，t，J =6.0 Hz，OCH_2CH_2)，4.05 (2H，t，J =6.0 Hz，OCH_2CH_2)，6.07 (1H，d，J =16.0 Hz，CH_3CHCHCHCHCO)，6.14-6.21 (1H，m，CH_3CHCHCHCHCO)，6.25-6.32 (1H，m，CH_3CHCHCHCH-CO)，6.89 (2H，d，J =8.0 Hz，Ar-H)，7.72 (1H，dd，J =12.0 Hz，16 Hz，CH_3CHCHCHCHCO)，7.55 (2H，d，J =8.0 Hz，Ar-H)，9.88 (1H，s，NH). IR (KBr) m/cm^{-1}: 3300, 3022, 1658, 1633, 1600, 1301, 1246, 929, 727. HRMS m/z (ESI): 315.2074 $[M+H]^+$, calcd for $C_{19}H_{26}N_2O_2$: 314.2, Purity: 98.3% by HPLC (MeOH/0.1% TEA 85:15 (v/v)).

(2E,4E)-N-(4-(2-(pyrrolidin-1-yl)ethoxy)phenyl)hexa-2,4-dienamide (18g)

White solid, yield 65.8%, mp: 122.6-123.7℃; ^{1}H NMR (400 MHz, DMSO-d_6) δ (ppm): 0.96-0.99 (4H，m，pyrrolidine-H)，1.83 (3H，d，J =4.0 Hz，

$C\underline{H}_3CHCHCHCHCO$)，2.57 (4H，m，pyrrolidine-$\underline{H}$)，2.82 (2H，t，J =6.0 Hz，$OCH_2C\underline{H}_2$)，4.04 (2H，t，J =6.0 Hz，$OC\underline{H}_2CH_2$)，6.08 (1H，d，J =16.0 Hz，$CH_3CHCHCHC\underline{H}CO$)，6.15-6.19 (1H，m，$CH_3C\underline{H}CHCHCHCO$)，6.25-6.32 (1H，m，$CH_3CHC\underline{H}CHCHCO$)，6.89 (2H，d，$J$ =8.0 Hz，Ar-$\underline{H}$)，7.09-7.16 (1H，m，$CH_3CHCHC\underline{H}CHCO$)，7.56 (2H，d，$J$ =8.0 Hz，Ar-$\underline{H}$)，9.89 (1H，s，N$\underline{H}$). IR (KBr) m/cm^{-1}: 3304, 3026, 1660, 1633, 1598, 1290, 1242, 958, 721. HRMS m/z (ESI): 301.1916 $[M+H]^+$, calcd for $C_{18}H_{24}N_2O_2$: 300.2, Purity: 98.5% by HPLC (MeOH/0.1% TEA 85:15 (v/v)).)

N-(2-(2-(dimethylamino)ethoxy)phenyl)hexanamide(22d)

White powder, yield: 68.3%, mp: 158.3-159.7 ℃; ^{1}H NMR (400 MHz, DMSO-d_6) δ (ppm): 0.89 (3H，t，J = 6.0 Hz，$C\underline{H}_3CH_2CH_2CH_2CH_2CO$)，1.30-1.31 (2H，m，$CH_3CH_2C\underline{H}_2CH_2CH_2CO$)，1.58-1.61 (2H，m，$CH_3C\underline{H}_2CH_2CH_2CH_2CO$)，2.23 (6H，s，2× $NC\underline{H}_3$)，2.29 (2H，t，J =6.0 Hz，$CH_3CH_2CH_2CH_2C\underline{H}_2CO$)，2.31-2.34 (2H，m，$CH_3CH_2CH_2C\underline{H}_2CH_2CO$)，2.66 (2H，t，$J$ =6.0 Hz，$OCH_2C\underline{H}_2$)，4.04 (2H，t，J =6.0 Hz，$OC\underline{H}_2CH_2$)，7.49 (1H，t，J =8.0 Hz，Ar-H)，7.65 (1H，t，J =8.0 Hz，Ar-H)，7.84 (1H，d，J =8.0 Hz，Ar-H)，8.08 (1H，d，J =4.0 Hz，Ar-H)，9.33 (1H，s，N$\underline{H}$). IR (KBr) m/cm^{-1}: 3232, 3002, 1298, 1174. HRMS m/z (ESI): 279.2071$[M+H]^+$, calcd for $C_{16}H_{26}N_2O_2$: 278.2, Purity: 98% by HPLC (MeOH/0.1% TEA 85:15 (v/v)).

N-(2-(2-(diethylamino)ethoxy)phenyl)hexanamide(22e)

Light yellow viscous liquid, yield 52.3%; ^{1}H NMR (400 MHz, DMSO-d_6) δ (ppm): 0.88 (3H，t，J = 6.0 Hz，$C\underline{H}_3CH_2CH_2CH_2CH_2CO$)，0.94 (6H，t，$J$ = 8.0 Hz，2× $NCH_2C\underline{H}_3$)，0.97-1.00 (4H，m，2× $NC\underline{H}_2CH_3$)，1.30 (2H，m，$CH_3C\underline{H}_2CH_2CH_2CH_2CO$)，2.55-2.59 (6H，m，$CH_3CH_2C\underline{H}_2C\underline{H}_2C\underline{H}_2CO$)，2.83 (2H，t，$J$ =6.0 Hz，$OCH_2C\underline{H}_2$)，4.61 (2H，t，J =6.0 Hz，$OC\underline{H}_2CH_2$)，7.48 (1H，t，J =8.0 Hz，Ar-H)，7.65 (1H，t，J =8.0 Hz，Ar-H)，7.84 (1H，d，J =8.0 Hz，Ar-H)，8.07 (1H，d，J =8.0 Hz，Ar-H)，8.97 (1H，s，N$\underline{H}$). IR (KBr) m/cm^{-1}: 3319, 3057, 1236, 1176. HRMS m/z (ESI): 307.2403 $[M+H]^+$, calcd for $C_{18}H_{30}N_2O_2$: 306.2, Purity: 98% by HPLC (MeOH/0.1% TEA 85:15 (v/v)).

N-(2-(2-(piperidin-1-yl)ethoxy)phenyl)hexanamide (22f)

Light yellow viscous liquid, yield 63.7%; ^{1}H NMR (400 MHz, DMSO-d_6) δ (ppm): 0.88 (3H，t，J = 8.0 Hz，$C\underline{H}_3CH_2CH_2CH_2CH_2CO$)，1.27-1.32 (4H，m，$CH_3C\underline{H}_2C\underline{H}_2CH_2CH_2CO$)，1.39 (2H，m，$CH_3CH_2CH_2C\underline{H}_2CH_2CO$)，1.52-1.60 (6H，m，piperidine-H)，2.29 (2H，t，J =6.0 Hz，$CH_3CH_2CH_2CH_2C\underline{H}_2CO$)，2.47 (4H，m，piperidine-H)，2.72 (2H，t，J =6.0 Hz，$OCH_2C\underline{H}_2$)，4.03 (2H，t，J =6.0 Hz，$OC\underline{H}_2CH_2$)，7.48 (1H，t，J =8.0 Hz，Ar-H)，7.66 (1H，t，J =8.0 Hz，Ar-H)，7.84 (1H，d，J =8.0 Hz，Ar-H)，8.07 (1H，d，J =8.0 Hz，Ar-H)，8.97 (1H，s，N$\underline{H}$). IR (KBr) m/cm^{-1}: 3330, 3029, 1275, 1165. HRMS m/z (ESI): 319.2389[M+H]$^+$, calcd for $C_{19}H_{30}N_2O_2$: 318.2, Purity: 98% by HPLC (MeOH/0.1% TEA 85:15 (v/v)).

N-(2-(2-(pyrrolidin-1-yl)ethoxy)phenyl)hexanamide(22g)

Light yellow viscous liquid, yield 68.1%; ^{1}H NMR (400 MHz, DMSO-d_6) δ (ppm): 0.88 (3H，t，J = 8.0 Hz，$C\underline{H}_3CH_2CH_2CH_2CH_2CO$)，1.27-1.31 (4H，m，$CH_3C\underline{H}_2C\underline{H}_2CH_2CH_2CO$)，1.57-1.60 (2H，m，$CH_3CH_2CH_2C\underline{H}_2CH_2CO$)，1.71 (4H，m，pyrrolidine-H)，2.29 (2H，t，J =6.0 Hz，$CH_3CH_2CH_2CH_2C\underline{H}_2CO$)，2.58 (4H，m，pyrrolidine-H)，2.84 (2H，t，J =6.0 Hz，$OCH_2C\underline{H}_2$)，4.04 (2H，t，J =6.0 Hz，$OC\underline{H}_2CH_2$)，7.49 (1H，t，J =8.0 Hz，Ar-H)，7.65 (1H，t，J =8.0 Hz，Ar-H)，7.84 (1H，d，J =8.0 Hz，Ar-H)，8.07 (1H，d，J =8.0 Hz，Ar-H)，8.97 (1H，s，N$\underline{H}$). IR (KBr) m/cm^{-1}: 3238, 2931, 1234, 1172. HRMS m/z (ESI): 305.2230 [M+H]$^+$, calcd for $C_{18}H_{28}N_2O_2$: 304.2, Purity: 98% by HPLC (MeOH/0.1% TEA 85:15 (v/v)).

N-(3-(2-(dimethylamino)ethoxy)phenyl)hexanamide(23d)

Light yellow viscous liquid, yield: 55.9%; ^{1}H NMR (400 MHz, DMSO-d_6) δ (ppm): 0.88 (3H，t，J = 8.0 Hz，$C\underline{H}_3CH_2CH_2CH_2CH_2CO$)，1.28-1.32 (4H，m，$CH_3C\underline{H}_2C\underline{H}_2CH_2CH_2CO$)，1.57-1.60 (2H，m，$CH_3CH_2CH_2C\underline{H}_2CH_2CO$)，2.29 (2H，t，$J$ =6.0 Hz，$CH_3CH_2CH_2CH_2C\underline{H}_2CO$)，2.85 (6H，s，2× $NC\underline{H}_3$)，3.13 (2H，t，J =6.0 Hz，$OCH_2C\underline{H}_2$)，4.16 (2H，t，J =6.0 Hz，$OC\underline{H}_2CH_2$)，6.65 (1H，d，J =4.0 Hz，Ar-$\underline{H}$)，7.09 (1H，t，J =4.0 Hz，Ar-$\underline{H}$)，7.21 (1H，t，J =8.0 Hz，Ar-$\underline{H}$)，7.44 (1H，s，Ar-$\underline{H}$)，9.91 (1H，s，N$\underline{H}$). IR (KBr) m/cm^{-1}: 3333, 3020, 1262, 1173. HRMS m/z (ESI): 279.2077 [M+H]$^+$, calcd for $C_{16}H_{26}N_2O_2$: 278.2, Purity:

98% by HPLC (MeOH/0.1% TEA 85:15 (v/v)).

N-(3-(2-(diethylamino)ethoxy)phenyl)hexanamide(23e)

Light yellow viscous liquid, yield 64.7%; ^{1}H NMR (400 MHz, DMSO-d_6) δ (ppm): 0.88 (3H，t，J = 6.0 Hz，$C\underline{H}_3CH_2CH_2CH_2CH_2CO$)，0.94 (6H，t，$J$ =8.0 Hz，2× $NCH_2C\underline{H}_3$)，0.97-1.00 (4H，m，2× $NC\underline{H}_2CH_3$)，1.30 (2H，m，$CH_3C\underline{H}_2CH_2CH_2CH_2CO$)，2.55-2.59 (6H，m，$CH_3CH_2C\underline{H}_2C\underline{H}_2C\underline{H}_2CO$)，2.84 (2H，t，$J$ =6.0 Hz，$OCH_2C\underline{H}_2$)，4.61 (2H，t，J =6.0 Hz，$OC\underline{H}_2CH_2$)，6.61 (1H，d，J =8.0 Hz，Ar-$\underline{H}$)，7.09 (1H，t，J =8.0 Hz，Ar-$\underline{H}$)，7.18 (1H，t，J =8.0 Hz，Ar-$\underline{H}$)，7.34 (1H，s，Ar-$\underline{H}$)，9.91 (1H，s，N$\underline{H}$). IR (KBr) m/cm^{-1}: 3313, 3049, 1234, 1172. HRMS m/z (ESI): 307.2400 [M+H]$^+$, calcd for $C_{18}H_{30}N_2O_2$: 306.2, Purity: 98% by HPLC (MeOH/0.1% TEA 85:15 (v/v)).

N-(3-(2-(piperidin-1-yl)ethoxy)phenyl)hexanamide(23f)

Light yellow viscous liquid, yield 59.2%; ^{1}H NMR (400 MHz, DMSO-d_6) δ (ppm): 0.88 (3H，t，J = 6.0 Hz，$C\underline{H}_3CH_2CH_2CH_2CH_2CO$)，1.27-1.32 (4H，m，$CH_3C\underline{H}_2C\underline{H}_2CH_2CH_2CO$)，1.39 (2H，m，$CH_3CH_2CH_2C\underline{H}_2CH_2CO$)，1.52-1.60 (6H，m，piperidine-H)，2.29 (2H，t，J =6.0 Hz，$CH_3CH_2CH_2CH_2C\underline{H}_2CO$)，2.47 (4H，m，piperidine-H)，2.72 (2H，t，J =6.0 Hz，$OCH_2C\underline{H}_2$)，4.03 (2H，t，J =6.0 Hz，$OC\underline{H}_2CH_2$)，6.61 (1H，d，J =8.0 Hz，Ar-$\underline{H}$)，7.08 (1H，t，J =4.0 Hz，Ar-$\underline{H}$)，7.18 (1H，t，J =6.0 Hz，Ar-$\underline{H}$)，7.34 (1H，s，Ar-$\underline{H}$)，9.84 (1H，s，N$\underline{H}$). IR (KBr) m/cm^{-1}: 3259, 3030, 1249, 1192. HRMS m/z (ESI): 319.2401 [M+H]$^+$, calcd for $C_{19}H_{30}N_2O_2$: 318.2, Purity: 98% by HPLC (MeOH/0.1% TEA 85:15 (v/v)).

N-(3-(2-(pyrrolidin-1-yl)ethoxy)phenyl)hexanamide(23g)

Light yellow viscous liquid, yield 72.6%; ^{1}H NMR (400 MHz, DMSO-d_6) δ (ppm): 0.88 (3H，t，J = 6.0 Hz，$C\underline{H}_3CH_2CH_2CH_2CH_2CO$)，1.27-1.31 (4H，m，$CH_3C\underline{H}_2C\underline{H}_2CH_2CH_2CO$)，1.57-1.60 (2H，m，$CH_3CH_2CH_2C\underline{H}_2CH_2CO$)，1.71 (4H，m，pyrrolidine-H)，2.29 (2H，t，J =6.0 Hz，$CH_3CH_2CH_2CH_2C\underline{H}_2CO$)，2.58 (4H，m，pyrrolidine-H)，2.84 (2H，t，J =6.0 Hz，$OCH_2C\underline{H}_2$)，4.04 (2H，t，J =6.0 Hz，$OC\underline{H}_2CH_2$)，6.65 (1H, d, J =4.0 Hz, Ar-H), 7.09 (1H, d, J =4.0 Hz,

Ar-H), 7.21 (1H，t，*J* =6.0 Hz，Ar-$\underline{H}$), 7.44 (1H，s，Ar-$\underline{H}$), 9.85 (1H，s，N$\underline{H}$). IR (KBr) m/cm^{-1}: 3253, 3030, 1288, 1157. HRMS m/z (ESI): 305.2219 [M+H]$^+$, calcd for $C_{18}H_{28}N_2O_2$: 304.2, Purity: 98% by HPLC (MeOH/0.1% TEA 85:15 (v/v)).

N-(4-(2-(dimethylamino)ethoxy)phenyl)hexanamide(24d)

Light yellow viscous liquid, yield: 74.7%; ^{1}H NMR (400 MHz, DMSO-d_6) δ (ppm): 0.88 (3H，t，*J* = 6.0 Hz，$C\underline{H}_3CH_2CH_2CH_2CH_2CO$)，1.26-1.31 (4H，m，$CH_3C\underline{H}_2C\underline{H}_2CH_2CH_2CO$)，1.56-1.59 (2H，m，$CH_3CH_2CH_2C\underline{H}_2CH_2CO$)，2.25 (2H，t，*J*=6.0 Hz，$CH_3CH_2CH_2CH_2C\underline{H}_2CO$)，2.50 (6H，s，2× $NC\underline{H}_3$)，2.75 (2H，t，*J* =6.0 Hz，$OCH_2C\underline{H}_2$)，3.96 (2H，t，*J* =6.0 Hz，$OC\underline{H}_2CH_2$)，6.85 (2H，d，*J* =8.0 Hz，Ar-H)，7.47 (2H，d，*J* =8.0 Hz，Ar-H)，9.68 (1H，s，N$\underline{H}$). IR (KBr) m/cm^{-1}: 3235, 2953, 1257, 1157. HRMS m/z (ESI): 279.2068 [M+H]$^+$, calcd for $C_{16}H_{26}N_2O_2$: 278.2, Purity: 98% by HPLC (MeOH/0.1% TEA 85:15 (v/v)).

N-(4-(2-(diethylamino)ethoxy)phenyl)hexanamide(24e)

Light yellow viscous liquid, yield 68.8%; ^{1}H NMR (400 MHz, DMSO-d_6) δ (ppm): 0.88 (3H，t，*J* = 6.0 Hz，$C\underline{H}_3CH_2CH_2CH_2CH_2CO$)，0.98 (6H，t，*J*=8.0 Hz，2× $NCH_2C\underline{H}_3$)，1.26-1.33 (4H，m，$CH_3C\underline{H}_2C\underline{H}_2CH_2CH_2CO$)，1.57-1.59 (2H，m，$CH_3CH_2CH_2C\underline{H}_2CH_2CO$)，2.26 (2H，t，*J*=6.0 Hz，$CH_3CH_2CH_2CH_2C\underline{H}_2CO$)，2.55 (4H，m，2× $NC\underline{H}_2CH_3$)，2.76 (2H，t，*J* =6.0 Hz，$OCH_2C\underline{H}_2$)，3.98 (2H，t，*J* =6.0 Hz，$OC\underline{H}_2CH_2$)，6.86 (2H，d，*J* =4.0 Hz，Ar-H)，7.48 (2H，d，*J* =8.0 Hz，Ar-H)，9.71 (1H，s，N$\underline{H}$). IR (KBr) m/cm^{-1}: 3244, 3035, 1255, 1180. HRMS m/z (ESI):307.2377[M+H]$^+$, calcd for $C_{18}H_{30}N_2O_2$: 306.2, Purity: 98% by HPLC (MeOH/0.1% TEA 85:15 (v/v)).

N-(4-(2-(piperidin-1-yl)ethoxy)phenyl)hexanamide(24f)

Light yellow viscous liquid, yield 55.3%; ^{1}H NMR (400 MHz, DMSO-d_6) δ (ppm): 0.88 (3H，t，*J* =6.0 Hz，$C\underline{H}_3CH_2CH_2CH_2CH_2CO$)，1.27-1.32 (4H，m，$CH_3C\underline{H}_2C\underline{H}_2CH_2CH_2CO$)，1.39 (2H，m，$CH_3CH_2CH_2C\underline{H}_2CH_2CO$)，1.51-1.59 (6H，m，piperidine-H)，2.26 (2H，t，*J*=6.0 Hz，$CH_3CH_2CH_2CH_2C\underline{H}_2CO$)，2.48 (4H，m，piperidine-H)，2.68 (2H，t，*J* =6.0 Hz，$OCH_2C\underline{H}_2$)，4.03 (2H，

t，J =6.0 Hz，OC$\underline{H}_2$CH$_2$)，6.87 (2H，d，J =8.0 Hz，Ar-H)，7.48 (2H，d，J =8.0 Hz，Ar-H)，9.73 (1H，s，N$\underline{H}$). IR (KBr) m/cm^{-1}: 3236, 3019, 1275, 1168. HRMS m/z (ESI): 319.2383 $[M+H]^+$, calcd for $C_{19}H_{30}N_2O_2$: 318.2, Purity: 98% by HPLC (MeOH/0.1% TEA 85:15 (v/v)).

N-(4-(2-(pyrrolidin-1-yl)ethoxy)phenyl)hexanamide(24g)

Light yellow viscous liquid, yield 72.4%; ^{1}H NMR (400 MHz, DMSO-d_6) δ (ppm): 0.88 (3H，t，J = 6.0 Hz，C$\underline{H}_3$CH$_2$CH$_2$CH$_2$CH$_2$CO)，1.27-1.33 (4H，m，CH$_3$C$\underline{H}_2$C$\underline{H}_2$CH$_2$CH$_2$CO)，1.55-1.61 (2H，m，CH$_3$CH$_2$CH$_2$C$\underline{H}_2$CH$_2$CO)，1.88 (4H，m，pyrrolidine-H)，2.27 (2H，t，J=6.0 Hz，CH$_3$CH$_2$CH$_2$CH$_2$C$\underline{H}_2$CO)，3.10 (4H，m，pyrrolidine-H)，2.68 (2H，t，J =6.0 Hz，OCH$_2$C$\underline{H}_2$)，4.21 (2H，t，J =6.0 Hz，OC$\underline{H}_2$CH$_2$)，6.92 (2H，d，J =8.0 Hz，Ar-H)，7.53 (2H，d，J =8.0 Hz，Ar-H)，9.81 (1H，s，N$\underline{H}$). IR (KBr) m/cm^{-1}: 3251, 3032, 1215, 1180. HRMS m/z (ESI): 305.2228 $[M+H]^+$, calcd for $C_{18}H_{28}N_2O_2$: 304.2, Purity: 98% by HPLC (MeOH/0.1% TEA 85:15 (v/v)).

Kinetic study

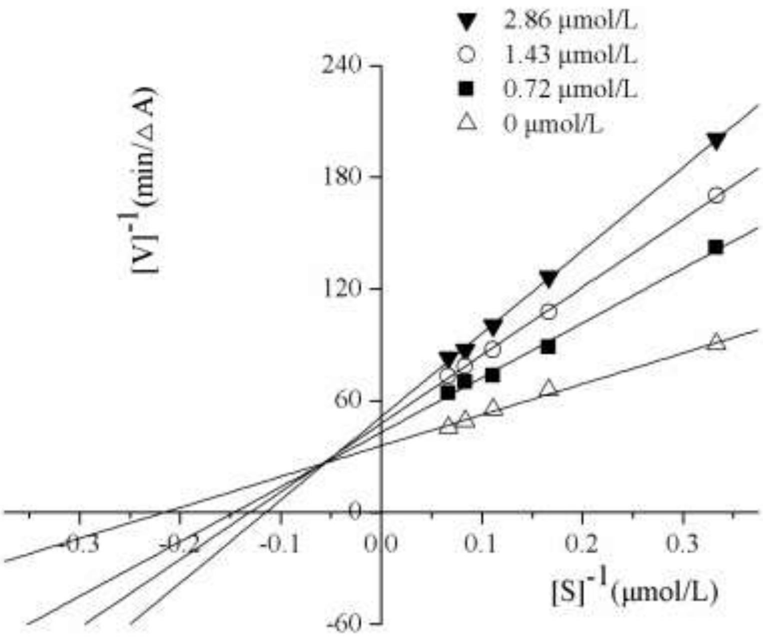

Fig. 2 Lineaweaver–Burk plot for the inhibition of AChE by compound **6g**

Table 2 Kinetic parameters of AChE inhibited by compound **6g**

C (μmol/L)	Michaelis-Menten equation	K_m (mmol/L)	V_{max} (ΔAmin^{-1})	Ki (μmol//L)	Ki’ (μmol/L)
0	$1/v$=165.85/[S]+ 35.88	4.62	0.028	7.27	2.14
0.72	$1/v$=293.49/[S]+ 43.07	6,82	0.023		
1.43	$1/v$=365.67/[S]+ 47.82	7.65	0.021		
2.86	$1/v$=446.46/[S]+ 51.47	8.68	0.019		

Molecular docking

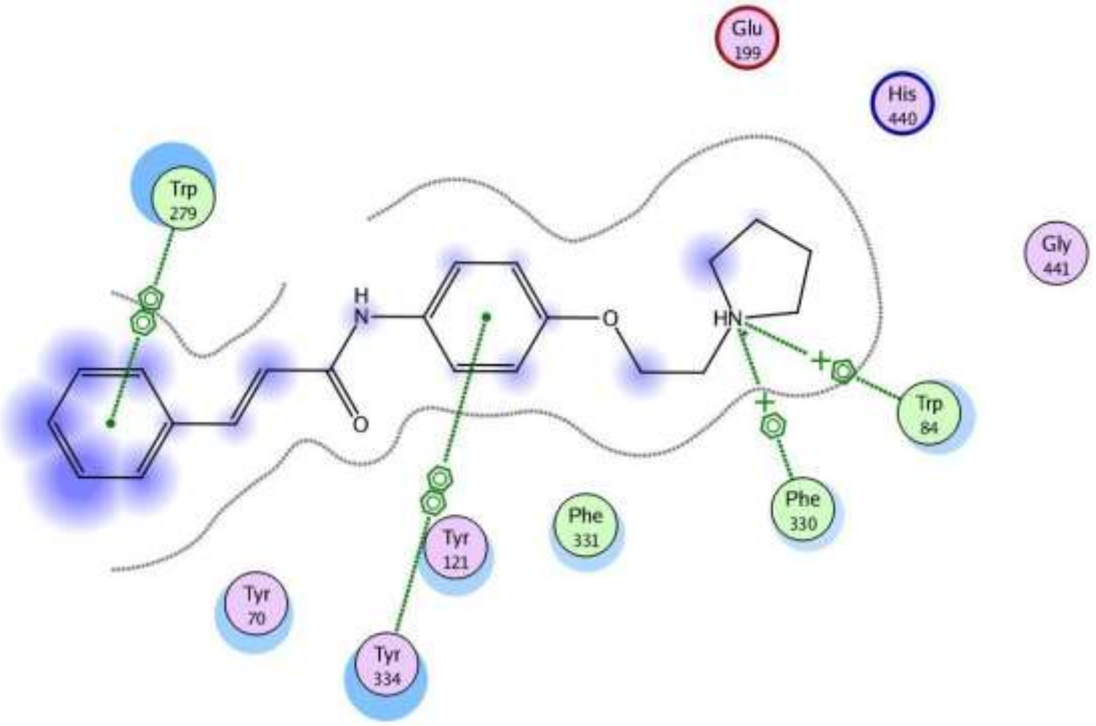

(A)

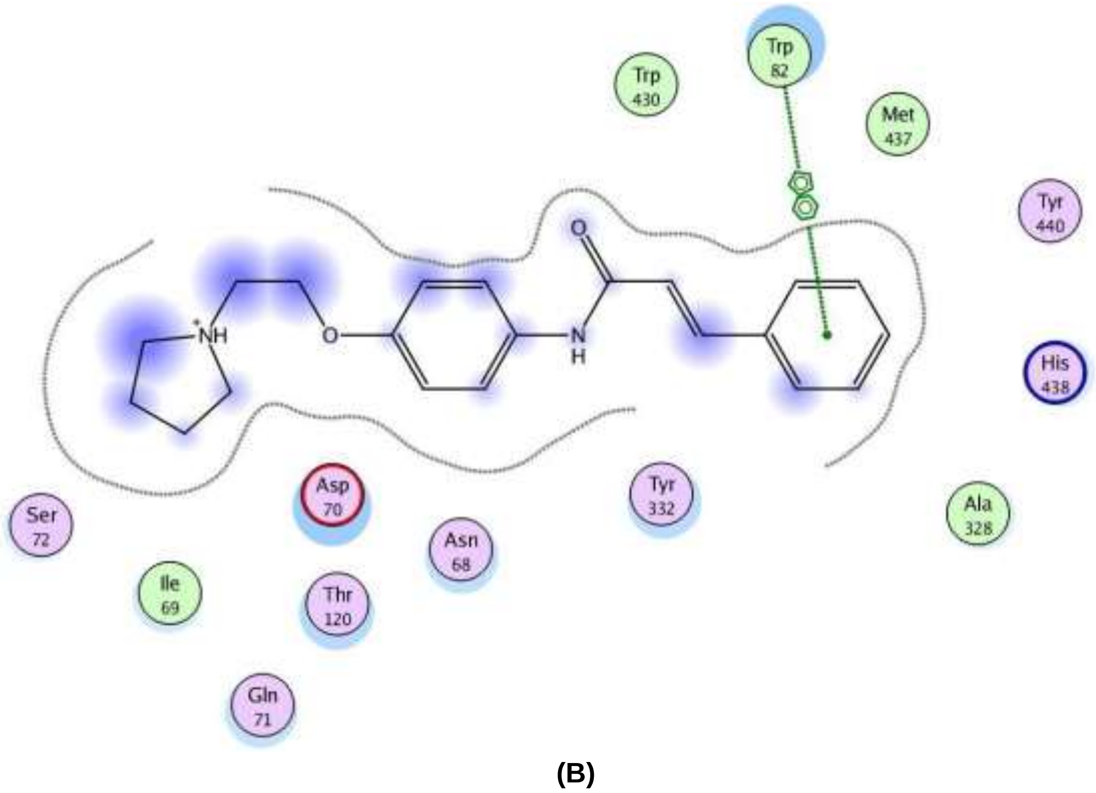

(B)

Fig. 3‡ Molecular modeling of compound **6g** with AChE (A) and BuChE (B) with MOE2008.

The ^{1}H NMR, IR and HRMS of new synthesized compounds

Compounds **4d** is a known compound.

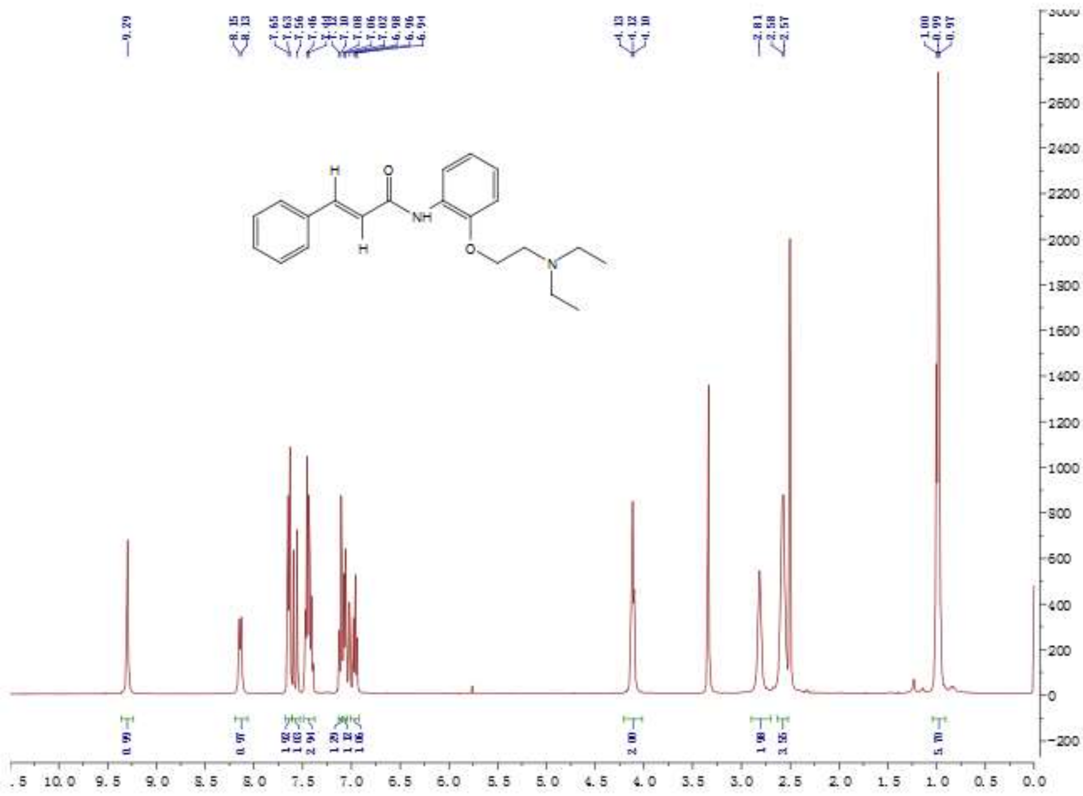

The ^{1}H-NMR (400MHz, DMSO-d_6) of compound **4e**

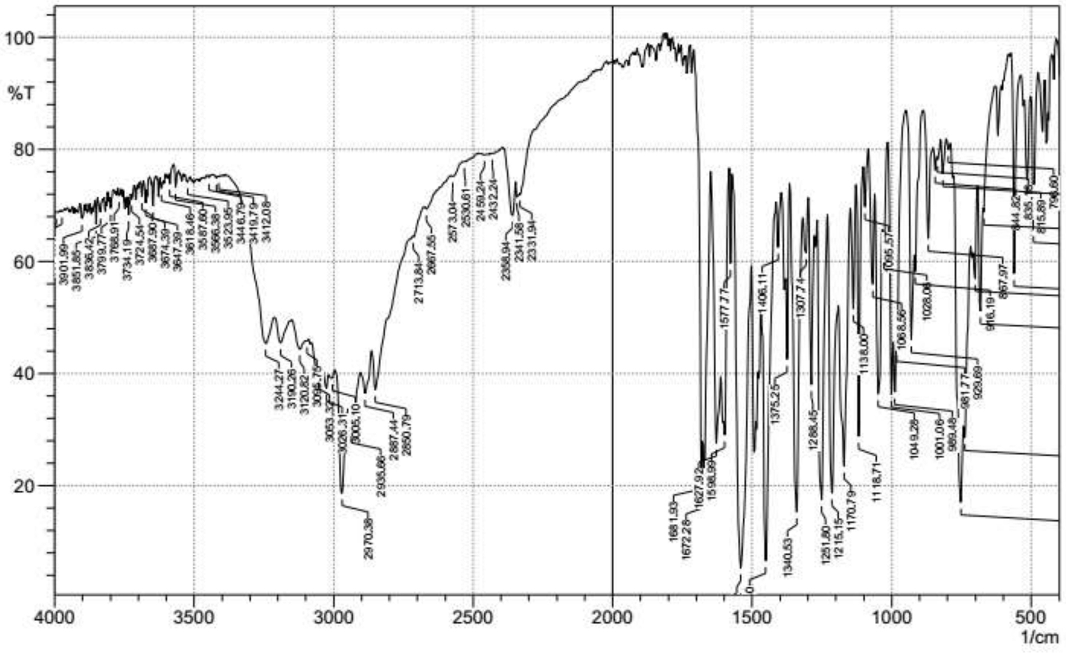

The IR spectra of compound **4e**

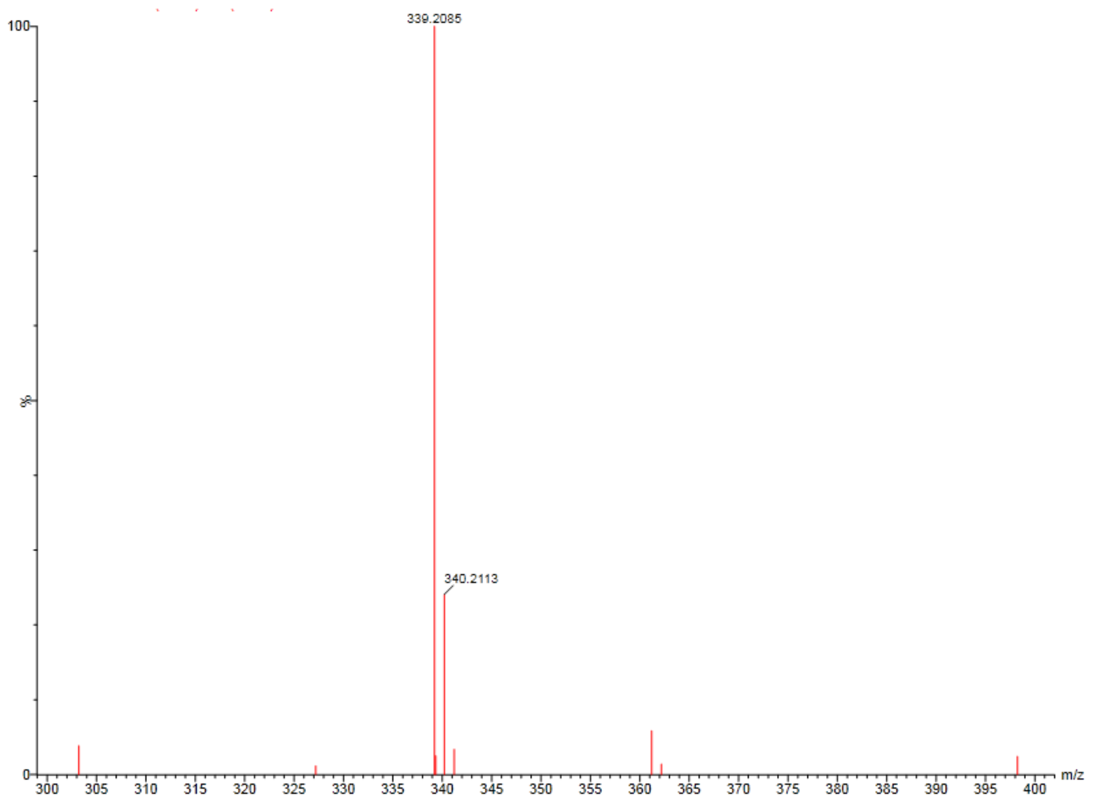

The HRMS of compound **4e**

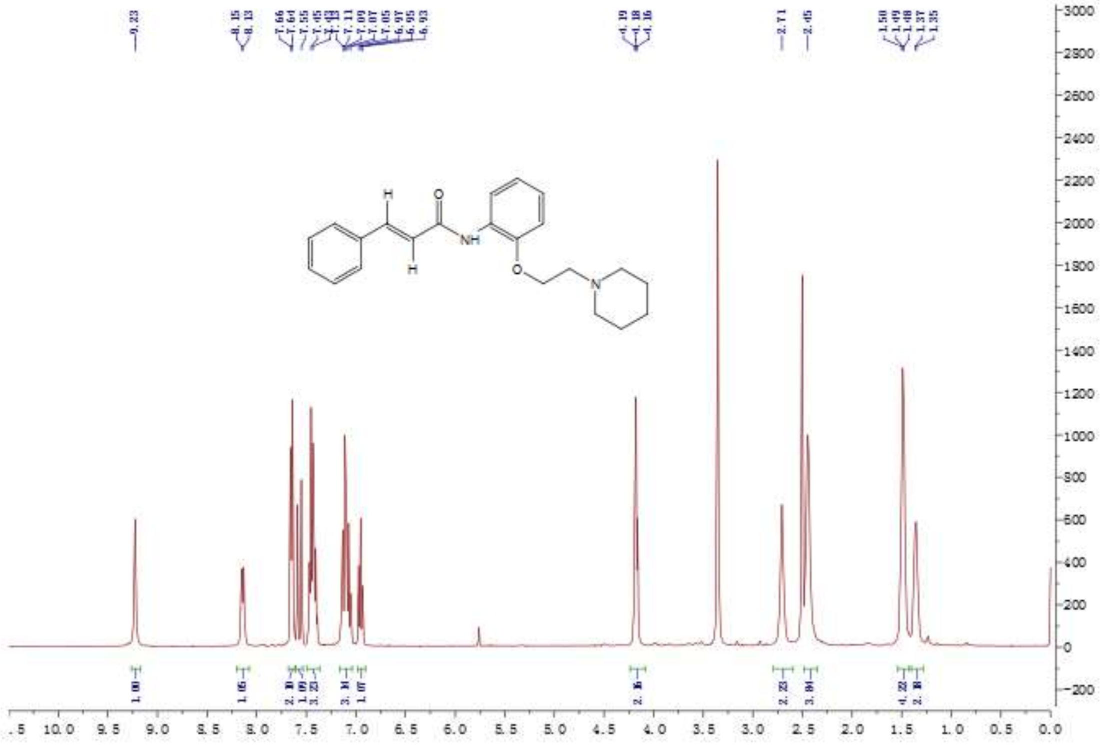

The ^{1}H-NMR (400MHz, DMSO-d_6) of compound **4f**

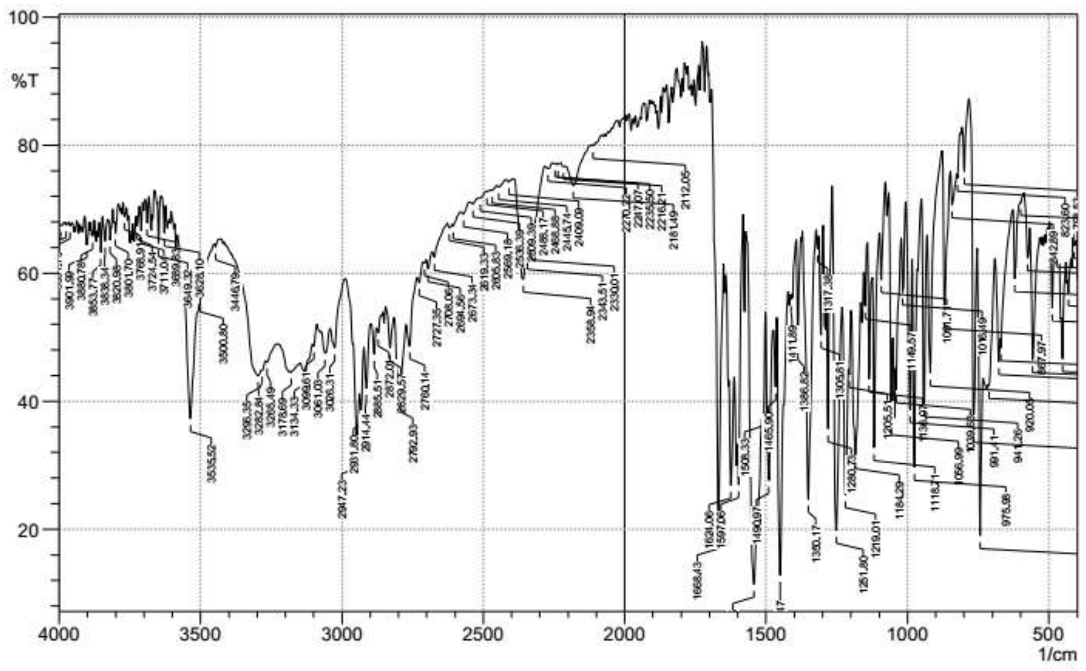

The IR spectra of compound **4f**

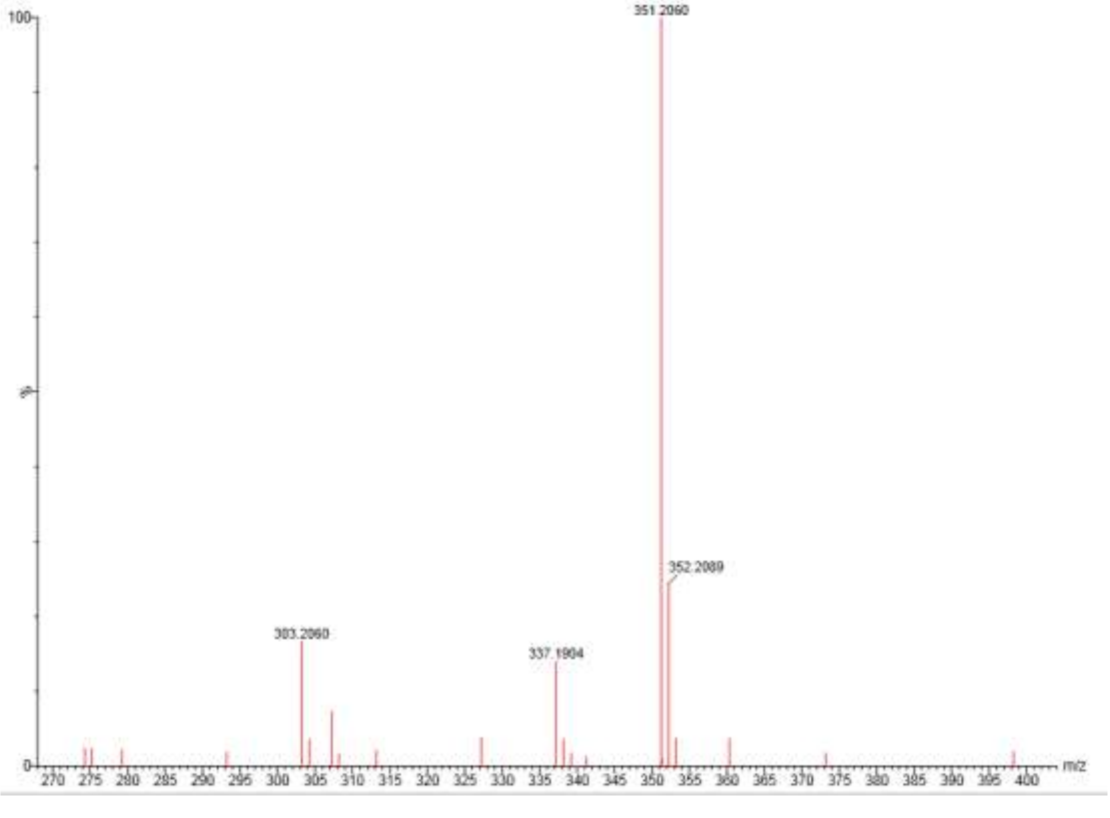

The HRMS of compound **4f**

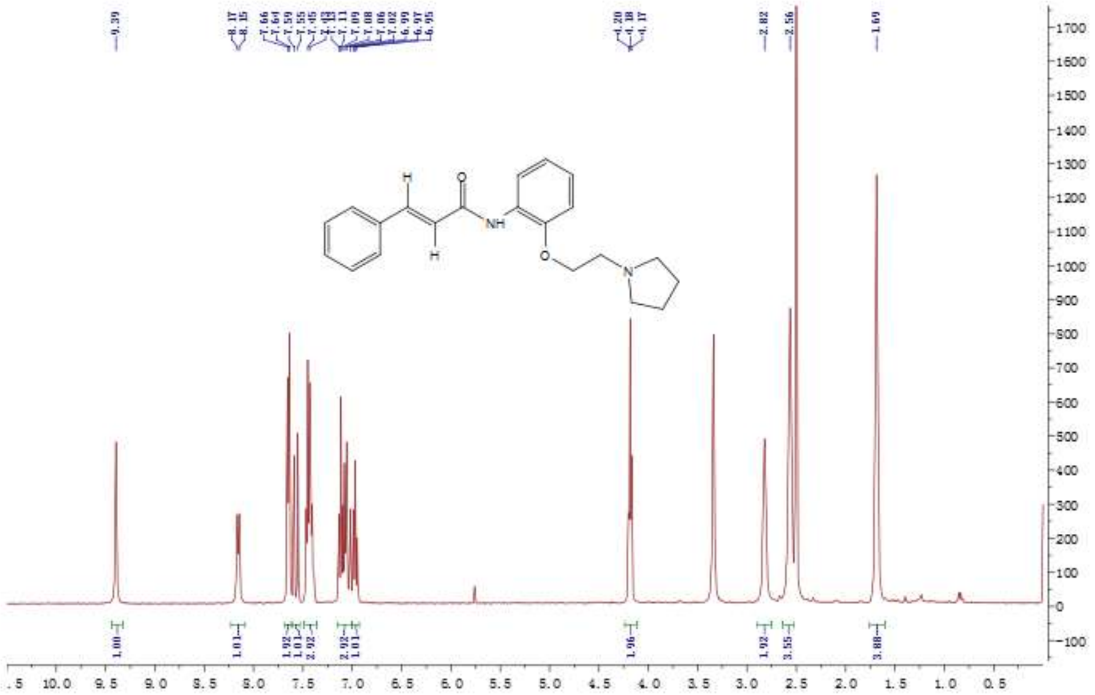

The ^{1}H-NMR (400MHz, DMSO-d_6) of compound **4g**

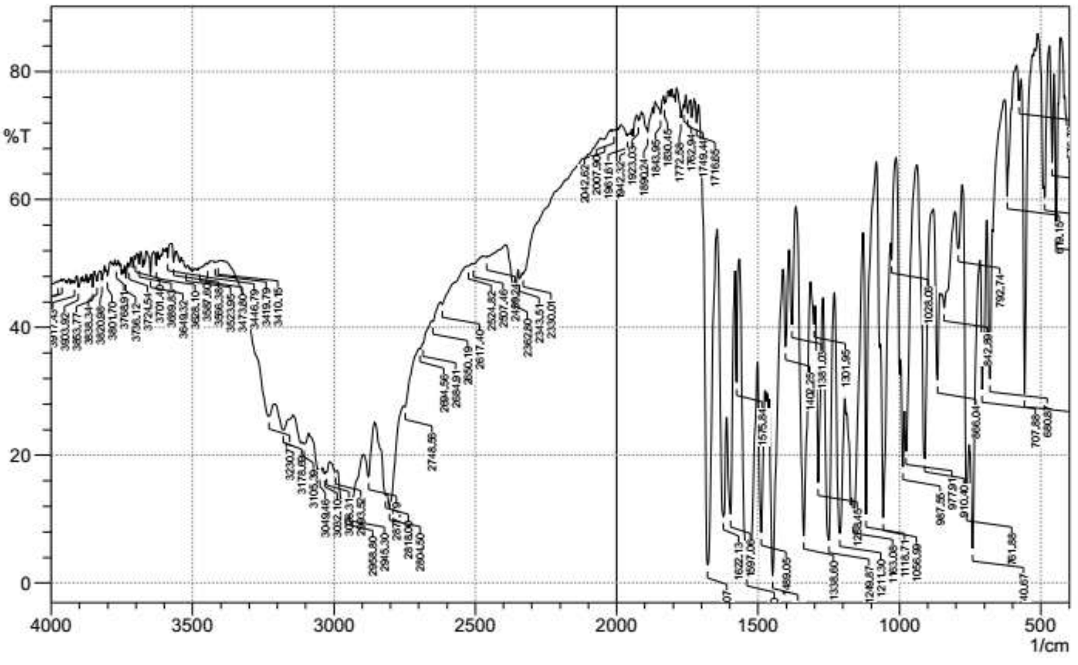

The IR spectra of compound **4g**

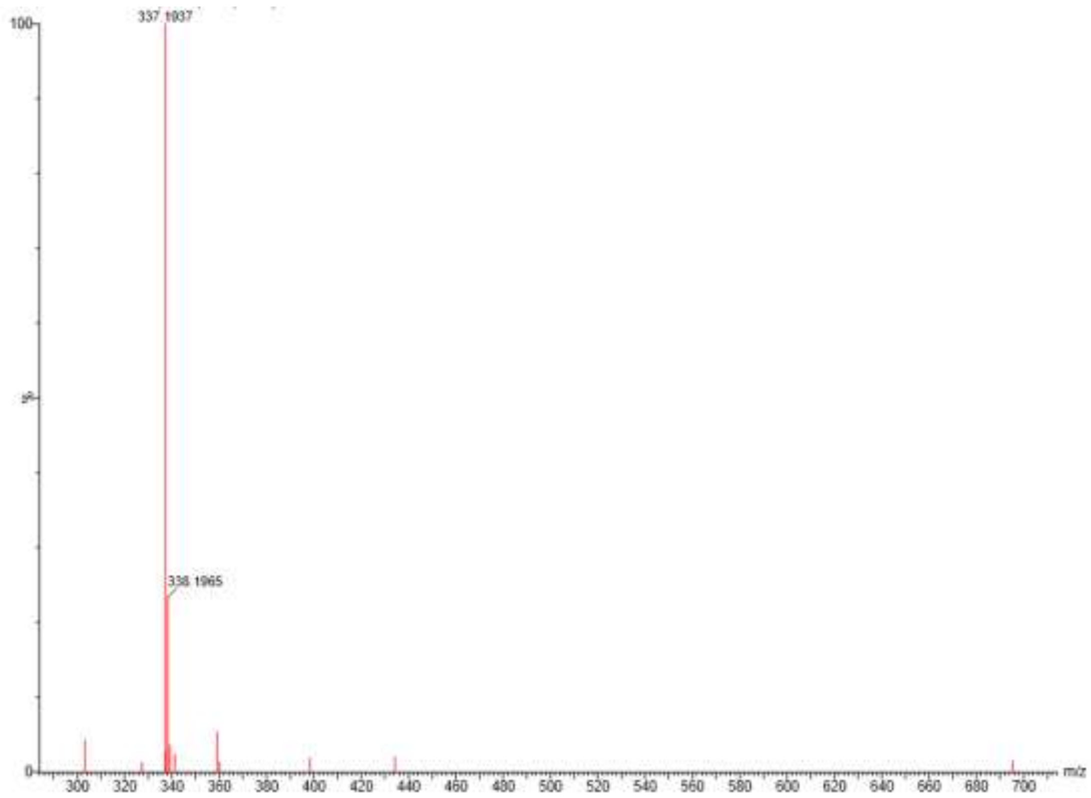

The HRMS of compound **4g**

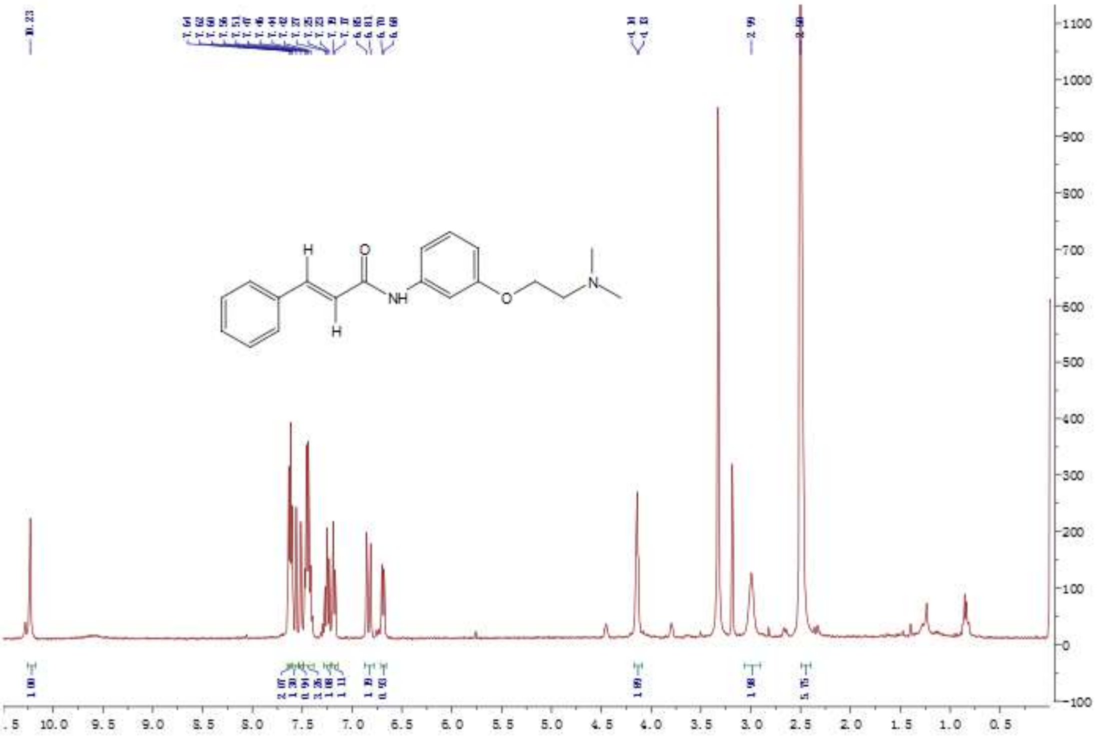

The ^{1}H-NMR (400MHz, DMSO-d_6) of compound **5d**

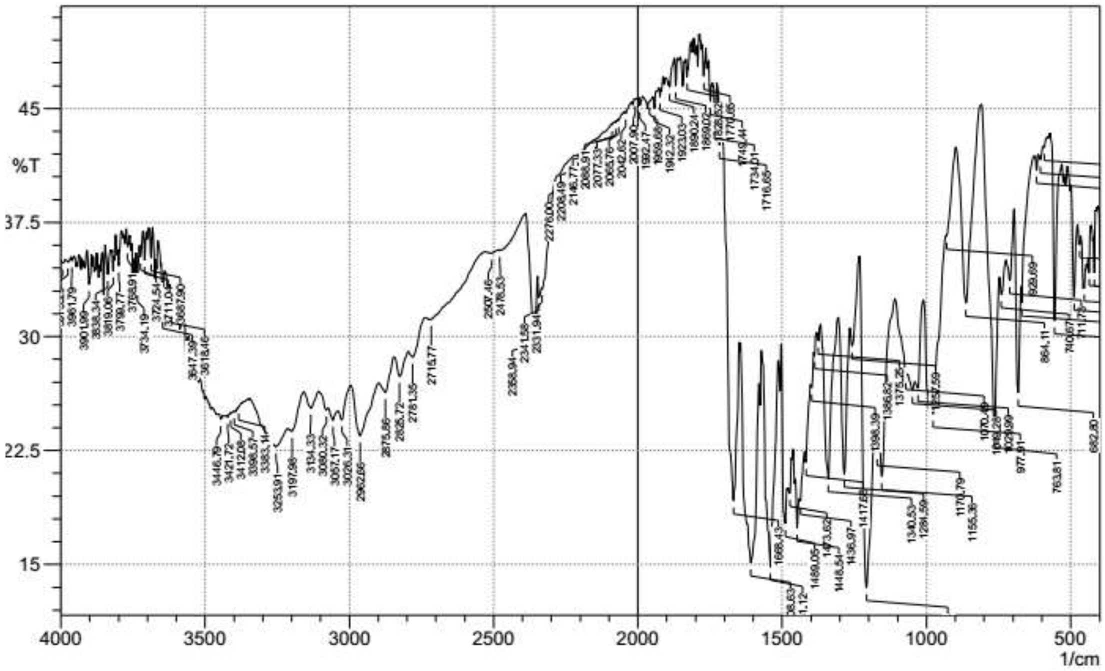

The IR spectra of compound **5d**

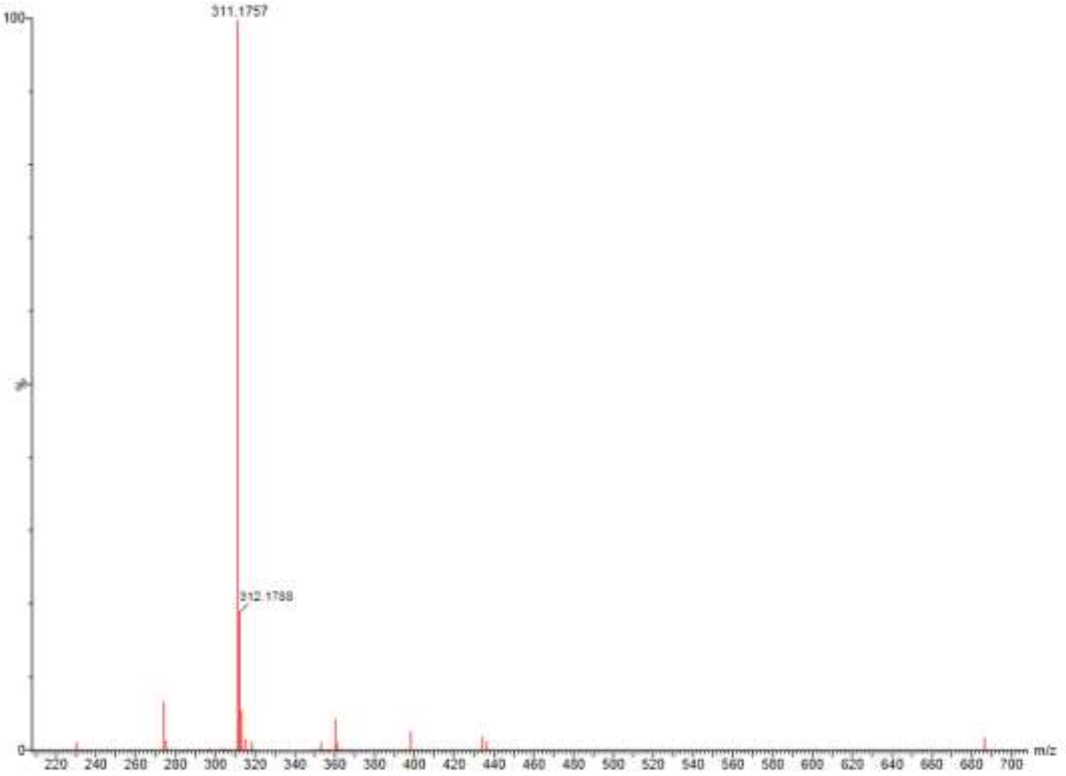

The HRMS of compound **5d**

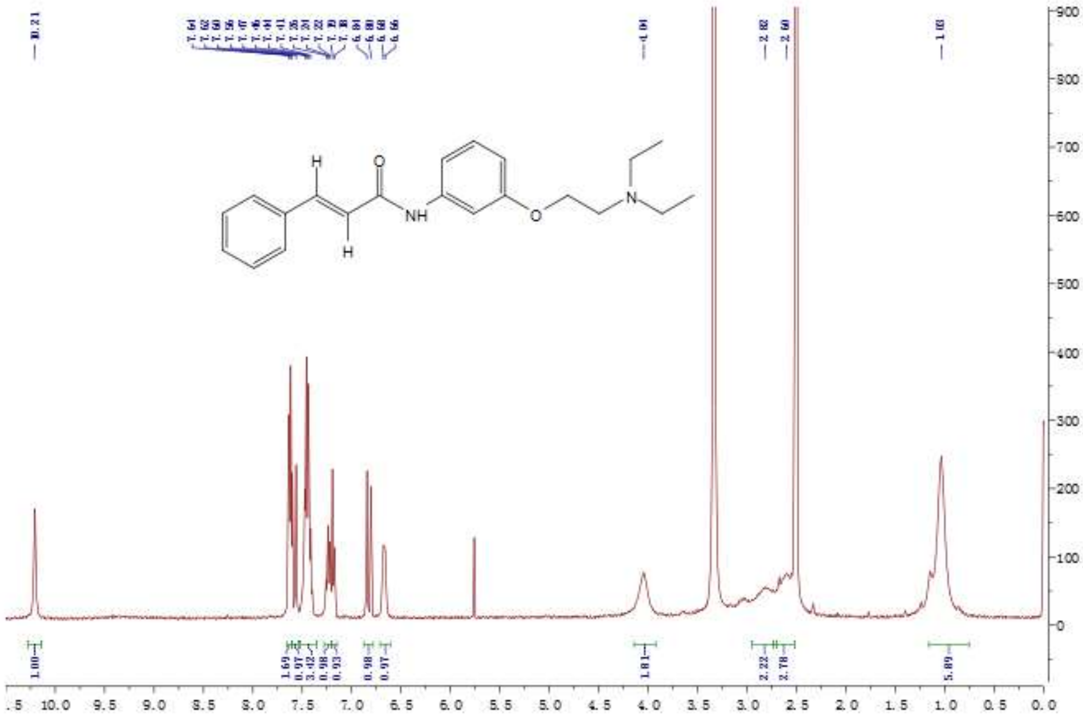

The ^{1}H-NMR (400MHz, DMSO-d_6) of compound **5e**

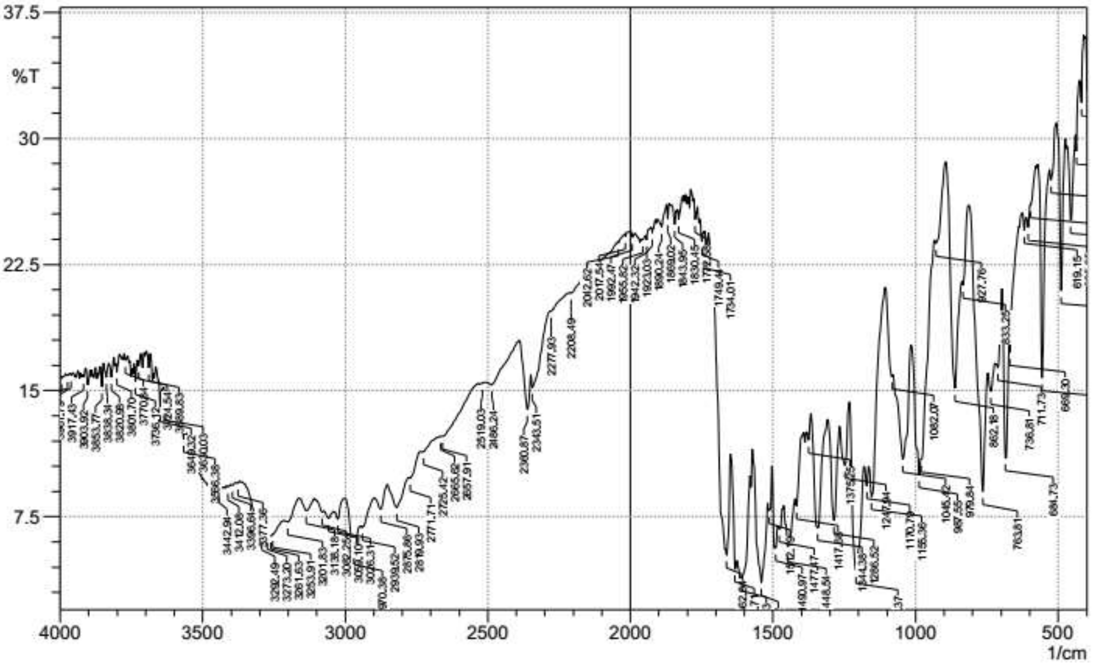

The IR spectra of compound **5e**

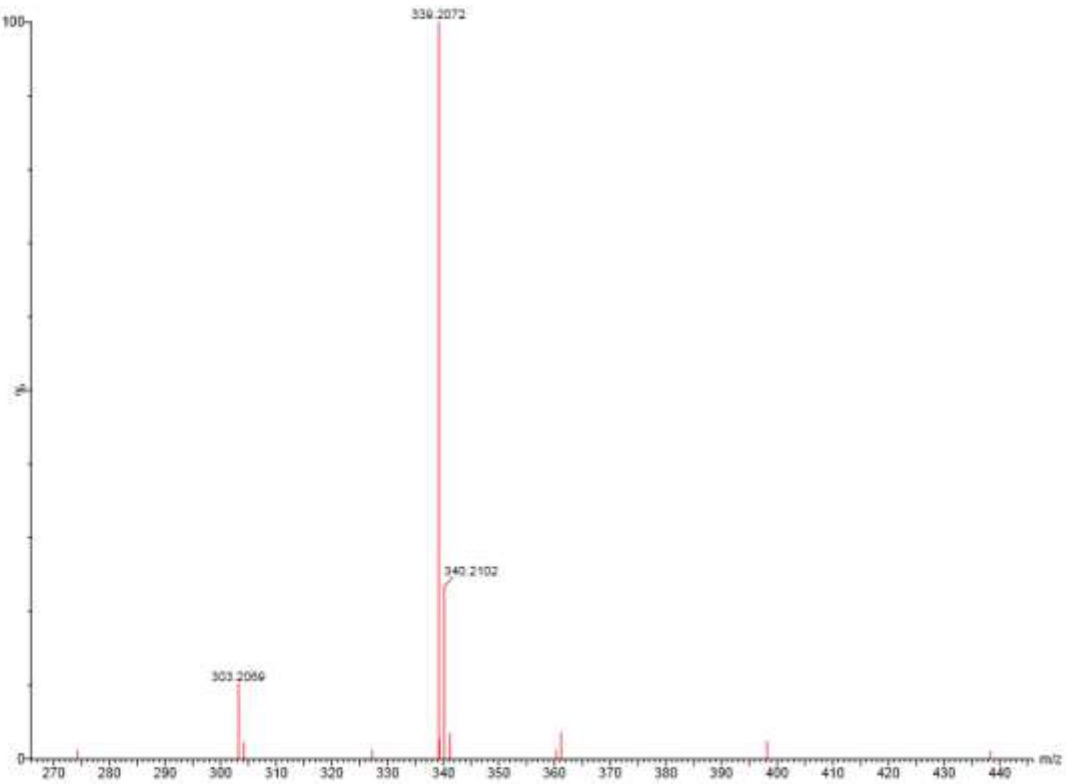

The HRMS of compound **5e**

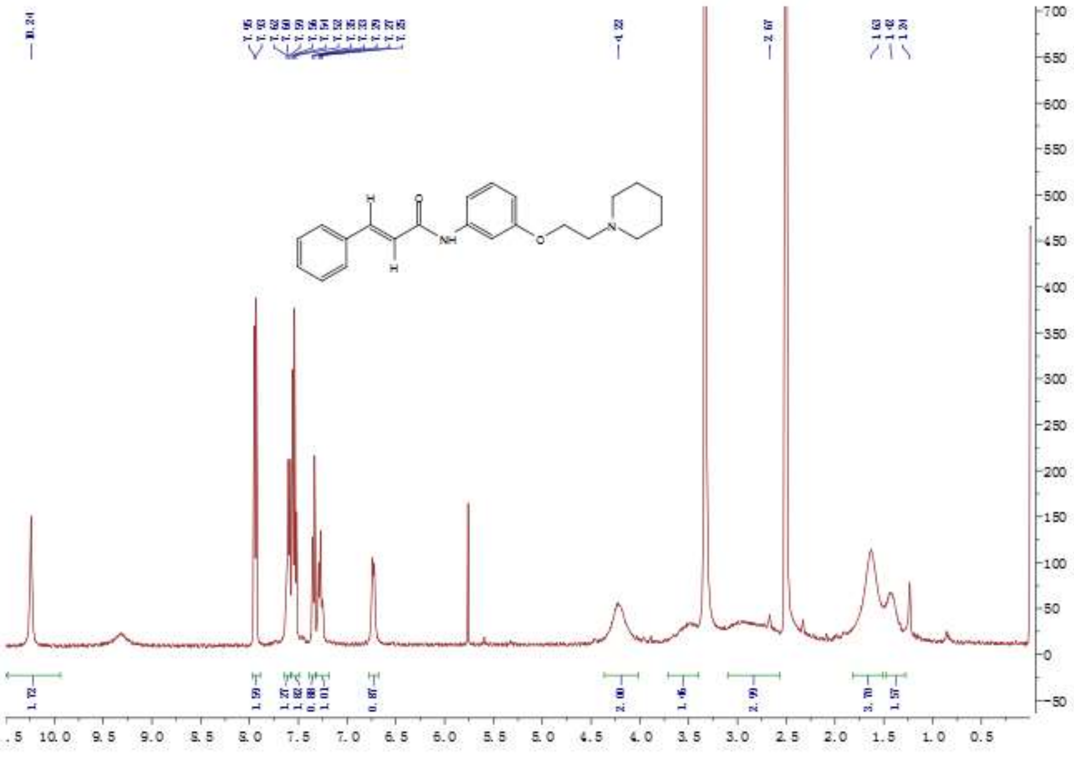

The ^{1}H-NMR (400MHz, DMSO-d_6) of compound **5f**

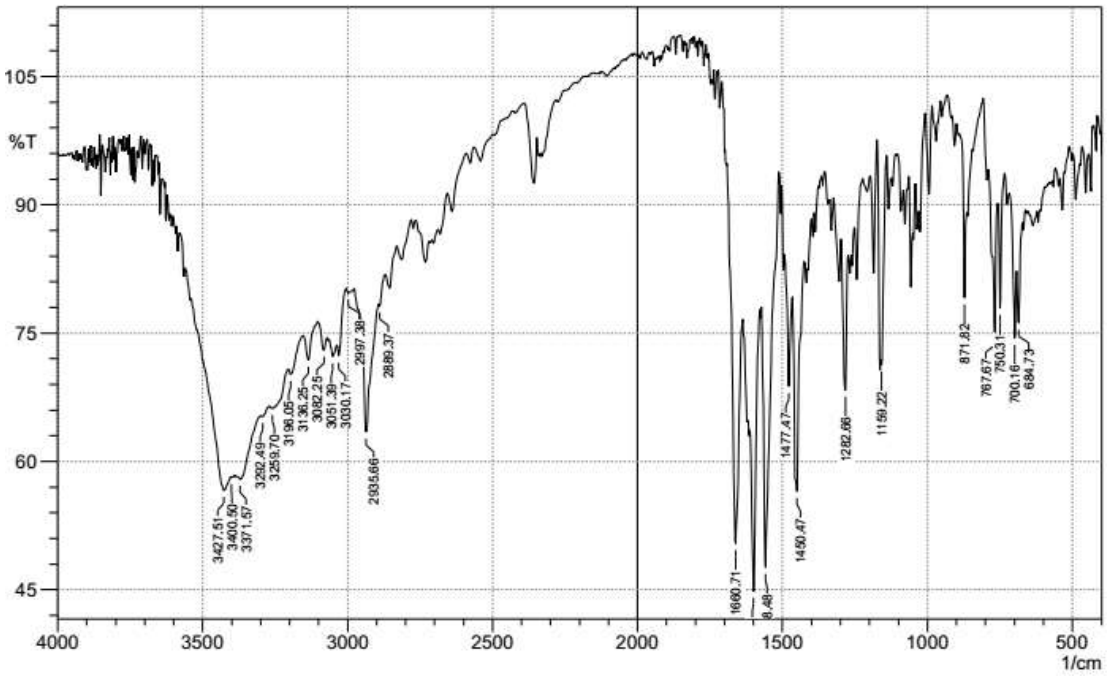

The IR spectra of compound **5f**

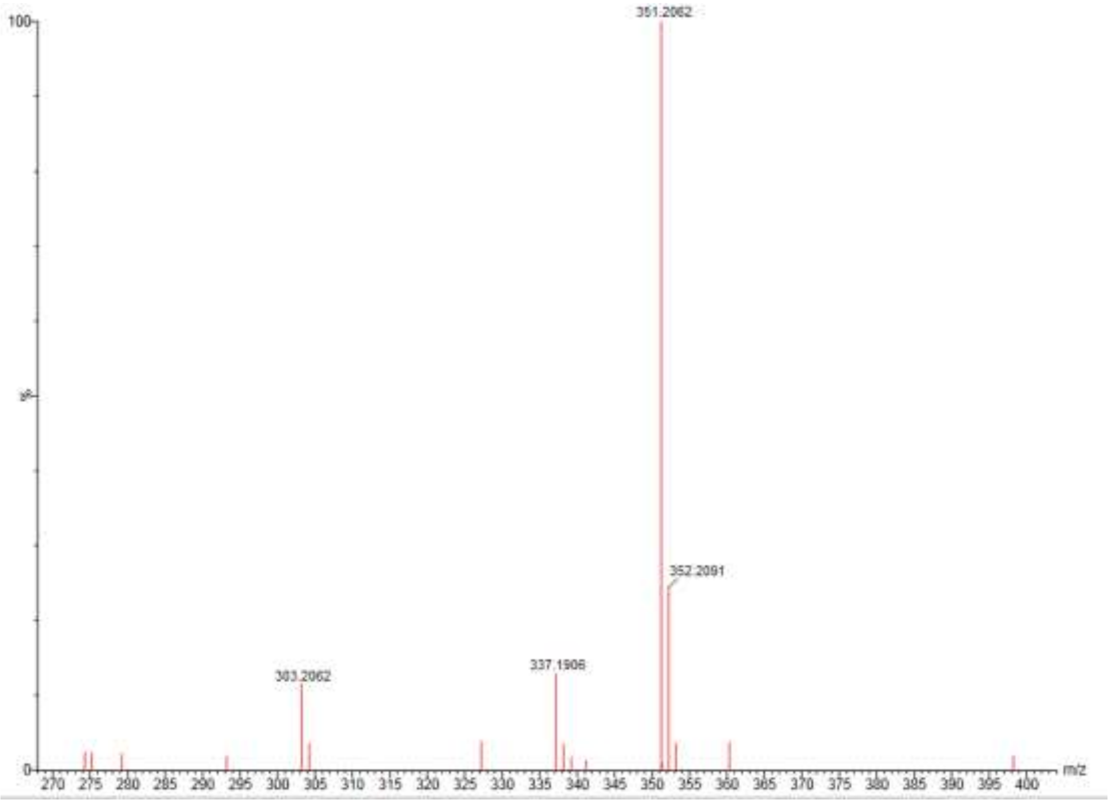

The HRMS of compound **5f**

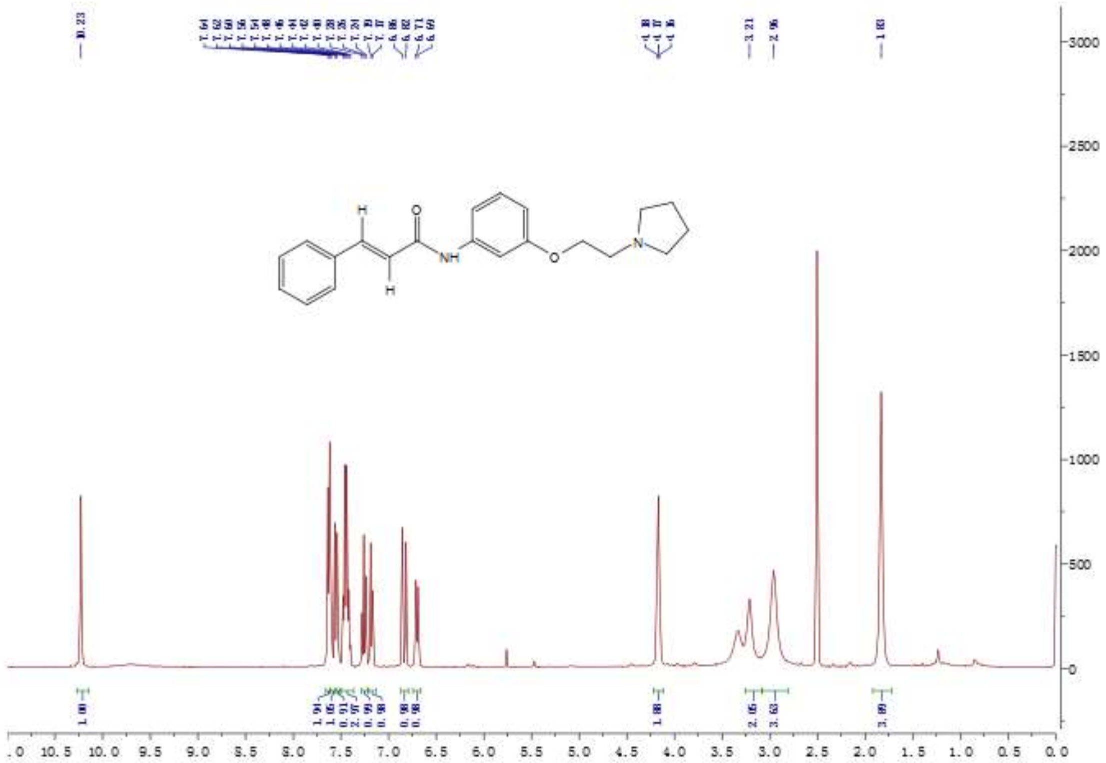

The ^{1}H-NMR (400MHz, DMSO-d_6) of compound **5g**

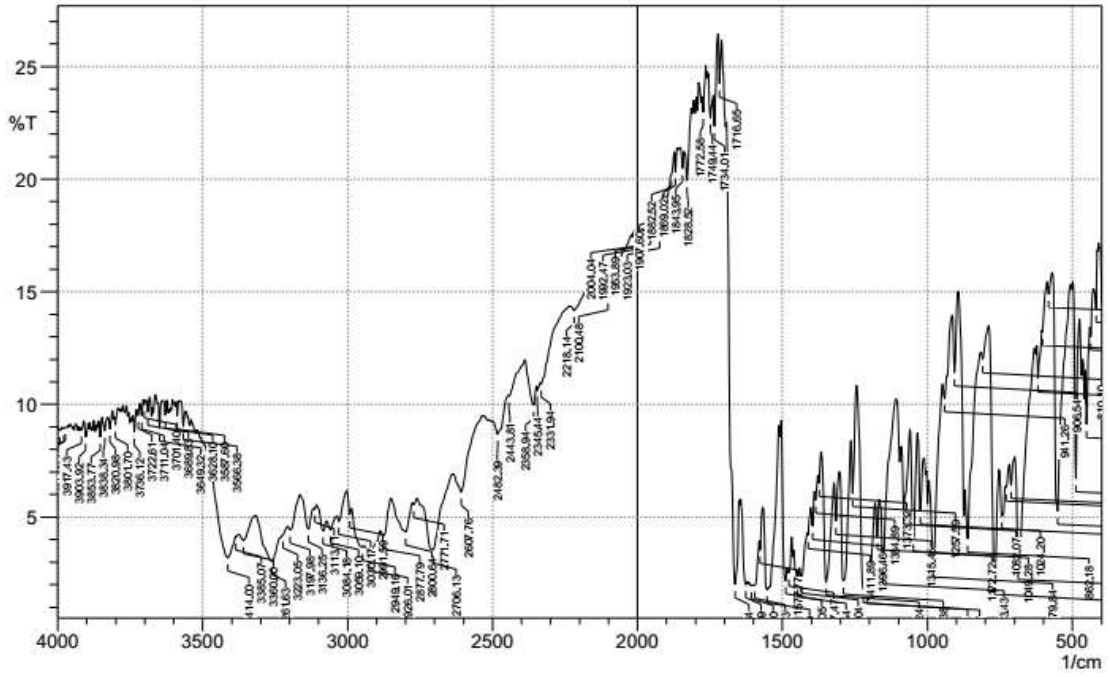

The IR spectra of compound **5g**

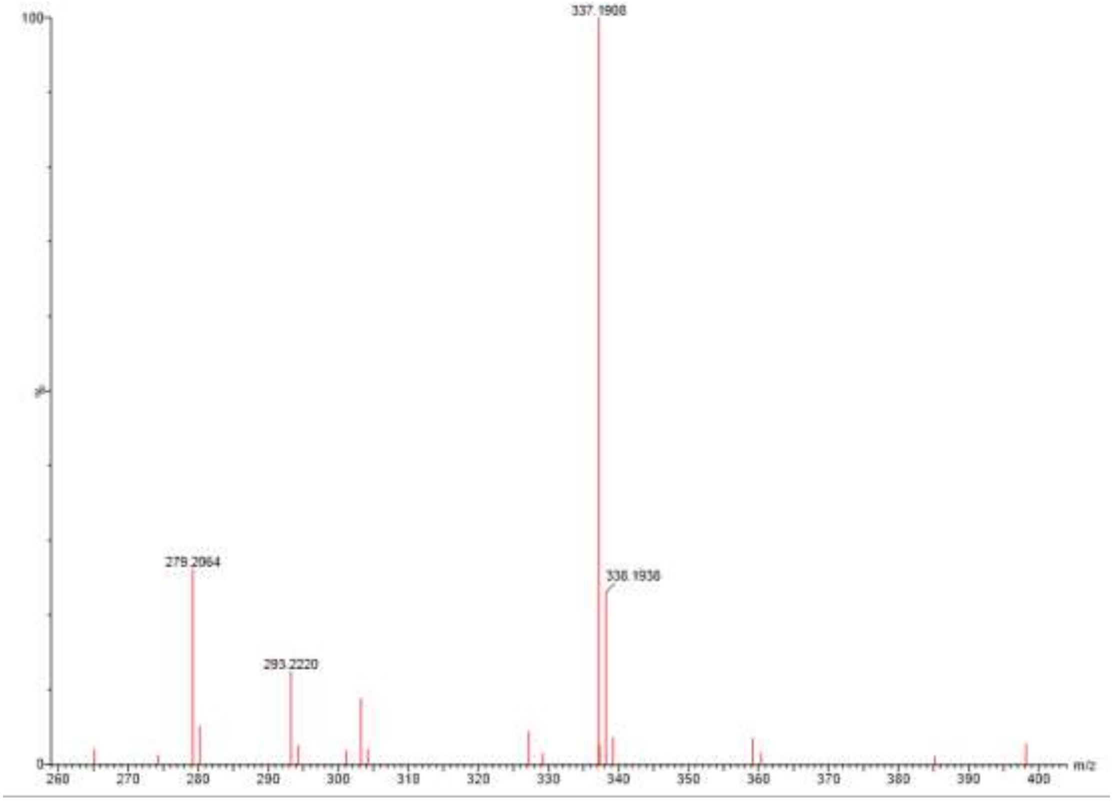

The HRMS of compound **5g**

Compounds **6d** is a known compound.

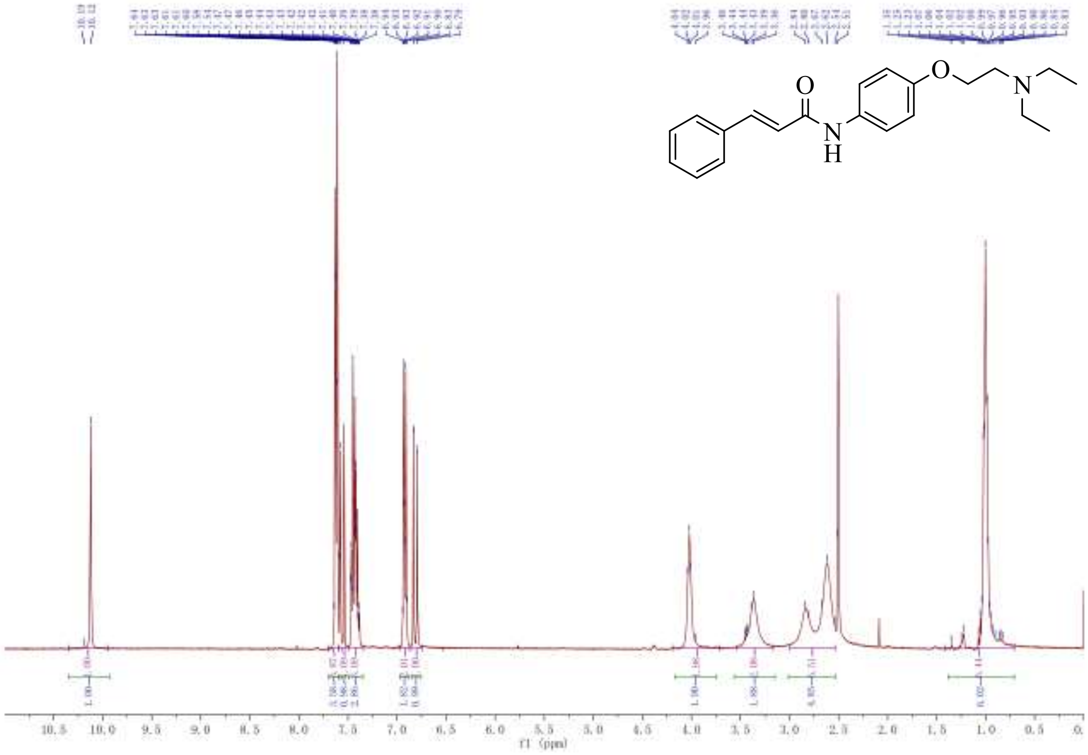

The ^{1}H-NMR (400MHz, DMSO-d_6) of compound **6e**

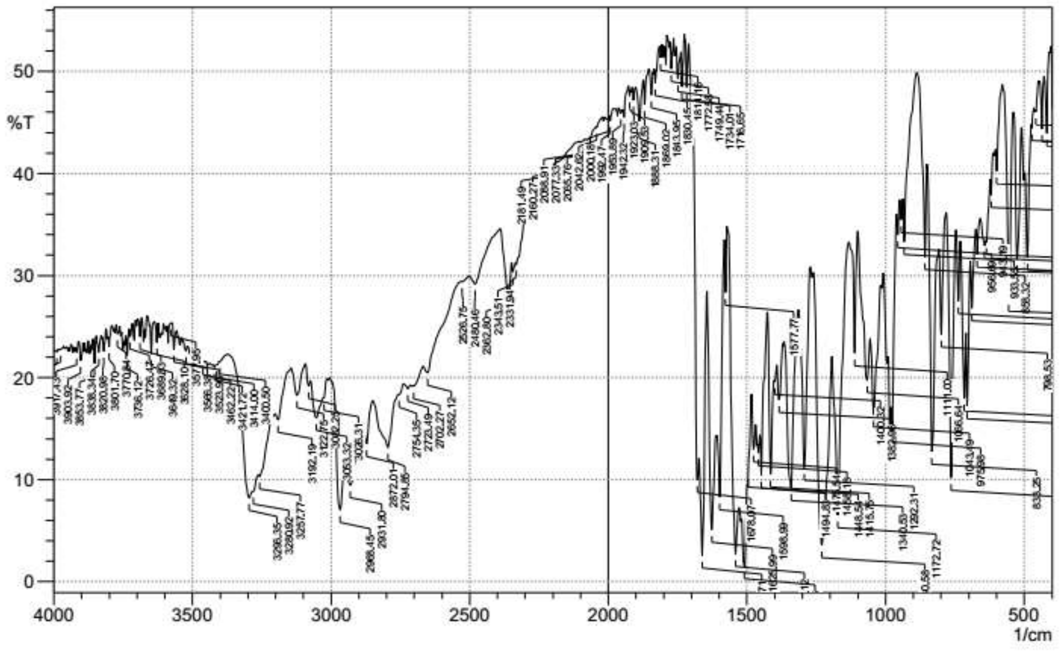

The IR spectra of compound **6e**

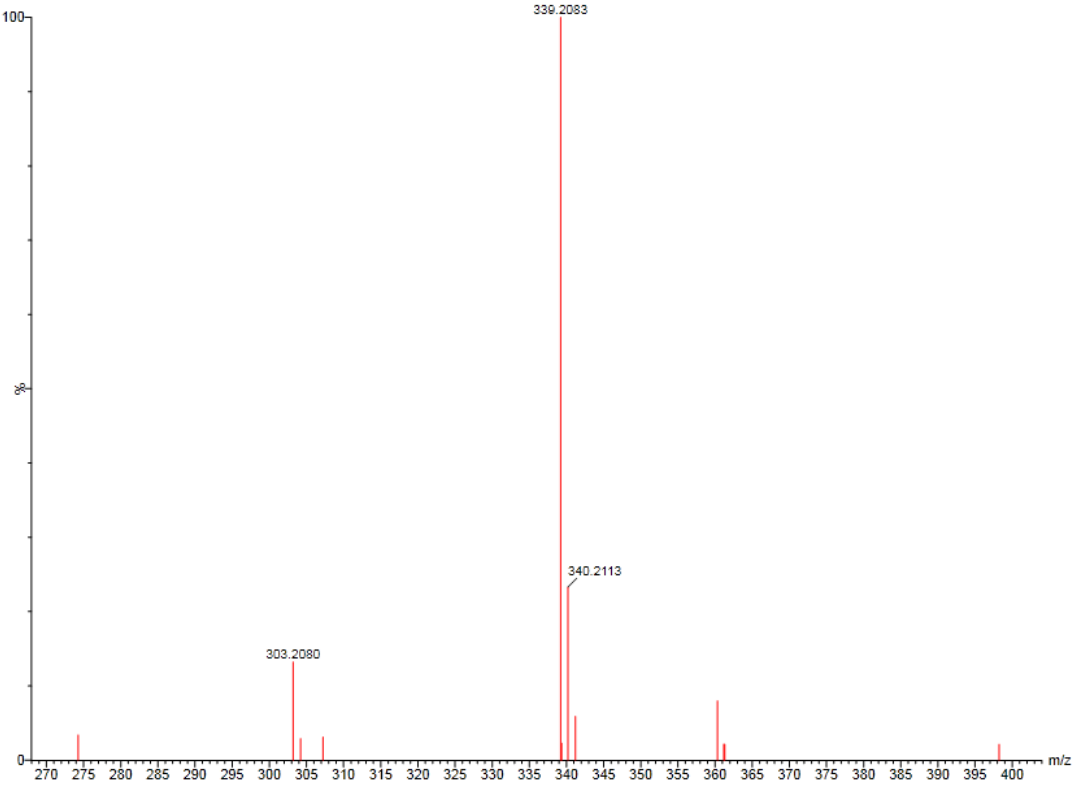

The HRMS of compound **6e**

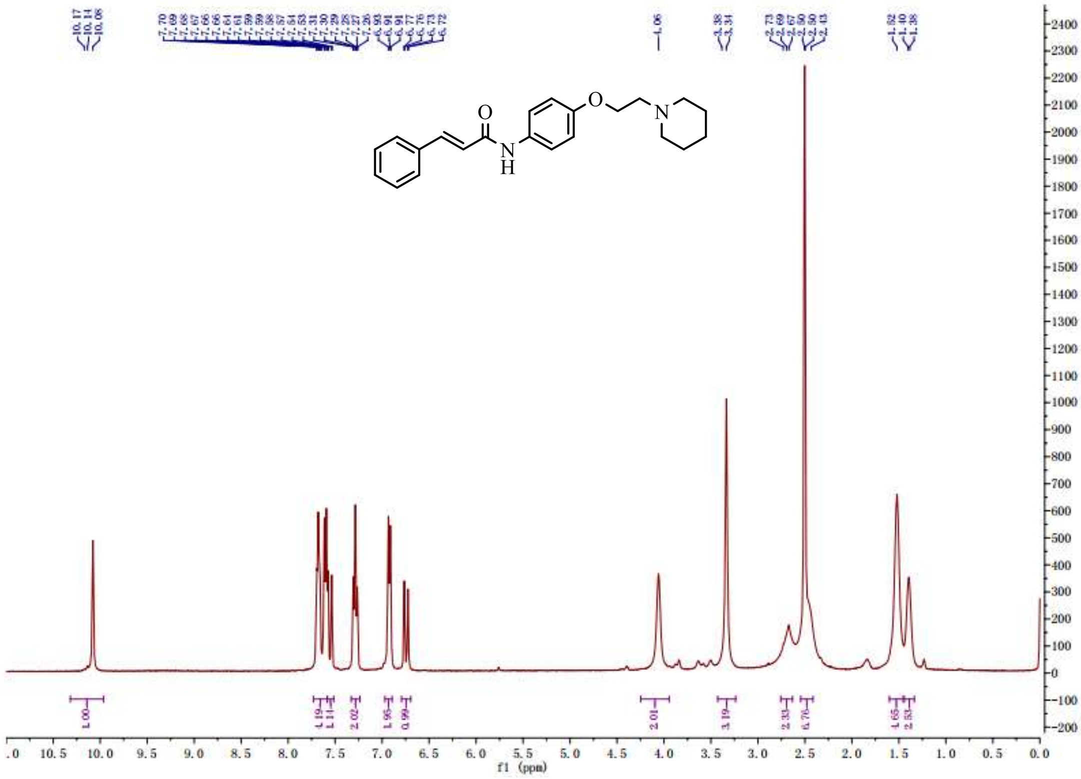

The ^{1}H-NMR (400MHz, DMSO-d_6) of compound **6f**

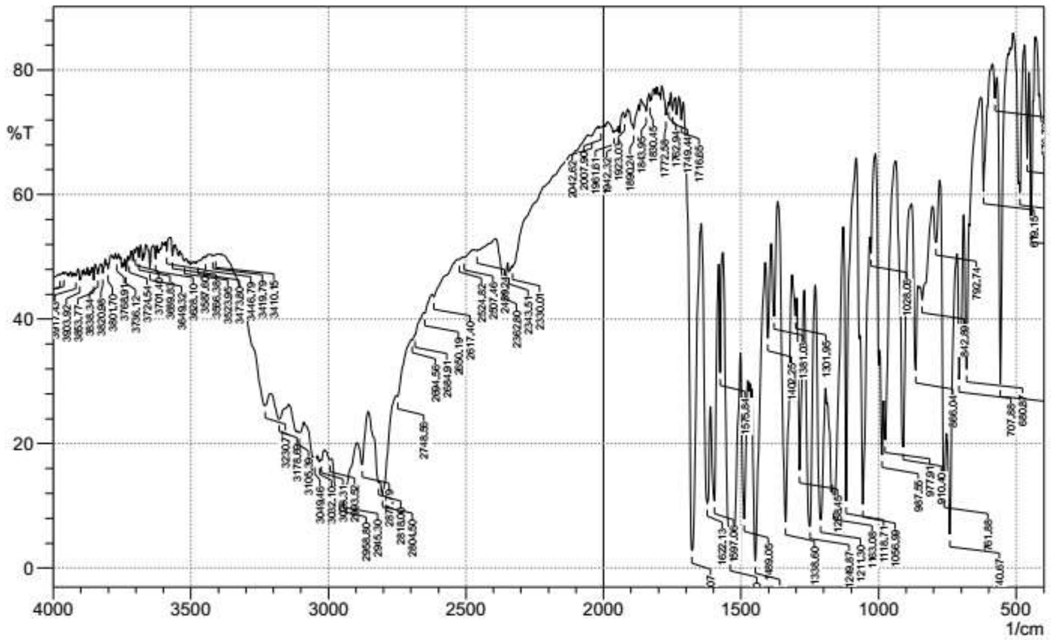

The IR spectra of compound **6f**

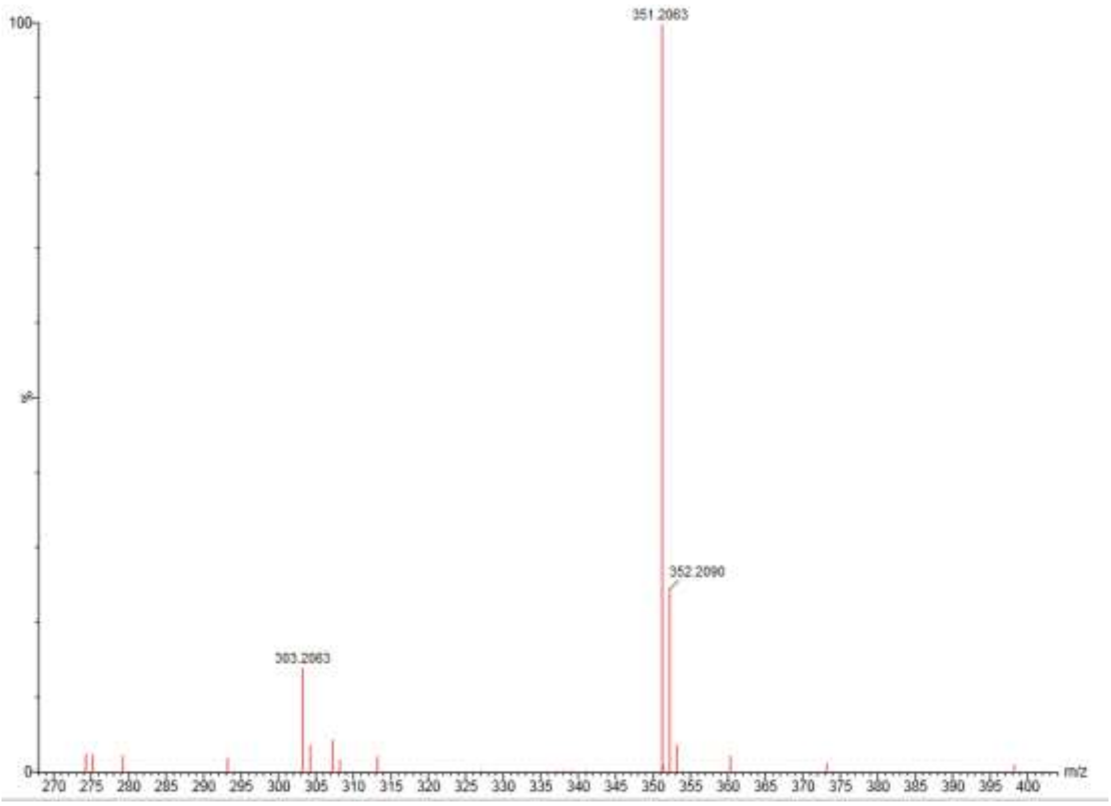

The HRMS of compound **6f**

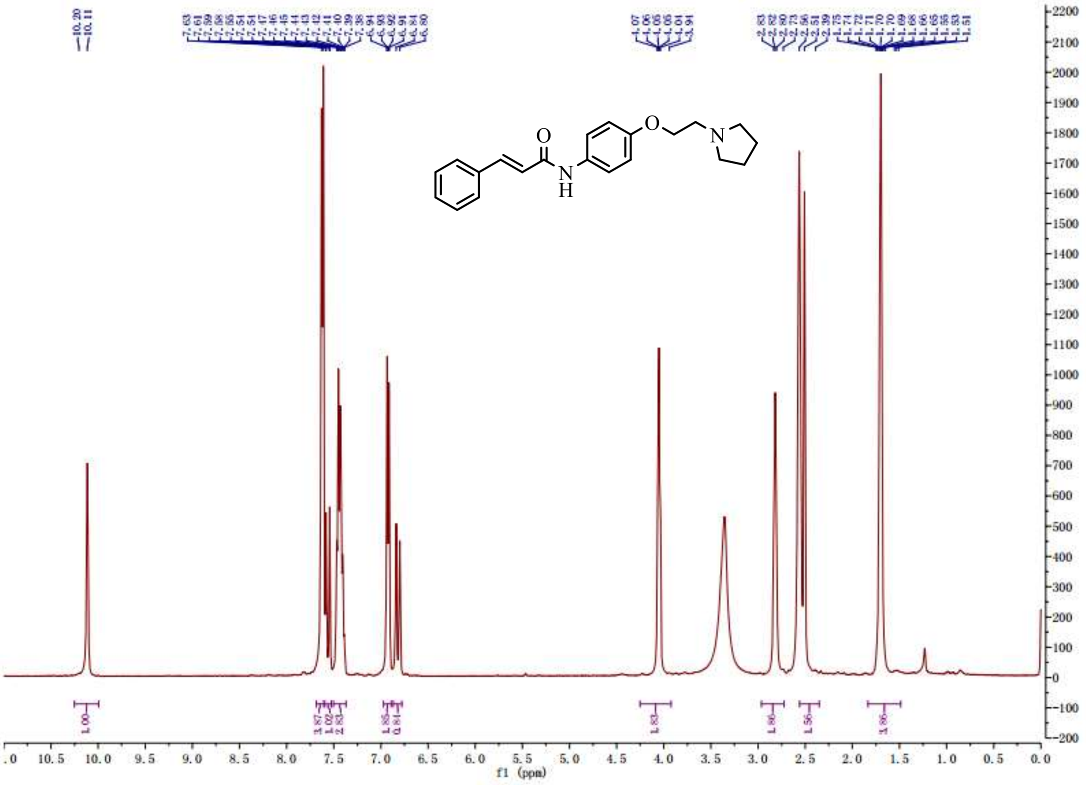

The ^{1}H-NMR (400MHz, DMSO-d_6) of compound **6g**

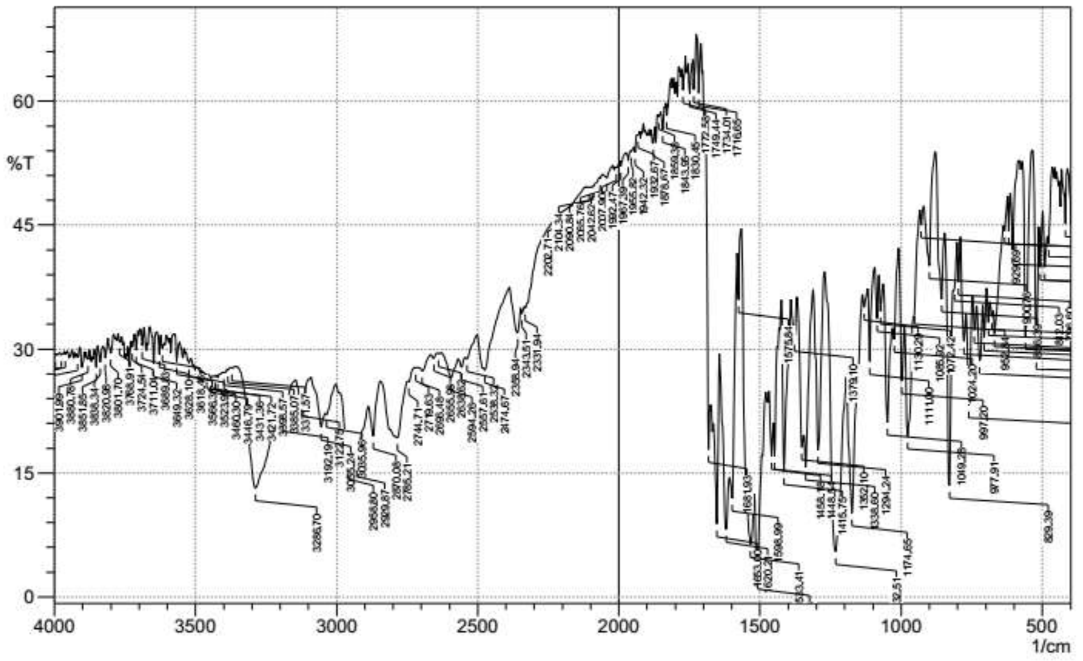

The IR spectra of compound **6g**

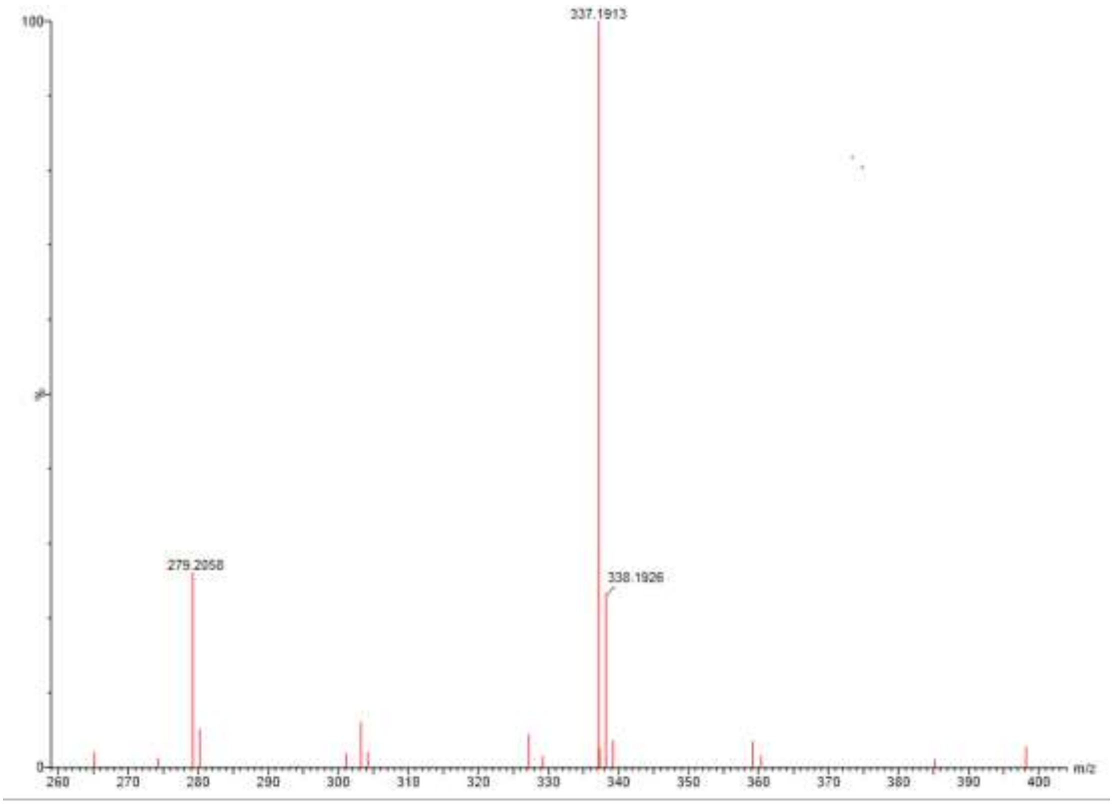

The HRMS of compound **6g**

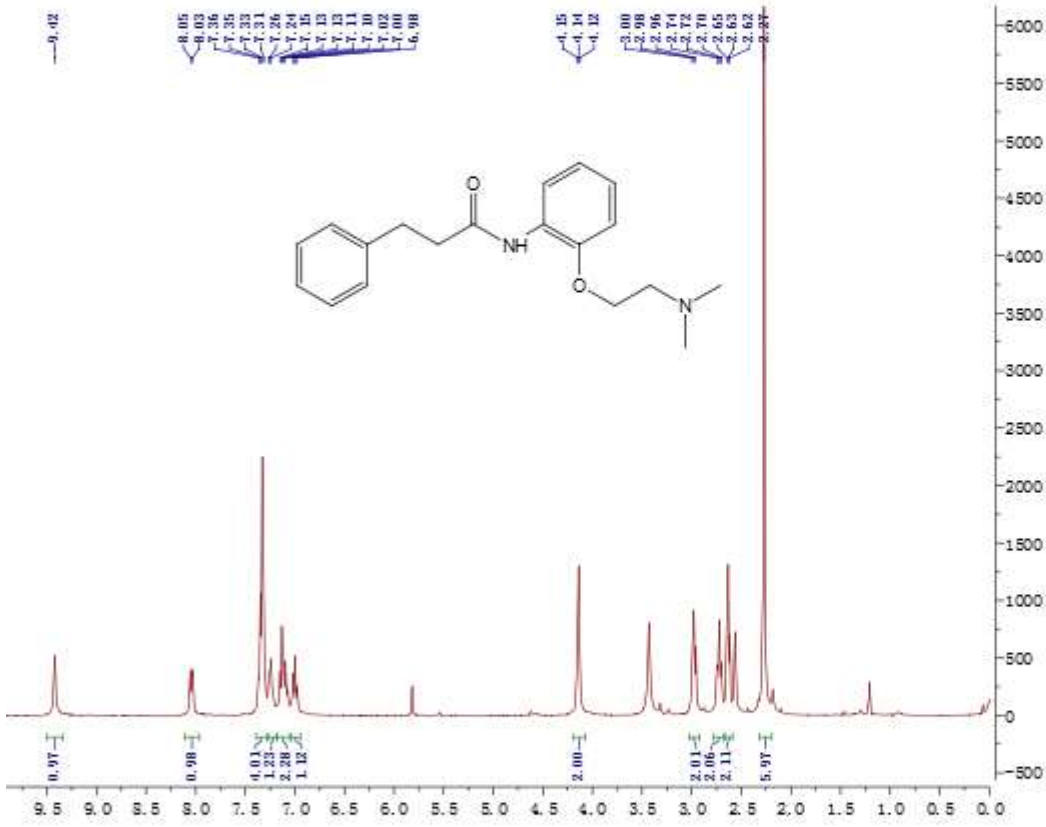

The ^{1}H-NMR (400MHz, DMSO-d_6) of compound **10d**

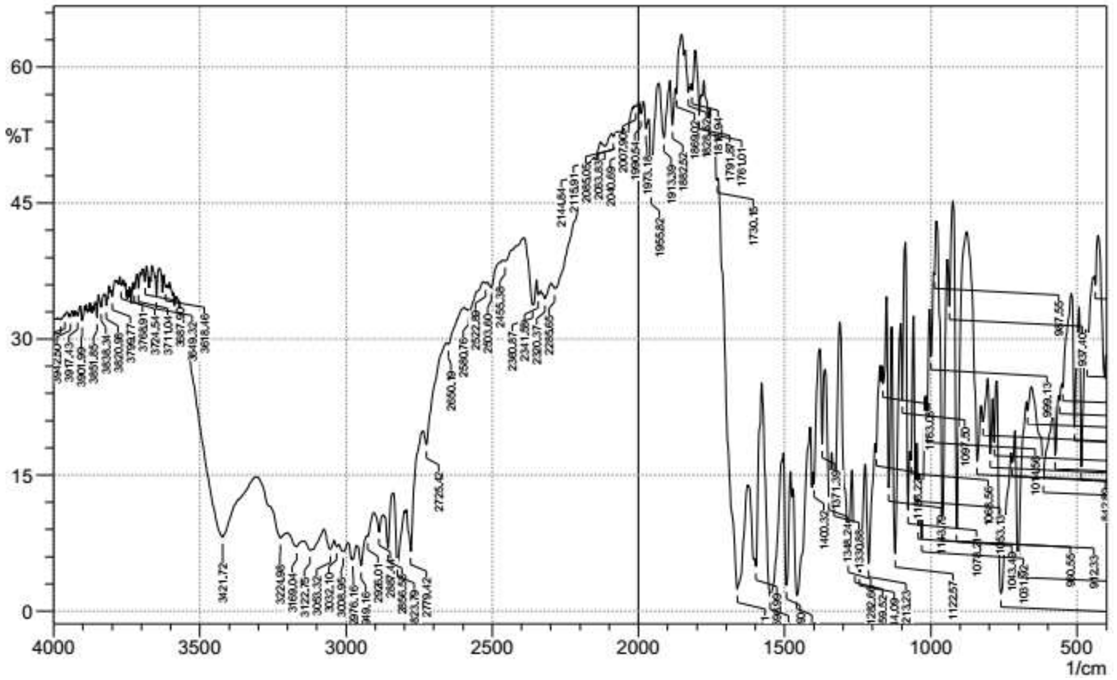

The IR spectra of compound **10d**

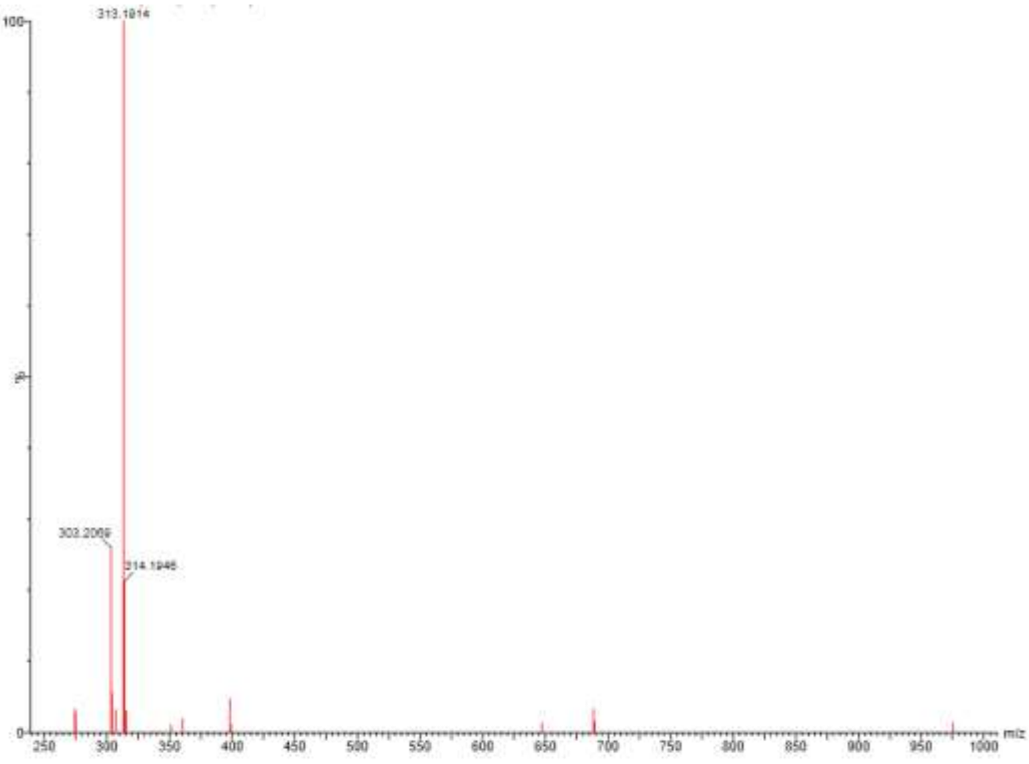

The HRMS of compound **10d**

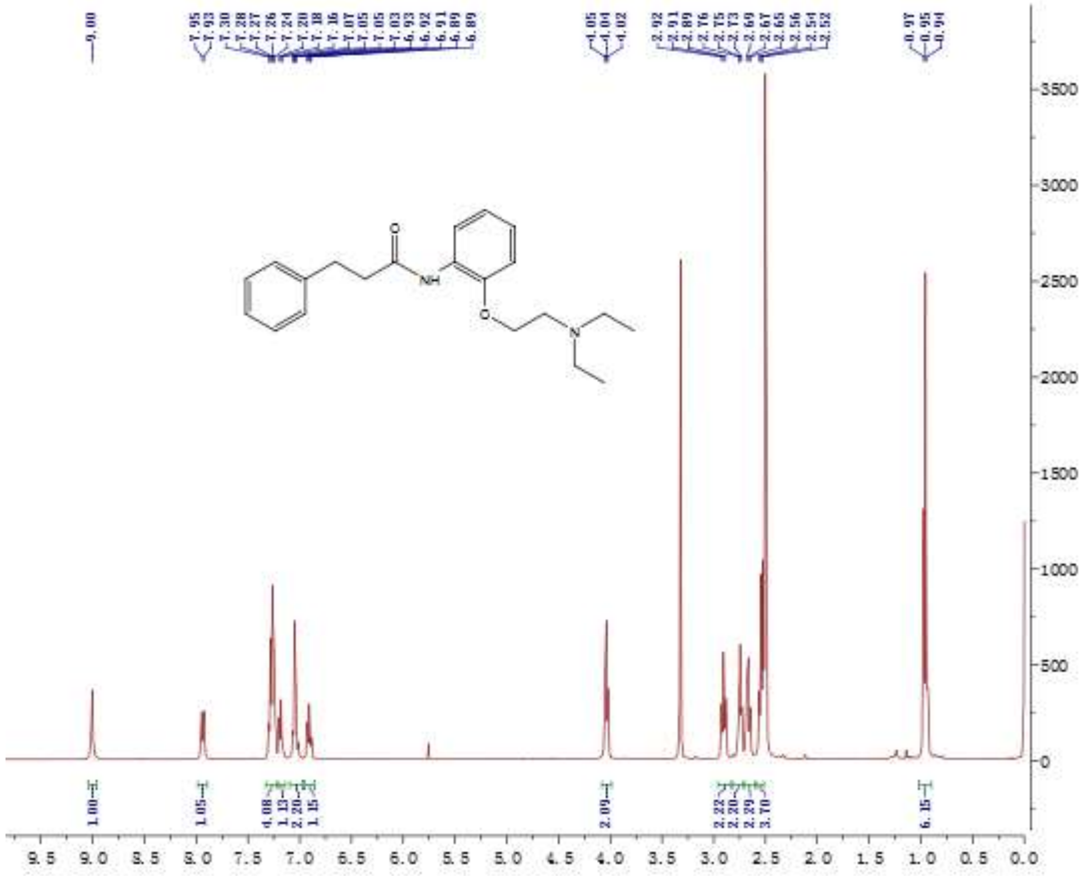

The ^{1}H-NMR (400MHz, DMSO-d_6) of compound **10e**

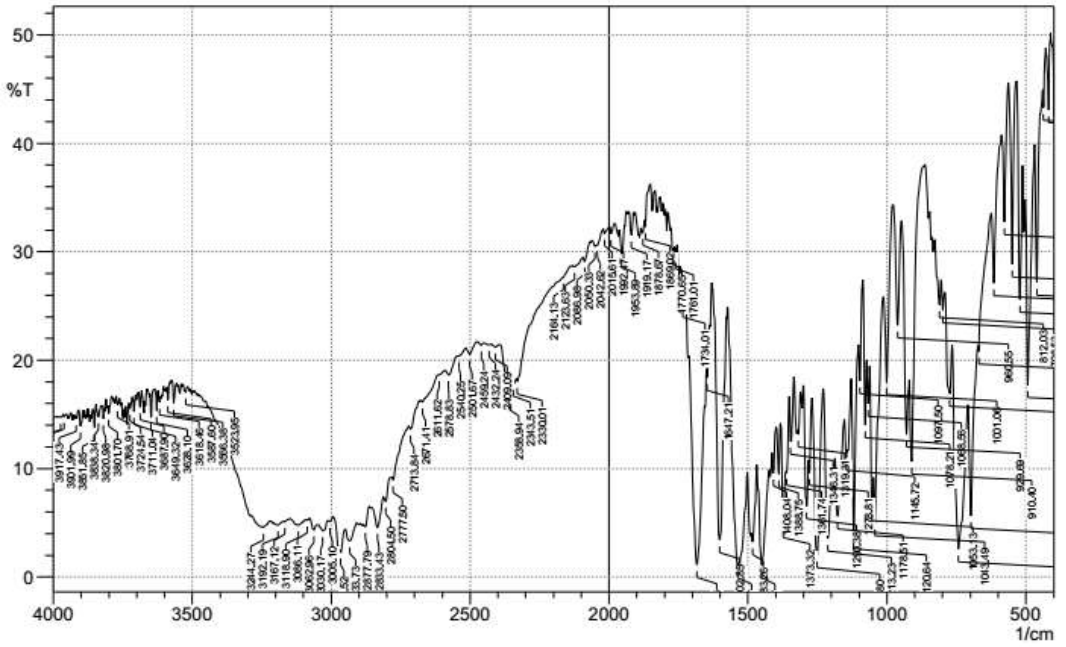

The IR spectra of compound **10e**

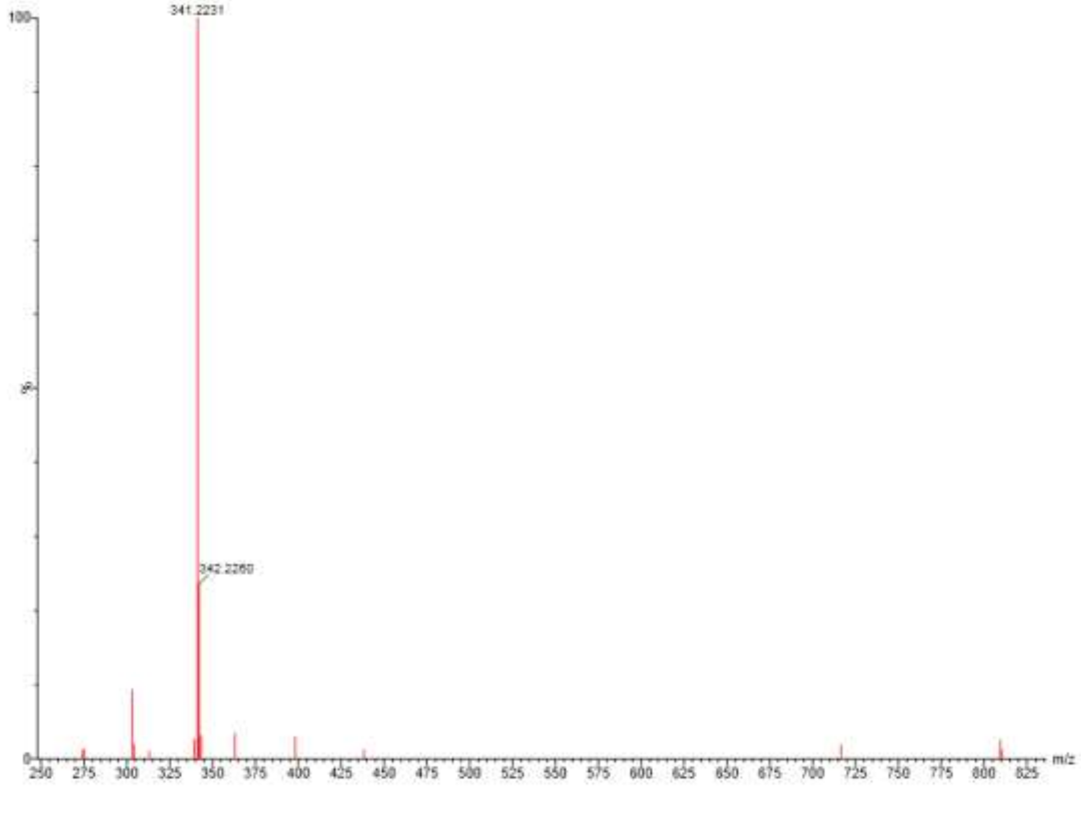

The HRMS of compound **10e**

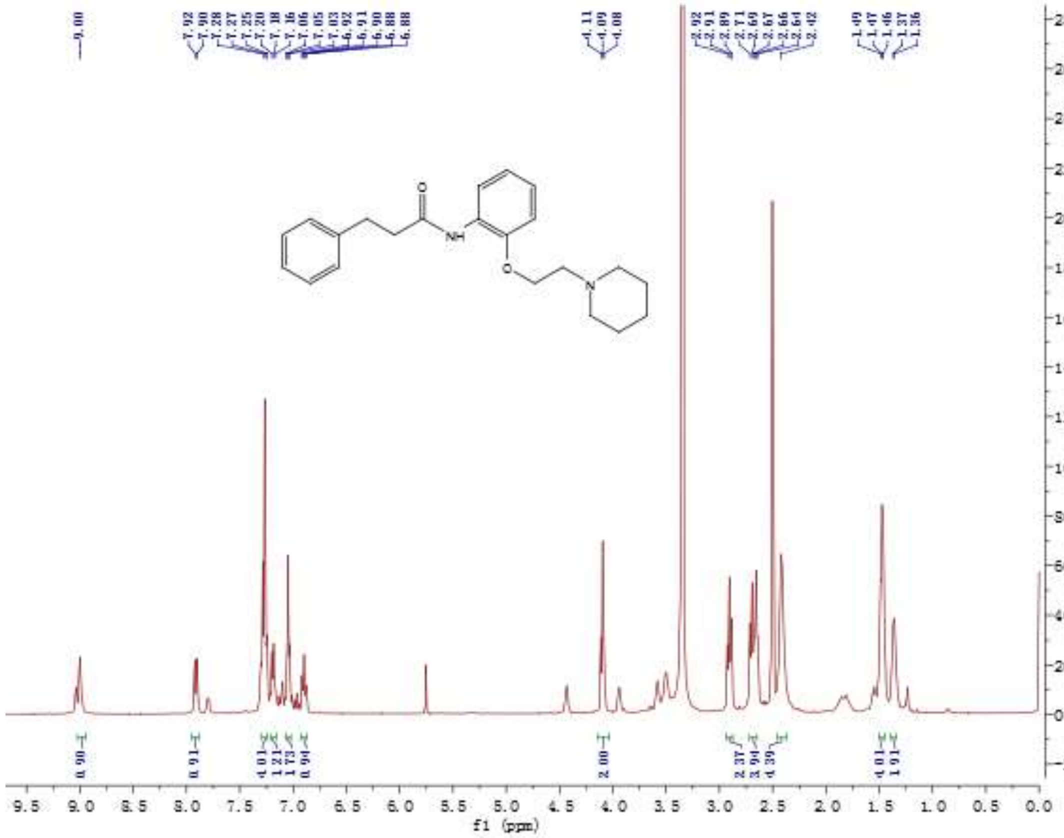

The ^{1}H-NMR (400MHz, DMSO-d_6) of compound **10f**

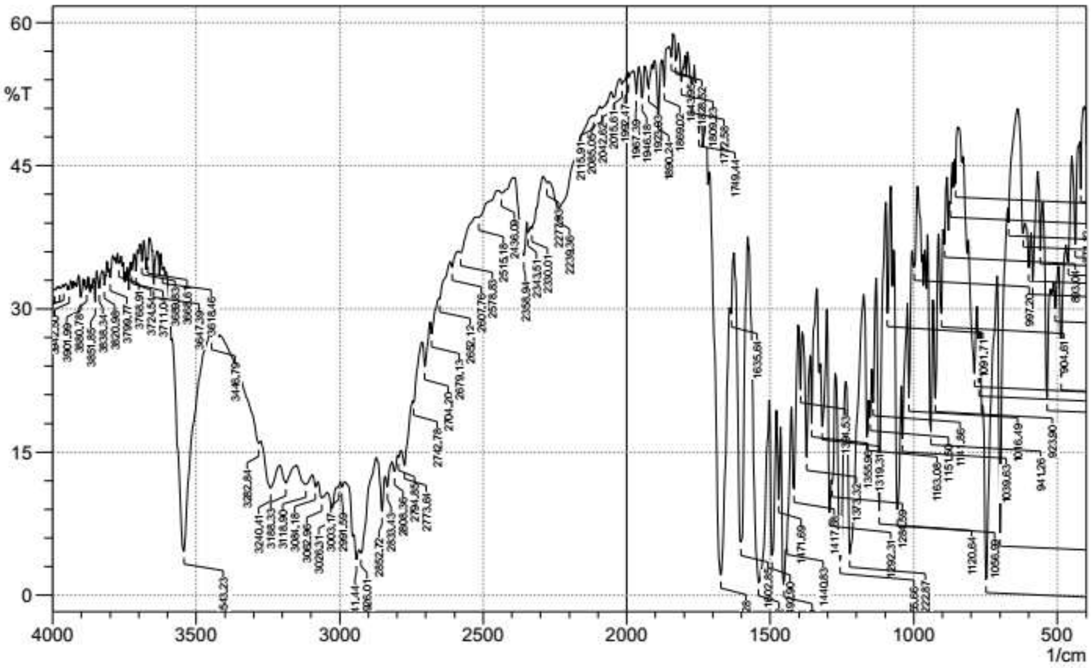

The IR spectra of compound **10f**

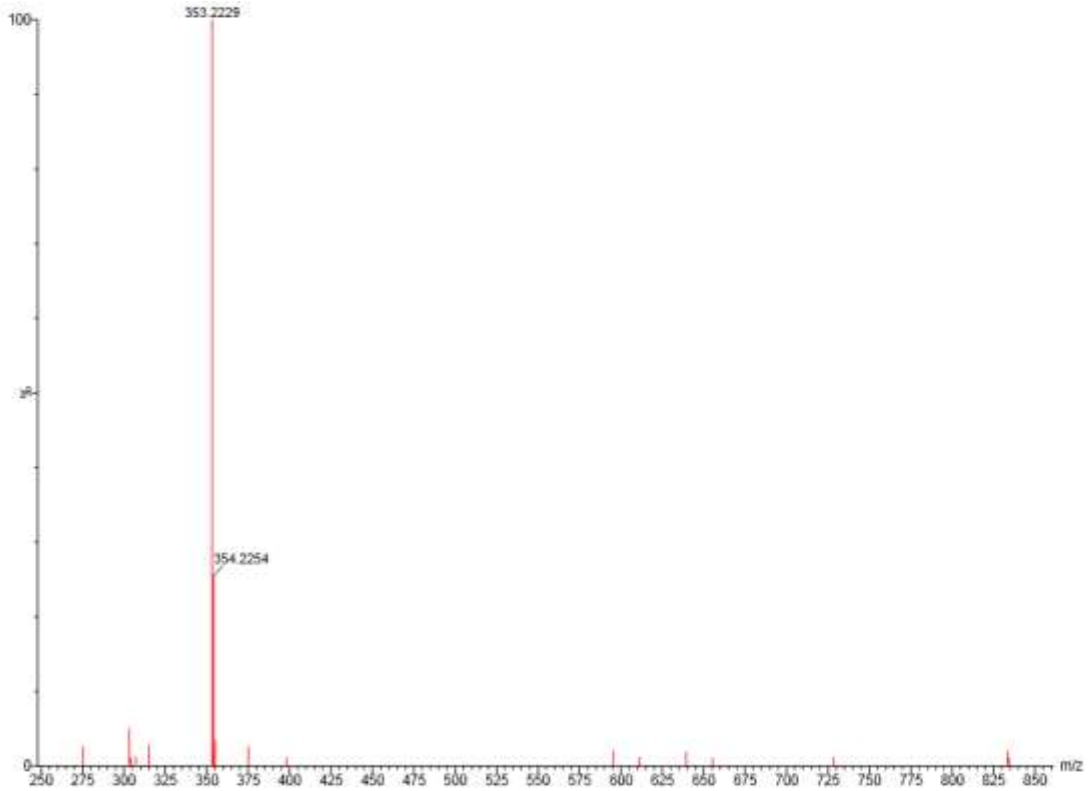

The HRMS of compound **10f**

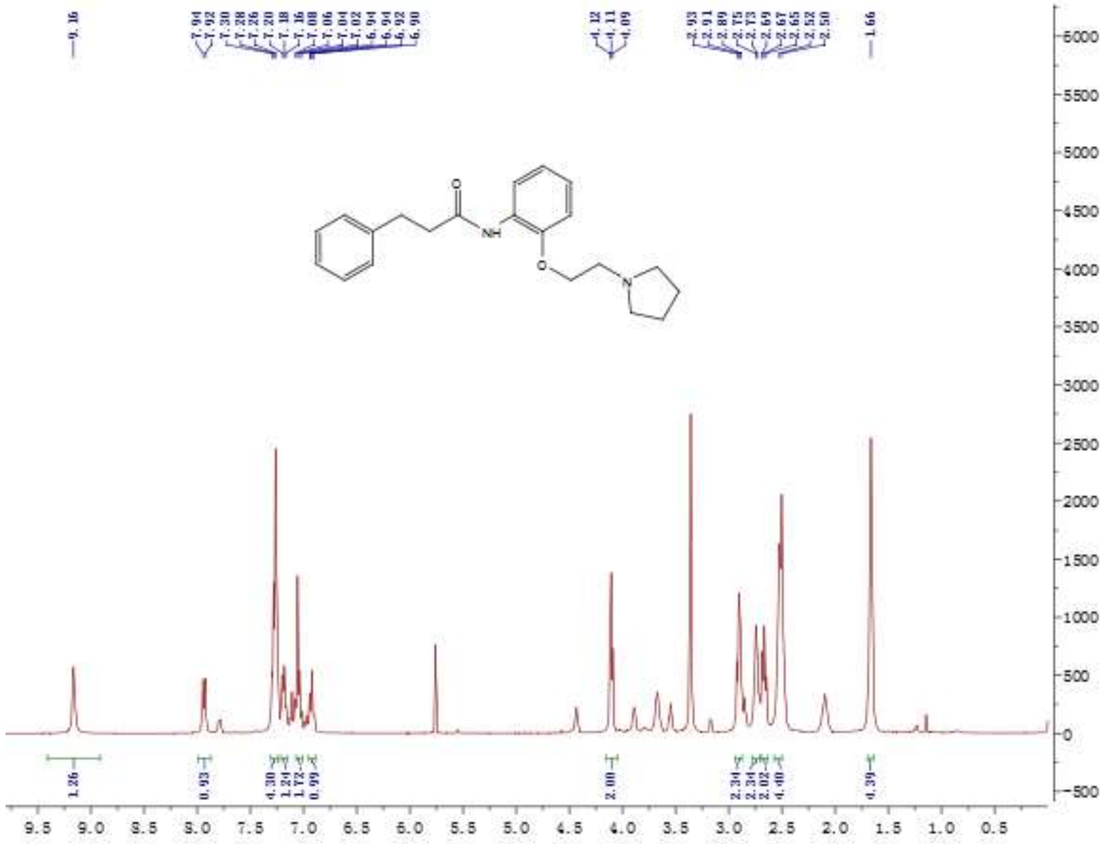

The ^{1}H-NMR (400MHz, DMSO-d_6) of compound **10g**

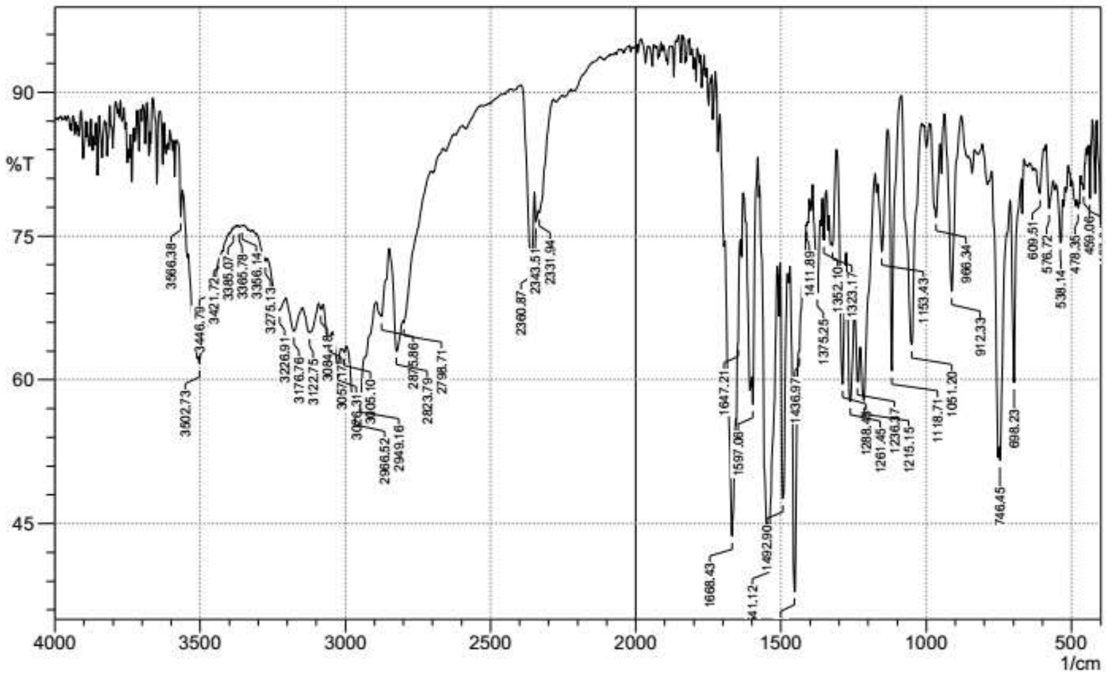

The IR spectra of compound **10g**

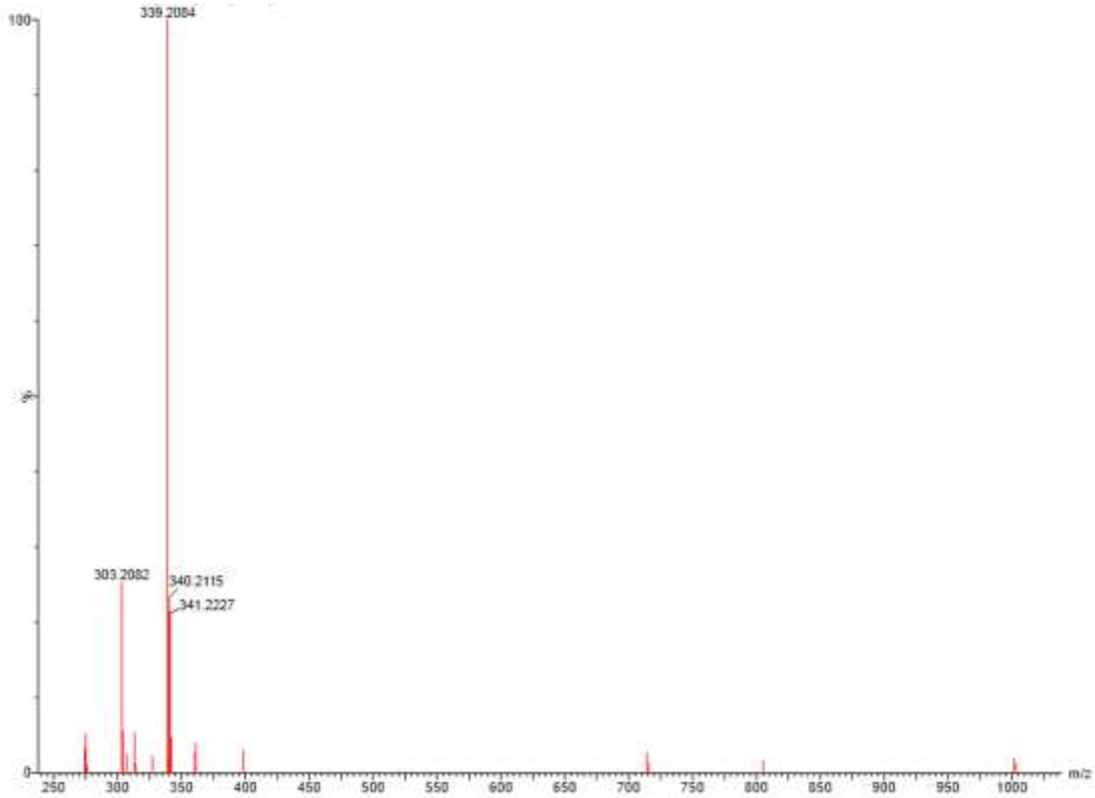

The HRMS of compound **10g**

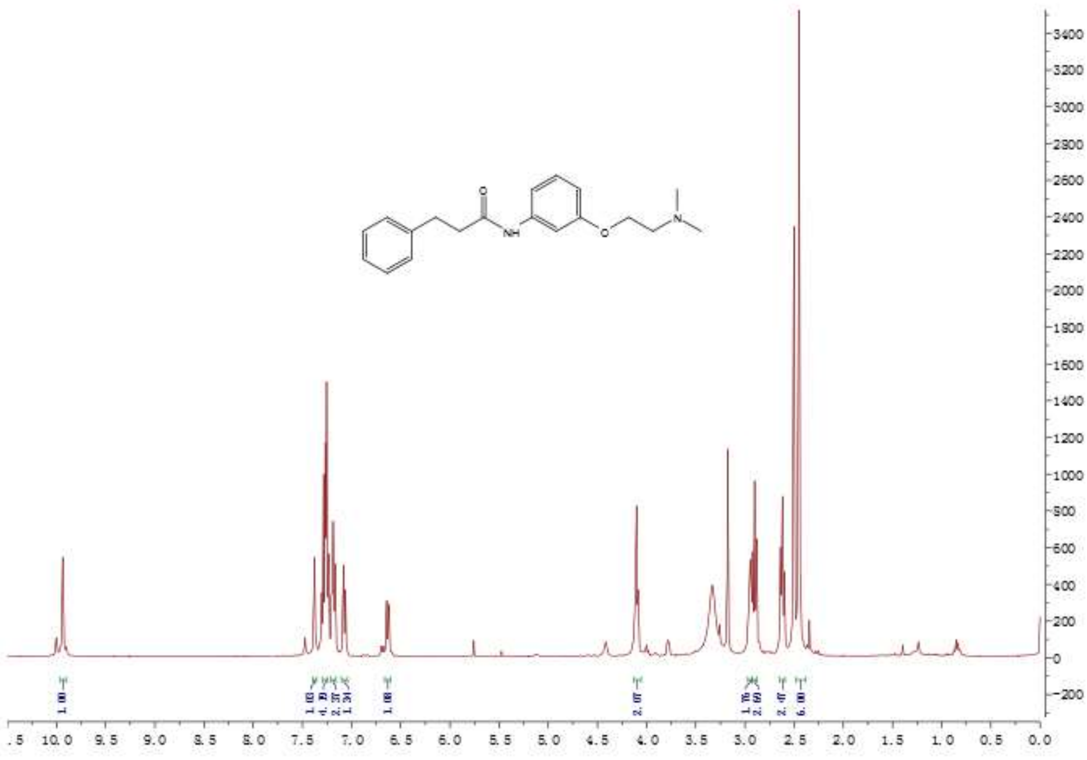

The ^{1}H-NMR (400MHz, DMSO-d_6) of compound **11d**

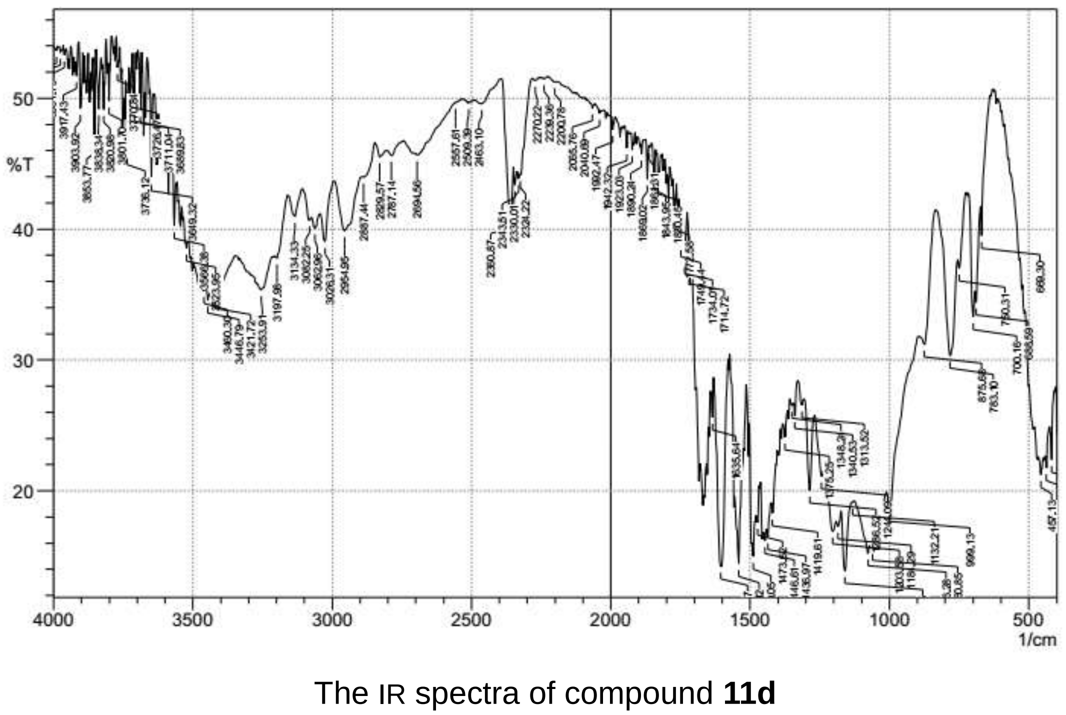

The IR spectra of compound **11d**

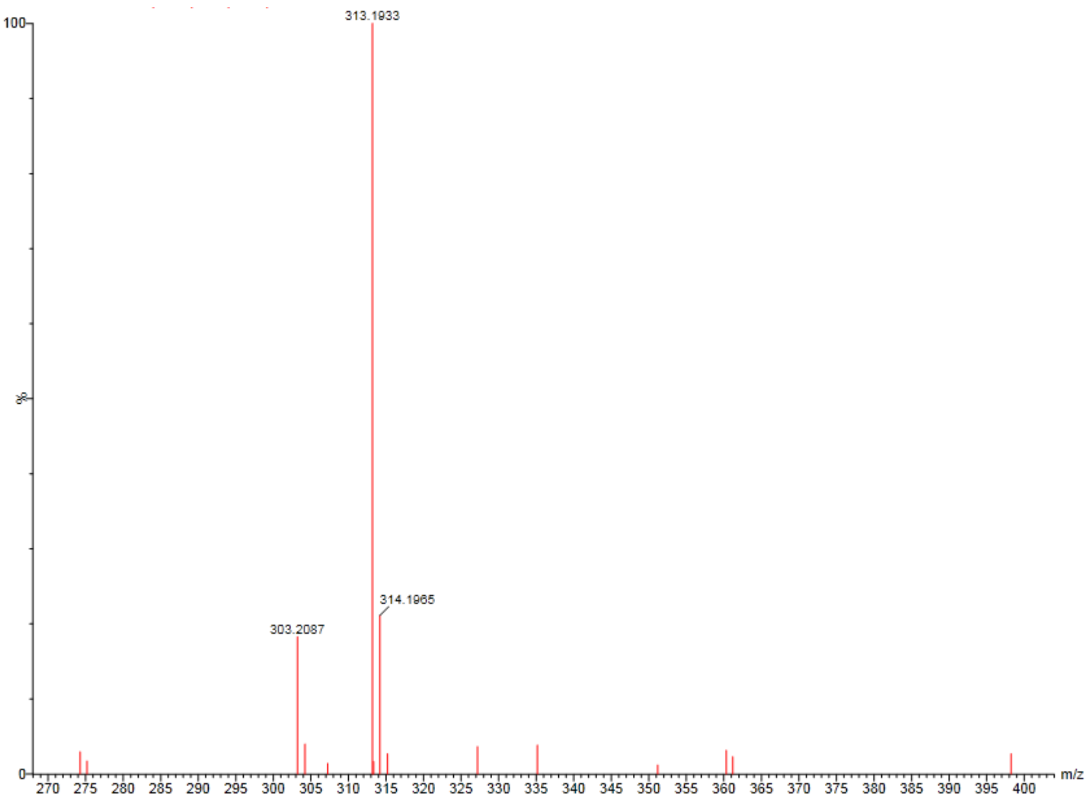

The HRMS of compound **11d**

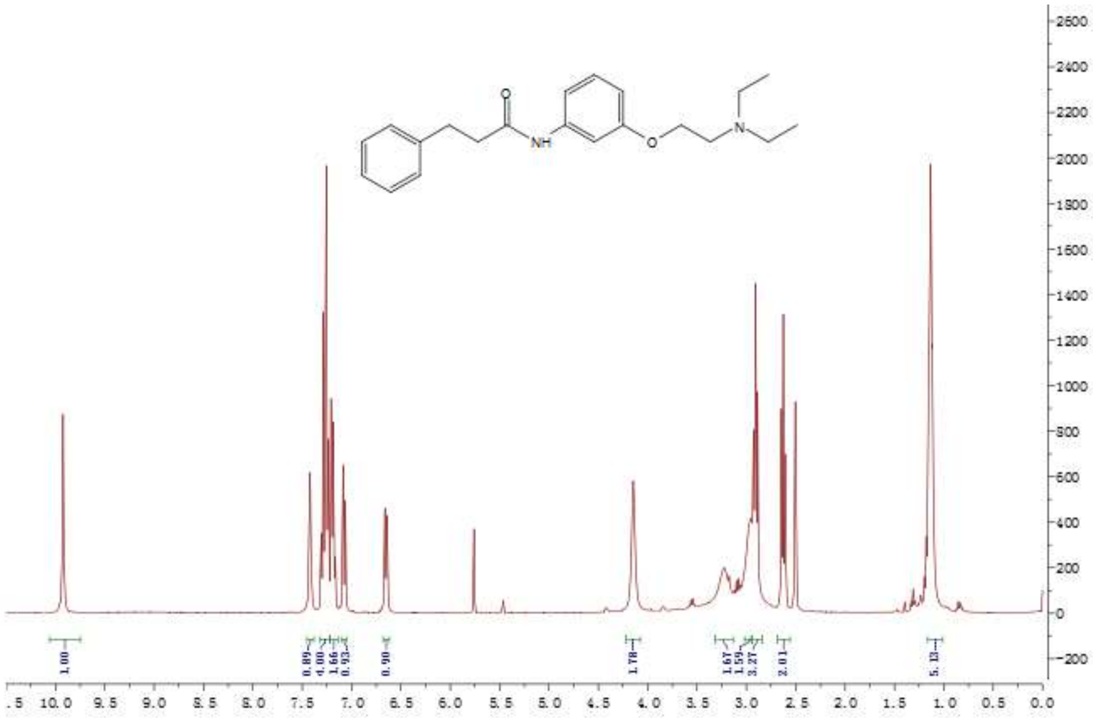

The ^{1}H-NMR (400MHz, DMSO-d_6) of compound **11e**

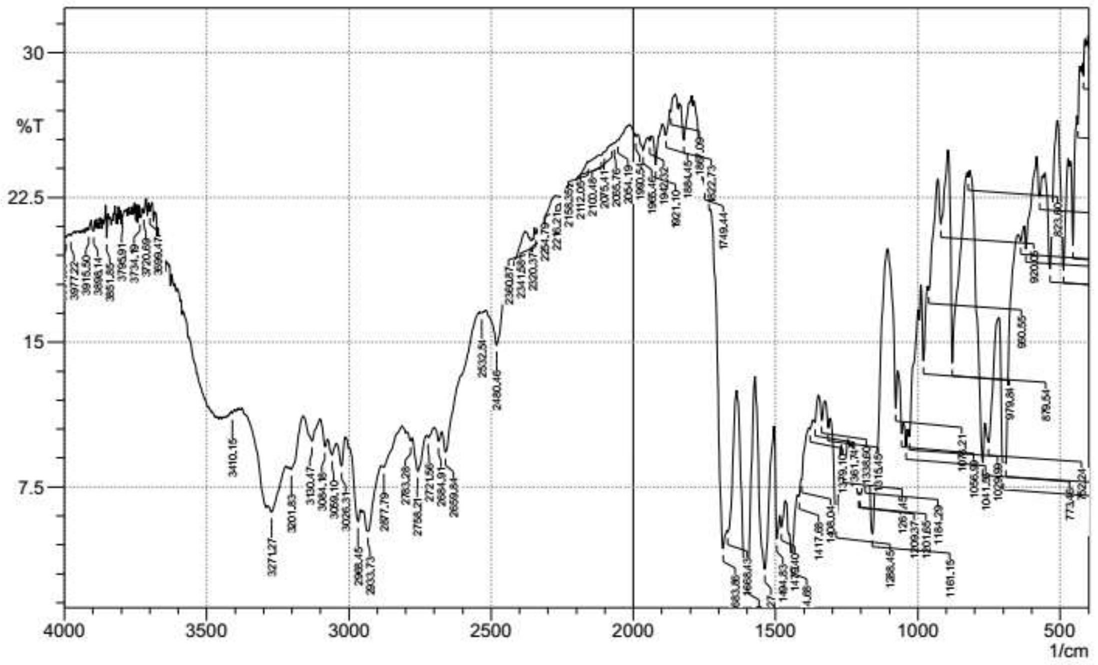

The IR spectra of compound **11e**

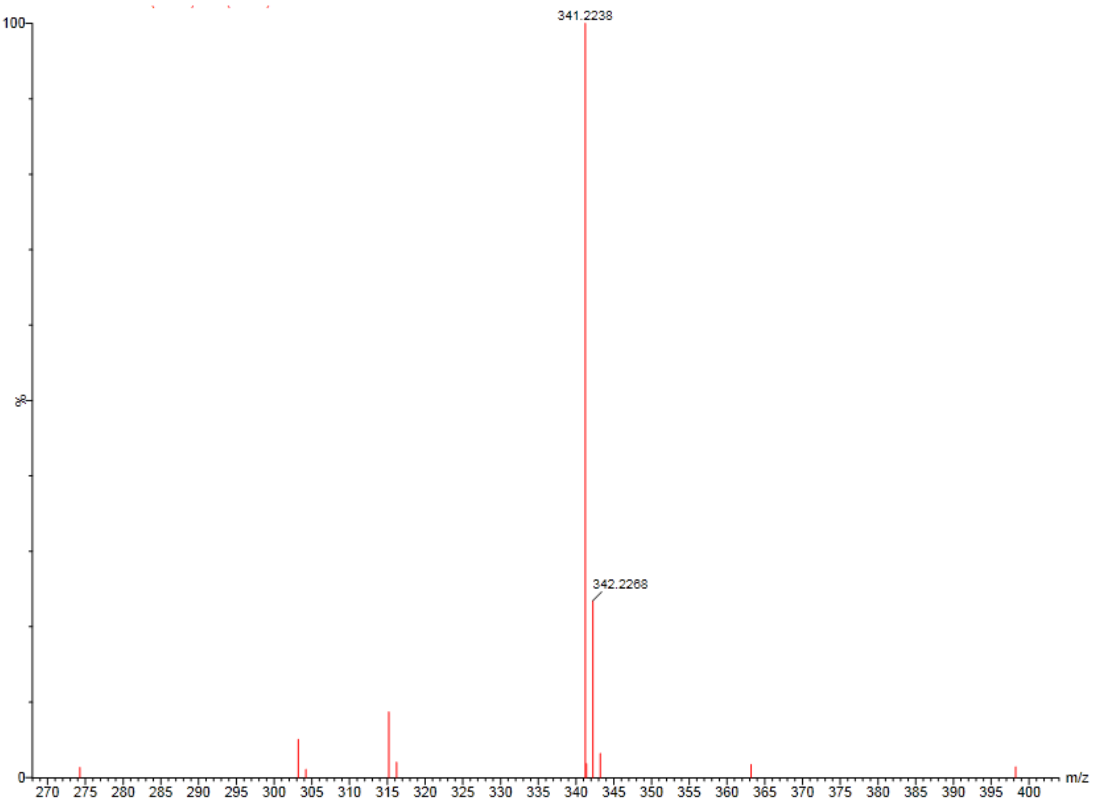

The HRMS of compound **11e**

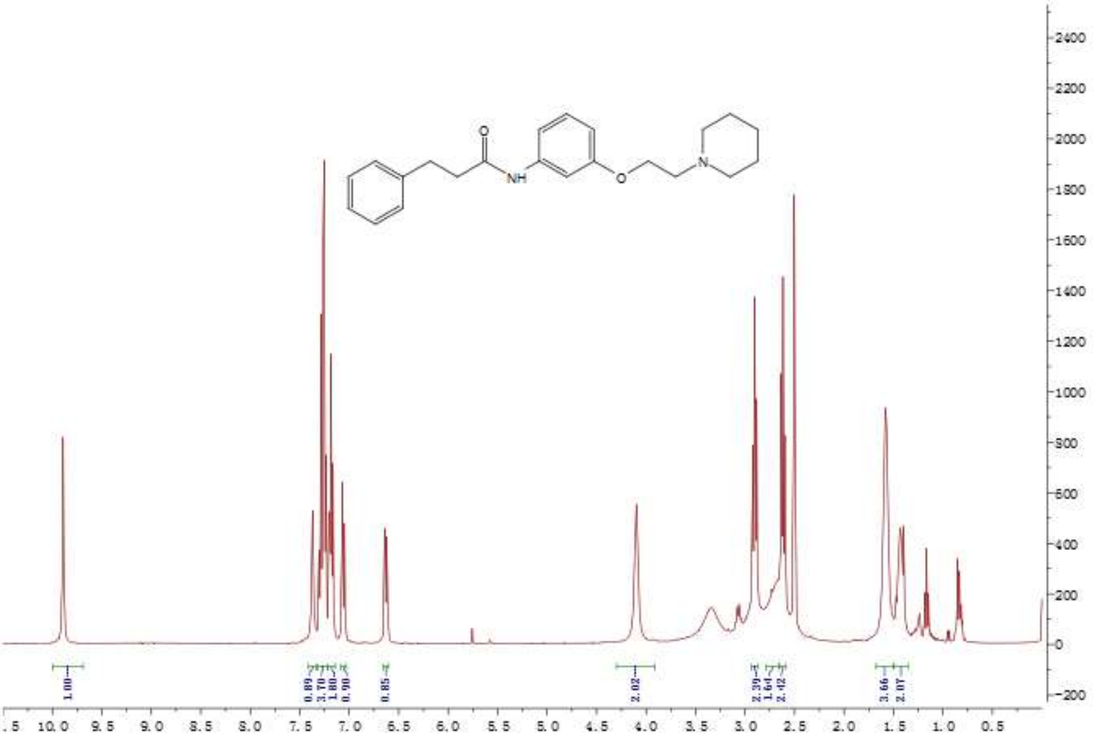

The ^{1}H-NMR (400MHz, DMSO-d_6) of compound **11f**

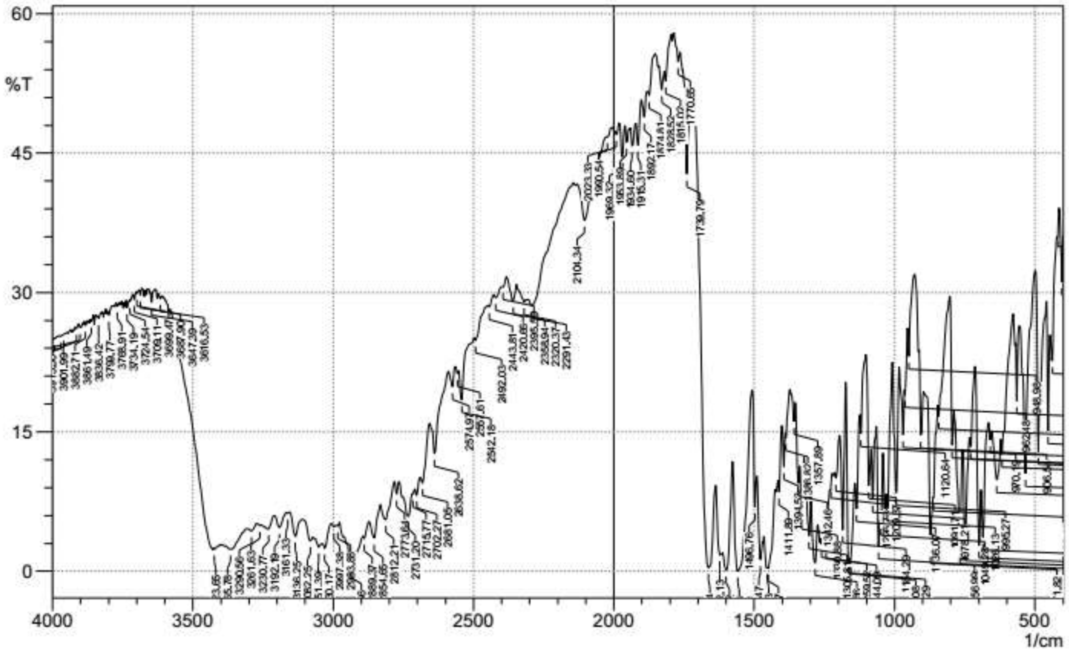

The IR spectra of compound **11f**

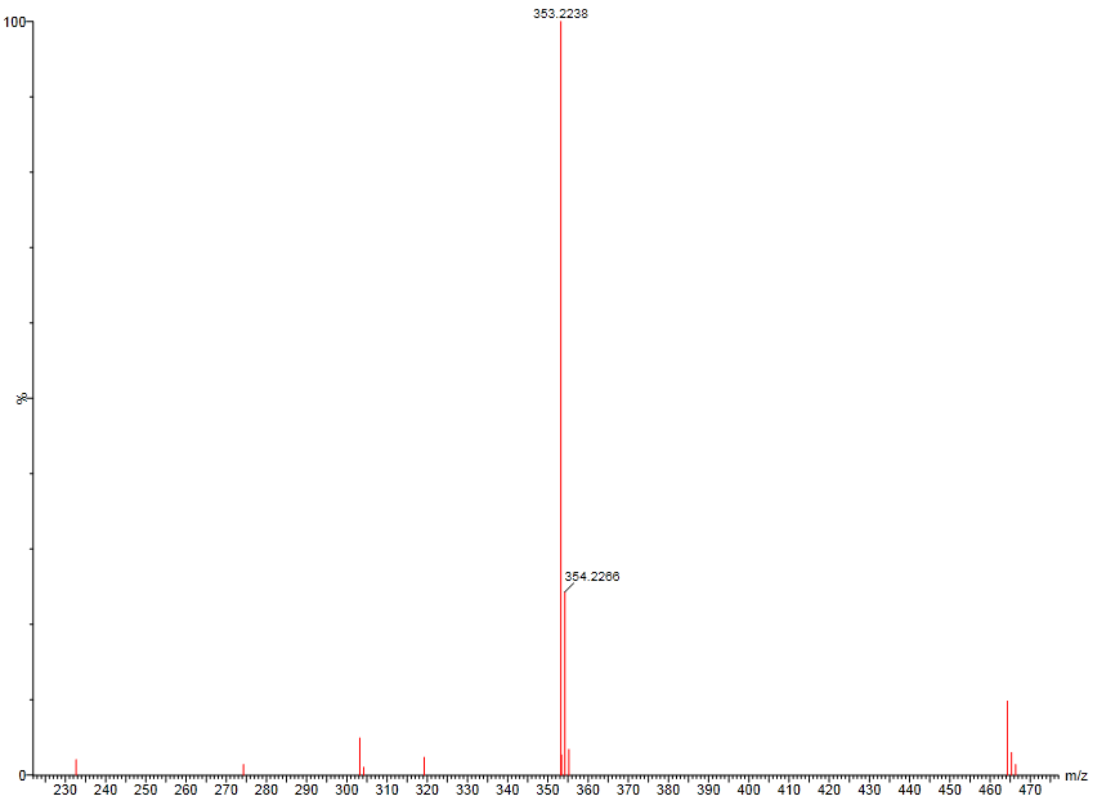

The HRMS of compound **11f**

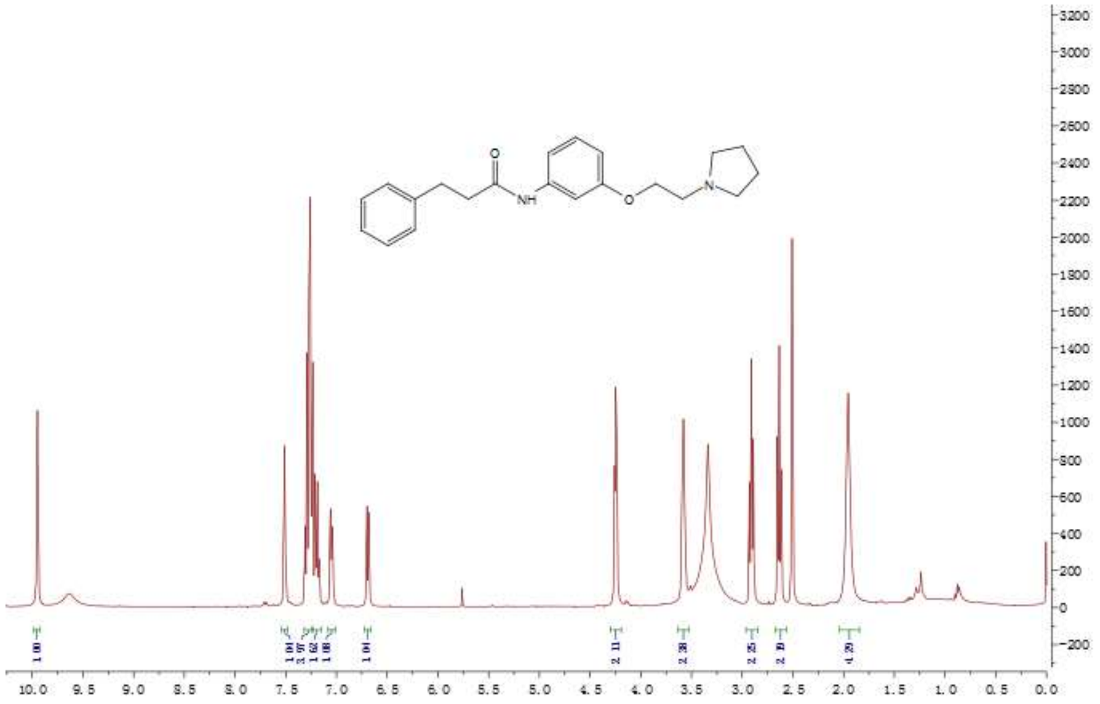

The ^{1}H-NMR (400MHz, DMSO-d_6) of compound **11g**

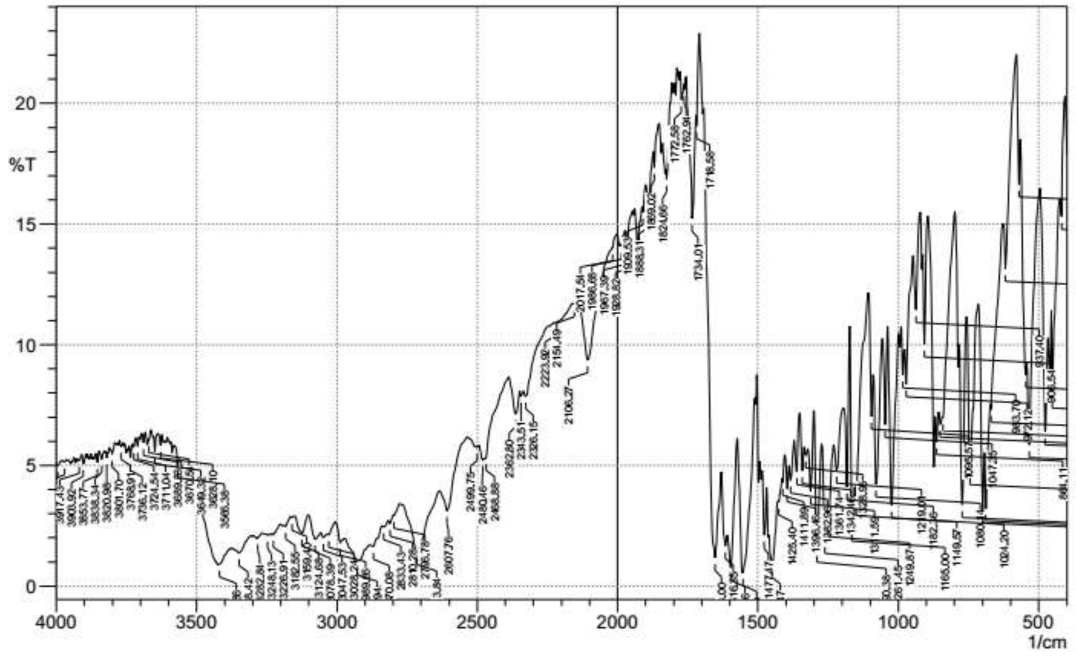

The IR spectra of compound **11g**

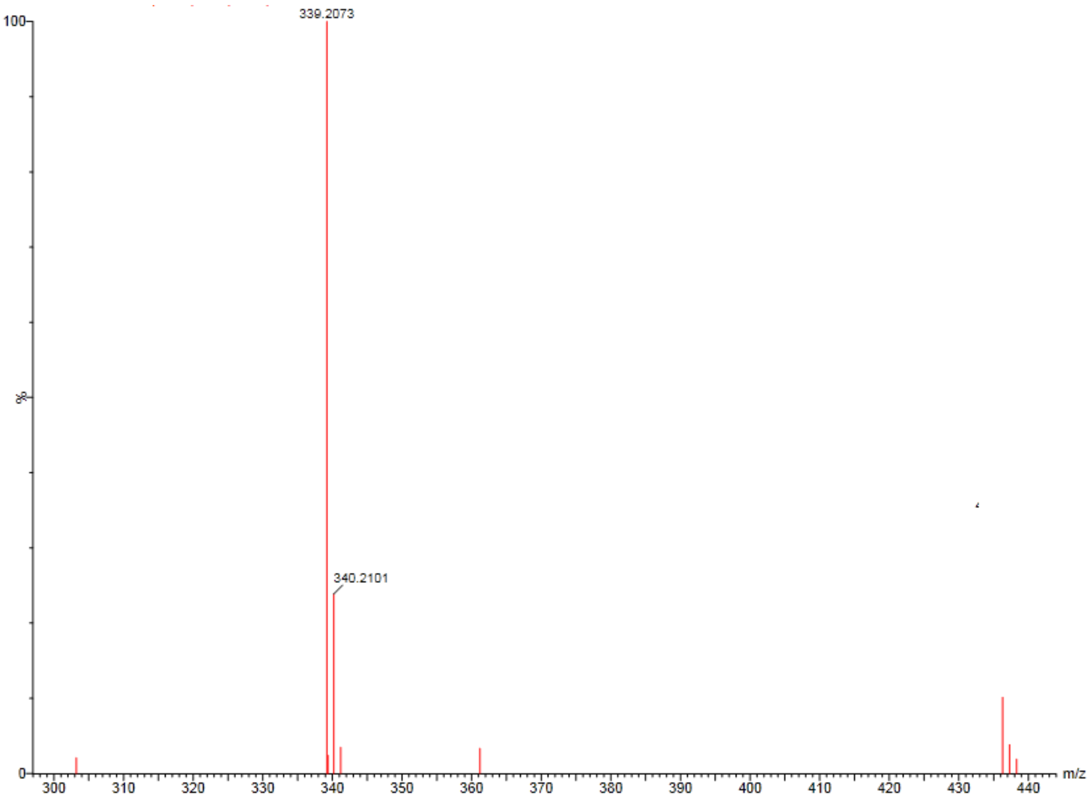

The HRMS of compound **11g**

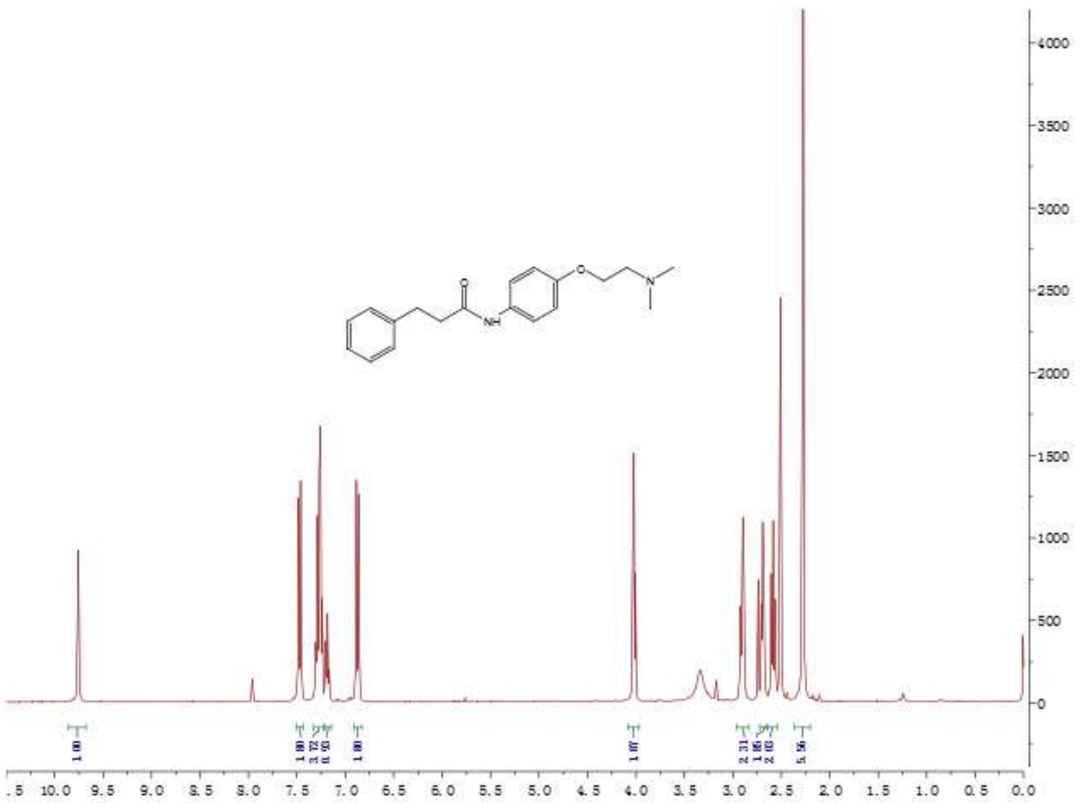

The ^{1}H-NMR (400MHz, DMSO-d_6) of compound **12d**

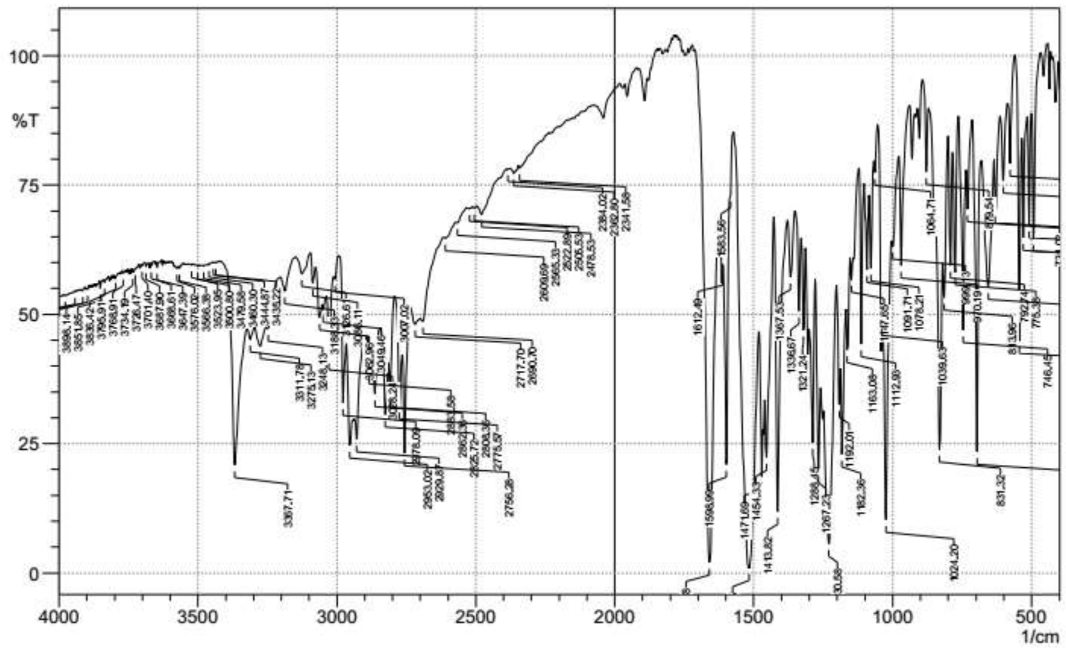

The IR spectra of compound **12d**

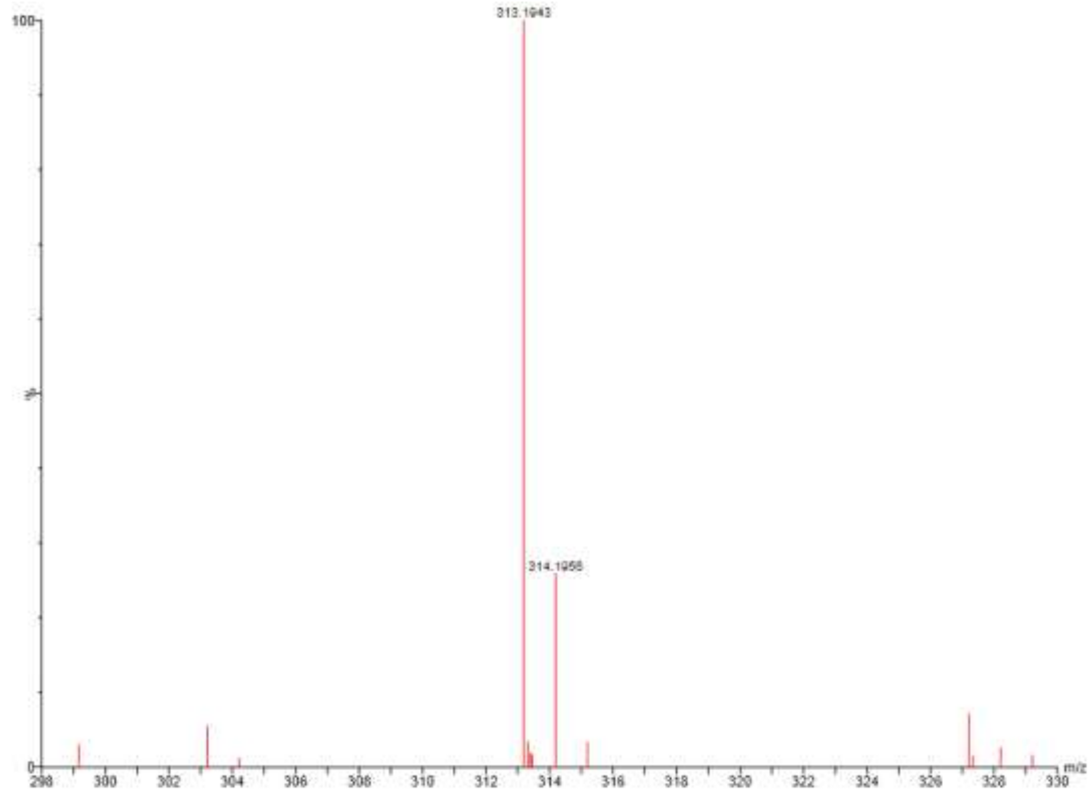

The HRMS of compound **12d**

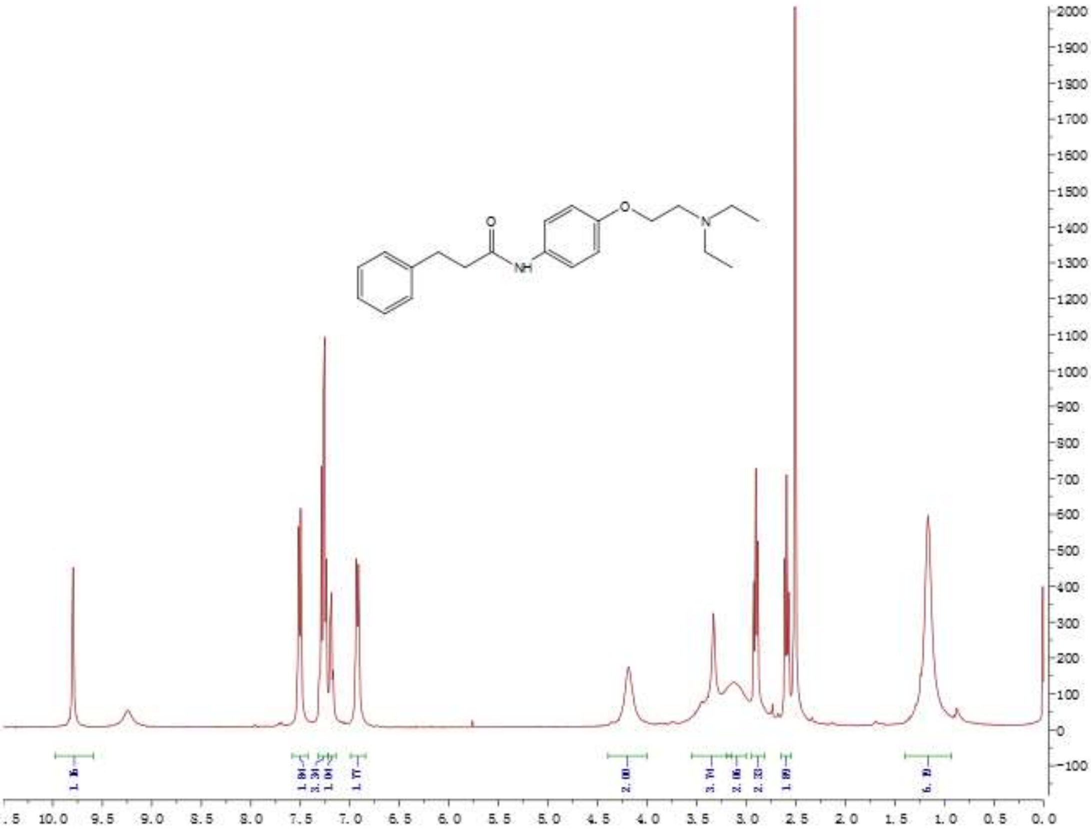

The ^{1}H-NMR (400MHz, DMSO-d_6) of compound **12e**

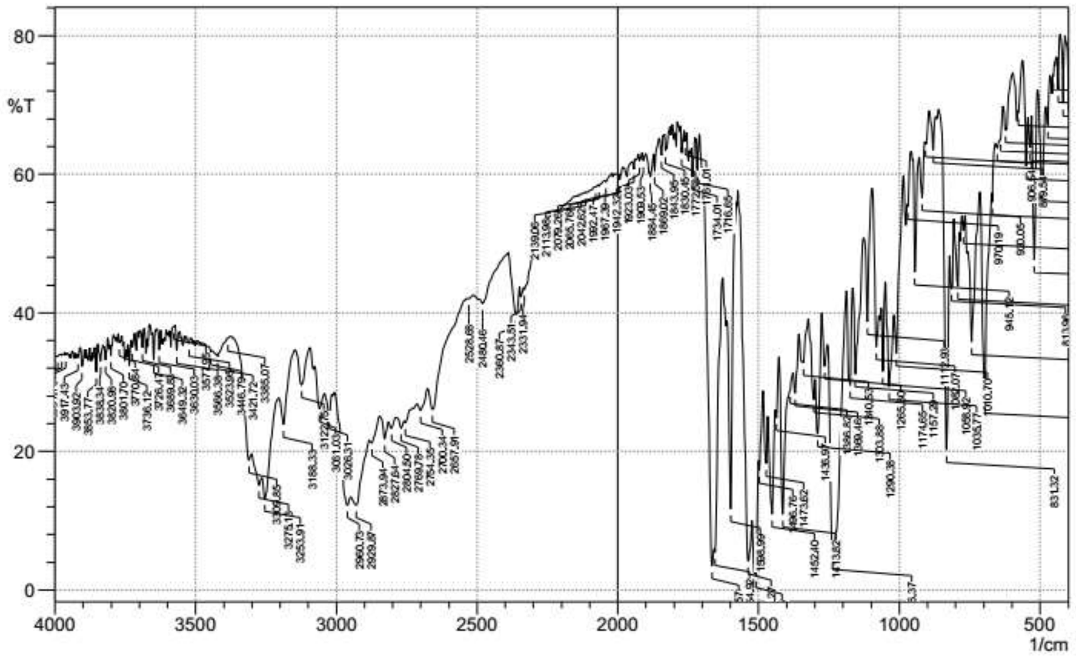

The IR spectra of compound **12e**

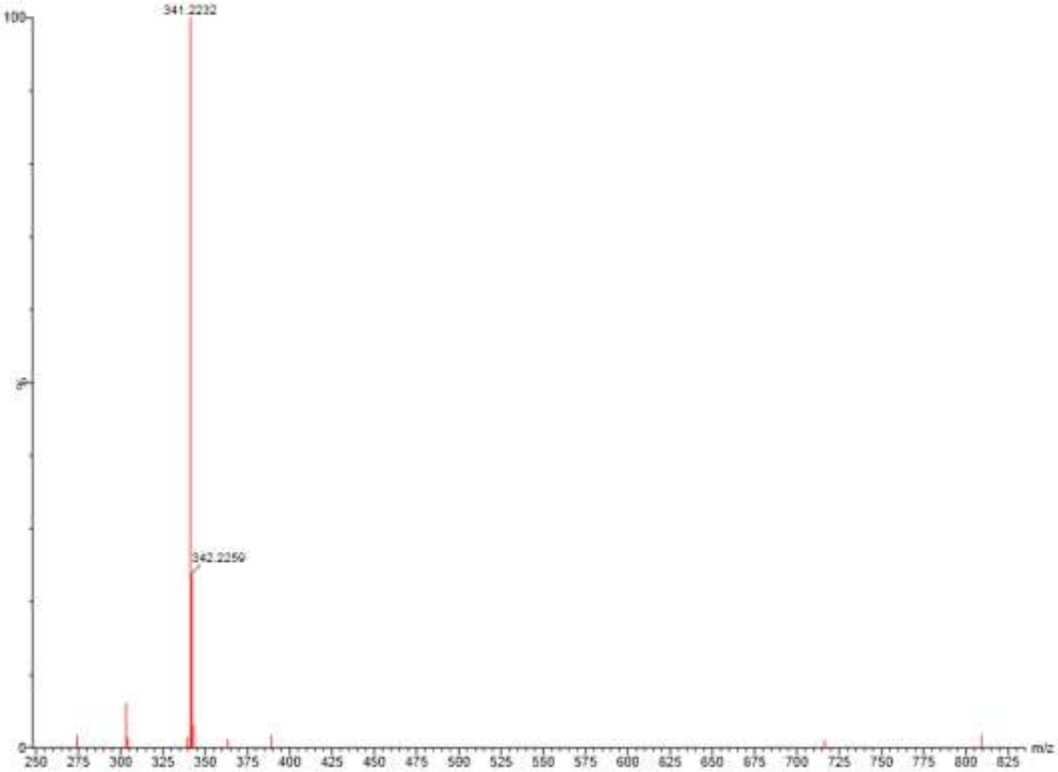

The HRMS of compound **12e**

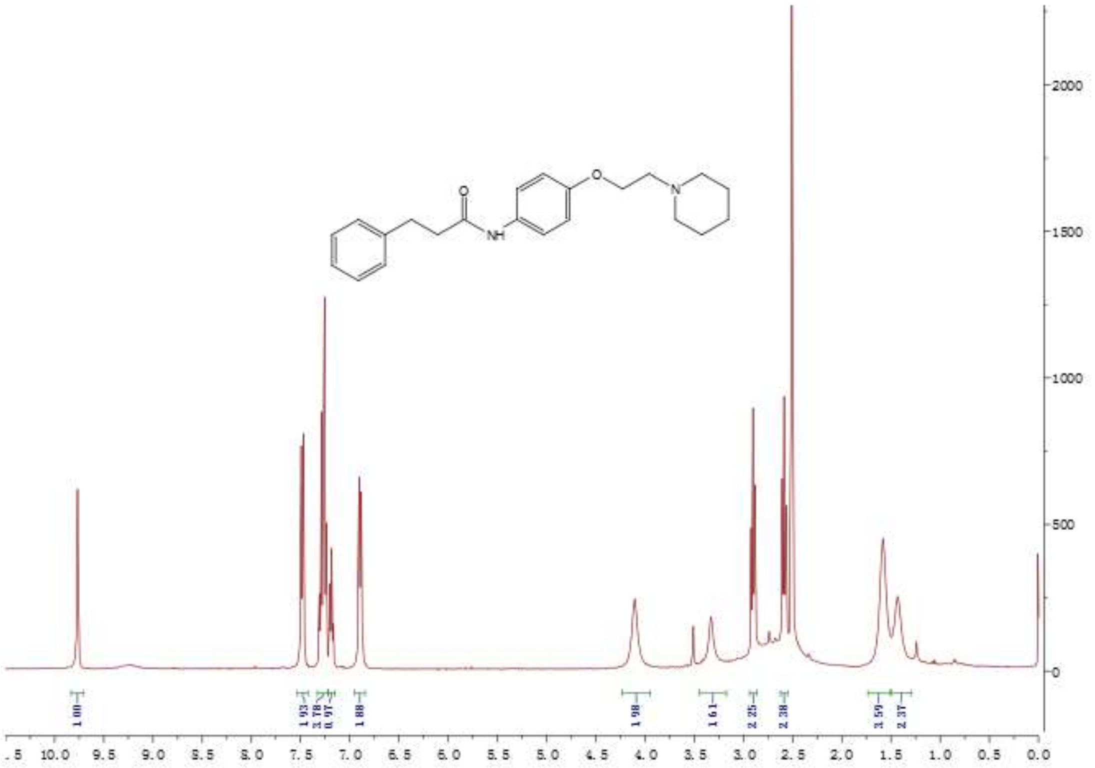

The ^{1}H-NMR (400MHz, DMSO-d_6) of compound **12f**

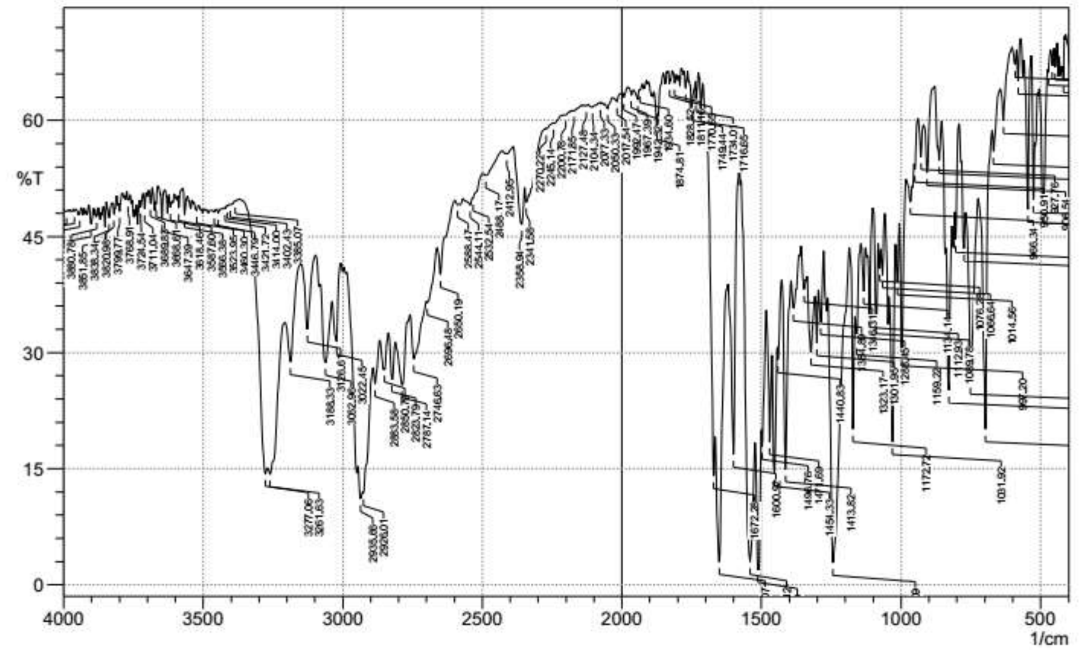

The IR spectra of compound **12f**

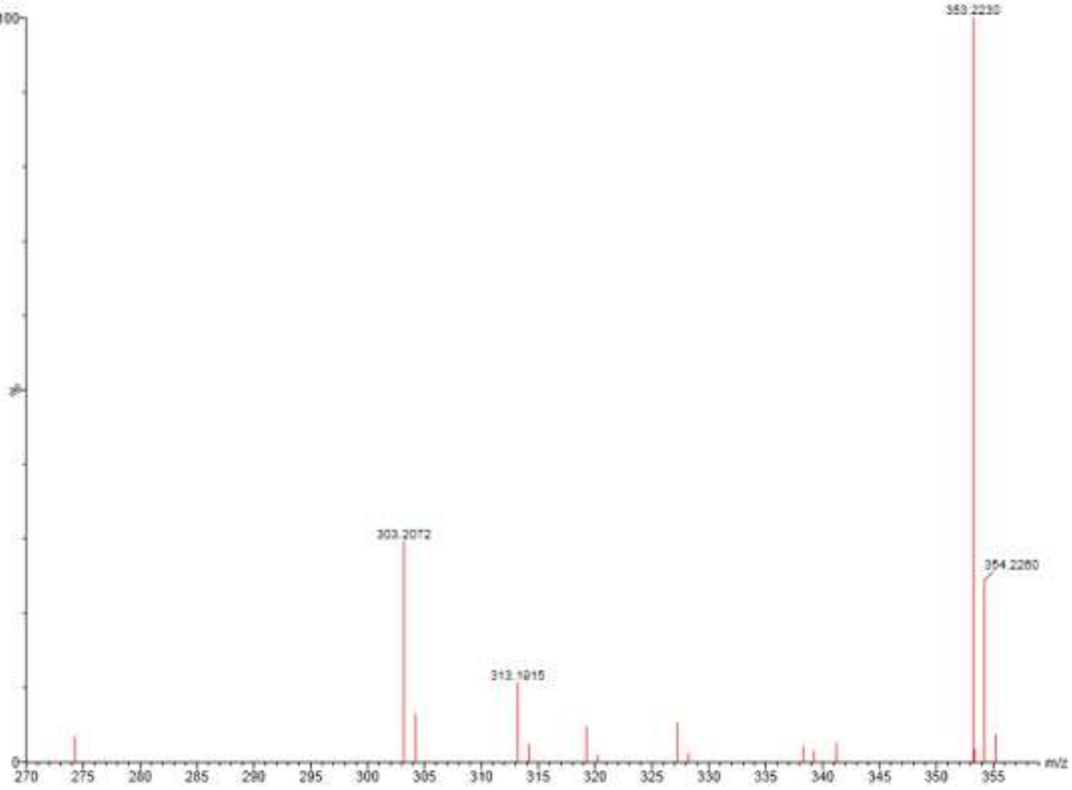

The HRMS of compound **12f**

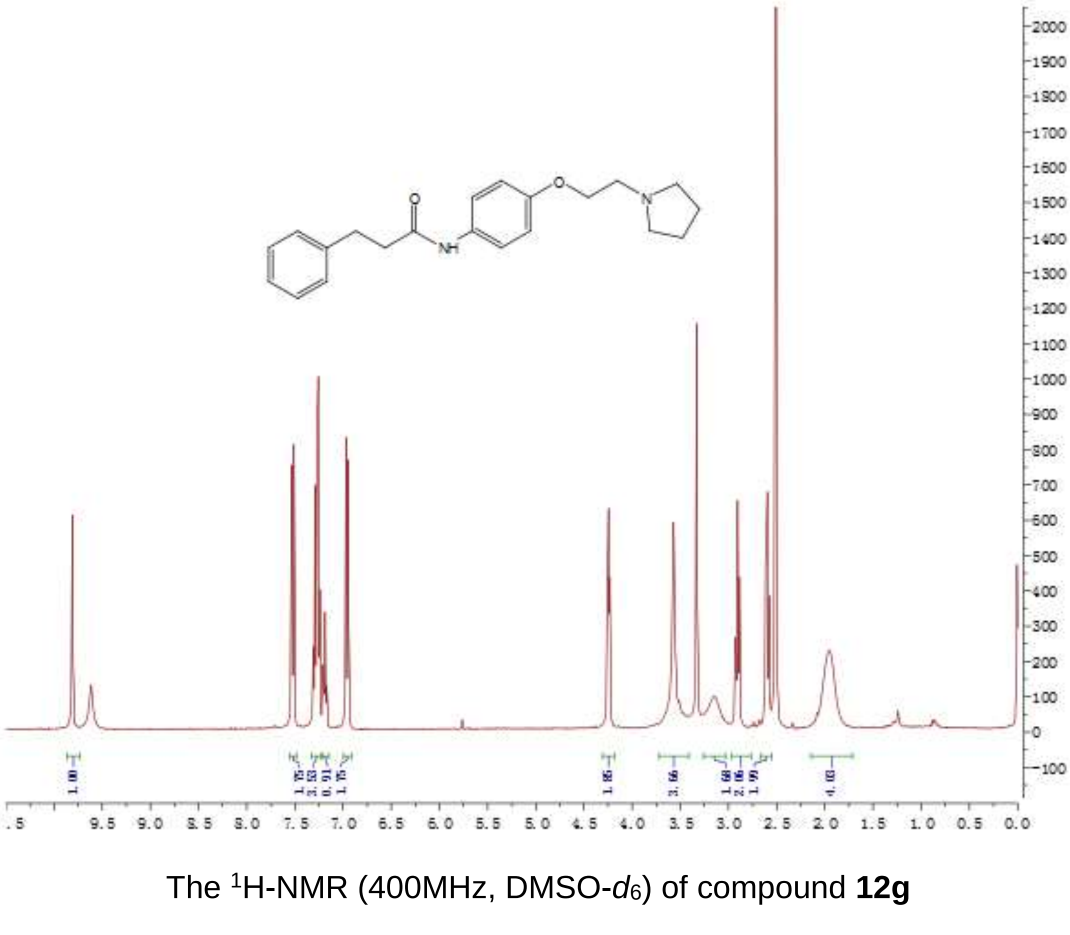

The ^{1}H-NMR (400MHz, DMSO-d_6) of compound **12g**

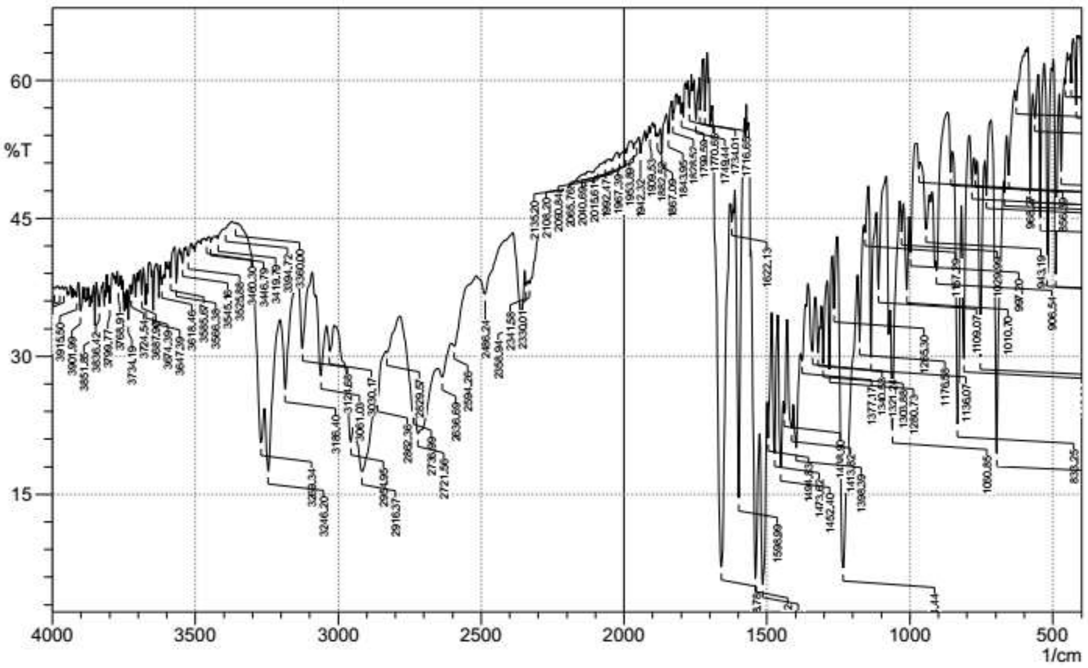

The IR spectra of compound **12g**

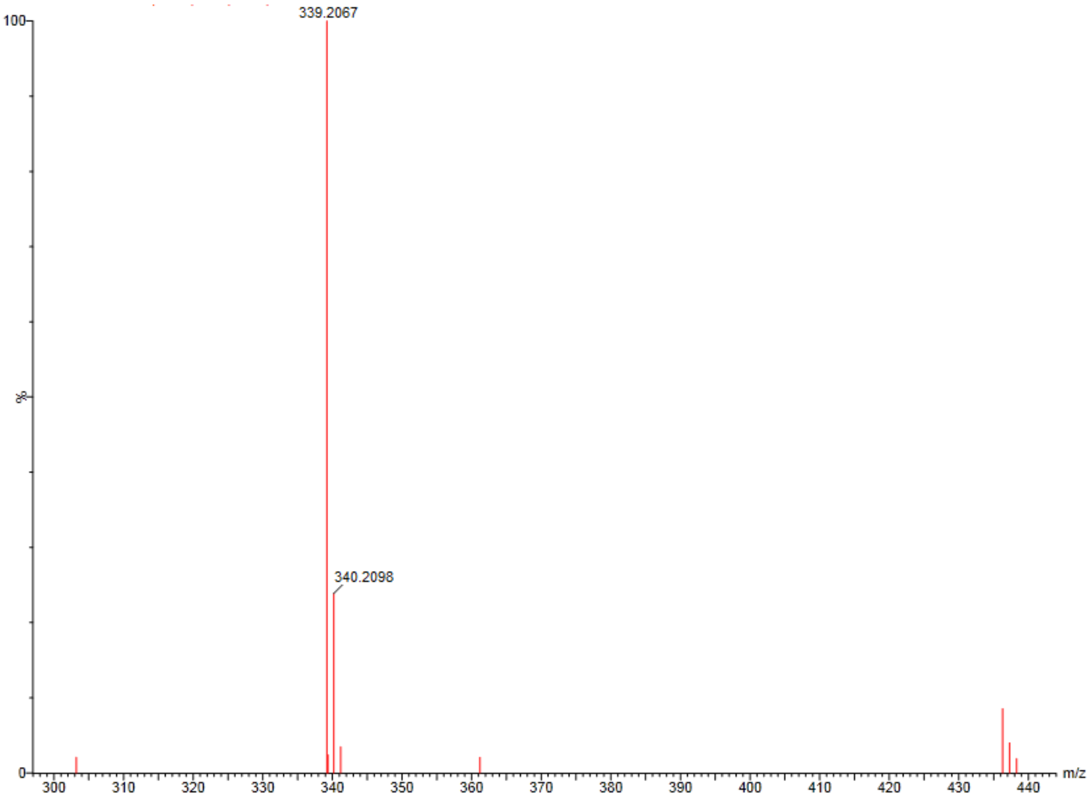

The HRMS of compound **12g**

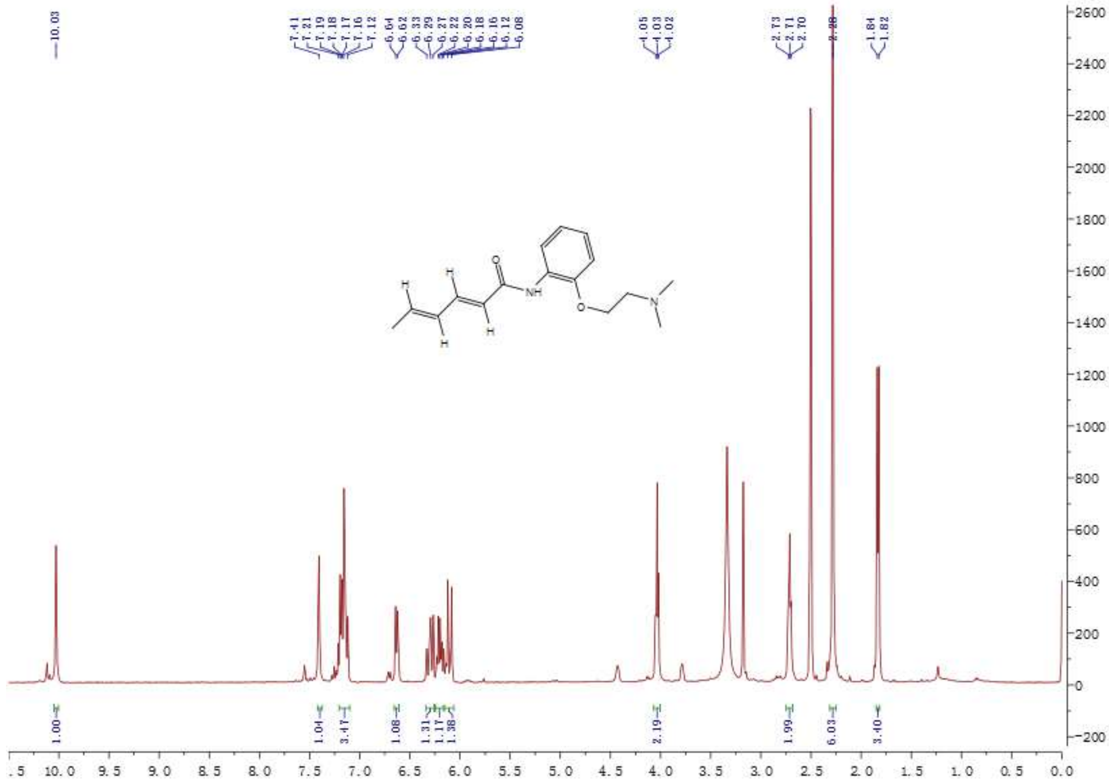

The ^{1}H-NMR (400MHz, DMSO-*d*6) of compound **16d**

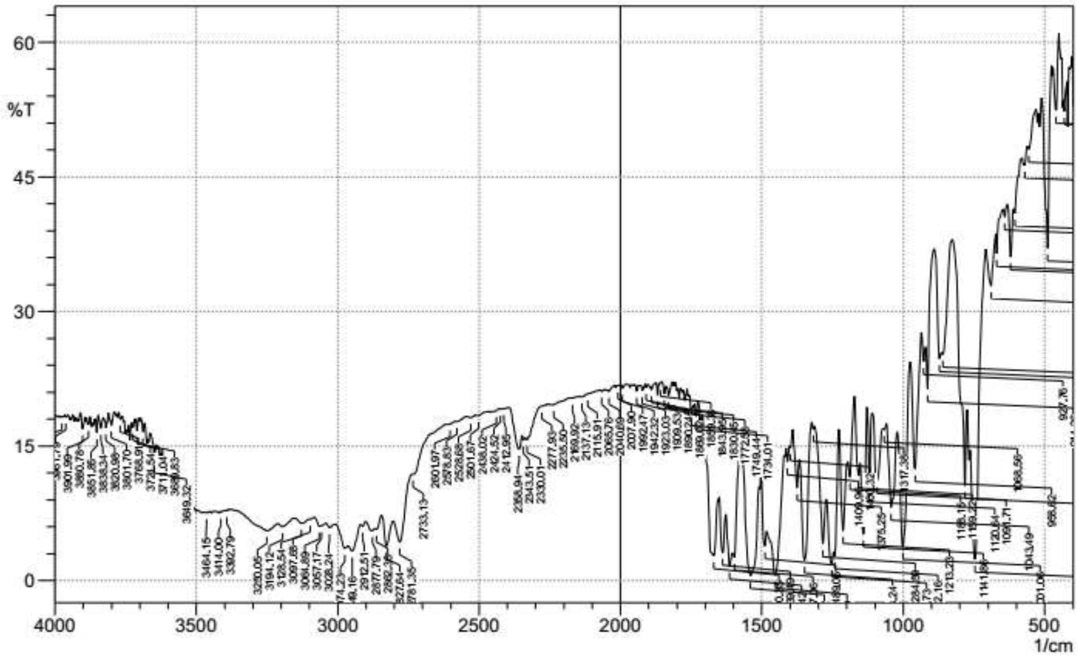

The IR spectra of compound **16d**

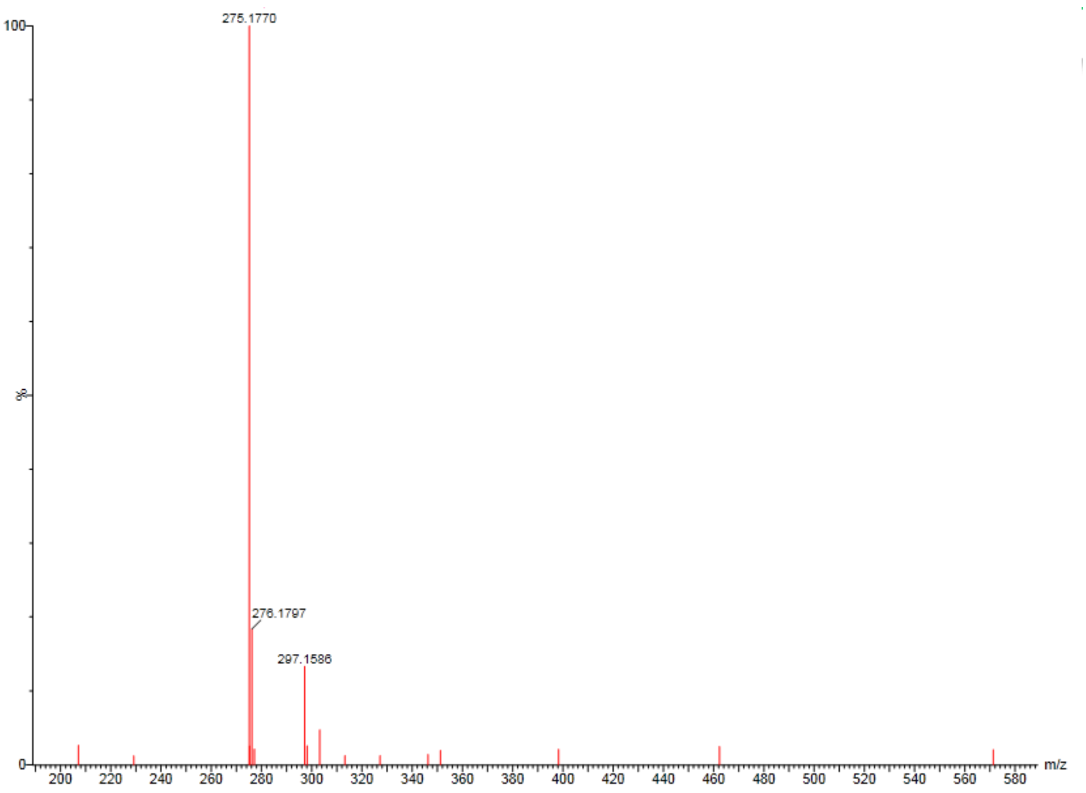

The HRMS of compound **16d**

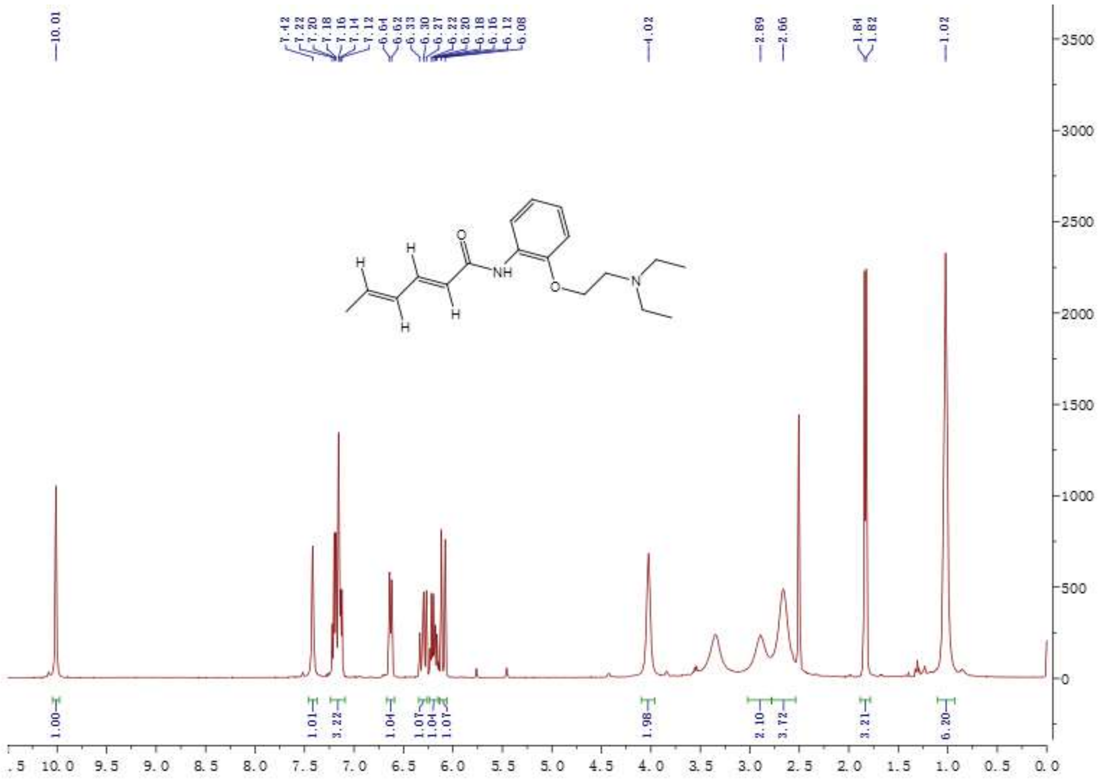

The ^{1}H-NMR (400MHz, DMSO-d_6) of compound **16e**

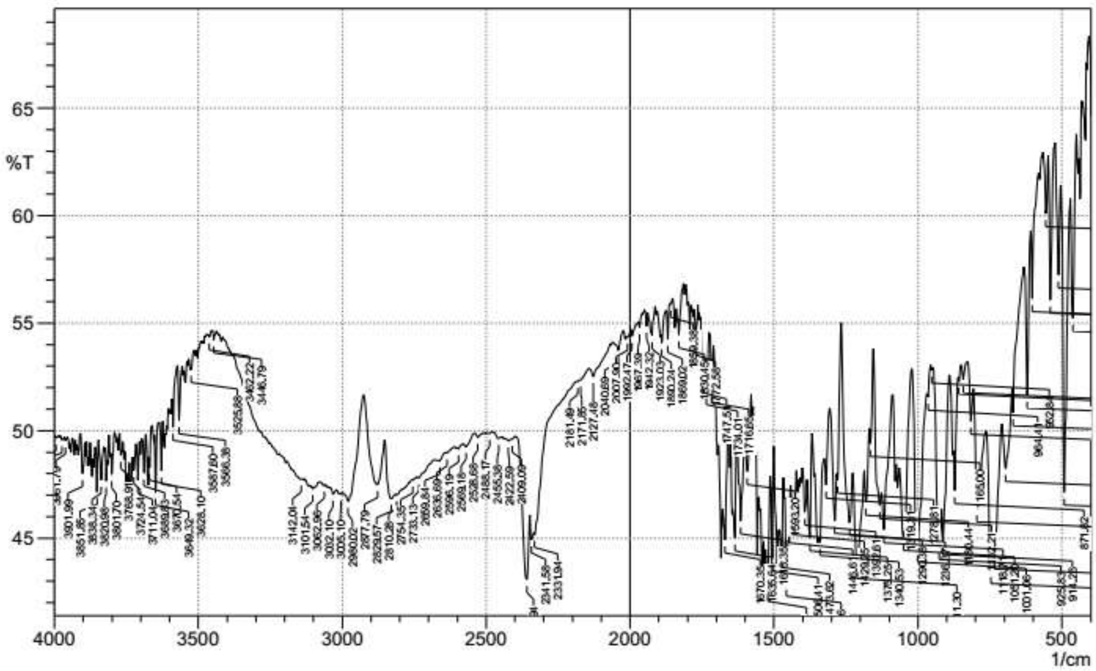

The IR spectra of compound **16e**

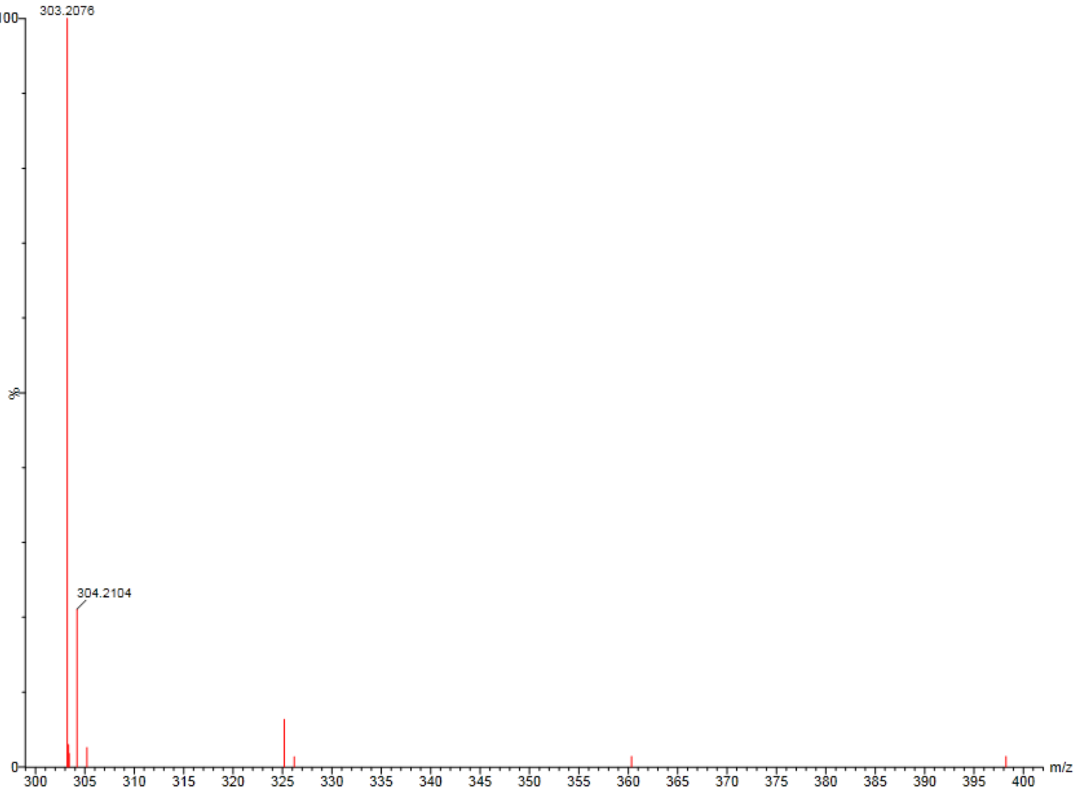

The HRMS of compound **16e**

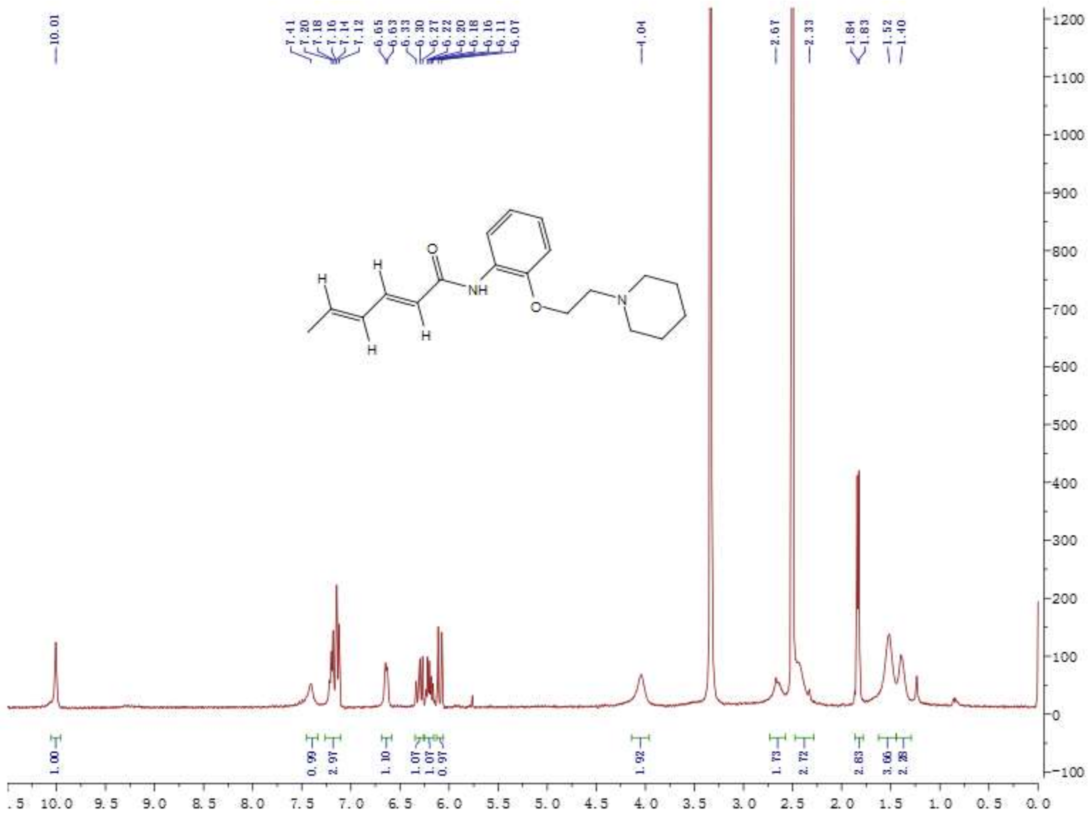

The ^{1}H-NMR (400MHz, DMSO-d_6) of compound **16f**

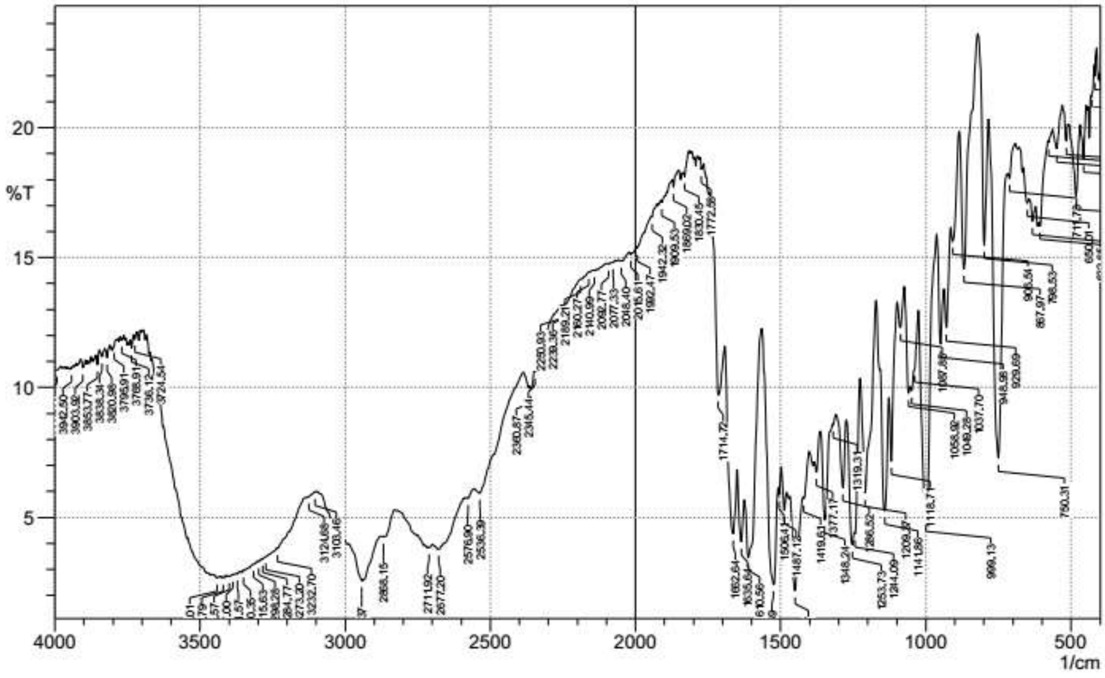

The IR spectra of compound **16f**

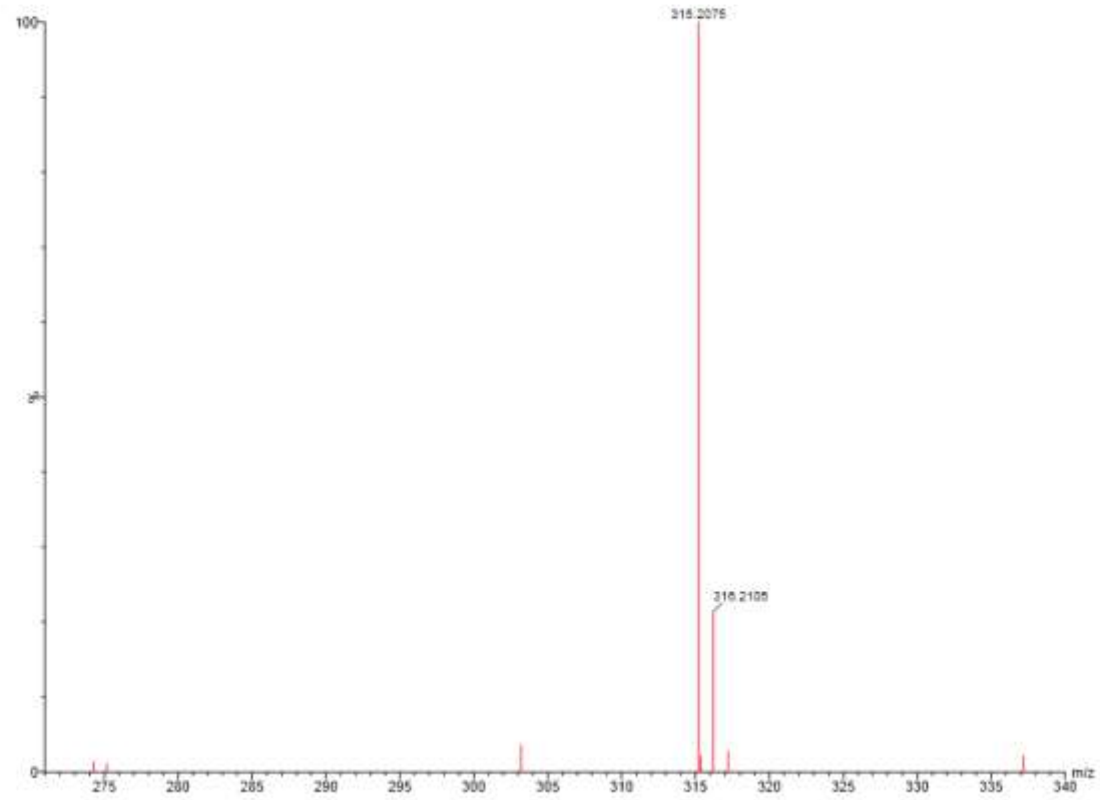

The HRMS of compound **16f**

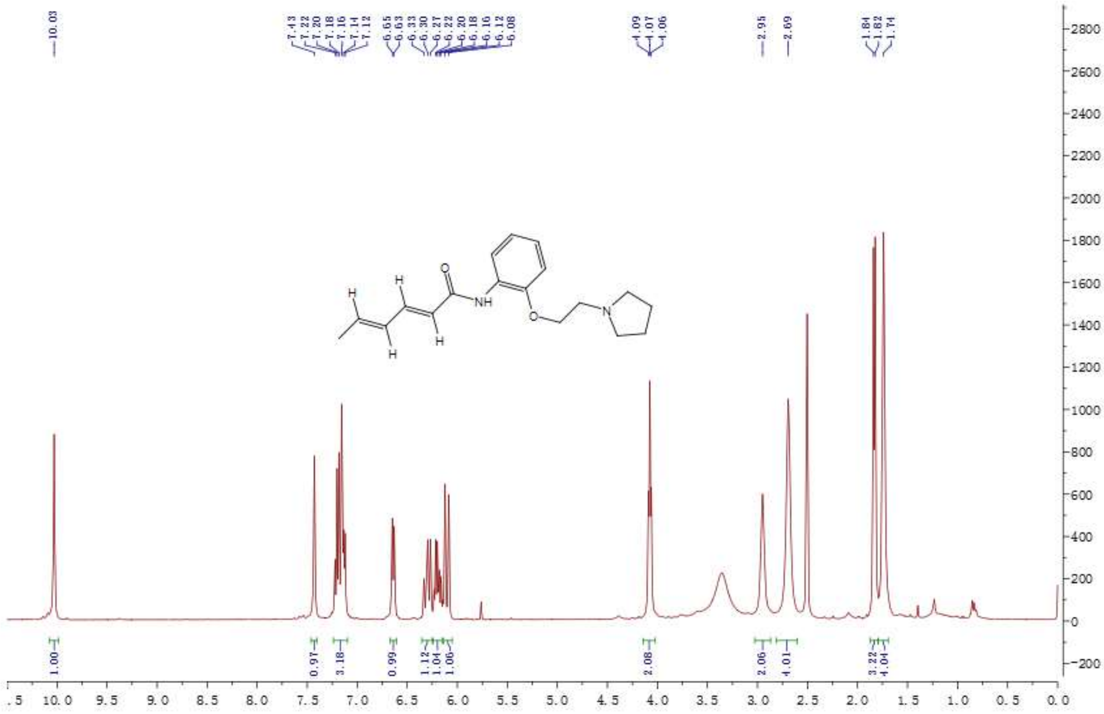

The ^{1}H-NMR (400MHz, DMSO-d_6) of compound **16g**

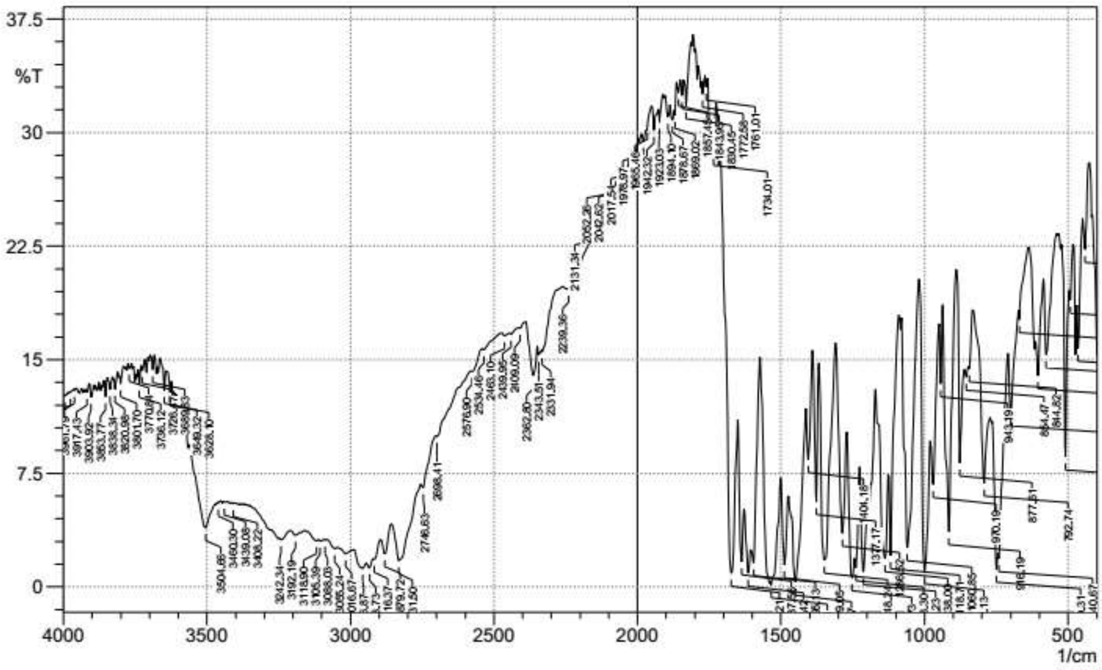

The IR spectra of compound **16g**

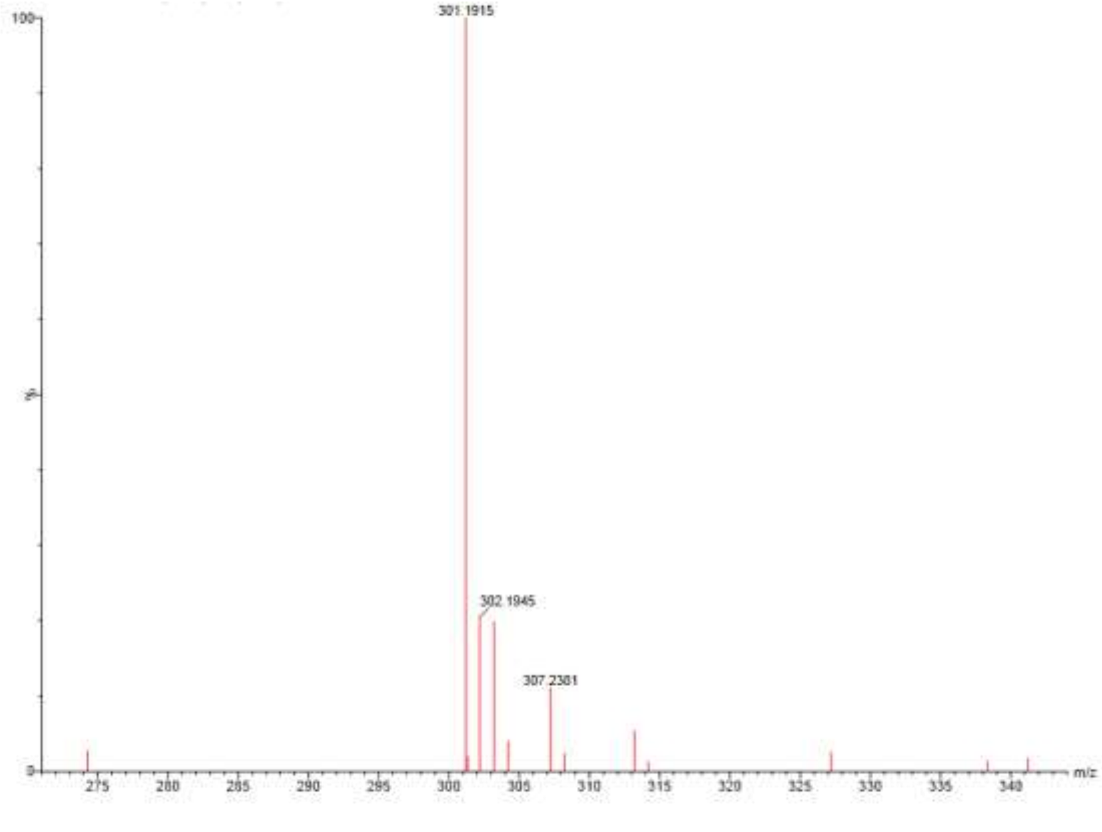

The HRMS of compound **16g**

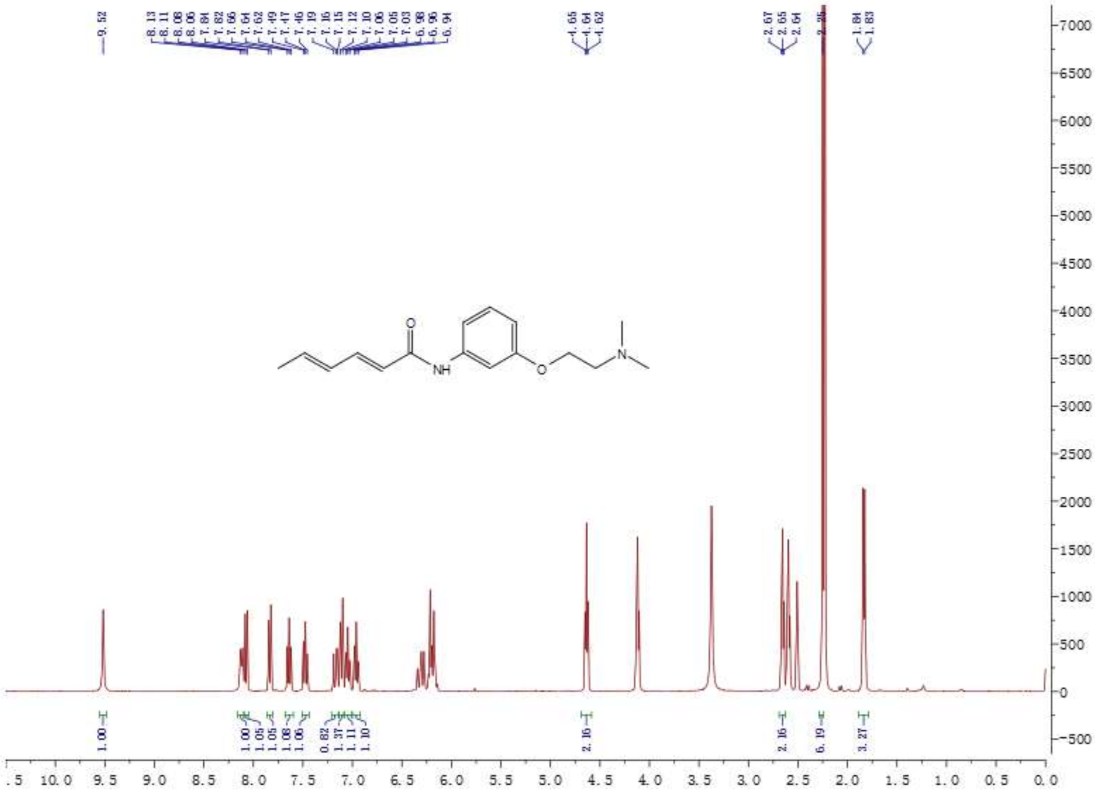

The ^{1}H-NMR (400MHz, DMSO-d_6) of compound **17d**

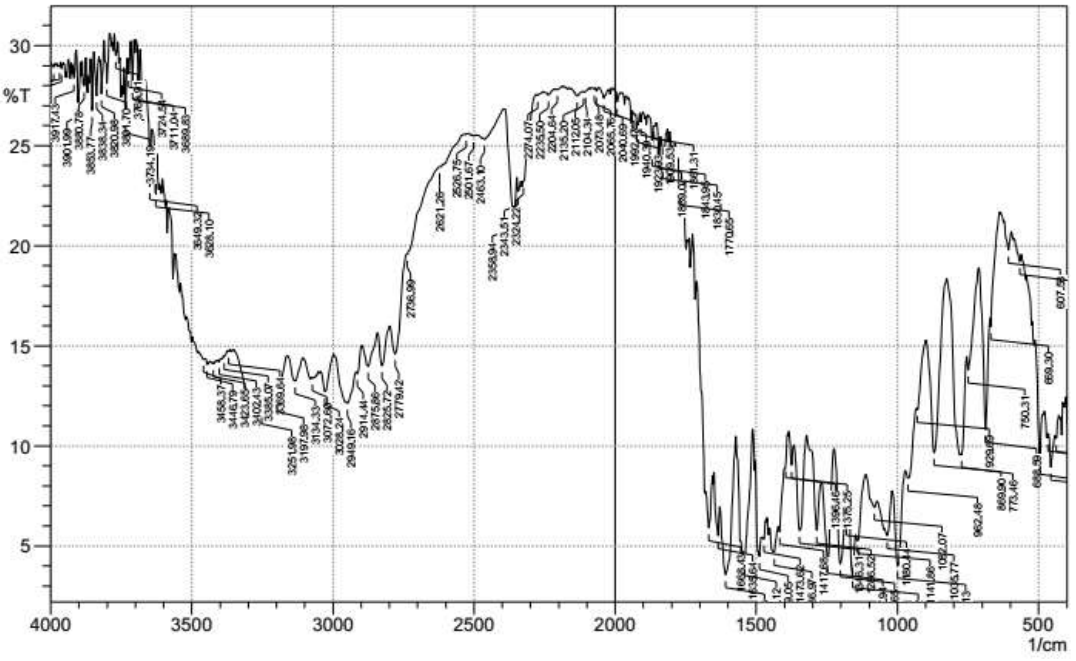

The IR spectra of compound **17d**

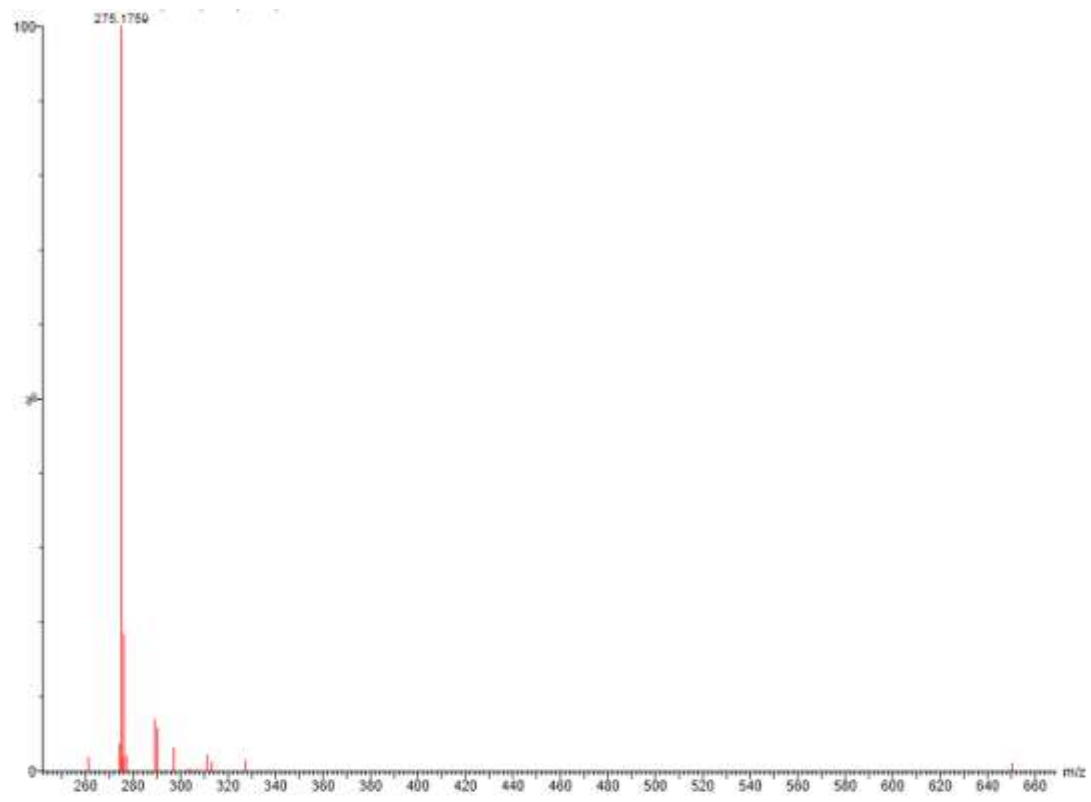

The HRMS of compound **17d**

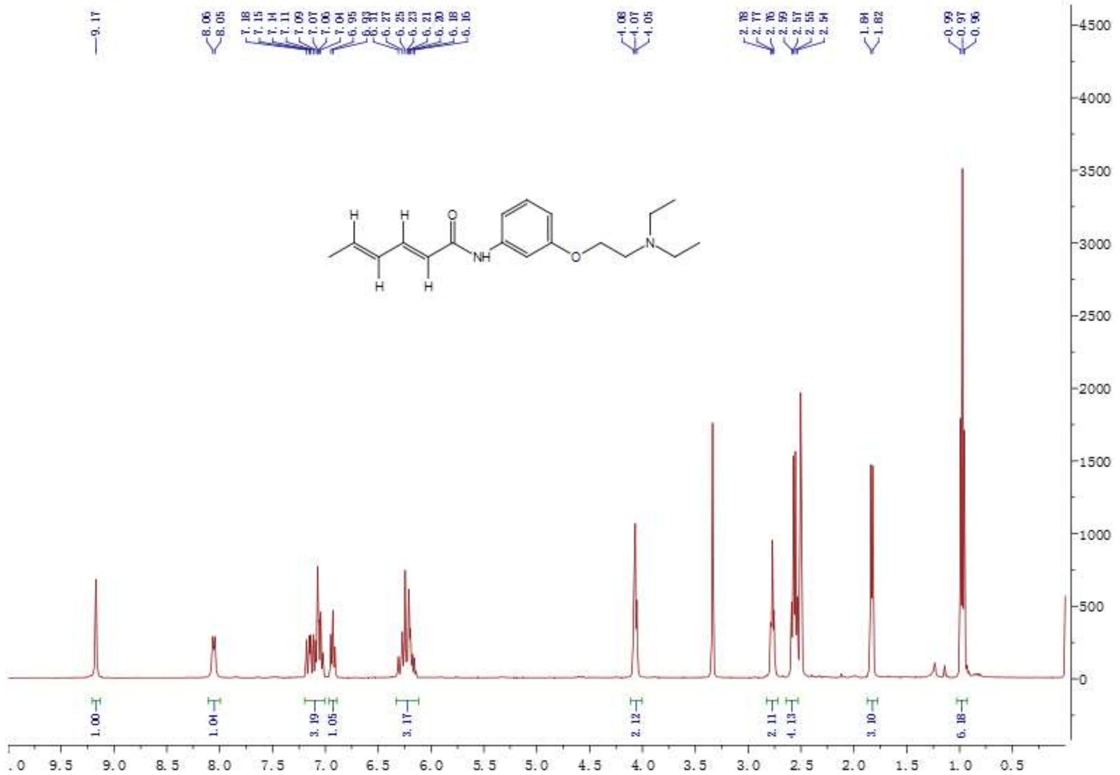

The ^{1}H-NMR (400MHz, DMSO-d_6) of compound **17e**

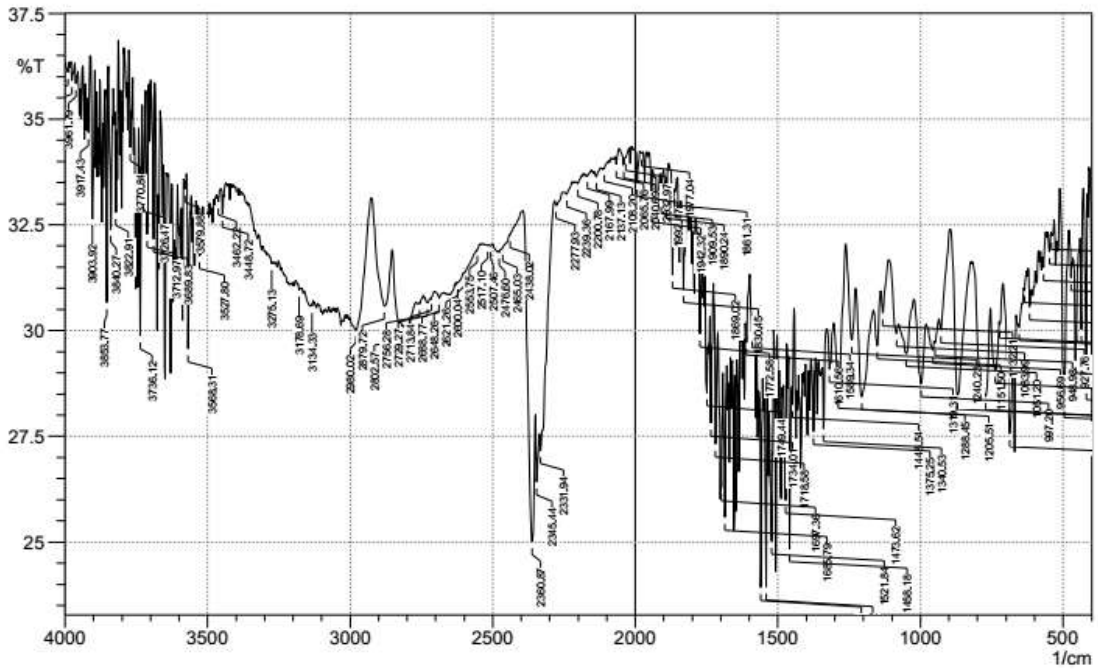

The IR spectra of compound **17e**

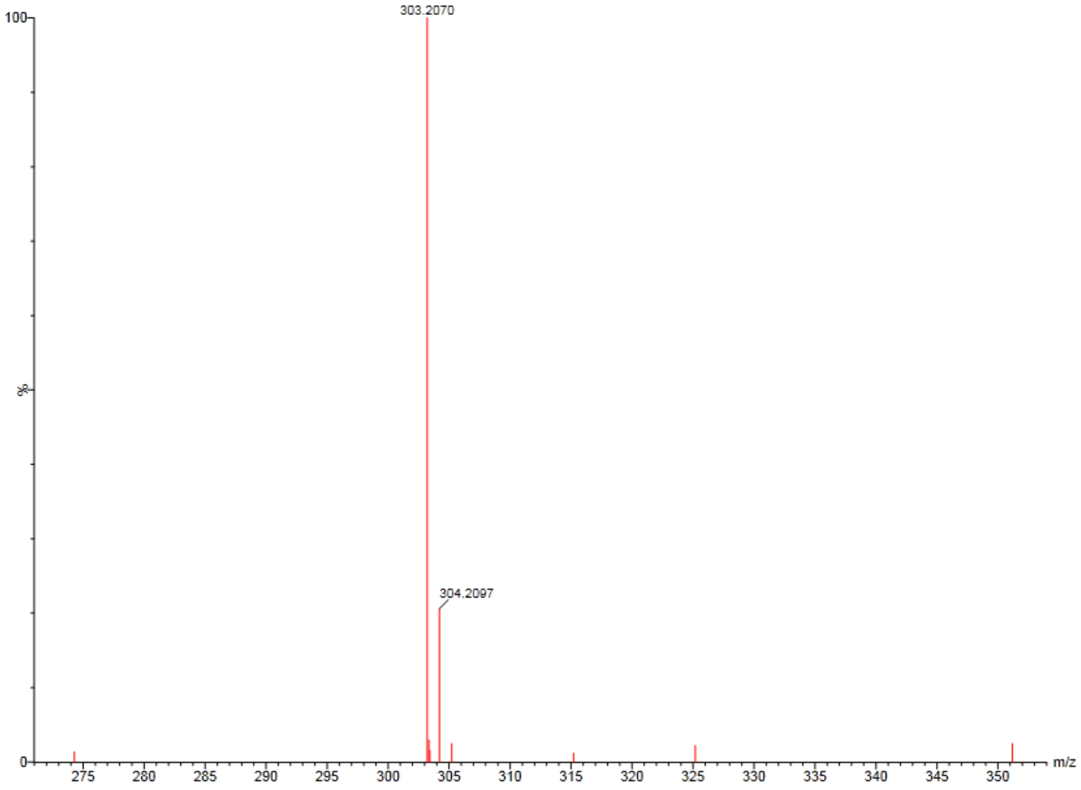

The HRMS of compound **17e**

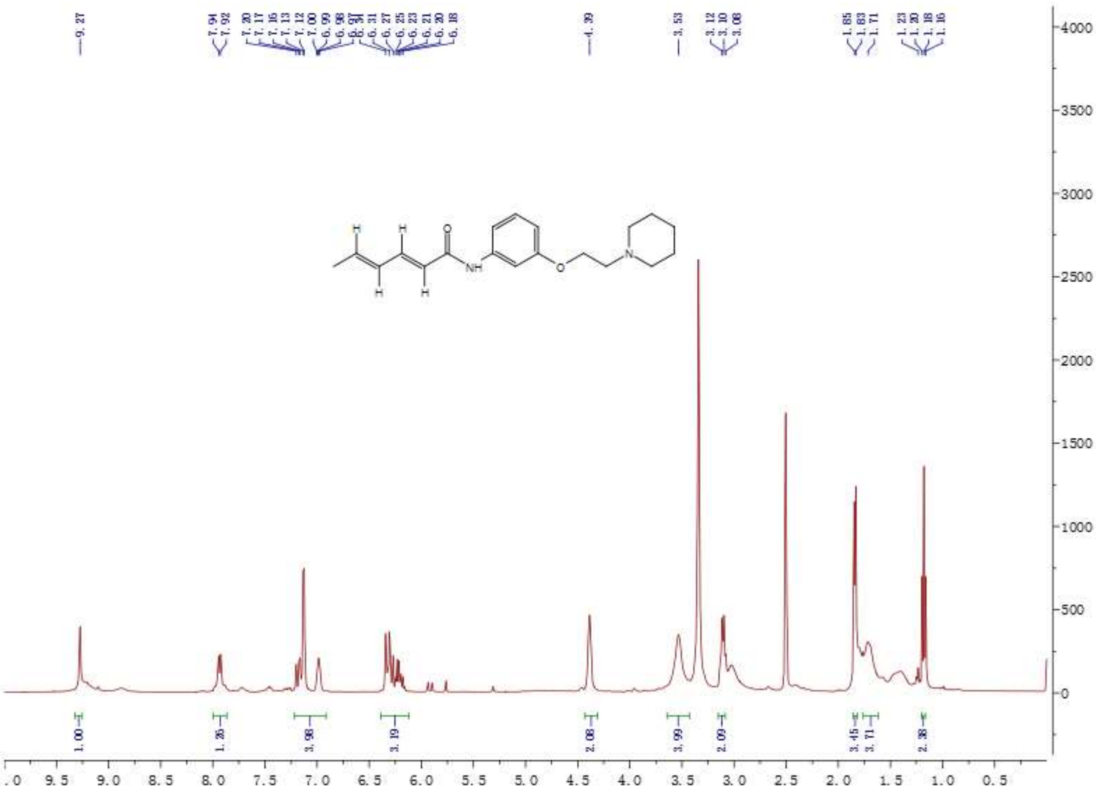

The ^{1}H-NMR (400MHz, DMSO-d_6) of compound **17f**

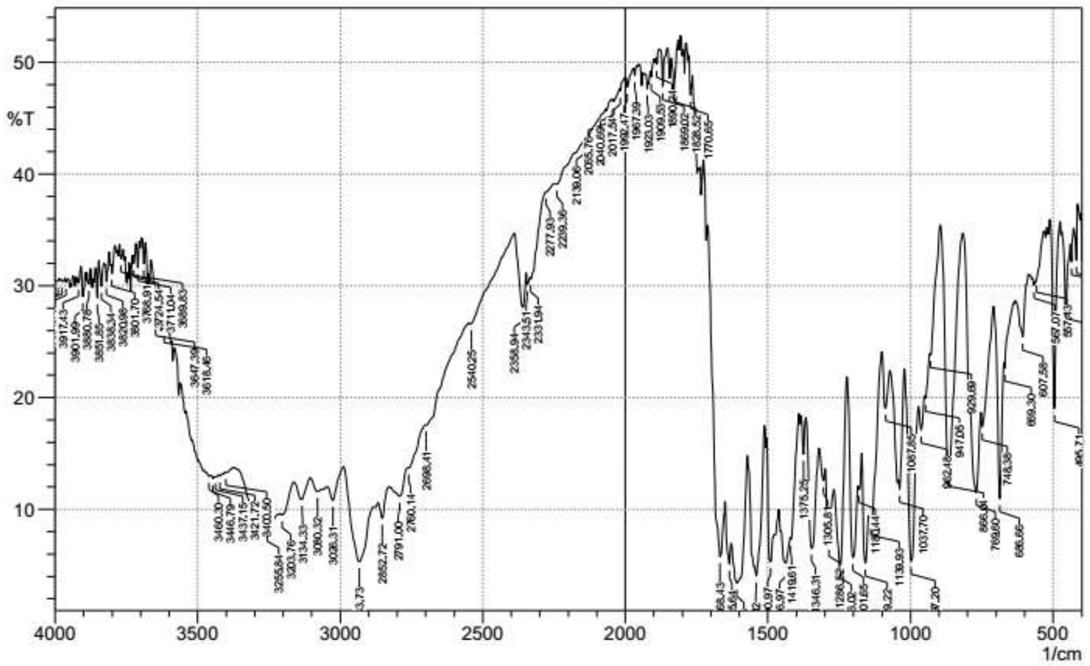

The IR spectra of compound **17f**

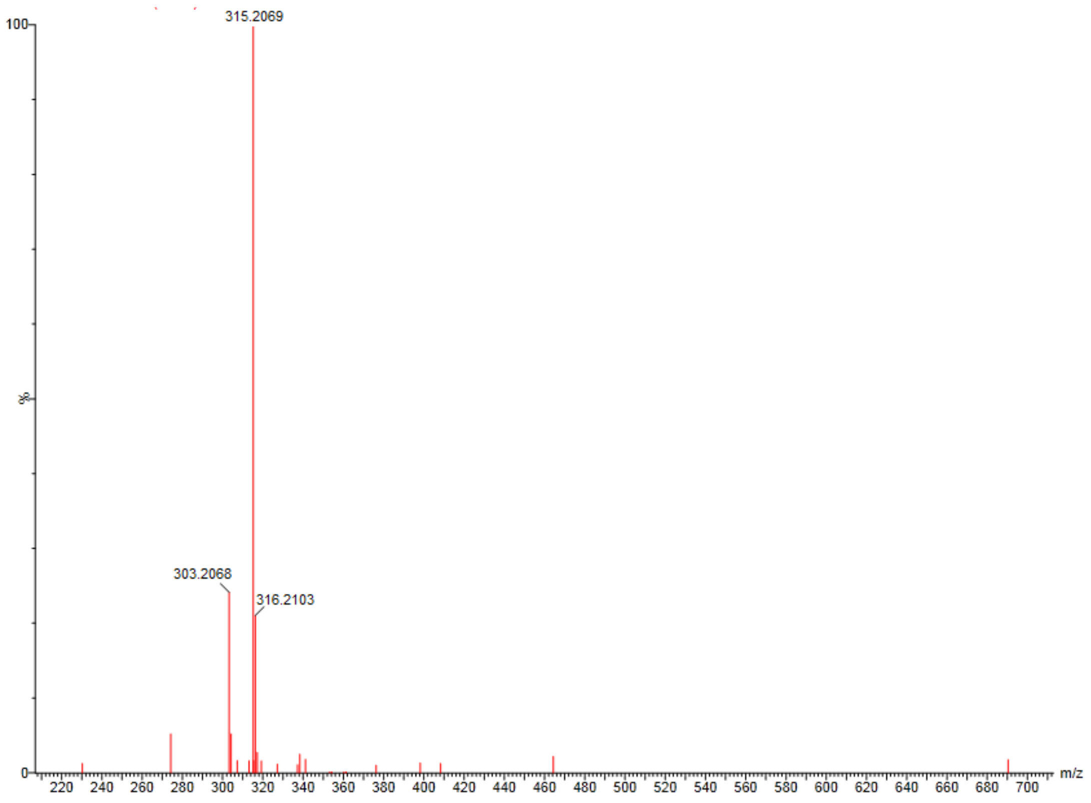

The HRMS of compound **17f**

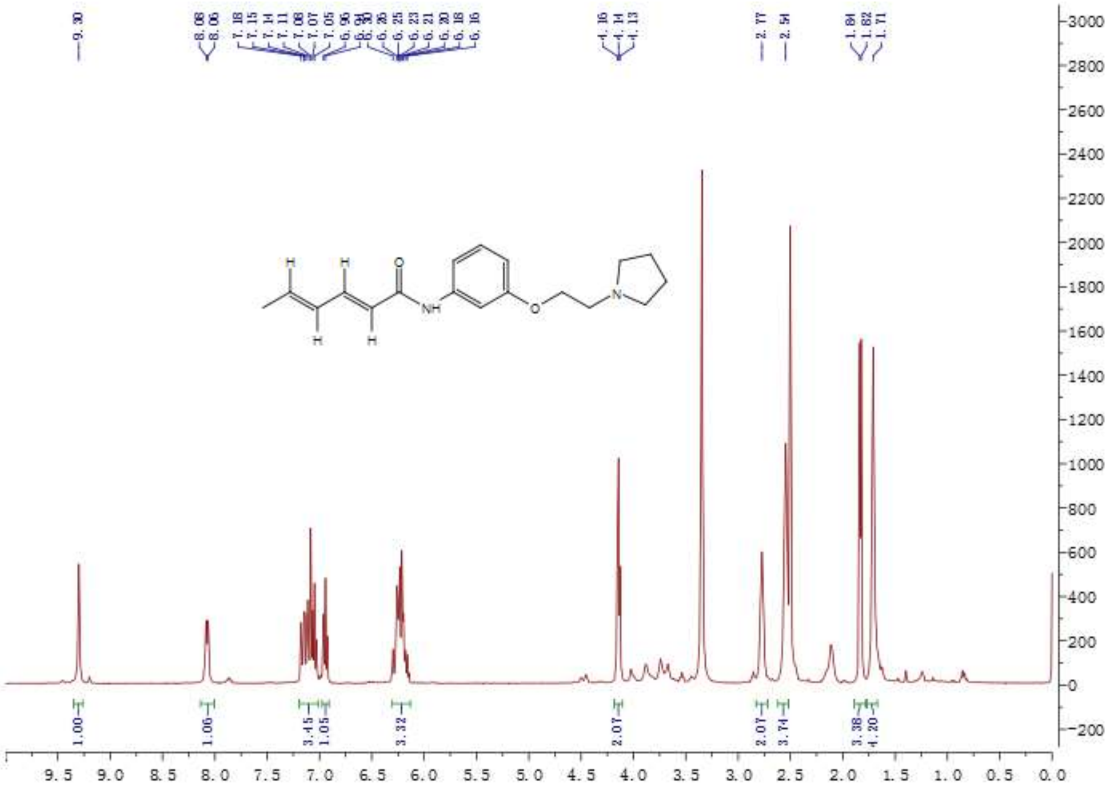

The ^{1}H-NMR (400MHz, DMSO-d_6) of compound **17g**

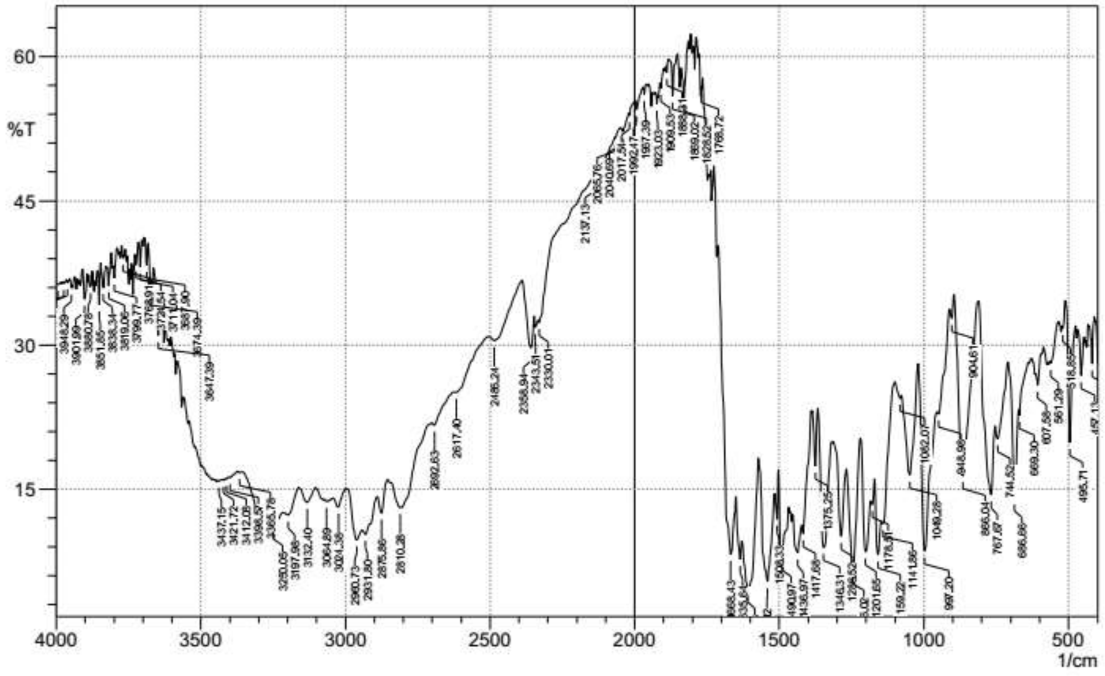

The IR spectra of compound **17g**

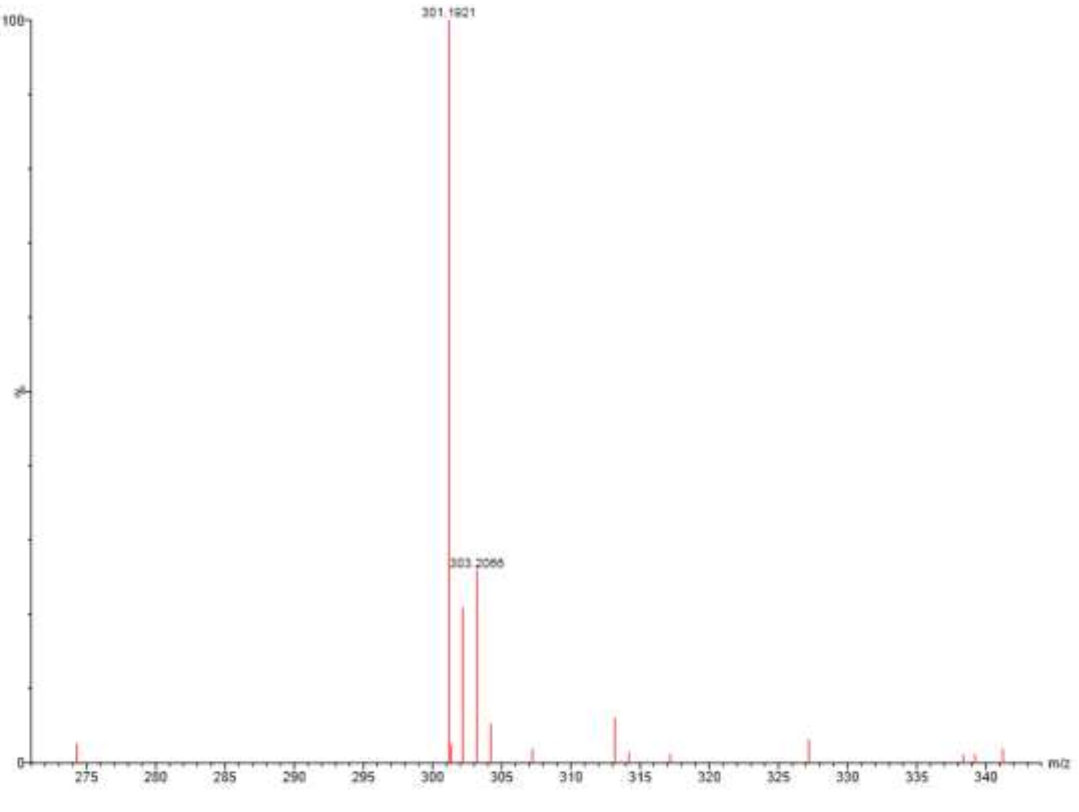

The HRMS of compound **17g**

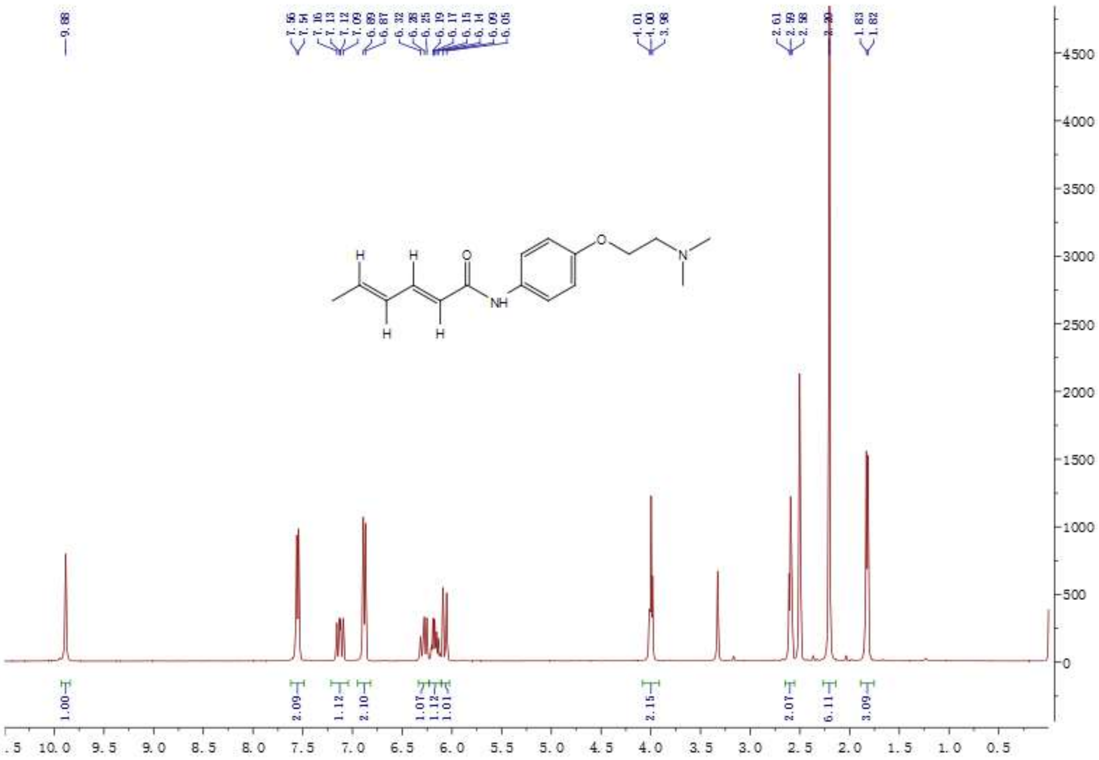

The ^{1}H-NMR (400MHz, DMSO-d_6) of compound **18d**

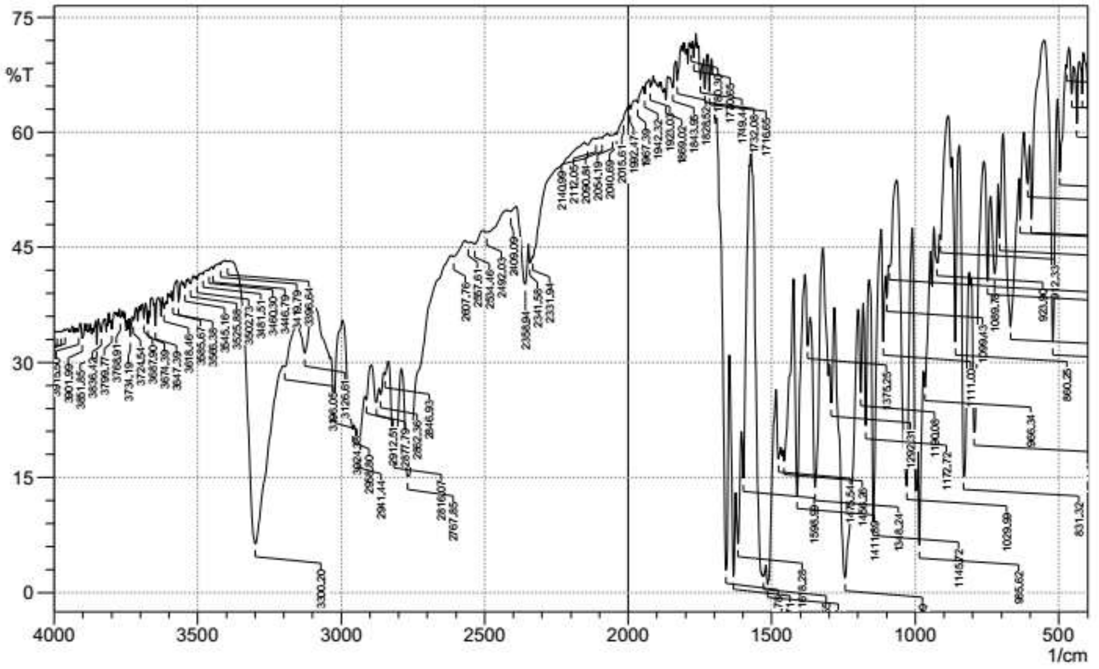

The IR spectra of compound **18d**

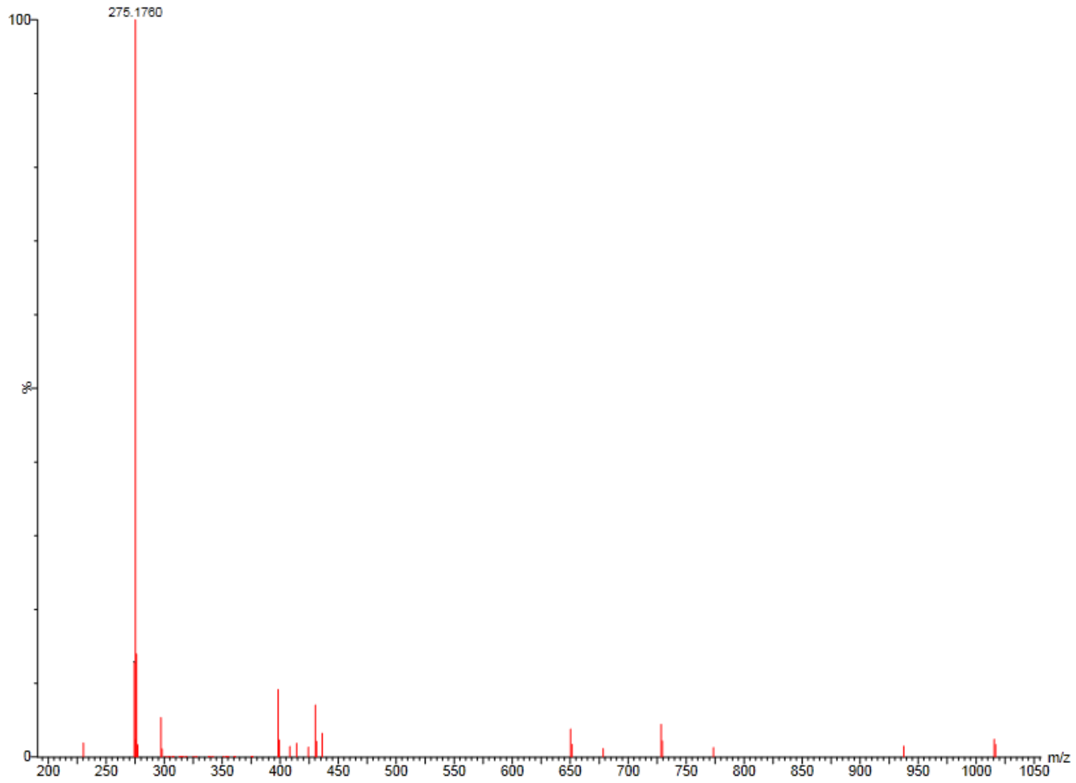

The HRMS of compound **18d**

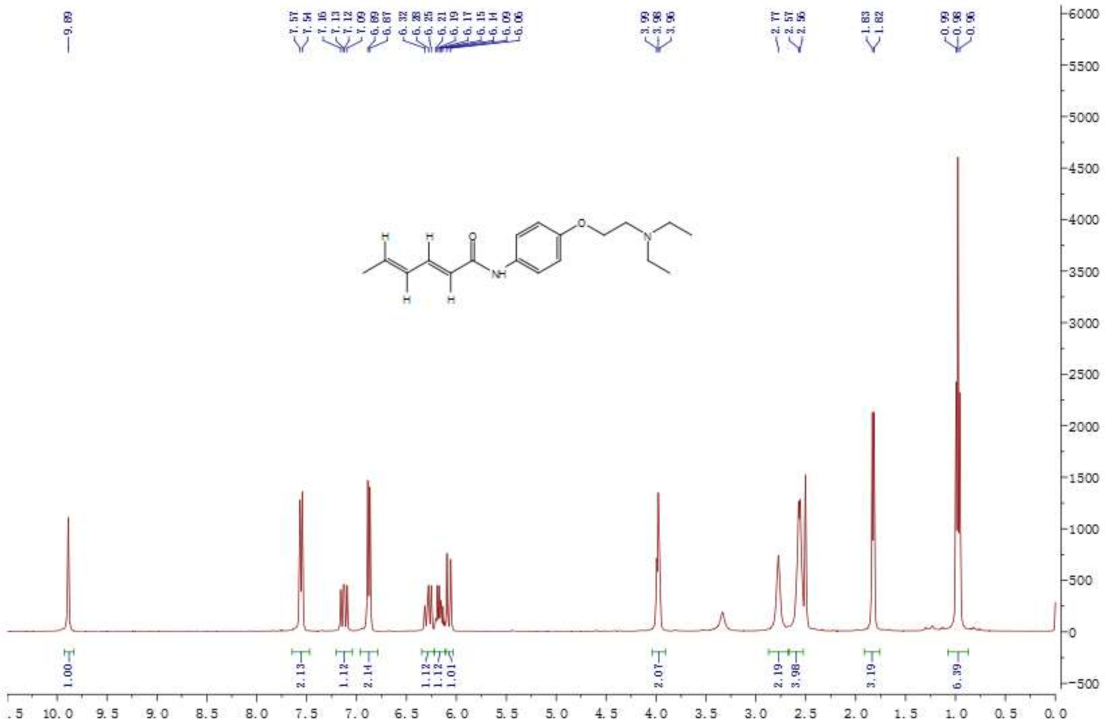

The ^{1}H-NMR (400MHz, DMSO-d_6) of compound **18e**

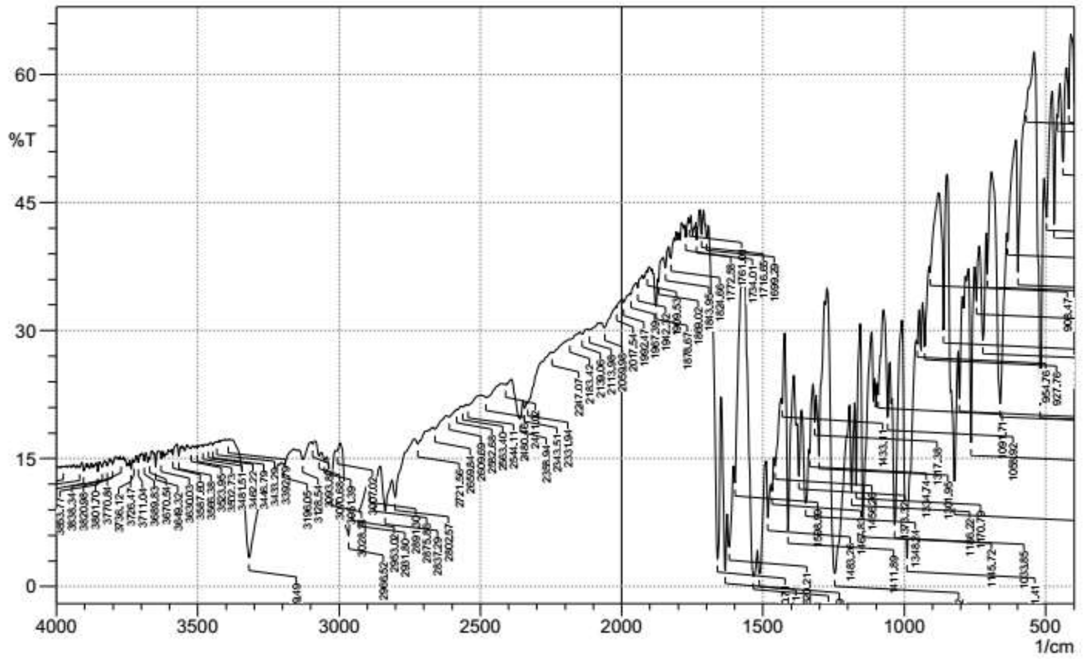

The IR spectra of compound **18e**

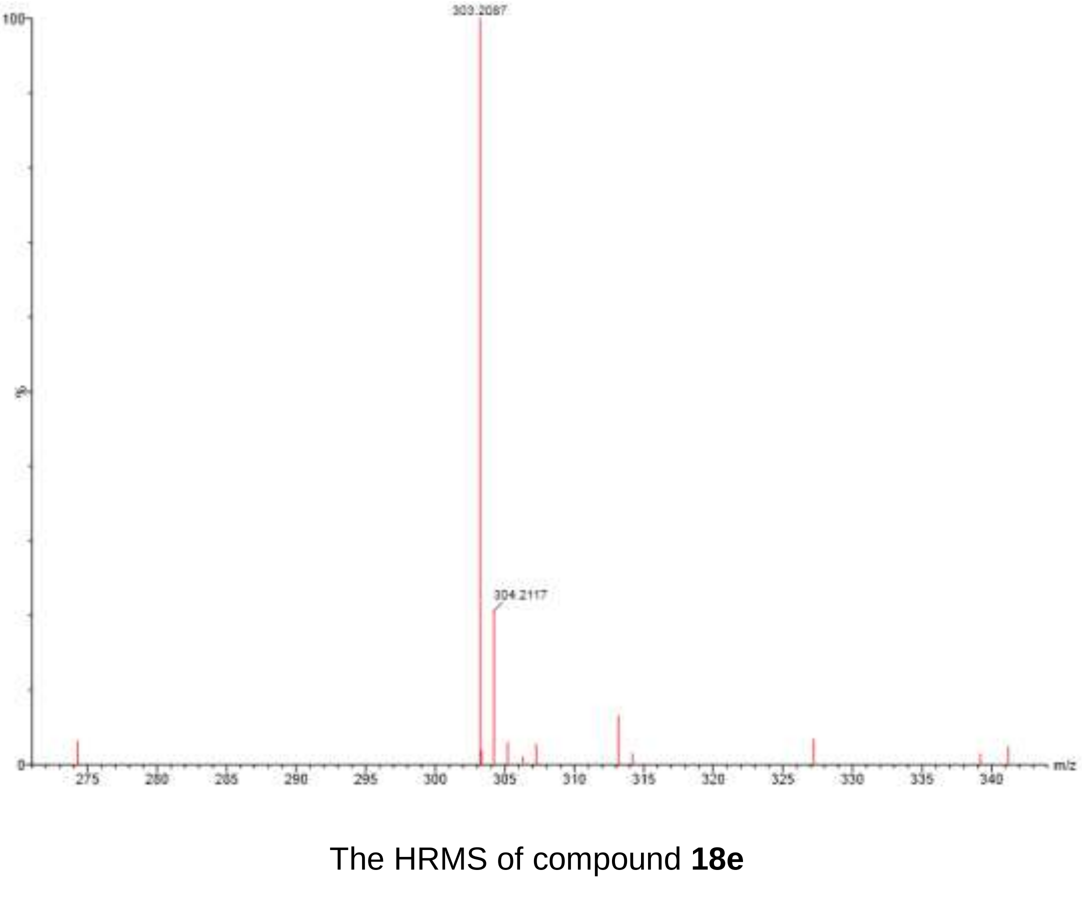

The HRMS of compound **18e**

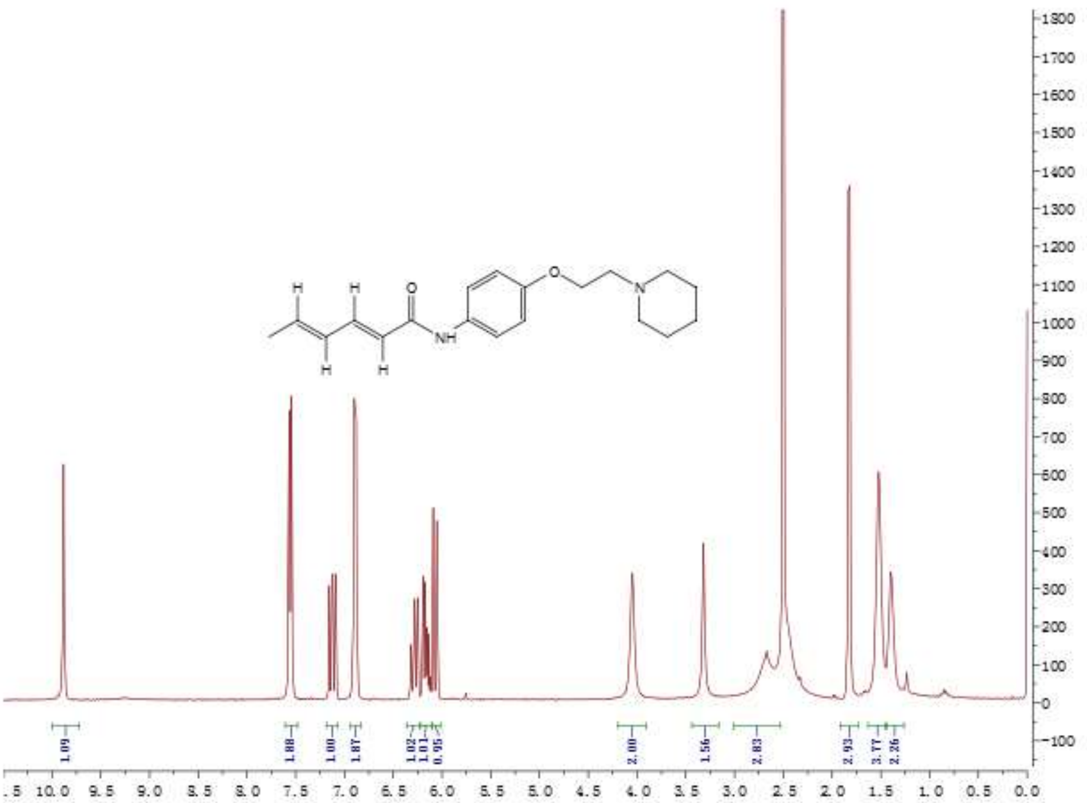

The ^{1}H-NMR (400MHz, DMSO-d_6) of compound **18f**

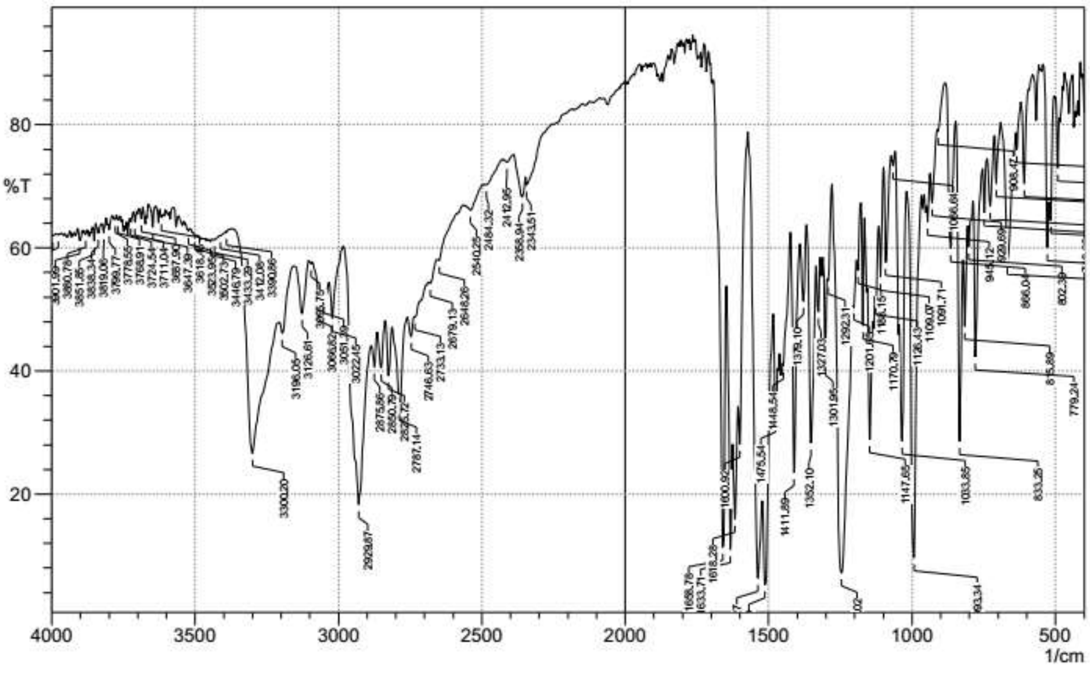

The IR spectra of compound **18f**

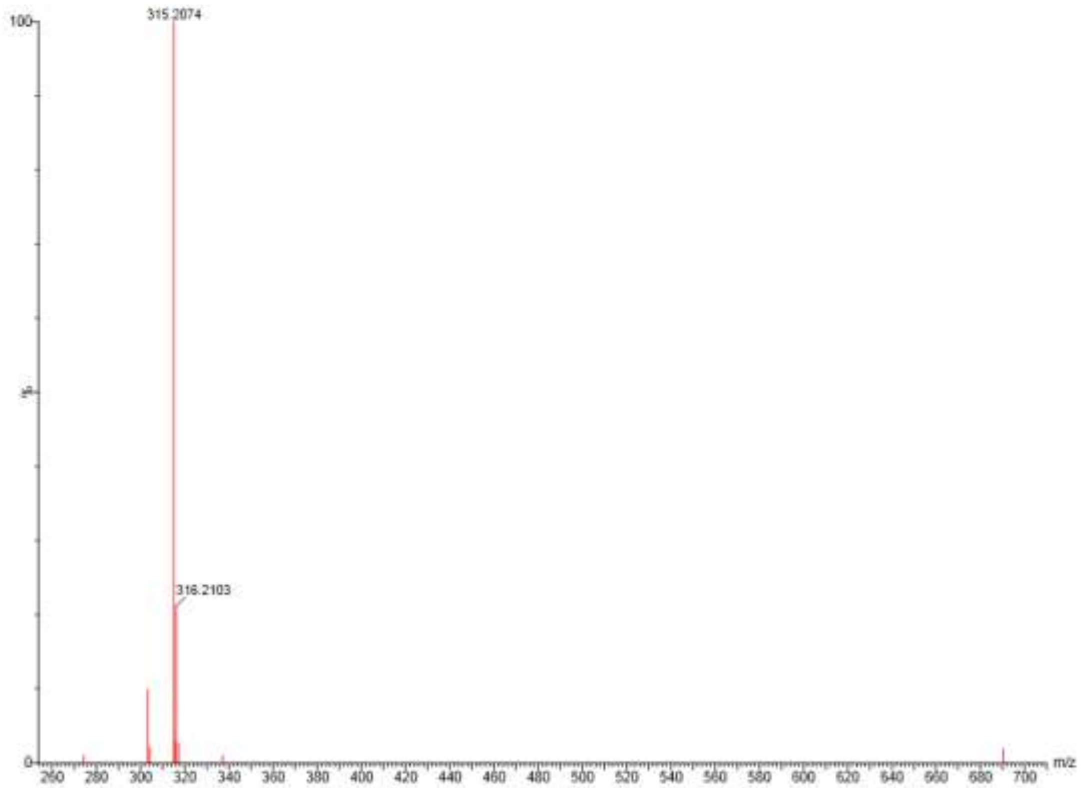

The HRMS of compound **18f**

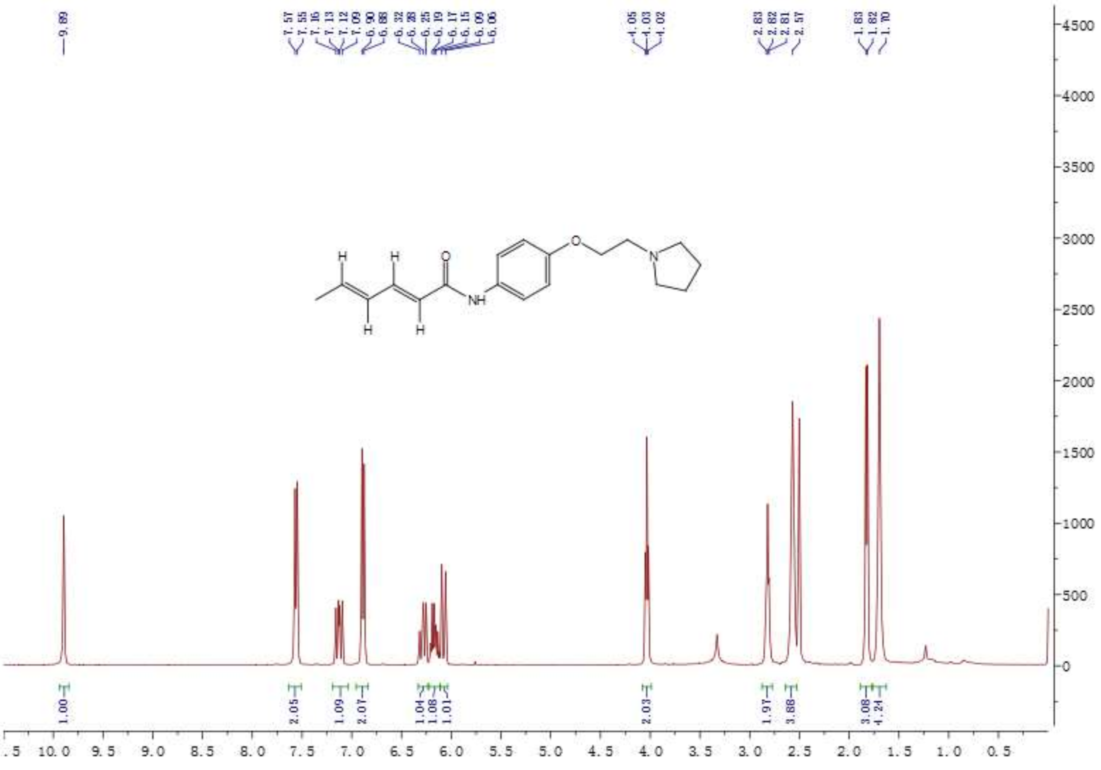

The ^{1}H-NMR (400MHz, DMSO-d_6) of compound **18g**

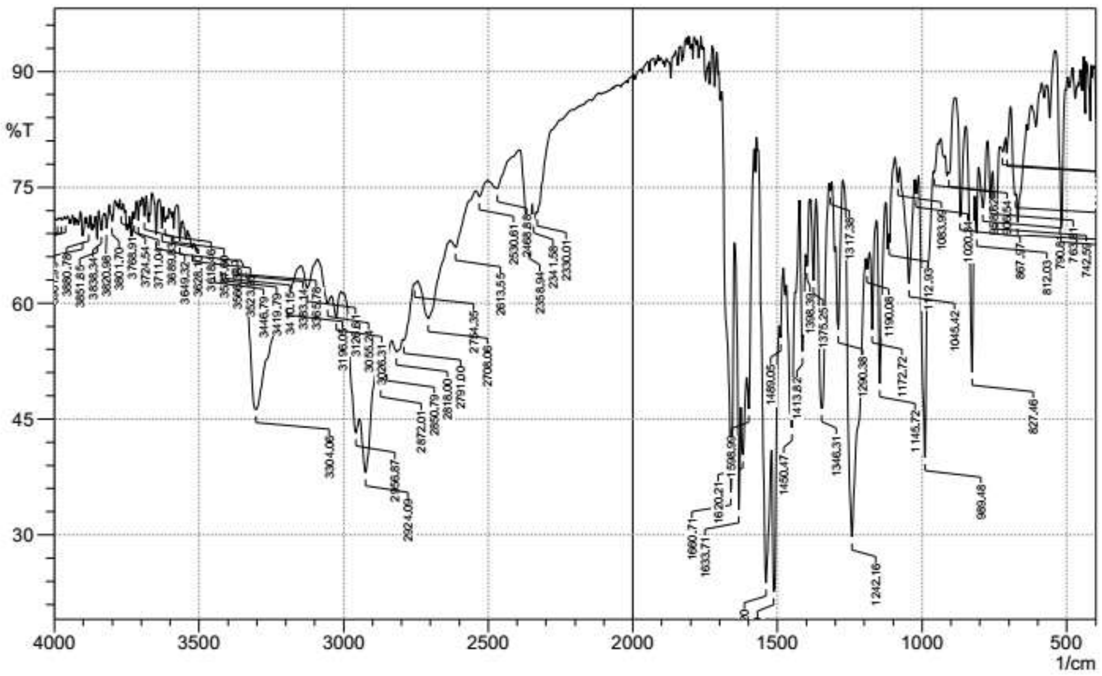

The IR spectra of compound **18g**

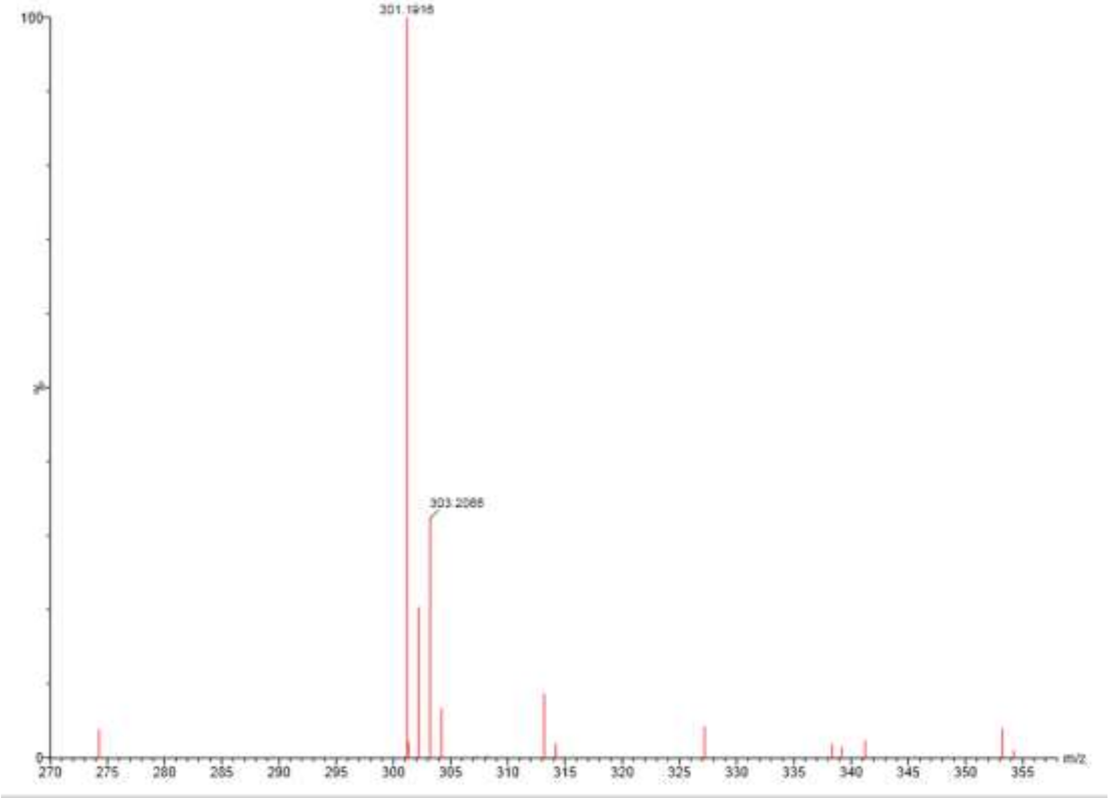

The HRMS of compound **18g**

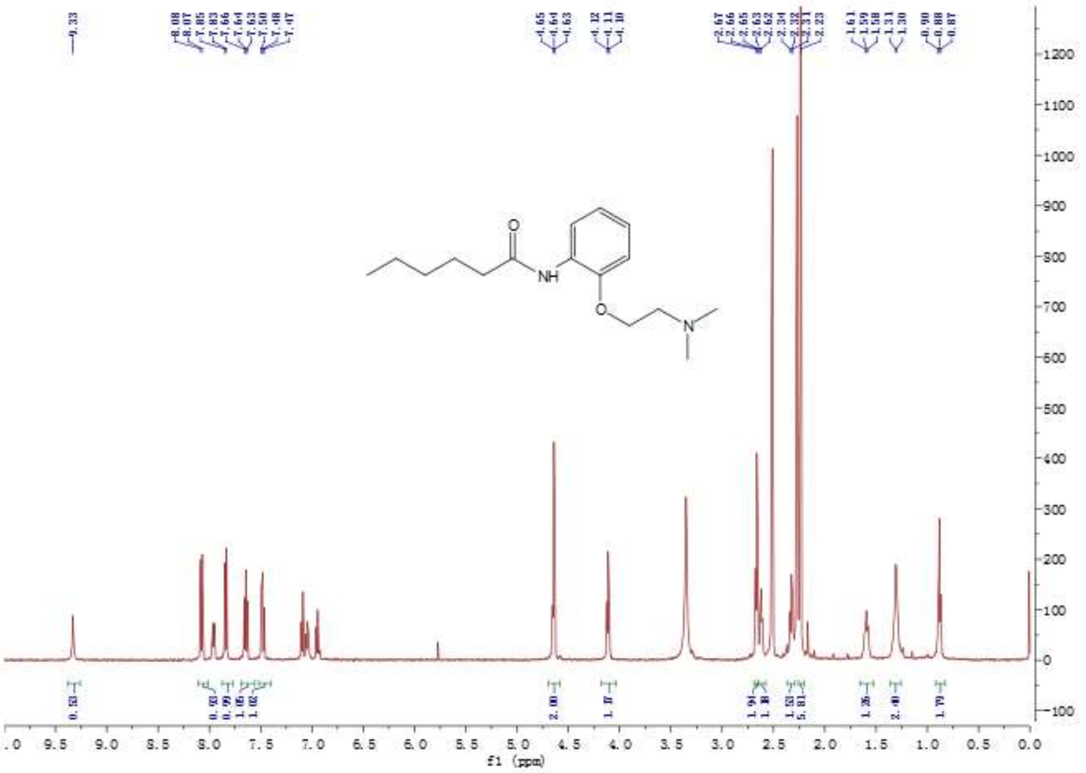

The ^{1}H-NMR (400MHz, DMSO-d_6) of compound **22d**

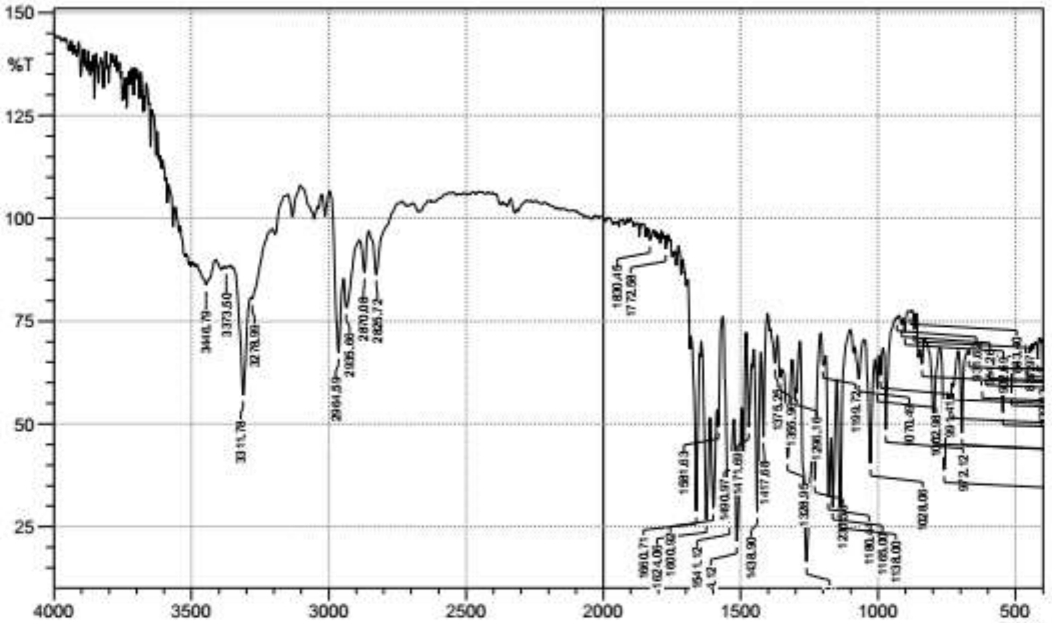

The IR spectra of compound **22d**

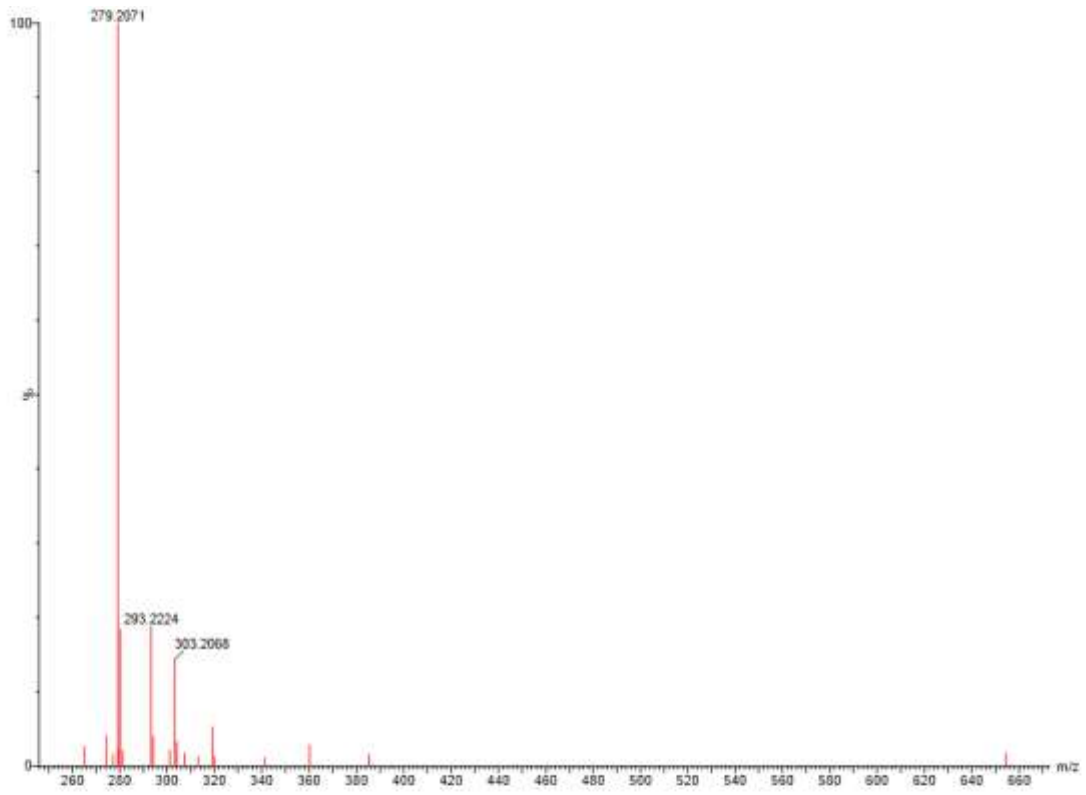

The HRMS of compound **22d**

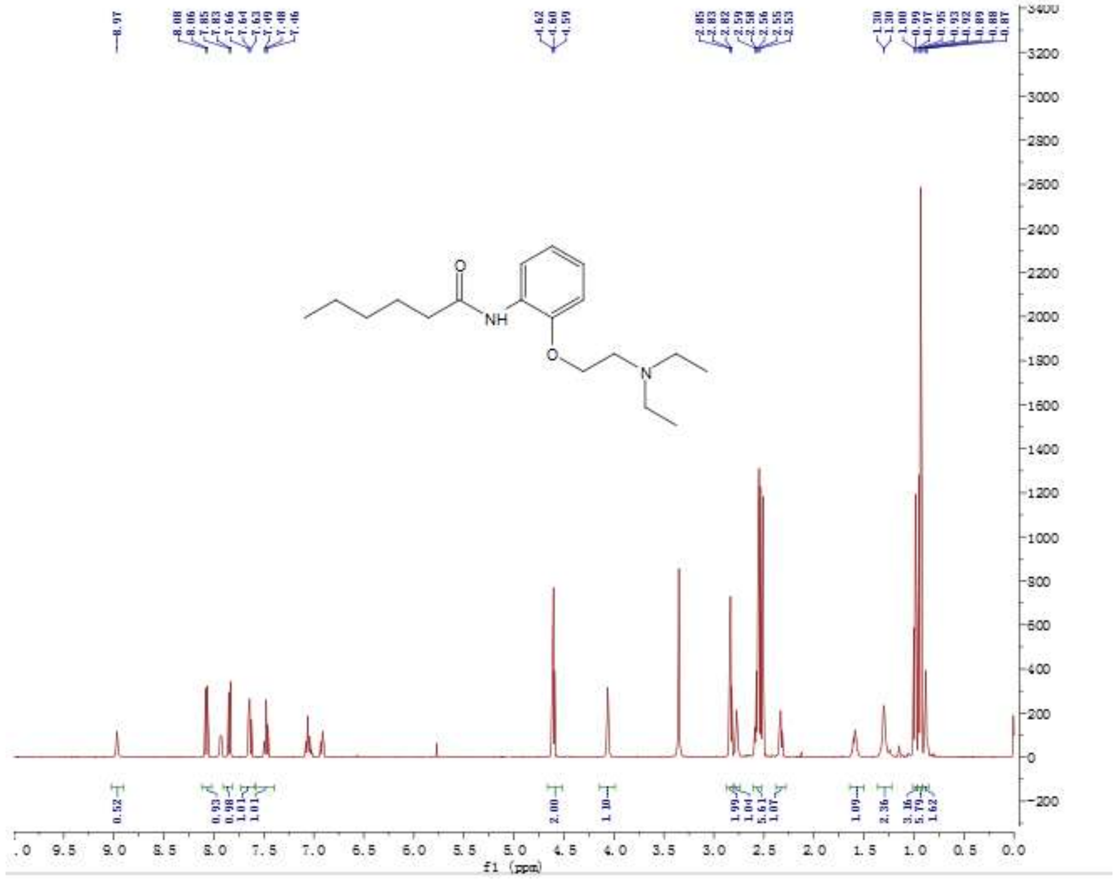

The ^{1}H-NMR (400MHz, DMSO-d_6) of compound **22e**

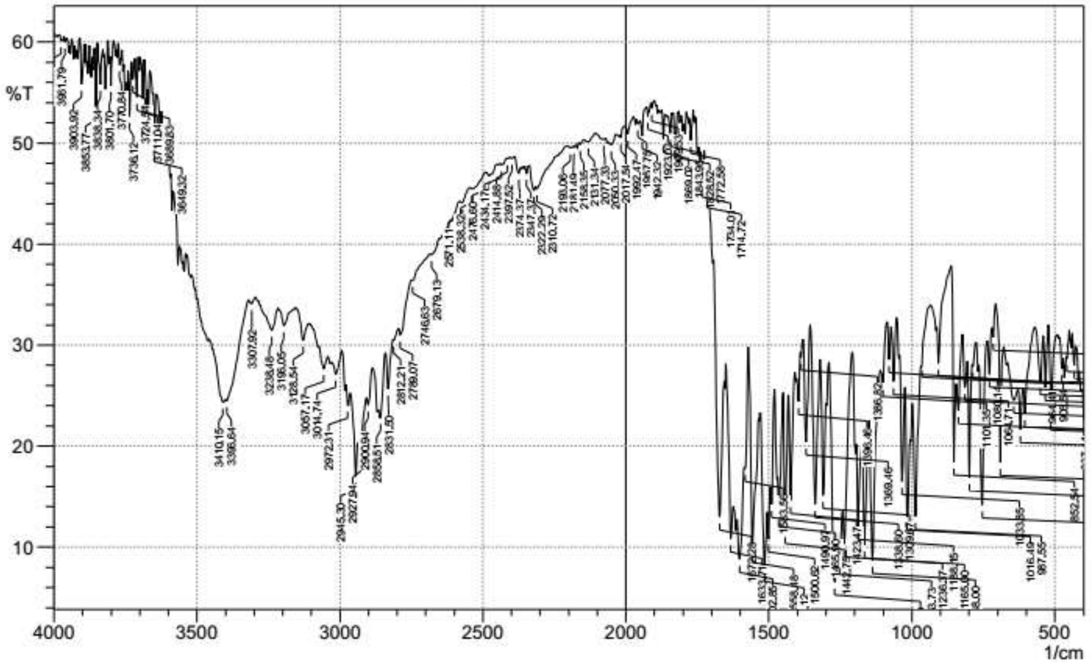

The IR spectra of compound **22e**

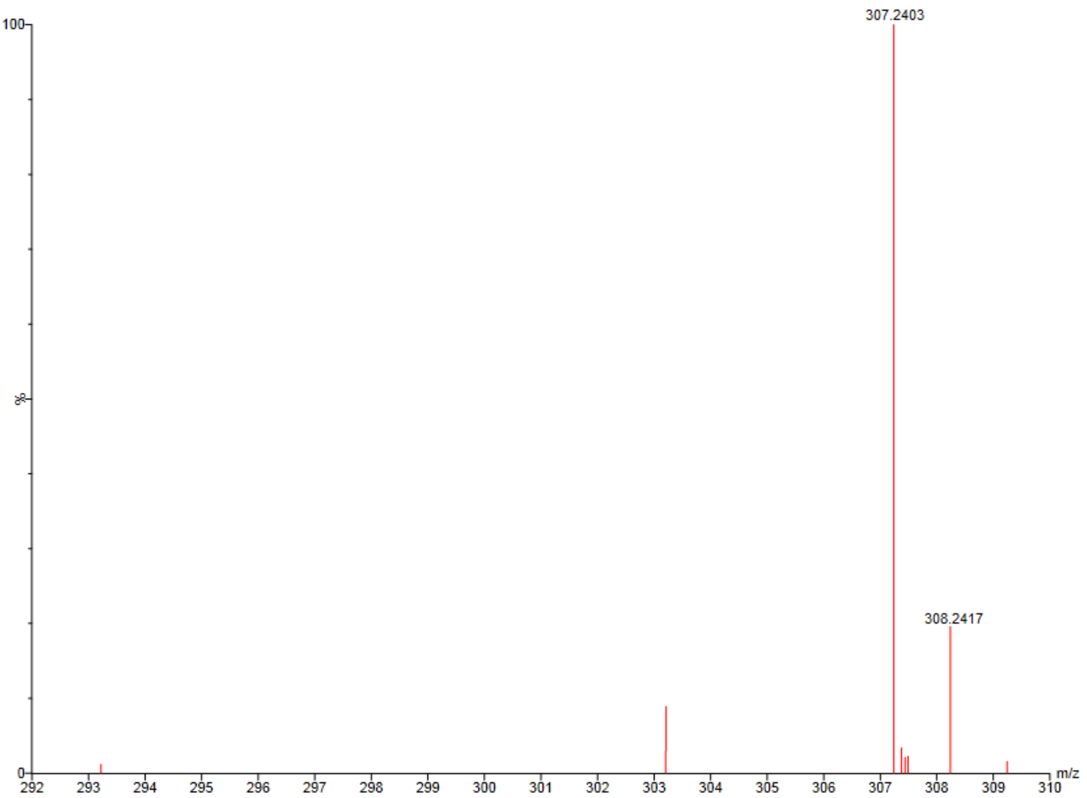

The HRMS of compound **22e**

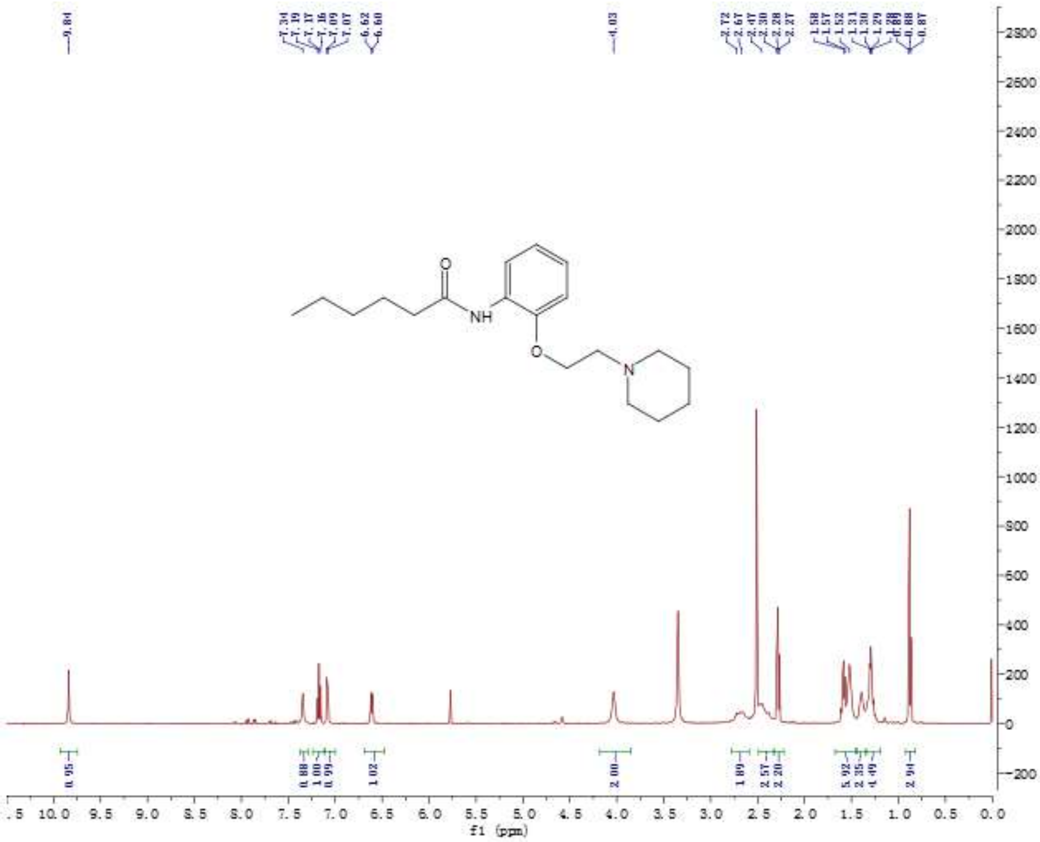

The ^{1}H-NMR (400MHz, DMSO-d_6) of compound **22f**

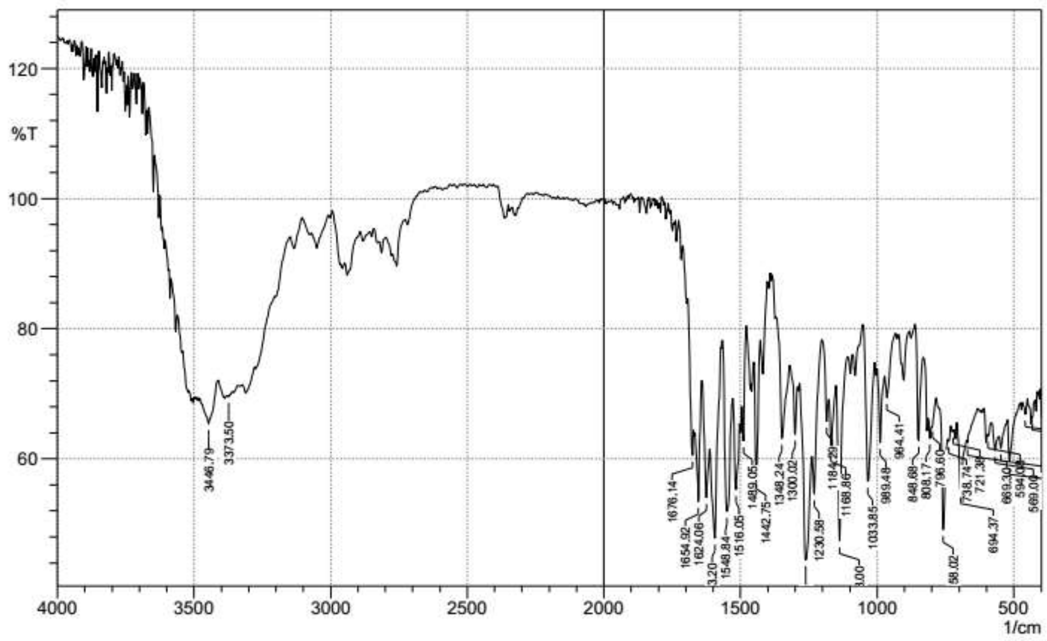

The IR spectra of compound **22f**

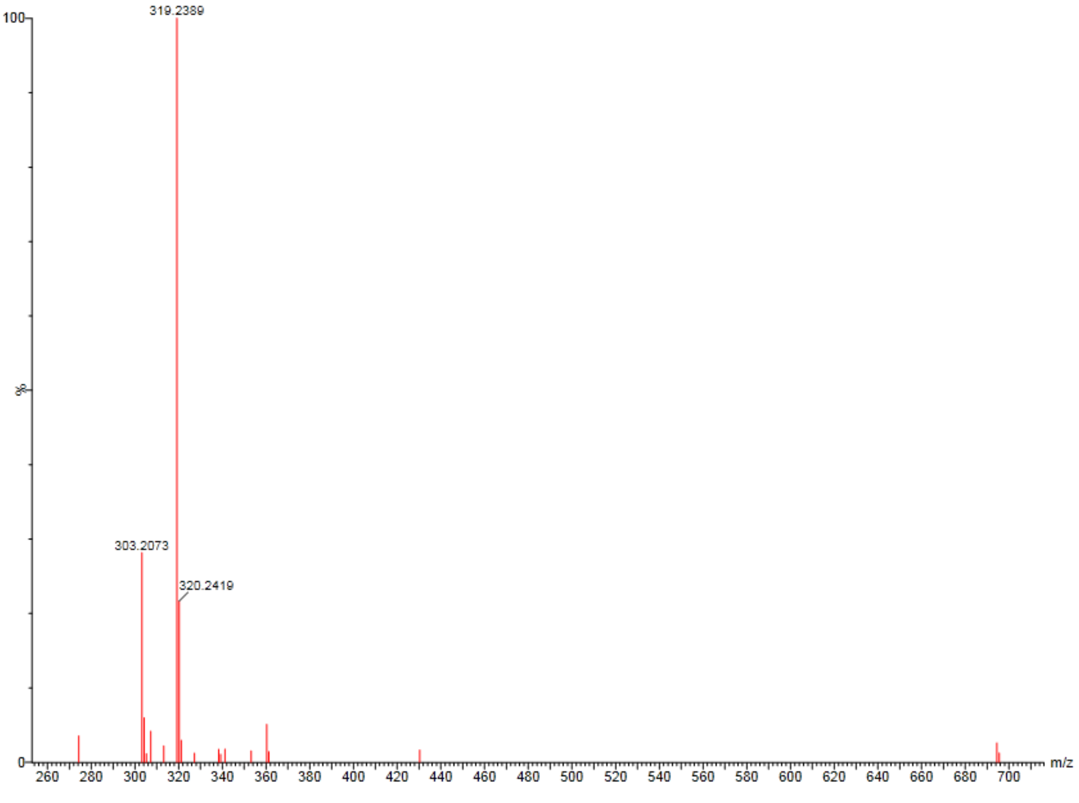

The HRMS of compound **22f**

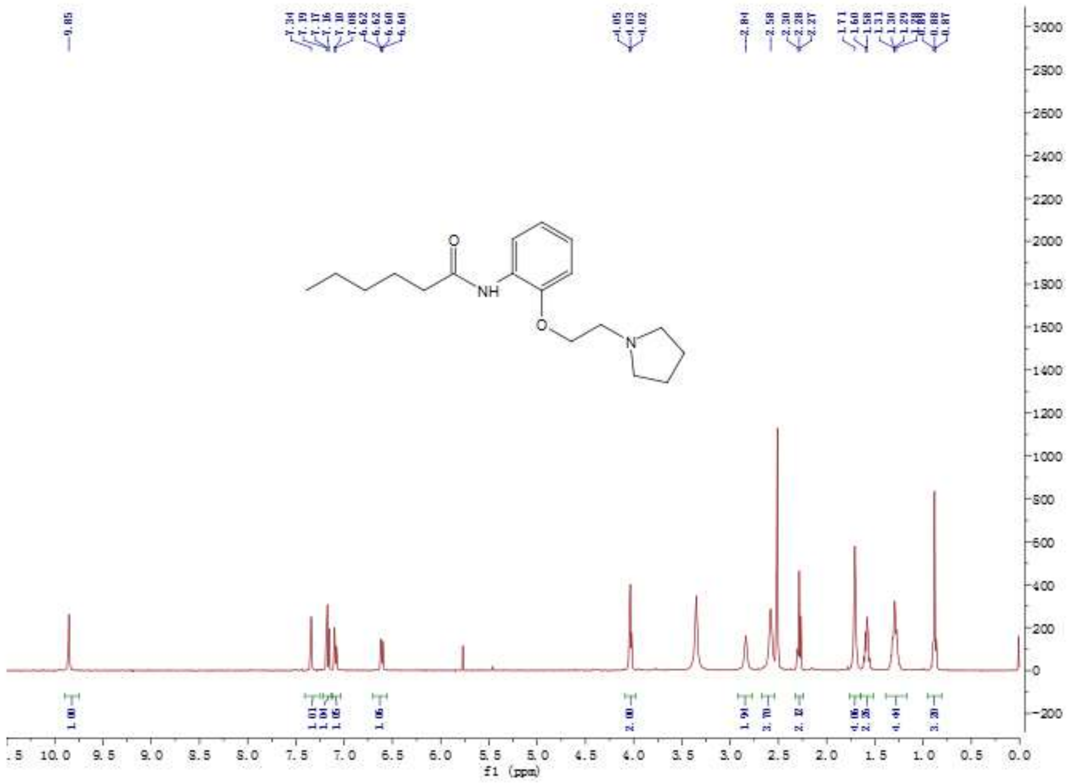

The ^{1}H-NMR (400MHz, DMSO-d_6) of compound **22g**

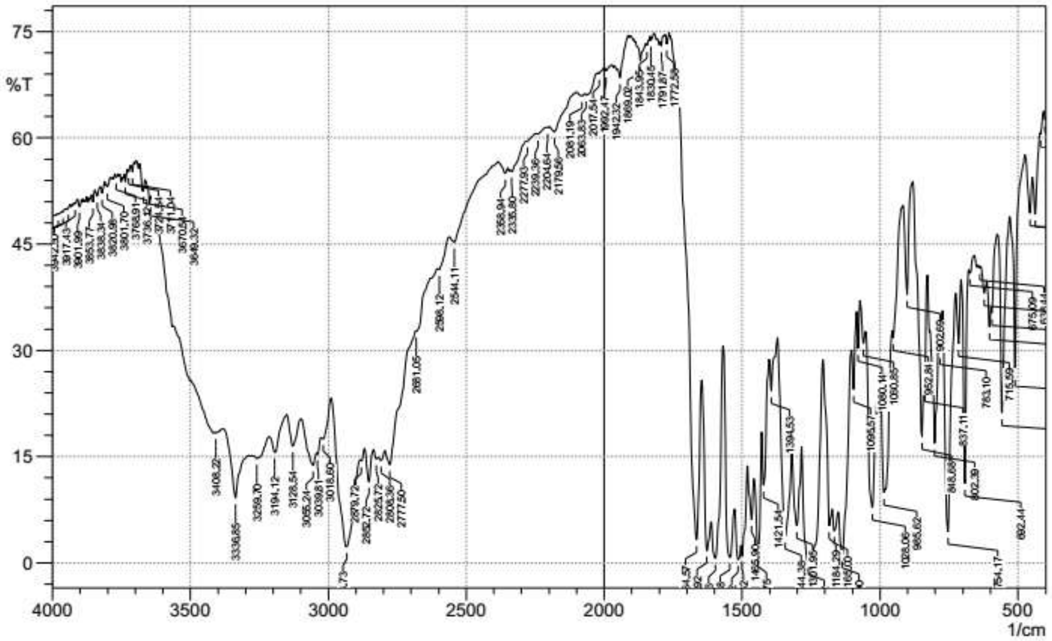

The IR spectra of compound **22g**

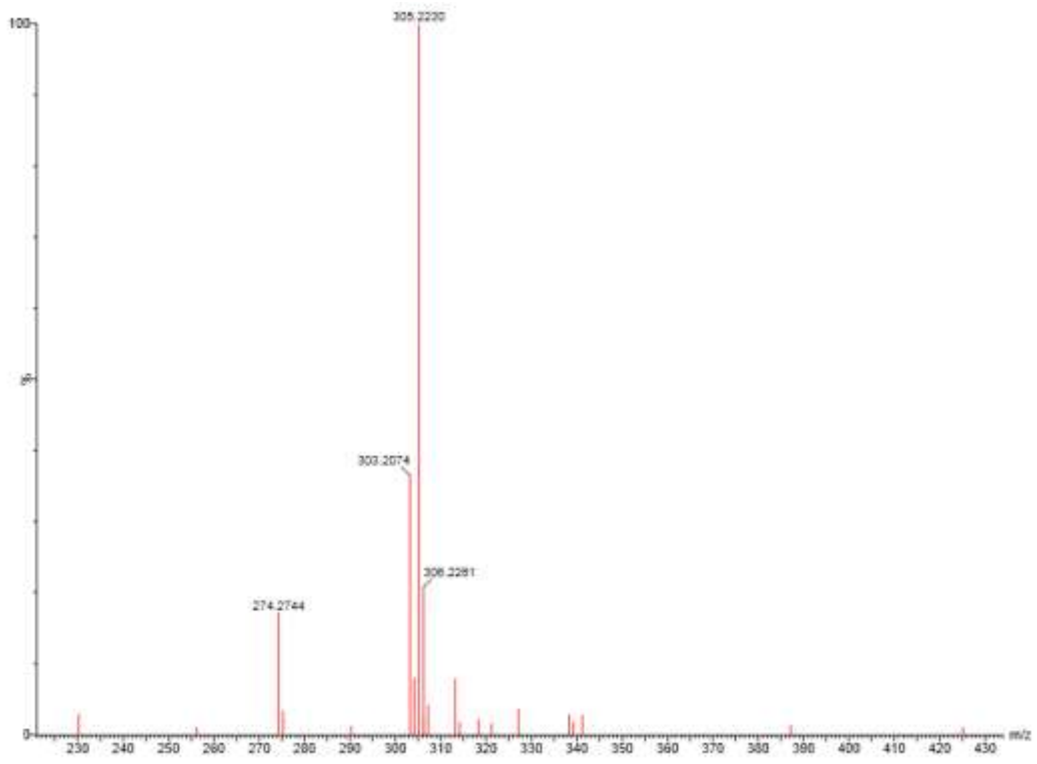

The HRMS of compound **22g**

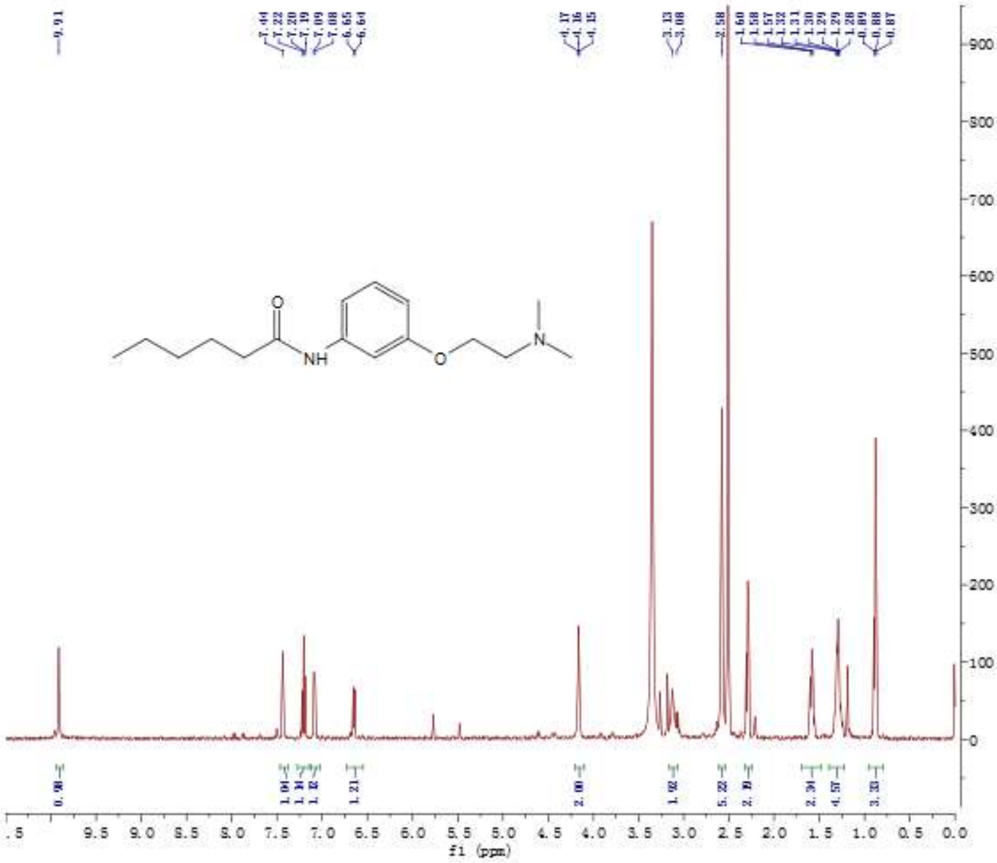

The ^{1}H-NMR (400MHz, DMSO-d_6) of compound **23d**

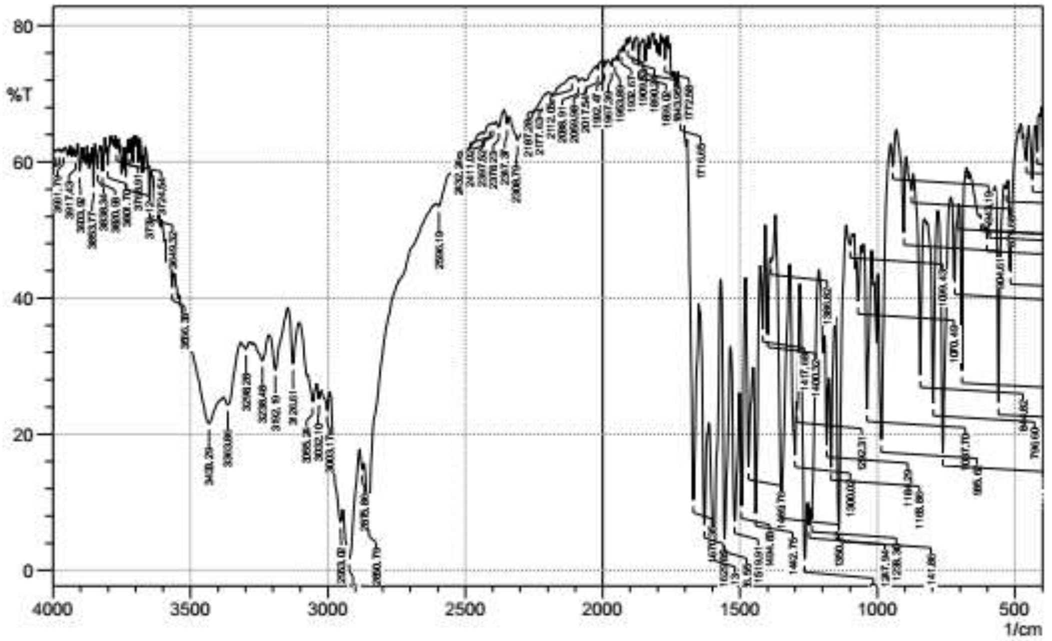

The IR spectra of compound **23d**

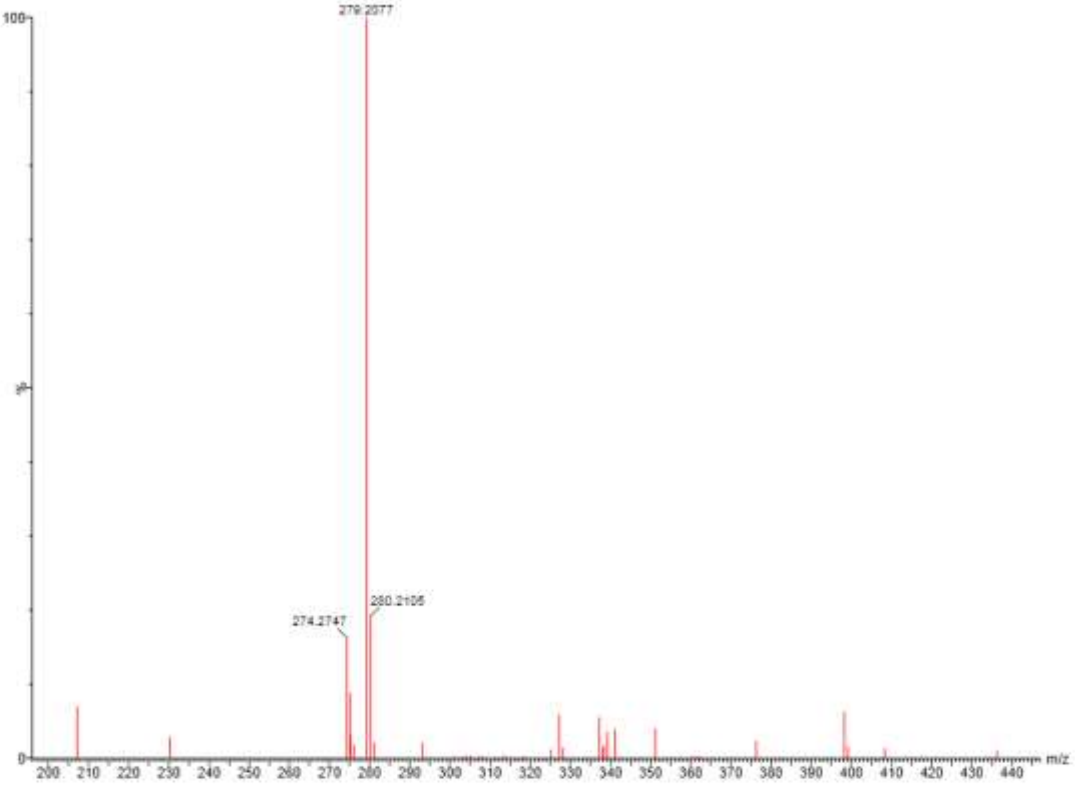

The HRMS of compound **23d**

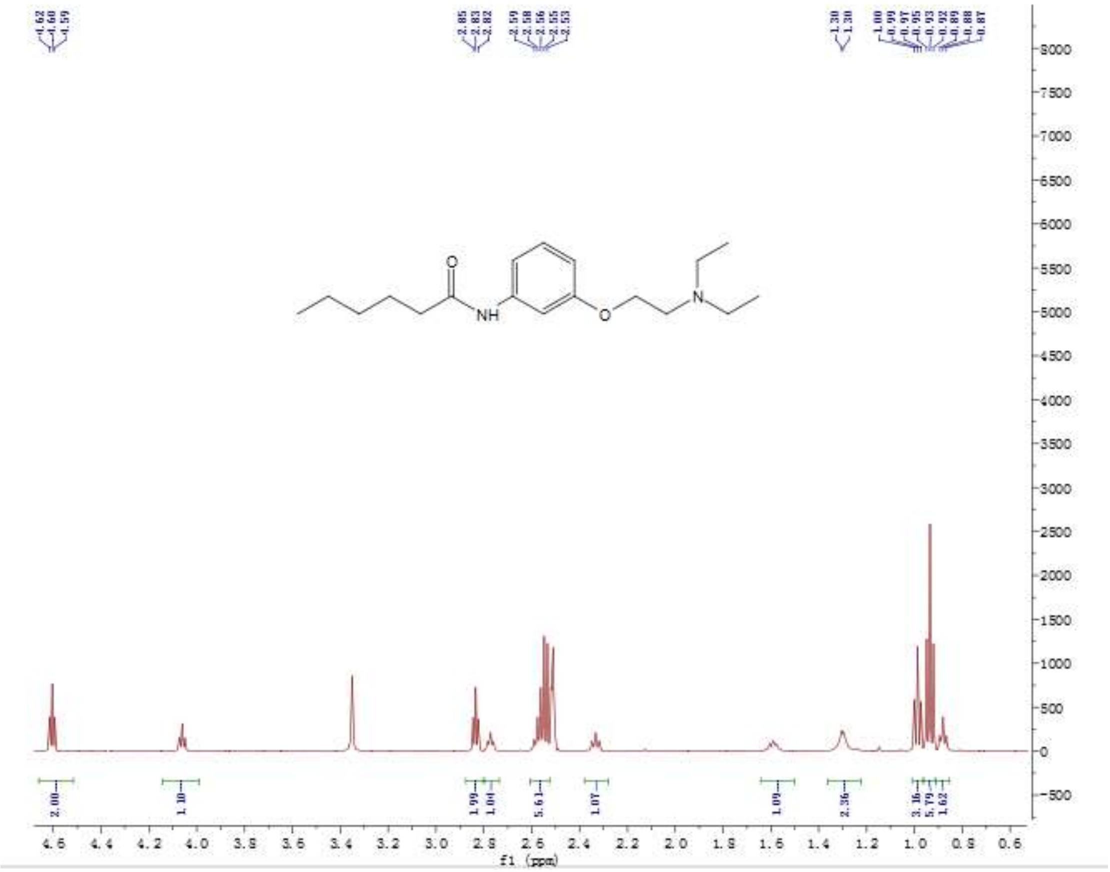

The ^{1}H-NMR (400MHz, DMSO-d_6) of compound **23e**

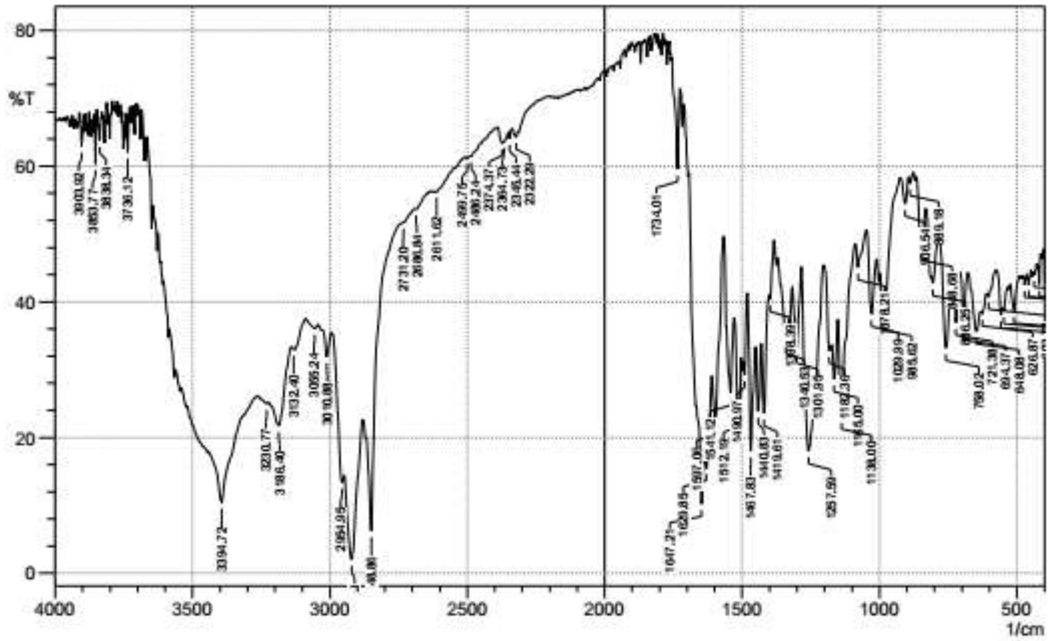

The IR spectra of compound **23e**

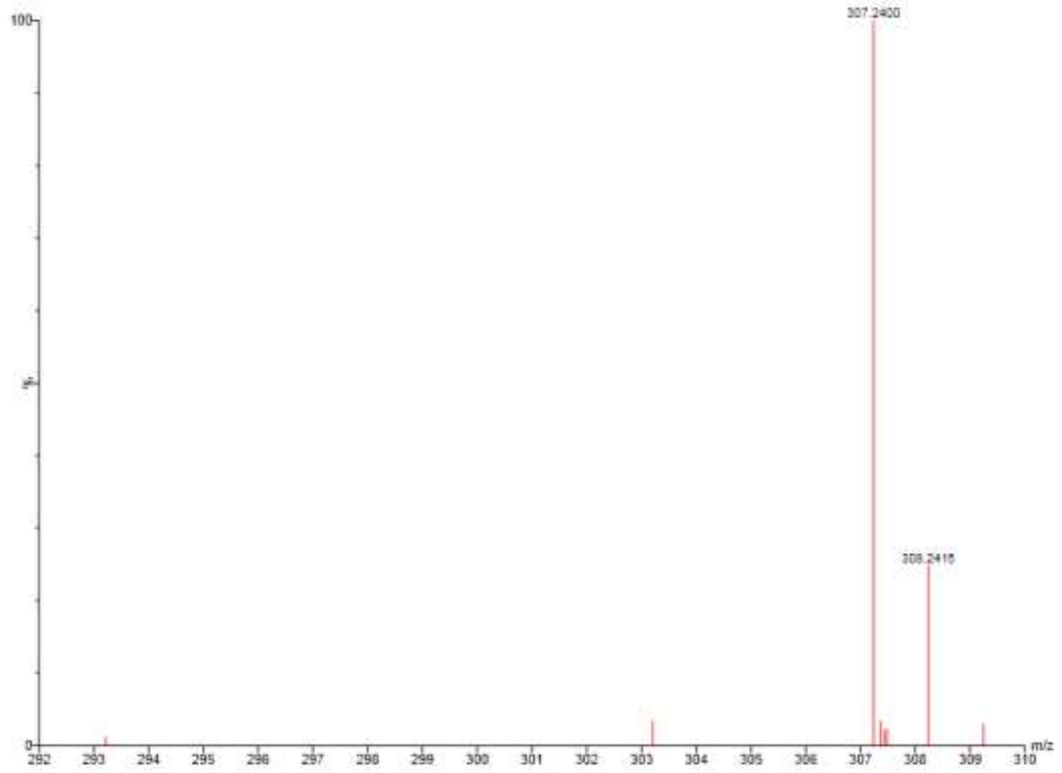

The HRMS of compound **23e**

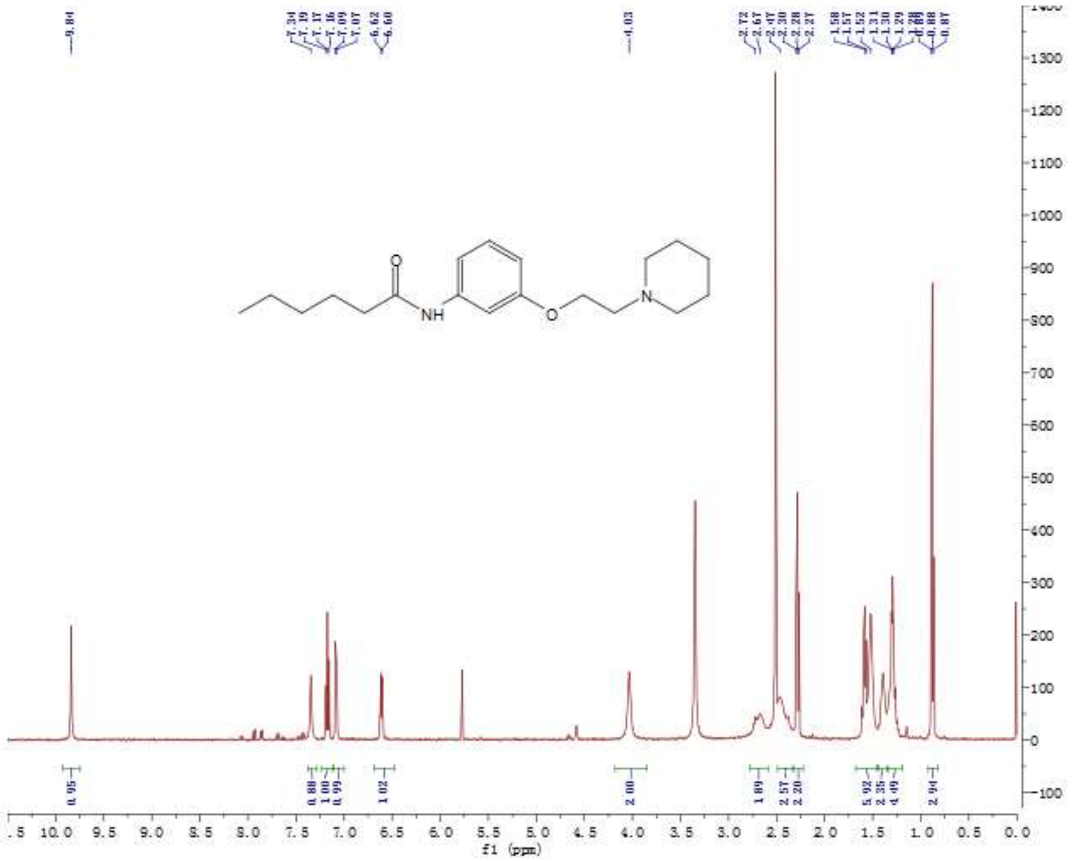

The ^{1}H-NMR (400MHz, DMSO-d_6) of compound **23f**

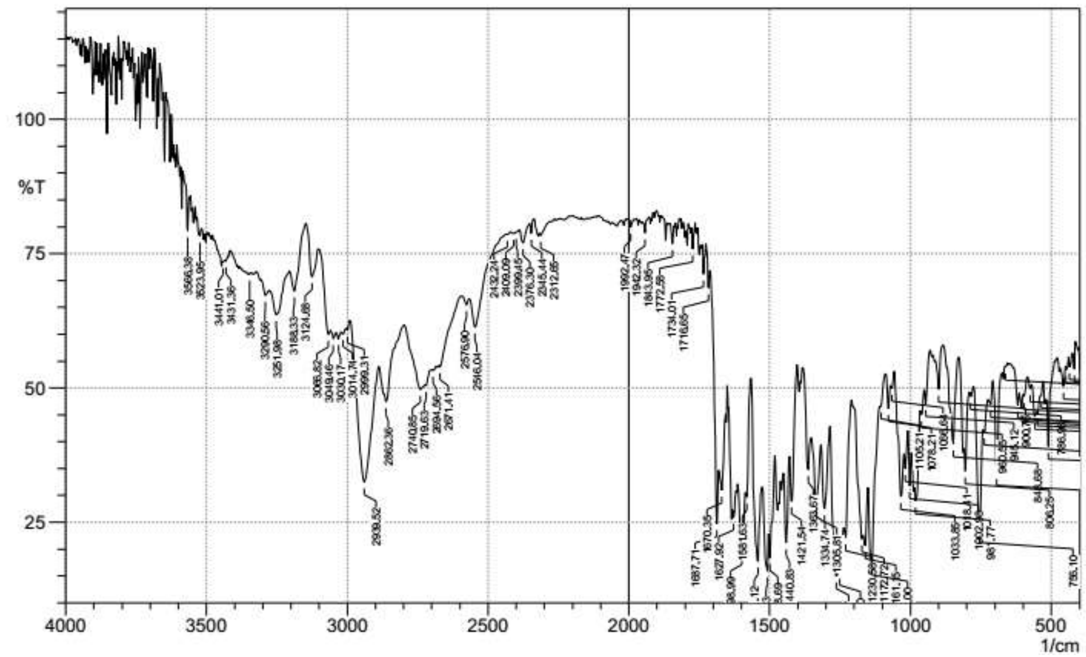

The IR spectra of compound **23f**

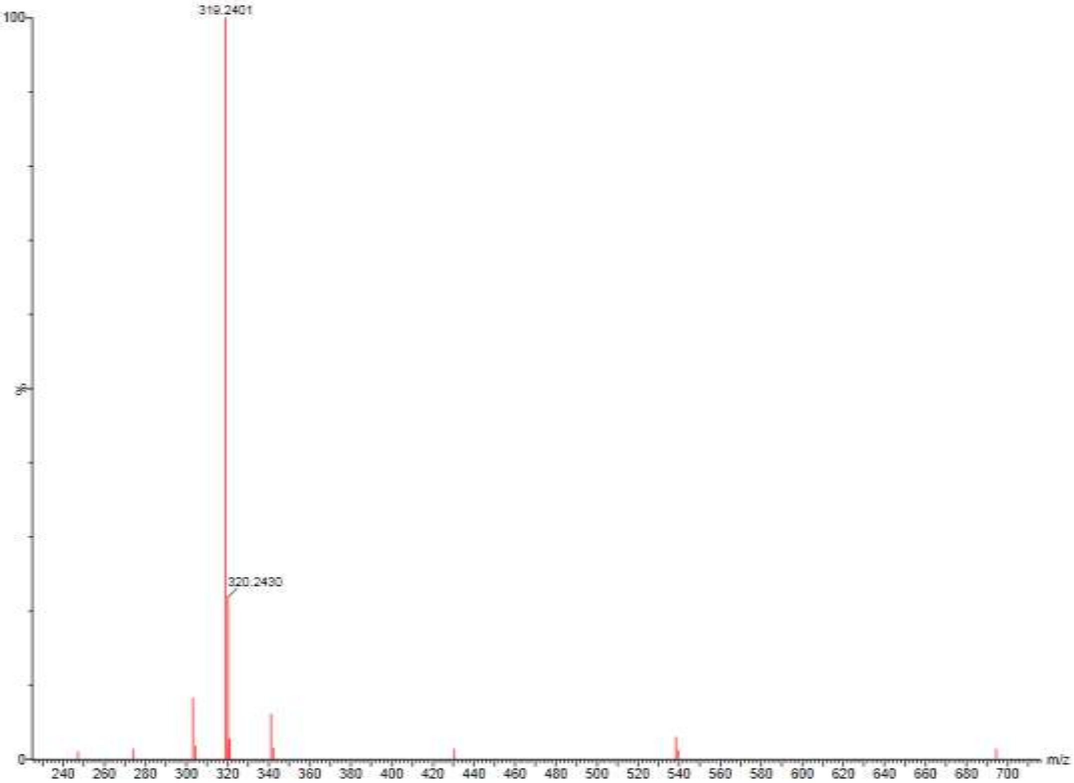

The HRMS of compound **23f**

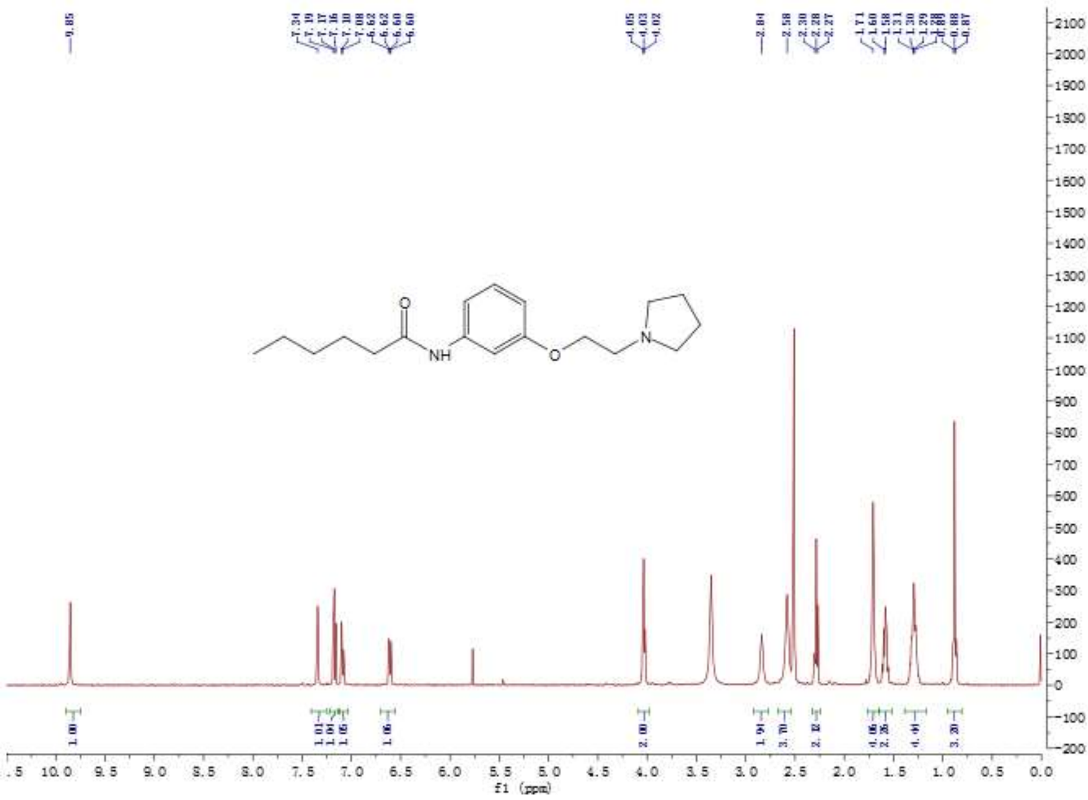

The ^{1}H-NMR (400MHz, DMSO-d_6) of compound **23g**

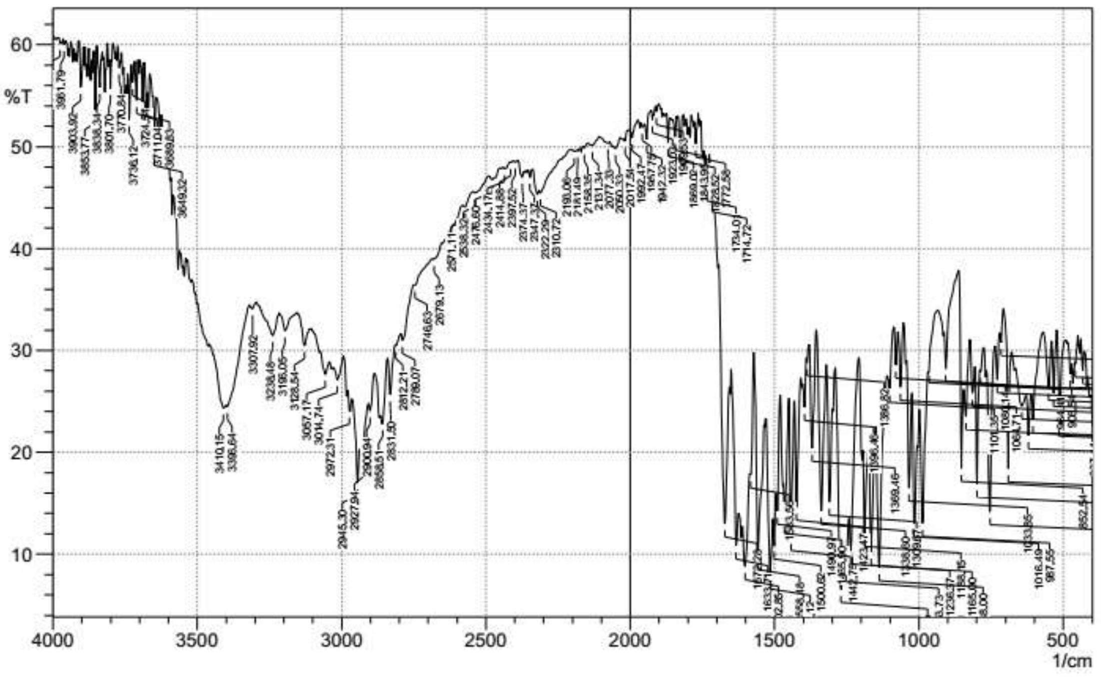

The IR spectra of compound **23g**

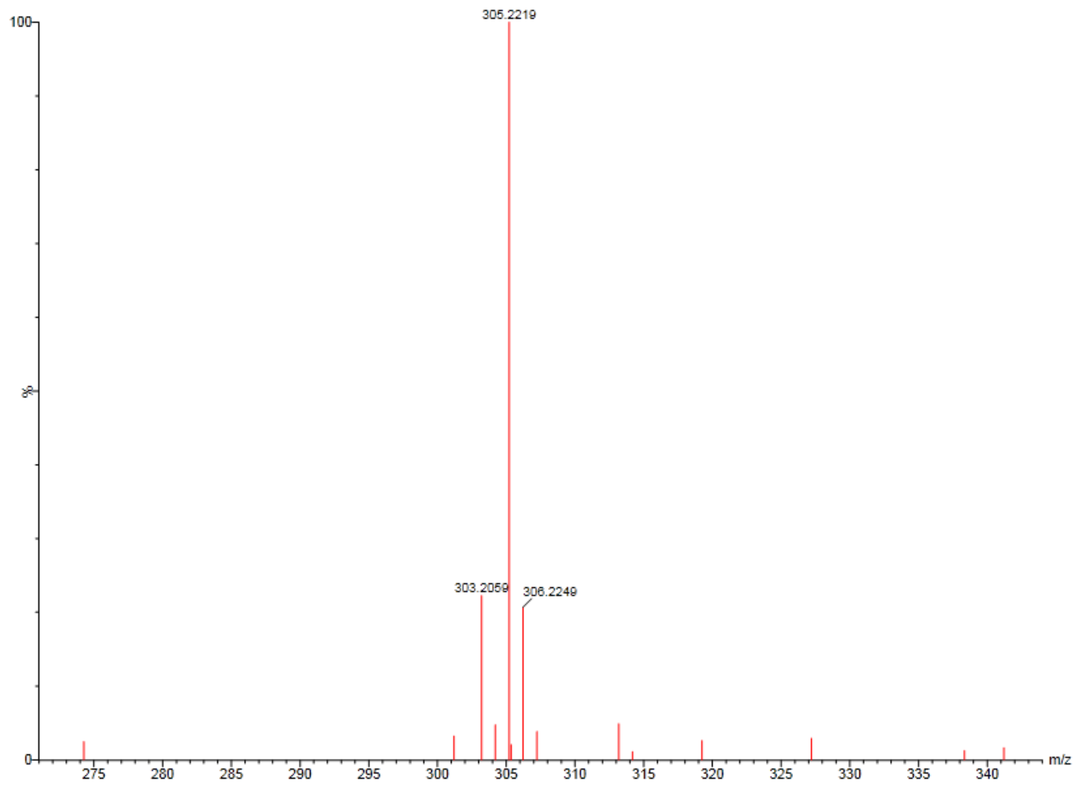

The HRMS of compound **23g**

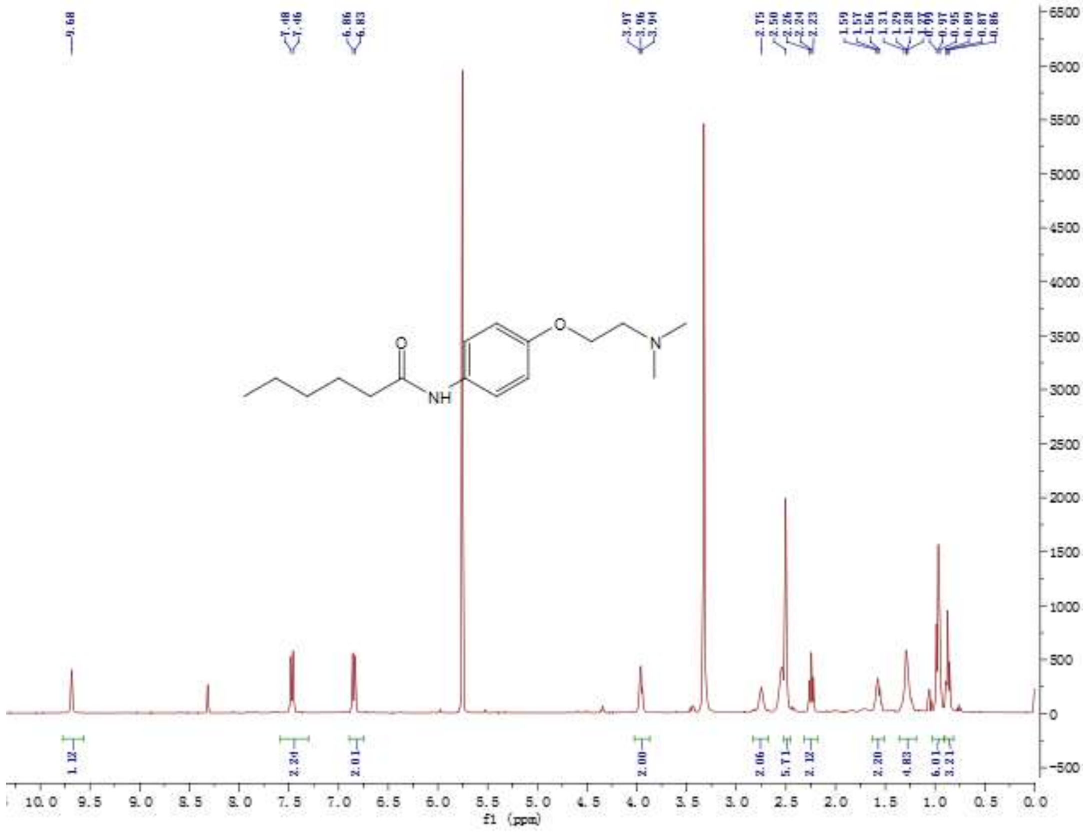

The ^{1}H-NMR (400MHz, DMSO-d_6) of compound **24d**

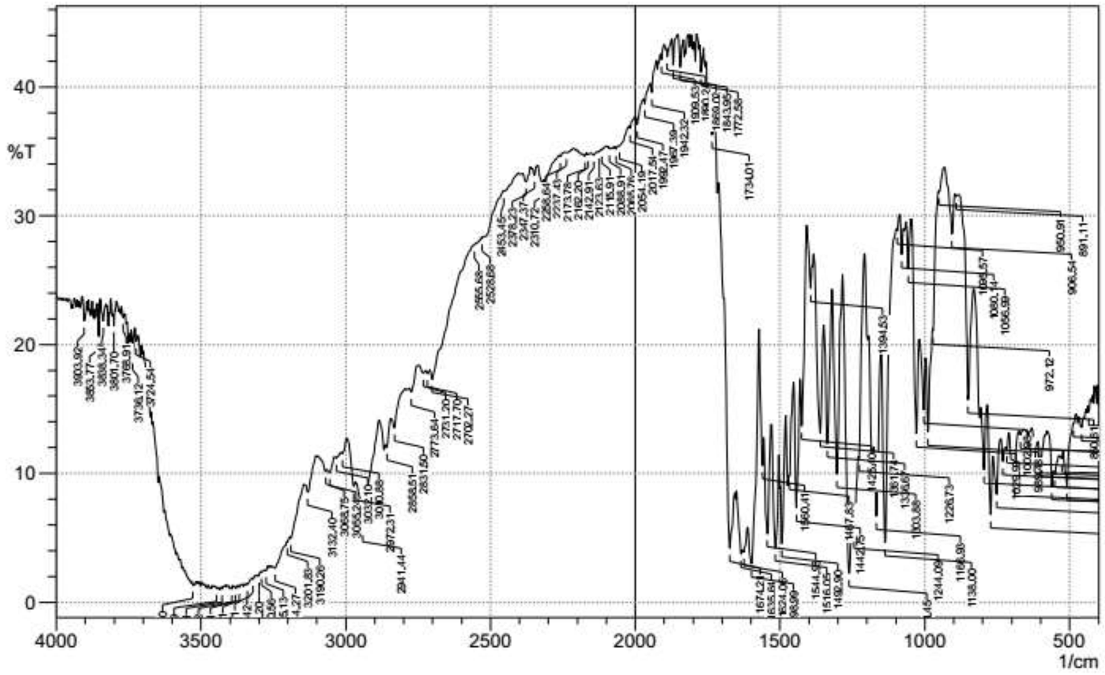

The IR spectra of compound **24d**

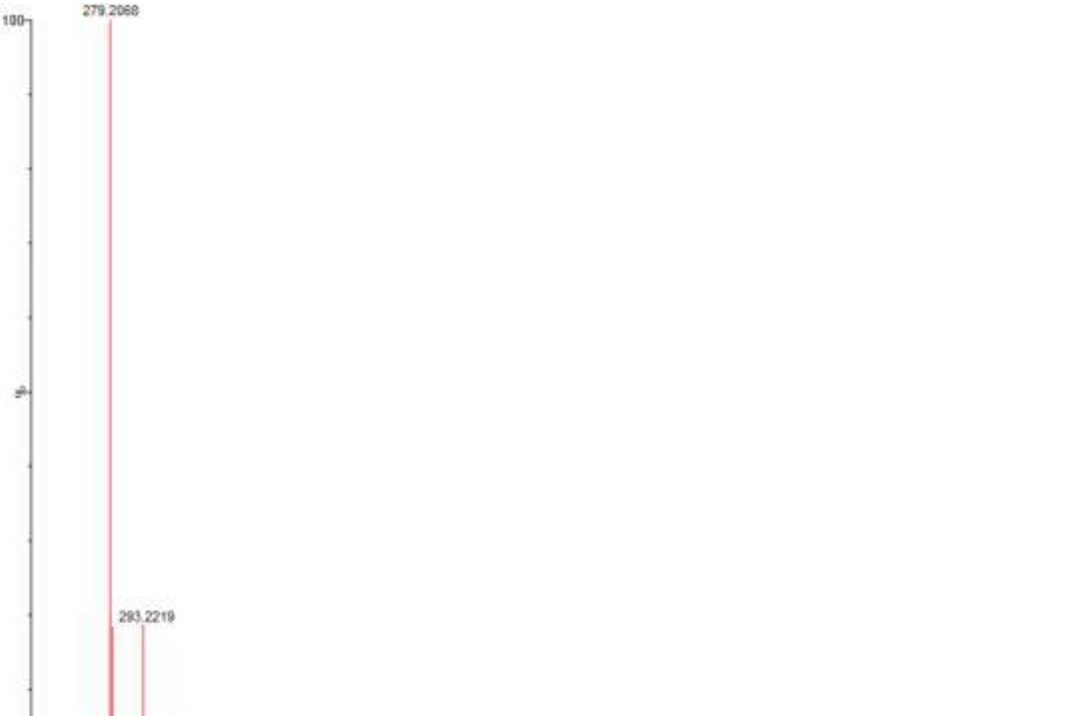

The HRMS of compound **24d**

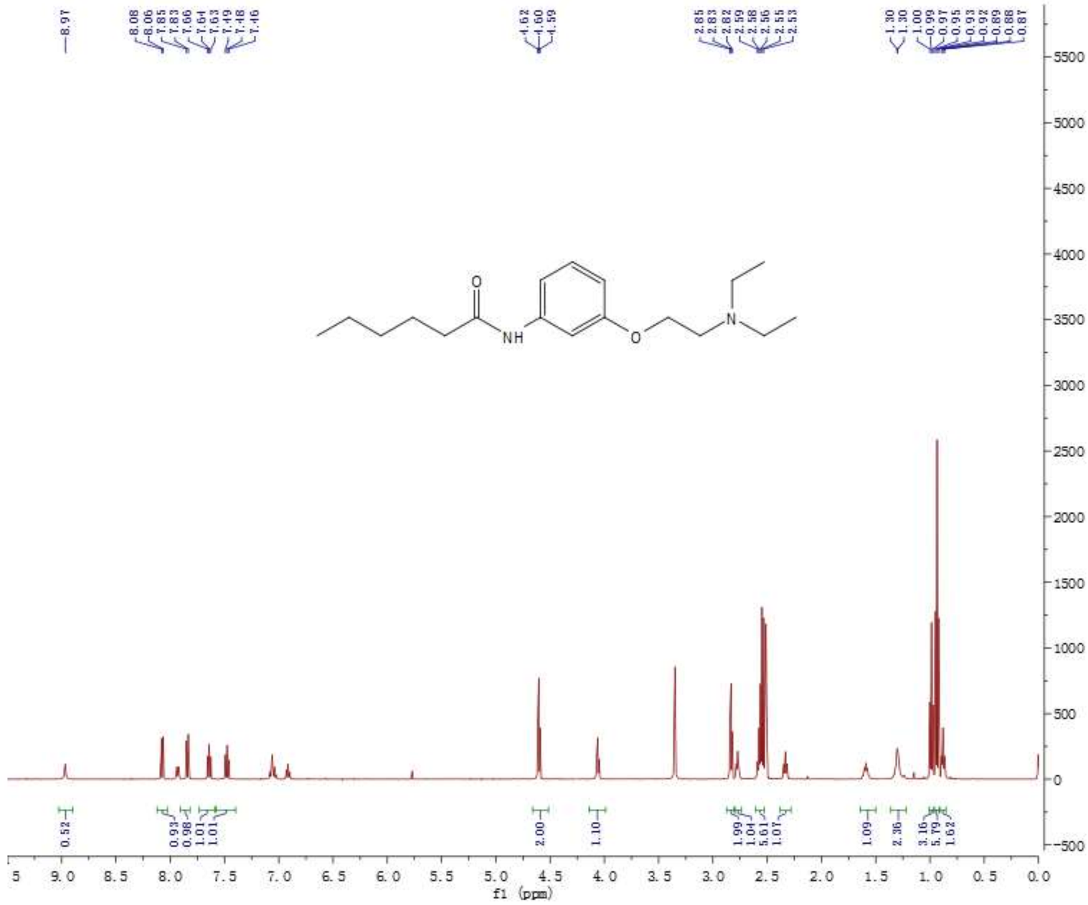

The ^{1}H-NMR (400MHz, DMSO-d_6) of compound **24e**

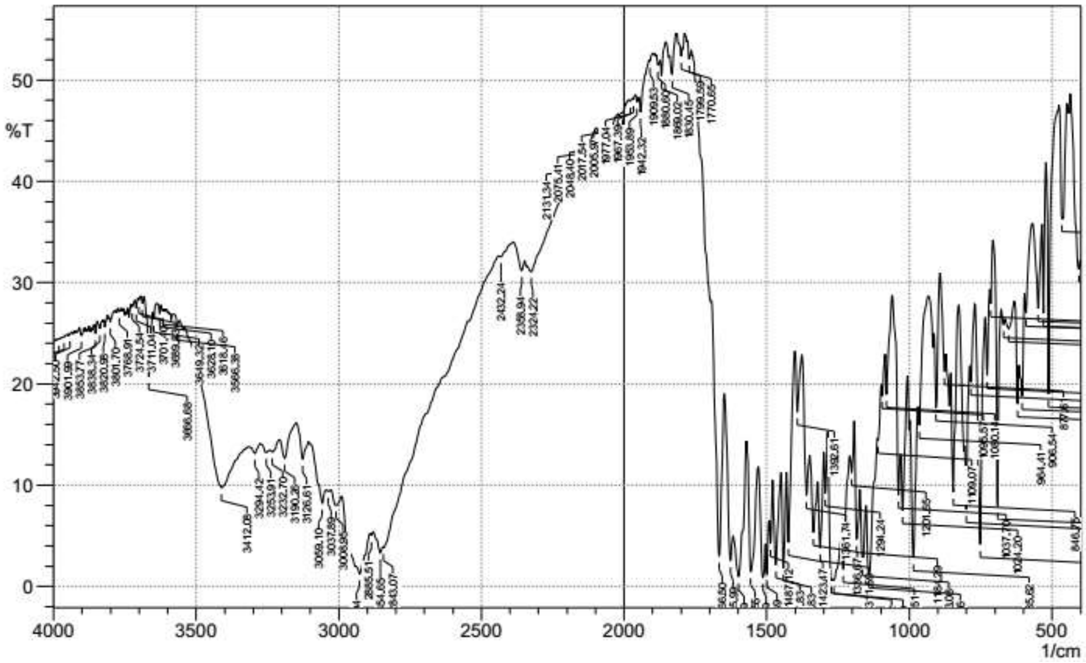

The IR spectra of compound **24e**

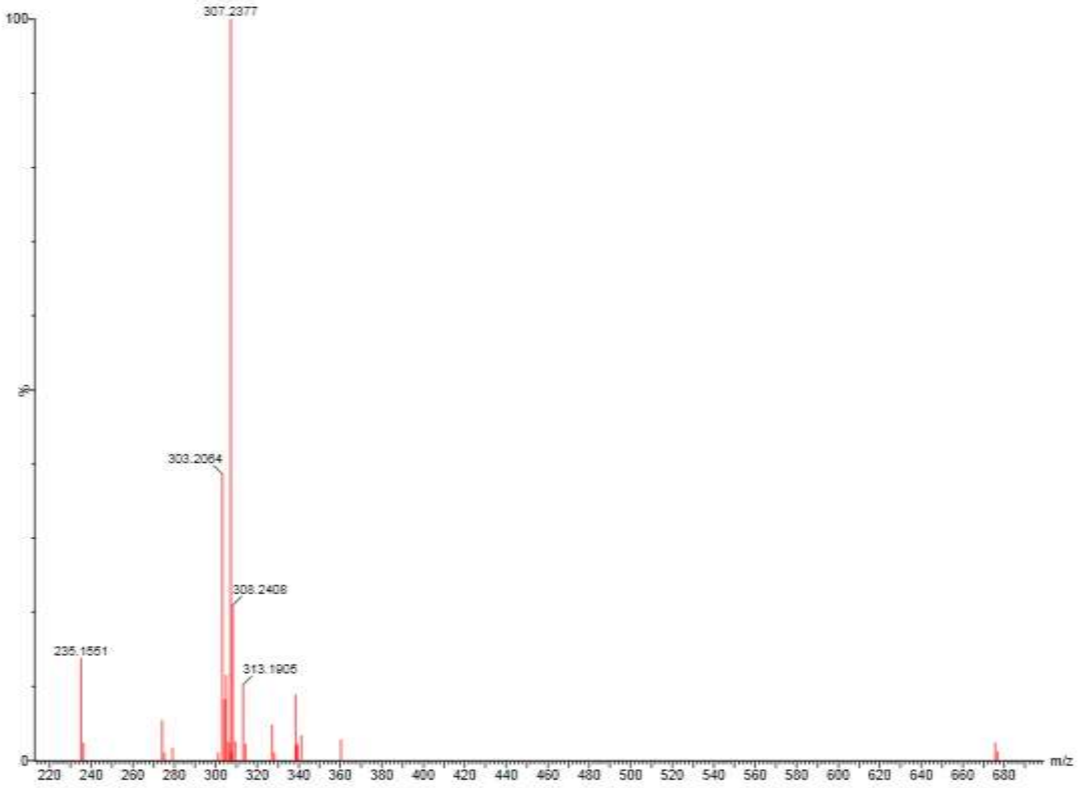

The HRMS of compound **24e**

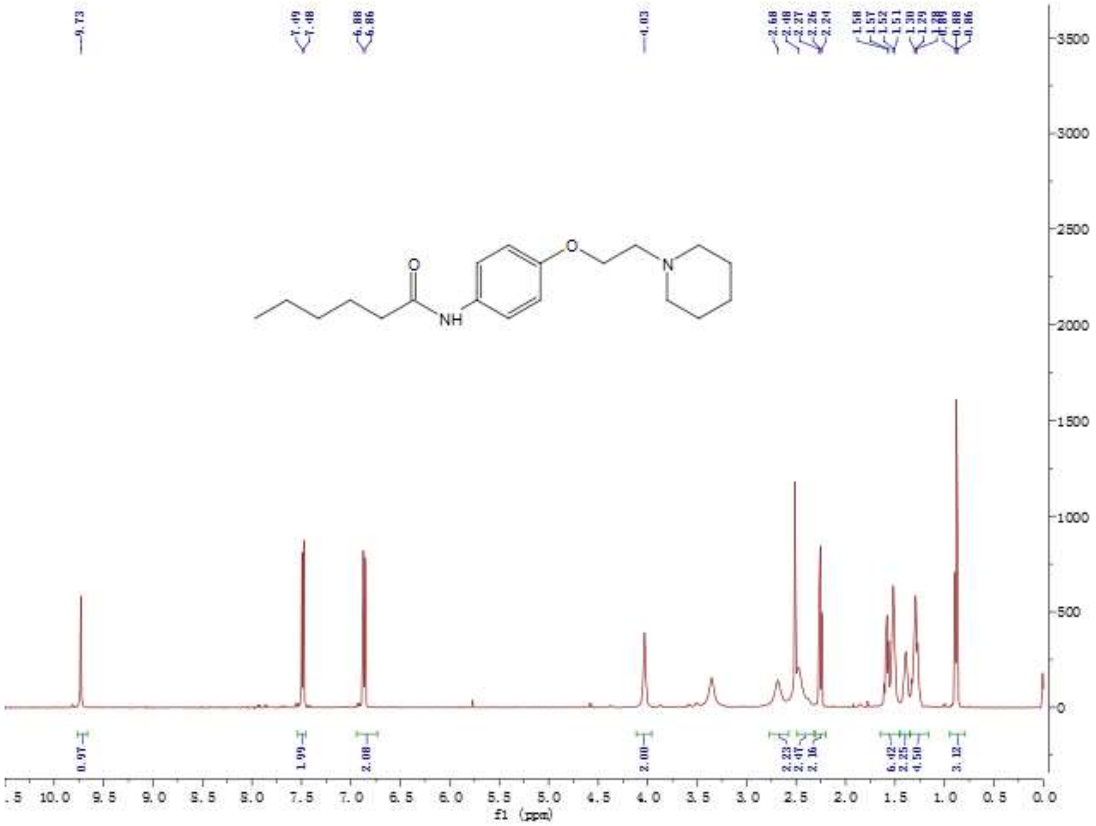

The ^{1}H-NMR (400MHz, DMSO-d_6) of compound **24f**

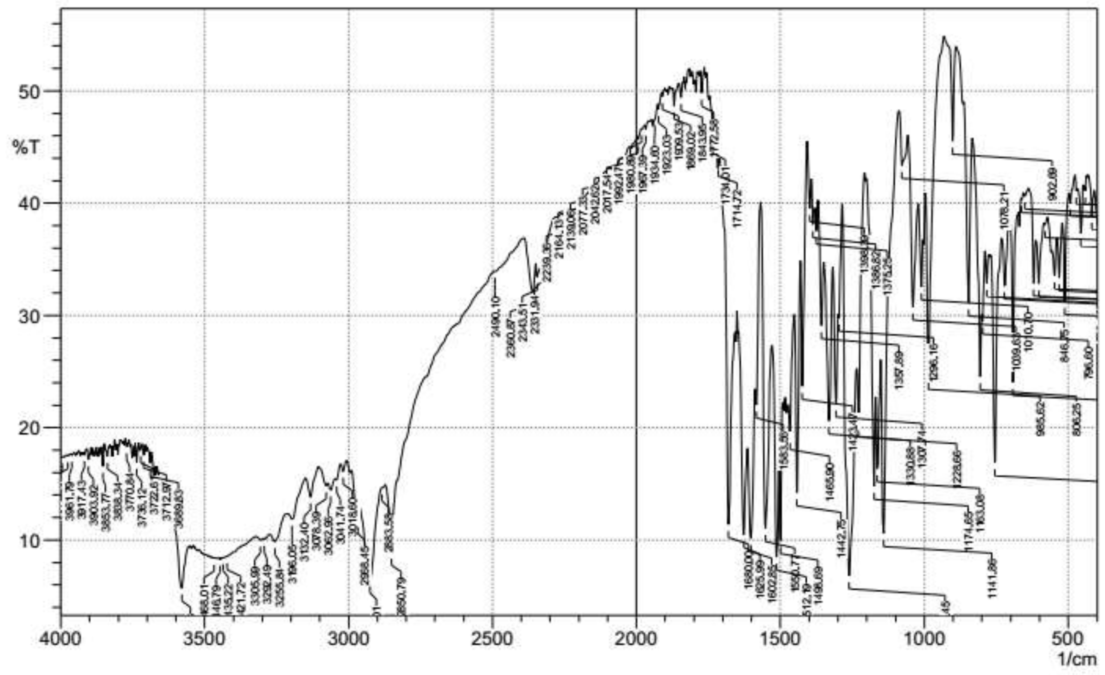

The IR spectra of compound **24f**

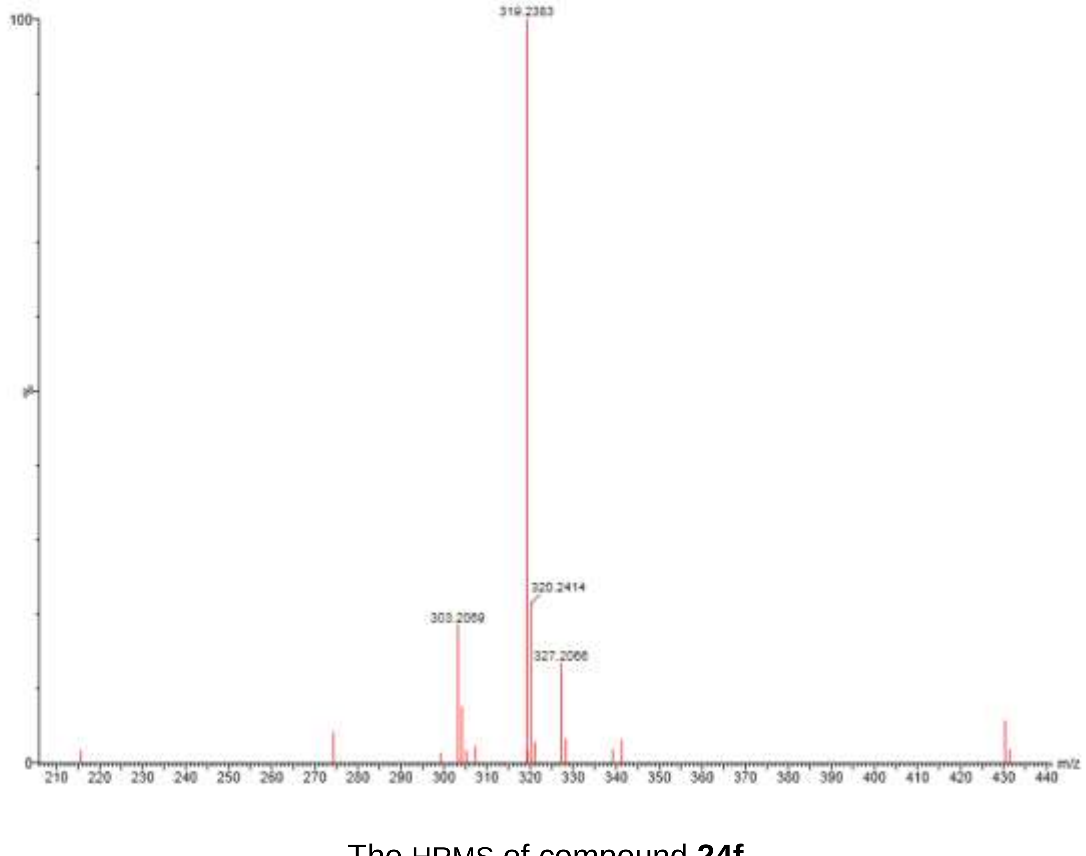

The HRMS of compound **24f**

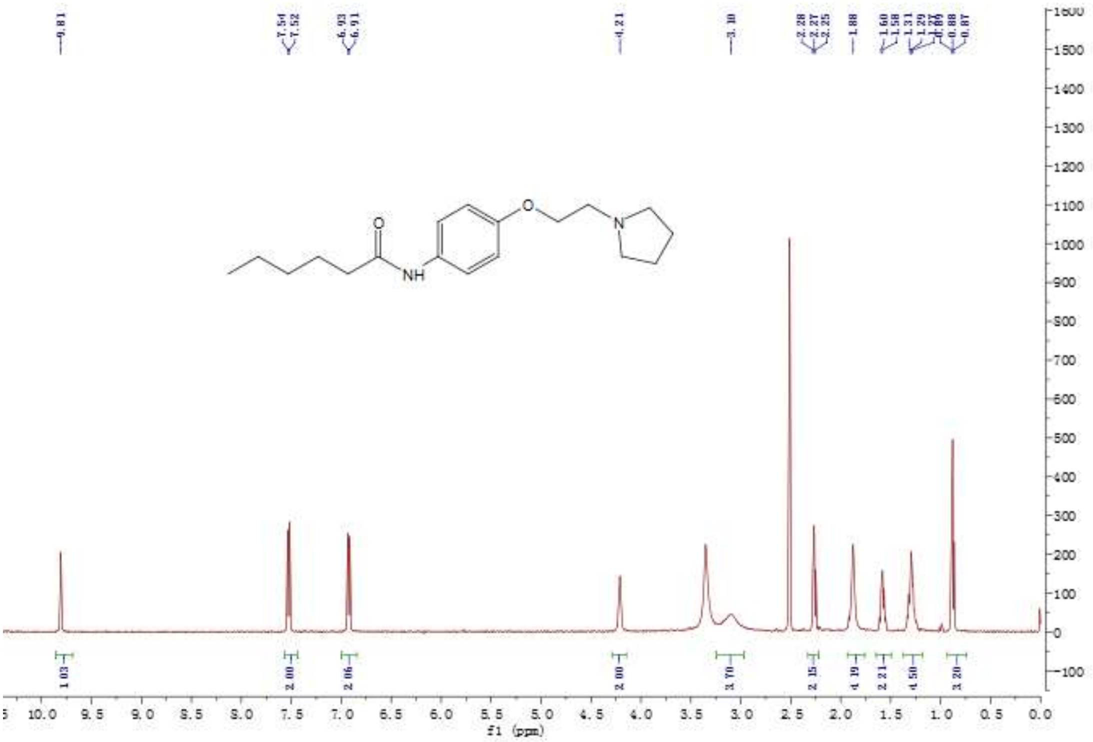

The ^{1}H-NMR (400MHz, DMSO-d_6) of compound **24g**

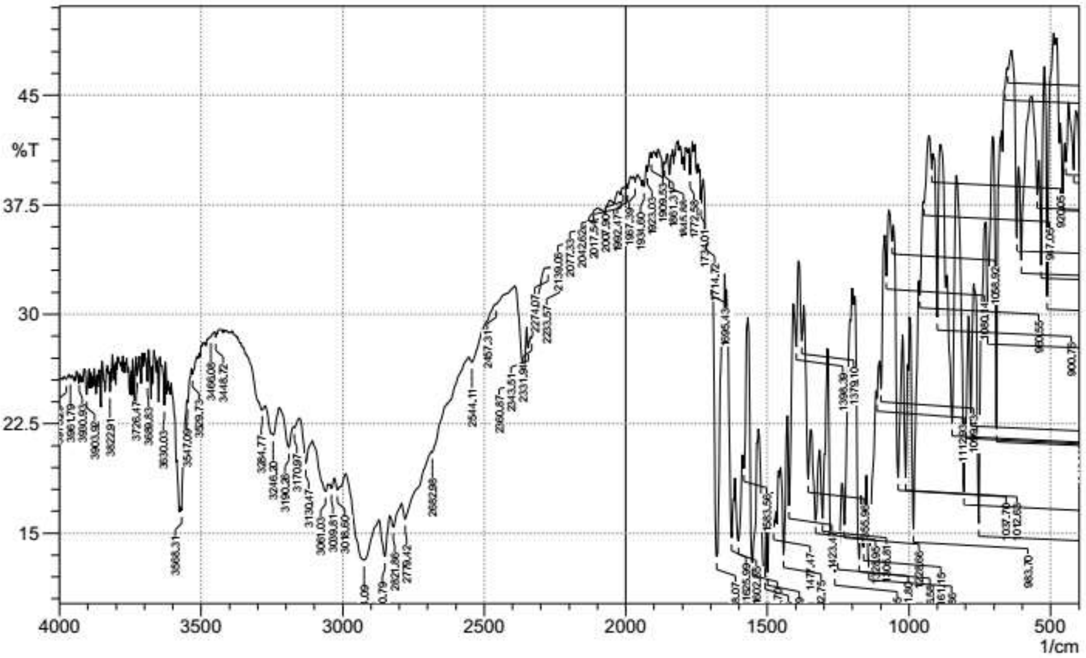

The IR spectra of compound **24g**

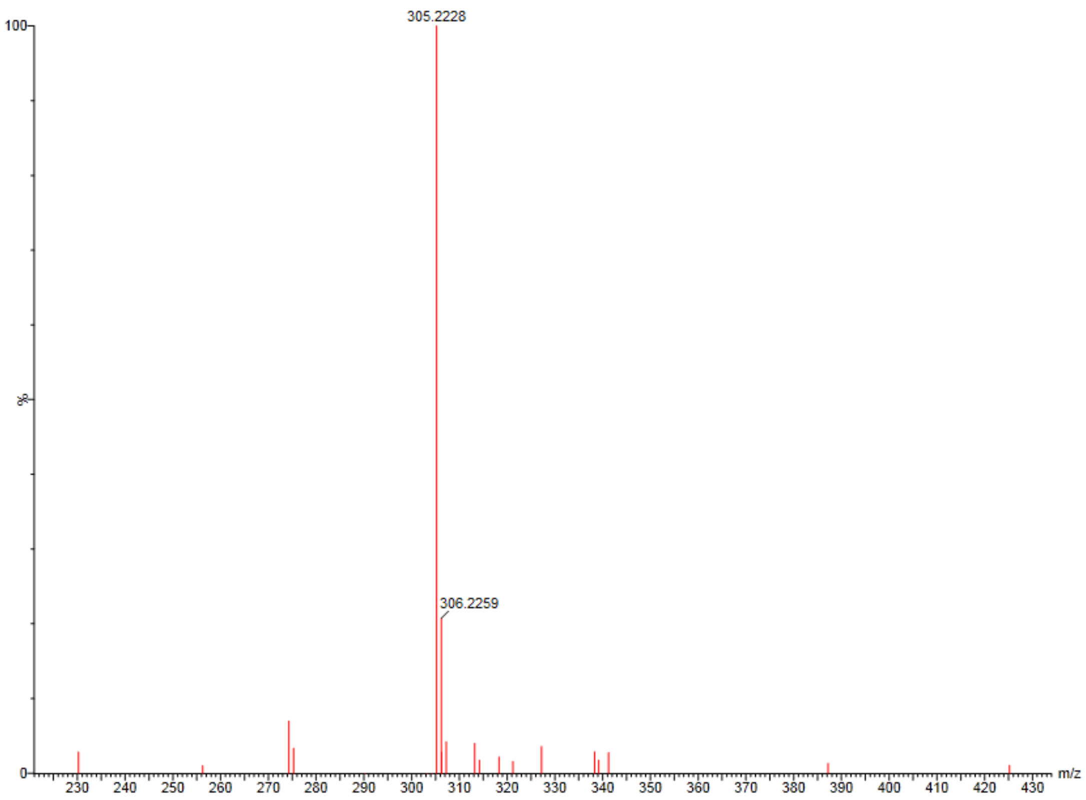

The HRMS of compound **24g**
